# Unravelling structure–function interactions between fluorinated heparan sulfate mimetics and signaling proteins†

**DOI:** 10.1039/d5cb00174a

**Published:** 2025-07-10

**Authors:** Virendrasinh Mahida, Rakesh Raigawali, Paula González, Ana Gimeno, Shani Leviatan Ben-Arye, Saurabh Anand, Sandhya Mardhekar, Jesús Jiménez-Barbero, Vered Padler-Karavani, Raghavendra Kikkeri

**Affiliations:** a Department of Chemistry, Indian Institute of Science Education and Research Pune 411008 India rkikkeri@iiserpune.ac.in; b CIC bioGUNE, Basque Research Technology Alliance, BRTA, Bizkaia Technology park 48160 Derio Spain jjbarbero@cicbiogune.es; c Ikerbasque, Basque Foundation for Science 48009 Bilbao Spain; d Dept. Organic Chemistry II, Faculty of Science and Technology, UPV-EHU 48940 Leioa Spain; e Department of Cell Research and Immunology, The Shmunis School of Biomedicine and Cancer Research, The George S. Wise Faculty of Life Sciences, Tel Aviv University Tel Aviv 69978 Israel vkaravani@tauex.tau.ac.il

## Abstract

Fluorinated carbohydrates are emerging scaffolds in glycobiology, enabling the elucidation of the roles of the individual hydroxyl groups of a carbohydrate in protein binding and drug discovery. Herein, we report a divergent strategy to synthesize seven heparan sulfate (HS) mimetics featuring a fluorine atom at the C3 position of the glucuronic acid residue, with the objective of modulating structure–function relationships. The sensitivity of fluorine signals to sulfation patterns was confirmed *via*^19^F-NMR spectroscopy, while ^3^*J*_HH_ coupling and NOE data demonstrated that the glucuronic acid residue retained its ^4^C_1_ conformation. Glycan microarray analysis and SPR binding studies revealed that a single hydroxyl-to-fluorine substitution in HS mimetics retains the binding of *N*-acetylated HS sequences for several growth factors and chemokines. Remarkably, GlcNAc6S-GlcA(3F) and GlcNS6S3S-GlcA(3F) exhibited binding properties comparable to those of highly *N*-sulfated native HS ligands. These findings provide valuable insights for the development of novel therapeutic agents targeting morphogens and cell signalling pathways.

## Abstract 2

Fluorinated carbohydrates are emerging scaffolds in glycobiology, enabling the elucidation of the roles of the individual hydroxyl groups of a carbohydrate in protein binding and drug discovery.

## Graphical abstract

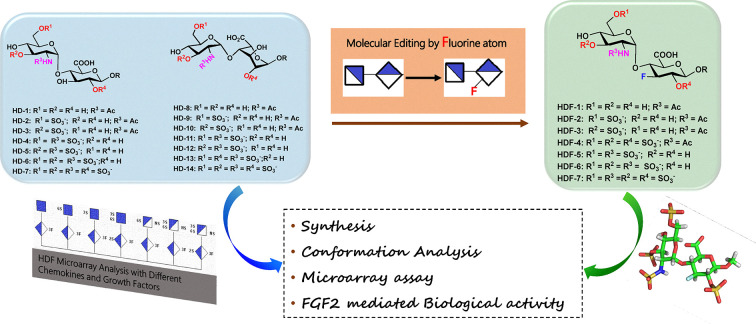

Heparan sulfate (HS) is an anionic polysaccharide that interacts with a wide variety of proteins, orchestrating cell signalling and disease progression.^1^ Routinely, HS binds to growth factors and regulates cell signalling pathways that drive processes like cell proliferation, differentiation, and angiogenesis.^2^ It also binds to chemokines, facilitating immune surveillance and tissue repair.^3^ Viruses, such as SARS-CoV-2, HSV-1, and dengue virus, exploit HS as a receptor for infection or a co-receptor to evade the immune system.^4^ These striking properties make HS ligands attractive targets for therapeutic and diagnostic applications.

HS structures comprise repeating disaccharide units of glucosamine and uronic acids, most notably the conformationally flexible l-iduronic acid. The HS chains display considerable diversity in both sulfation patterns and chain lengths,^1–3^ creating millions of distinct structural variants.^5^ However, structure–function relationship studies with synthetic HS glycans revealed that a single HS structure is often bound by several different proteins, limiting its usage in diagnostics or therapy. For example, 3-*O*-sulfated HS oligosaccharides with uronic acids participate in anticoagulation activity, neurite growth factor binding, and HSV-1 virus attachment.^6^ Likewise, *N*-sulfated l-iduronic acid-based HS glycans are critical for binding to VEGF, but also for binding to chemokines such as CCL2 and CCL5, and the SARS-CoV-2 spike protein.^7^ To address these limitations, HS mimetics are synthesized, where isostructural sugars are substituted on the HS backbone to alter the biological activities. For example, l-iduronic acid in the idraparinux drug was substituted with d-glucuronic acid, d-xylose, 6-deoxy-l-talose, and even ^1^C_4_ and ^2^S_0_-conformation locked l-iduronic acid moieties to study the anticoagulant activity of this drug.^8^ Alternatively, molecular editing of hydroxyl groups with fluorine atoms has been extensively employed in carbohydrate chemistry to develop glycomimetics. Replacing C–OH bonds with C–F bonds in glycans induces several beneficial effects, including enhanced lipophilicity, improved cellular permeability, and increased stability against hydrolytic cleavage of glycosidic bonds.^9^ The bioisosteric replacement of hydroxyl groups by fluorine atoms preserves the hydrogen acceptor nature of carbohydrates, while the ionic nature of the C–F(δ^−^) bond stabilizes electrostatic interactions with adjacent electropositive groups, potentially modulating carbohydrate–protein interactions.^9*h*^ Furthermore, the incorporation of fluorine enables ^19^F-NMR monitoring, facilitating direct detection of metabolic stability and carbohydrate–protein interactions.^10^ Recent studies on GM_1_, galectin-specific ligands, and Lewis X glycan that were systematically modified with fluorine atoms have demonstrated the potential of fluorination to fine-tune carbohydrate–protein interactions.^9*e*–*g*,10^ Therefore, fluorinated HS mimetics are postulated to fine-tune the microenvironment of carbohydrate–protein interactions, thereby enabling the development of small, selective ligands targeting HS-binding proteins. Herein, we present the design, synthesis and conformational analysis of seven fluorinated HS disaccharide mimetics and native HS disaccharides (Fig. 1), followed by high-throughput glycan microarray binding studies of several growth factors and chemokines. Disaccharide analogs were selected for this initial proof-of-concept study to optimize the synthetic methodology and investigate how minimal structural units can reflect binding variations with growth factors and chemokines, driven by differences in sulfation patterns and the uronic acid configuration.^14,15*a*^ Our results demonstrate that the incorporation of fluorine atoms within the *N*-acetate domains of HS mimetics preserves their binding preferences and, in some cases, also leads to increased binding when compared to their *N*-sulfated counterparts. Comprehensive conformational analyses, molecular docking simulations, surface plasmon resonance (SPR) assays, fibroblast growth factor 2 (FGF2)-induced cell proliferation studies, and mitogen-activated protein kinase (MAPK) signalling assays collectively support these observations. Together, our findings underscore the potential of fluorinated HS analogues as next-generation tools for therapeutic and diagnostic applications, offering a novel avenue for targeting HS-mediated biological processes with improved precision.

**Fig. 1:**
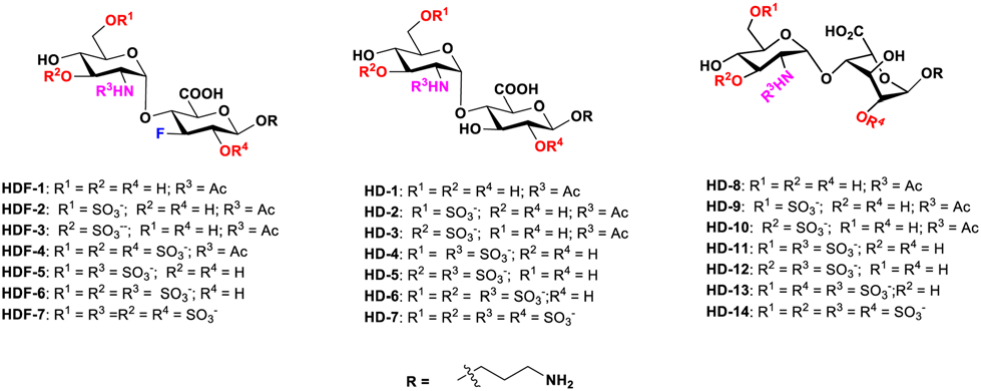
Chemical structures of 7-fluorinated d-glucuronic acid based HS mimetics (HDF-1 to HDF-7) and 14 native HS disaccharides (d-glucuronic acid based HD-1 to HD-7 and l-iduronic acid based HD-8 to HD14).

## Results and discussion

**Scheme 1:**
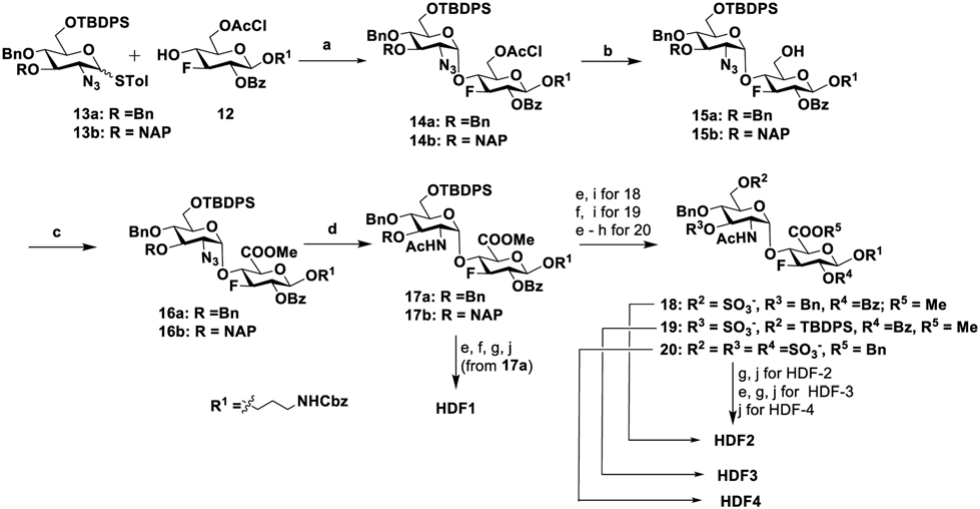
Synthesis of glucuronic acid based HS disaccharides: (a) NIS, TMSOTf, DCM, −78 °C, 15 min; (b) thiourea, Py : MeOH (1 : 1), 80 °C, 2 h; (c) (i) TEMPO, BAIB, DCM : H_2_O(1 : 1), RT; (ii) MeI, K_2_CO_3_, DMF, 6 h, RT; (d) Zn dust, THF : AcOH : Ac_2_O (3 : 2 : 1), RT, 12 h; (e) HF.Py, Py, 0 °C, 12 h; (f) DDQ, DCM : H_2_O (18 : 1), RT, 1 h; (g) LiOH, H_2_O : THF(1 : 1), RT, 12 h; (h) BnBr, NaHCO_3_, DMF, 60 °C, 2 h; (i) SO_3_.Et_3_N, DMF, 60 °C, 48–72 h; and (j) H_2_, Pd(OH)_2_, H_2_O, RT, 48 h.

Fluorinated HS disaccharides with regioselective *N*-acetylated or *N*-sulfated and *O*-sulfated analogues were synthesized from disaccharide building blocks 16a and 16b using a divergent synthetic approach described in Scheme 1. The synthesis of disaccharides 16a/16b required 3-fluoro-3-deoxy glucose 12 and glucosamine building blocks 13a/13b^11^ with orthogonal protecting groups to control regioselective glycosylation and sulfation patterns. The 3-fluoro-3-deoxy-d-glucose building block 12 was derived from 1,2 : 5,6-*O*-isopropylidene-α-d-allofuranose as the starting material, employing a standard procedure (Scheme 1).^9*d*^ Glycosylation of donor 13a/13b with acceptor 12 in the presence of NIS and TMSOTf yielded the α-disaccharides 14a and 14b, respectively. Mild thiourea and pyridine-catalyzed deprotection of chloroacetate groups, followed by oxidation using the catalytic 2,2,6,6-tetramethylpiperidinyloxyl (TEMPO) free radical in the presence of excess [bis(acetoxy)iodo]benzene (BAIB), was carried out. This was followed by methyl iodide and potassium bicarbonate-mediated esterification, resulting in disaccharides 16a and 16b, respectively (Scheme 1).

**Scheme 2:**
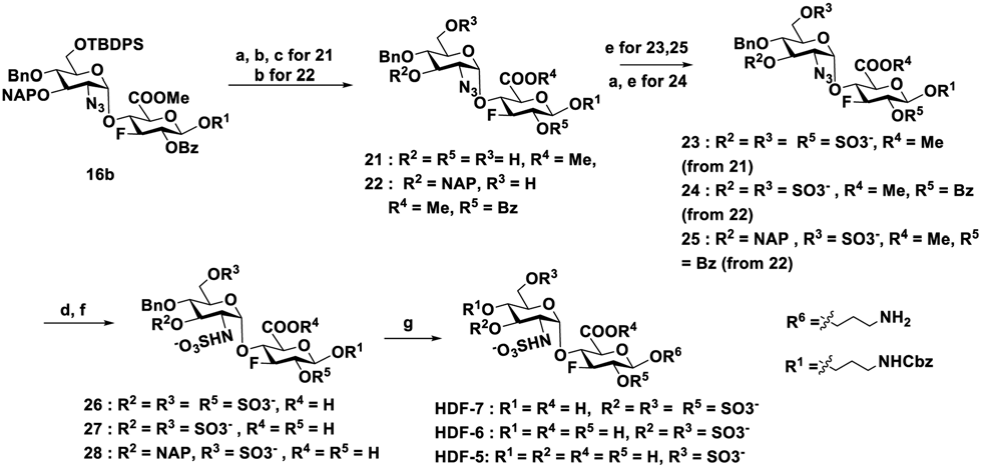
Synthesis of fluorinated *N*-sulfated HS disaccharides: (a) DDQ, DCM : H_2_O (18 : 1), RT, 1 h; (b) HF.Py, Py, 0 °C, 12 h; (c) NaOMe, MeOH, RT, 6 h; (d) LiOH, H_2_O : THF(1 : 1), RT, 12 h; (e) SO_3_.Et_3_N, DMF, 60 °C, 48–60 h; (f) (i) PMe_3_.THF, RT, 24 h; (ii) SO_3_.Py. MeOH, 1 M NaOH, 0 °C, 48 h; and (g) H_2_, Pd(OH)_2_, H_2_O, RT, 48 h.

Next, TBDPS or NAP groups were selectively removed using 70% HF.Py or DDQ, followed by sulfation with the SO_3_-TEA complex to afford 6-*O*-sulfated and 3-*O*-sulfated derivatives 18 and 19, respectively. Subsequently, lithium hydroxide mediated ester hydrolysis and Pd(OH)_2_ catalyzed hydrogenolysis yielded HDF2 and HDF3. Cleavage of TBDPS, Bz, and 2-NAP groups of 16a/16b, followed by either non-sulfation or sulfation using the SO_3_–TEA complex, and subsequent global deprotection yielded HDF1 and HDF4, respectively (Scheme 1). For the *N*-sulfated series, 16b underwent chemoselective cleavage of TBDPS, Bz, and 2-NAP groups, followed by *O*-sulfation, and subsequently, the azide group was converted to amines using trimethylphosphine. *N*-Sulfation was achieved using the SO_3_.Py complex, followed by global deprotection (Scheme 2). The non-fluorinated HS disaccharides comprising of d-glucuronic acid (HD-1 to HD-7) and l-iduronic acid based (HD-8 to HD-14) were synthesized and characterized using a divergent strategy, as previously described.^11*d*^

**Fig. 2:**
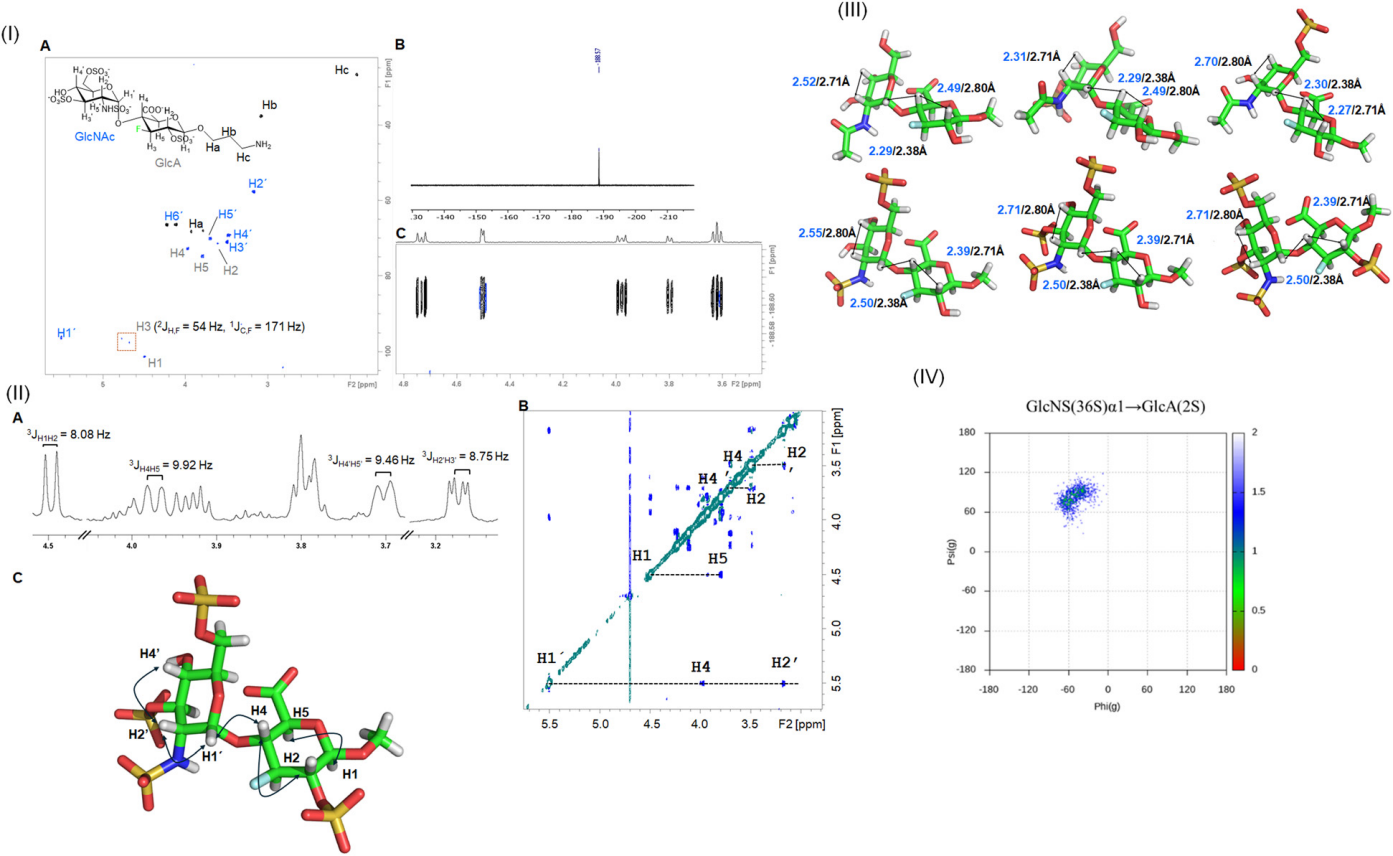
Structural characterization of HDF-7: (i) NMR-based characterization of compound HDF-7. (A) ^1^H–^13^C-HSQC spectrum. ^1^H-resonance assignment has been annotated: blue for GlcNAc, gray for GlcA, and black for the linker. (B) 1D ^19^F-NMR spectrum of HDF-7. (C) 2D ^19^F-relay-[H]H TOCSY spectrum; (ii) conformational analysis of compound HDF-7. (A) Expansion of the ^1^H-NMR spectrum, showing the key ^1^H resonances, where ^3^*J*_HH_ values (Hz) have been indicated. (B) 2D NOESY spectra recorded for HDF-7. Relevant correlations are annotated. (C) Representative 3D model of HDF-7, generated using GLYCAM-Web tools and validated by NOE data; (iii) molecular models of fluorinated HS disaccharides. Key NOE-derived inter-proton distances (blue) are compared with theoretical values (black): (a) HDF-1, (b) HDF-2, (c) HDF-3, (d) HDF-5, (e) HDF-6 and (f) HDF-7; (iv) docking and conformational analysis of the GlcNS(3S,6S)β1-4-GlcA(2S) disaccharide performed by MD simulations. Plots of *ϕ*/*ψ* values explored along the 100 ns MD trajectory are displayed. The points are colored as a function of the population density.

The conformation of the fluorinated HS disaccharides with different sulfation patterns was analyzed using NMR spectroscopy. Due to the high structural similarity of the six fluorinated compounds synthesized, representative NMR analysis of HDF-7 is detailed in Fig. 2(i) and (ii). The NMR spectrums of HDF1-3 and HDF5-6 and information of all analyzed compounds are included in Sections S4–S6, ESI.†

All compounds displayed well-dispersed NMR signals, facilitating resonance assignment. While the presence of fluorine was ascertained by 1D ^19^F NMR spectroscopy, the information gathered from HSQC and ^19^F-relay-[H]H TOCSY experiments confirmed its location at the C3 position. The conformation of the studied oligosaccharides was then inferred from the analysis of the vicinal coupling constants (^3^*J*_HH_) and NOE contacts. First, ^3^*J*_HH_, containing information on ring puckering, was extracted from the 1D ^1^H-NMR spectra. In both GlcNAc and GlcA rings, regardless of the sulfation pattern, the H1–H4 protons exhibited ^3^*J*_HH_ values above 8.0 Hz, consistent with the *anti*-arrangement of vicinal protons and, therefore, with the predominance of the ^4^C_1_ conformation. On the other hand, the values determined for ^3^*J*_H1H2_ were in agreement with the α and β configurations of GlcNAc and GlcA rings, respectively (Fig. 2(ii)A).

The global conformations of the disaccharides HDF-1 to HDF-3 and HDF-5 to HDF-7 were investigated using NOESY experiments assisted by computational calculations.^12^ The 2D NOESY spectrum obtained for HDF-7 is shown in Fig. 2(ii)B, as an illustrative example, where key correlations defining the conformation of the disaccharide have been indicated (see Fig. 2(ii) B and C). The molecule displayed positive NOEs, in agreement with its small size and short rotational correlation time, as expected for a disaccharide. In addition, key intra-residue and inter-residue cross-peaks were identified, which allowed defining unequivocally the sugar conformation (Fig. 2(ii)B). Intra-residue NOE cross-peaks between H1–H3, H1–H5 and H5–H3 proton pairs of GlcA were detected, indicative of the ^4^C_1_ conformer. Although some overlapping occurs for GlcNAc protons signals, the NOE cross-peak for the H2–H4 proton pair was also observed, in agreement with the ^4^C_1_ conformation. Fittingly, the inter-residue NOE contact between H1GlcNAc and H4GlcA was clearly detected, which is exclusive for the *exo-syn-ϕ*/*syn-ψ* conformation around the glycosidic linkage. Initial geometries for all compounds were built using the carbohydrate building module in the GLYCAM-Web portal. The disaccharide structures were then modified using the MAESTRO suite of programs to include a fluorine atom at position C3 and to display the corresponding sulfation pattern. Then, they were submitted to an energy minimization process with a low gradient convergence threshold (0.05) in 2500 steps, employing the AMBER force field.

Fittingly, the distances predicted from the molecular modelling approaches (in black) were in full agreement (Fig. 2(iii)) with those estimated experimentally by analysis of the NOEs (in blue), thus validating the modelling protocol. Tables with all experimental and theoretical inter-proton distances for disaccharides are included in the Materials and methods section. Collectively, the ^3^*J*_HH_ values and NOE data are in agreement with the almost exclusive presence of a major conformer for all disaccharides, regardless of the sulfation pattern.

Overall, GlcNAc and GlcA residues behave as single ^4^C_1_ chair conformation, and display the *exo-syn-ϕ*/*syn-ψ* conformation around the glycosidic linkage. Although no experimental data were recorded for the natural disaccharides (HD1-14), molecular dynamics (MD) simulations predicted a similar behavior to those observed for their fluorinated counterparts. In particular, 100 ns MD simulations for the GlcNS(3S,6S)β1-4-GlcA(2S) disaccharide (HD-7 analog) predicted ^4^C_1_ chairs for both pyranose rings and the *exo-syn-ϕ*/*syn-ψ* conformation as the major one around the glycosidic linkage (Fig. 2(iv)). Of note, although the conformation of HDF-4 was not experimentally measured, it is anticipated to exhibit similar behaviour to the other analogues and is therefore not discussed in detail.

Next, to determine the effect of the fluorine substituent on protein binding to the HS disaccharidases, all the 7 HDFs (fluorinated) and 14 HDs (non-fluorinated HS disaccharides) were immobilised onto epoxy coated glycan microarray slides and binding patterns for 7 prominent human HS-binding growth factors and 9 chemokines were investigated as previously described:^10*a*–*c*^ two fibroblast growth factors (FGF1 and FGF2), two epidermal growth factors (EGF and HB-EGF), vascular endothelial growth factor (VEGF), amphiregulin and bone morphogenetic protein (BMP2). Each protein was tested at three different concentrations.

**Fig. 3:**
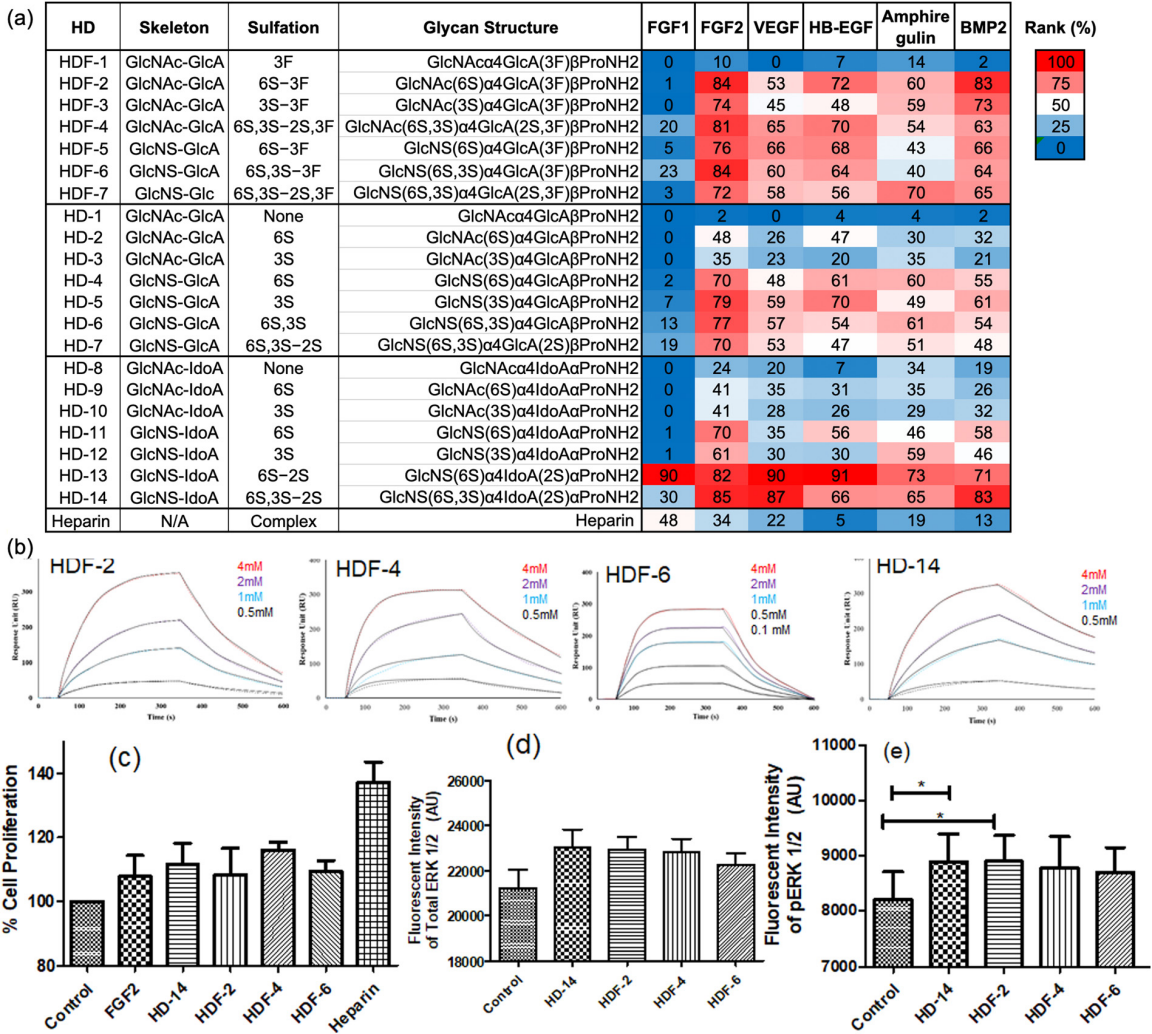
Growth factors binding to HD and HDF mimetics: (a) HS disaccharide mimetics glycan microarray analysis of human FGF1, FGF2, VEGF_195_, HB-EGF, amphiregulin and BMP2. All proteins were tested at three concentrations (10, 3.3, and 1.6 ng μl^−1^), each per sub-array. Binding was tested with biotinylated secondary antibodies and then detected with Cy3-streptavidin. Relative fluorescence units (RFU) were ranked according to the highest signal in each sub-array, and then ranks of all examined concentrations was averaged and plotted as a heatmap (red, highest rank; white, 50th percentile; and blue, lowest rank). (b) Human FGF2 SPR binding analysis with HDF-2, HDF-4, HDF-6 and HD-14. FGF2 concentrations ranged from 0.1 to 4 mM, and a global fit according to a 1 : 1 binding model was applied (black curves); the dissociation constant (*K*_D_) for HDF-2 is 72 ± 0.63 μM; the dissociation constant for HDF-4 is 71 ± 0.61; the dissociation constant for HDF-6 is 89 ± 0.36 and the dissociation constant for HD-14 is 64 ± 0.12; (c) NIH-3T3 WST cell proliferation assay. Cells were seeded on a HS disaccharide coated 96-well plate and treated with FGF2 (10 ng ml^−1^). Control wells (without disaccharide or FGF2) were used as baseline for proliferation (set as 100%) and then quantified after 48 h. Statistics (mean ± SD) was performed in triplicate. (d) MAPK pathway activation assay. NIH-3T3 cells were seeded on a HS disaccharide coated 96-well plate (along with wells with no disaccharides as control), and treated with FGF2 (50 ng ml^−1^) or PBS for control, and total ERK1/2 and pERK1/2 were quantified by respective fluorescently labeled antibodies. All statistical analyses were performed using GraphPad Prism 5. Significant differences between control and HS mimetics are indicated with asterisk (*p < 0.05), and data are expressed as mean ± SD (*n* = 4), using one-way ANOVA.

The resulting fluorescence intensities from the sub-arrays were normalised (expressed as a percentage of the maximum binding signal in each sub-array), averaged, and presented as a heatmap (Fig. 3a), as previously used to study carbohydrate–protein interactions.^11*b*,*c*,13^EGF showed minimal background fluorescence and was therefore excluded from analysis. The remaining six growth factors demonstrated binding preferences dependent on the sulfation pattern, fluorine substitution and the uronic acid composition of the HS disaccharides. Notably, FGF1 exhibited unique and strong binding to the HD-13 disaccharide ligand, but rather weak binding across other HS glycans, regardless of fluorination. This FGF1 binding to HD-13 is consistent with previously reported values.^14^ In contrast, FGF2 displayed a different binding pattern where it strongly bound both fluorinated and non-fluorinated disaccharides. It showed high binding preferences for non-fluorinated *N*-sulfated l-iduronic acid-based HS ligands—namely, HD-13 and HD-14 (ranking 82% and 85%, respectively), and some fluorinated mimetics exhibited similarly high binding preferences (HDF-2, HDF-4 and HDF-6, ranging from 81 to 84%), while higher than their other non-fluorinated counterparts (HD-6 and HD-7: ranking 77% and 70%, respectively). Likewise, HDF-2, which contains an *N*-acetyl domain, exhibited 84% ranking compared to 48% for its bioisosteric analogue HD-2. Furthermore, the increased degree of sulfation in fluorinated ligands led to enhanced binding: HDF-4 bound at 81%, while *N*-sulfated analogues HDF-6 and HDF-7 showed binding of 84% and 72%, respectively. For VEGF_195_, HB-EGF, amphiregulin, and BMP2, the fluorinated HS ligands (HDF2–HDF7) consistently exhibited stronger binding than their non-fluorinated equivalents (HD2–HD7). Nevertheless, among all disaccharides tested, highly sulfated l-iduronic acid-based HD-13 and HD-14 showed the strongest binding across most proteins, illustrating the preference of growth factors to l-iduronic acid over d-glucuronic acid, as previously suggested.^14,15^ Collectively, these results prompted further investigation into the relationship between human FGF2 and fluorinated HS disaccharides, particularly in *N*-acetate domain d-glucuronic acid-based HS structures (HDF-2 and HDF-4). To corroborate the binding specificity, we performed surface plasmon resonance (SPR) measurements between FGF2 and HDF-2, HDF-4, and HDF-6, in comparison to HD-14. The SPR analysis demonstrated that all four disaccharides displayed similar affinities in the micromolar range (Fig. 3b and Table S7, ESI†), highlighting that the fluoro-HS mimetics, although not containing l-iduronic acid, can be used as higher affinity compounds. Although molecular modelling studies for the interaction between FGF2*vs.*HDF-2 and HDF-4 did not reveal further stabilizing contacts for the fluorinated analogues compared to the natural disaccharides, the incorporation of fluorine did not disrupt any long-lasting interaction (Fig. S1 and S2, ESI†). Notably, besides the hydrophobic contribution, nonbonding protein–fluorine interactions such as orthogonal CF–C=O(N) contacts, fluorine–nonpolar hydrogen contacts, and hydrogen bonds could be operative and potentially increased the binding affinity of the fluorinated mimetics. Finally, to further investigate the interaction between human FGF2 and the fluorinated mimetics in biologically relevant context, cell proliferation and MAPK pathway activation assays were conducted with NIH-3T3 cells treated with human FGF2 with and without exposure to the disaccharide mimetics. All four HS disaccharides (HDF-2, HDF-4, HDF-6 and HD-14) displayed similar cell proliferation and MAPK activity, suggesting that the incorporation of a non-natural fluorine atom into *N*-acetylated HS mimetics did not interfere with the biological activity of the cells (Fig. 3c–e).

**Fig. 4:**
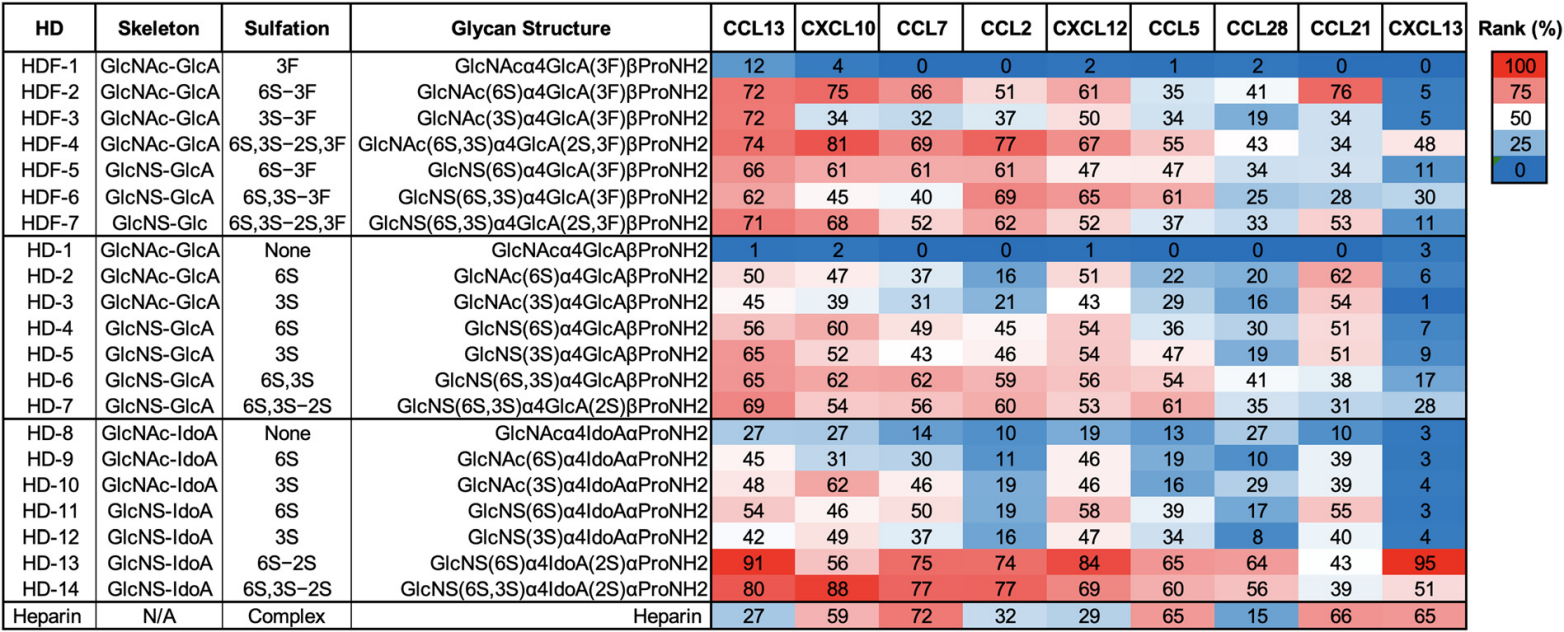
Chemokines binding to HD and HDF mimetics: (a) HS disaccharide mimetics microarray analysis of human CCL13, CXCL10, CCL7, CCL2, CXCL12, CCL5, CCL28, CCL21, and CXCL13. All proteins were tested at three concentrations (10, 3.3 and 1.6 ng μl^−1^) each per sub-array. Binding was tested with biotinylated secondary antibodies and then detected with Cy3-streptavidin. Relative fluorescence units (RFU) were ranked according to the highest signal in each sub-array, and then ranks of all of the examined concentrations were averaged and plotted as a heatmap (red, highest rank; white, 50th percentile; blue, lowest rank).

Next, nine human chemokines were examined by the glycan HS mimetics microarray (Fig. 4): three homeostatic chemokines (CCL28, CXCL12, and CCL21) and six inflammatory chemokines [CXCL13, CXCL10 (IP-10), CCL2 (MCP-1), CCL7 (MCP-3), CCL13 (MCP-4), and CCL5 (RANTES)]. Glycan microarray analysis showed that overall, all chemokines, but CXCL13, bound well to the fluorinated mimetics. Human CCL13 showed the widest recognition profile, with high binding to all fluorinated and sulfated HS mimetics and top binding to highly sulfated l-iduronic acid containing HS mimetics (HD-13 and HD-14). Similarly, all the other inflammatory chemokines (CXCL10, CCL7, CCL2, and CCL5) showed the highest binding to HD-13 and HD-14 and also recognized the 6-*O*-sulfated fluorinated mimetics (HDF-2 to HDF-7) well, slightly better than the non-fluorinated disaccharides (HD-2 to HD-7). Each of the hemostatic chemokines showed different binding patterns. Human CCL28 preferred HD13 and HD-14 with only lower ranked binding to the 6-*O*-sulfated fluorinated mimetics HFD-2 and HFD-4 and to the non-fluorinated glycan HD-6. Interestingly, human CCL21 top ranked glycan was HFD-2, whereas CXCL13 preferred HD-13 with lower preference to HD-14 and HFD-4. Further research is needed to determine whether these preferences can be exploited to design HS mimetics for glycotherapy.

## Conclusions

We herein reported a divergent strategy to synthesize a first generation of fluorinated HS disaccharide ligands having both *N*-acetate and *N*-sulfated glucosamine moieties. ^19^F-NMR studies have shown that the chemical shifts are sensitive to the sulfation pattern. Highly sulfated ligands showed upward shifts compared to low or non-sulfated HS ligands. Conformation plasticity analysis of fluorinated HS showed that all compounds keep the canonical ^4^C_1_-glucuronic acid conformation. Finally, a systematic glycan microarray and SPR analysis showed that fluorine edition to HS mimetics significantly modulates the binding preferences compared with the low-sulfated HS ligands to growth factors and chemokines. Molecular modelling studies of the interaction between FGF2 and HDF analogs suggest that the newly synthesized molecules bind to the protein with a binding mode similar to the natural disaccharides. These results suggest that fluorinated heparan sulfate (HS) ligands may exhibit distinct and modular binding interactions with growth factors and chemokines compared to their native counterparts. While the current study provides important initial insights, the use of higher oligosaccharides may offer a more comprehensive understanding of these interactions. Ongoing work in our laboratory is focused on extending this approach to higher-order HS structures.

## Author contributions

R. K., J. J. B. and V. P. K. planned the project, analysed data, and wrote manuscript with some assistance from other co-authors. V. M., R. R., S. A., S. M., and A. C. synthesized, purified, and characterized the HS ligands and performed all assays. P. G. and A. G. performed conformation studies. S. L. B.-A. performed HS-protein binding assay on microarrays and critically reviewed the manuscript.

## Conflicts of interest

The authors declare no conflict of interest.

## Supplementary Material

CB-OLF-D5CB00174A-s001

CB-OLF-D5CB00174A-s002

CB-OLF-D5CB00174A-s003

## Data Availability

The data supporting this article have been included as part of the ESI.†
