# Supplementary material for: Unravelling structure–function interactions between fluorinated heparan sulfate mimetics and signaling proteins
Source: RSC Chem Biol. 2025 Jul 10;6(9):1465–72. doi: 10.1039/d5cb00174a (PMC12311619; doi:10.1039/d5cb00174a)
Supplement: CB-006-D5CB00174A-s003 [file CB-006-D5CB00174A-s003.pdf]

Supplementary Information (SI) for RSC Chemical Biology.
This journal is © The Royal Society of Chemistry 2025

Unravelling Structure-Function Interactions Between Fluorinated Heparan Sulfate Mimetics and Signaling Proteins

Virendrasinh Mahida,[a,d] Rakesh Raigawali,[a,d] Paula González,[b] Ana Gimeno,[b,c] Shani Ben-Arye,[c] Saurabh Anand,[a] Sandhya Mardhekar,[a] Jesús Jiménez-Barbero,[b,c,d]* Vered Padler-Karavani,[e,*] Raghavendra Kikkeri,[a,*]

[a]Indian Institute of Science Education and Research, Dr. Homi Bhabha Road, Pune-411008, India

[b]CIC bioGUNE, Basque Research Technology Alliance, BRTA, Bizkaia Technology park, 48160 Derio, Spain

[c]Ikerbasque, Basque Foundation for Science, 48009 Bilbao, Spain

[d]Dept. Organic Chemistry II, Faculty of Science and Technology, UPV-EHU, 48940 Leioa, Spain

[e]Department of Cell Research and Immunology, The Shmunis School of Biomedicine and Cancer Research, The George S. Wise Faculty of Life Sciences, Tel Aviv University, Tel Aviv, 69978, Israel.

Corresponding Email: jjbarbero@cicbiogune.es
vkaravani@tauex.tau.ac.il
rkikkeri@iiserpune.ac.in

Table of contents.

1. General instructions.
2. Synthesis of fluorine-glucose building block (**12**)
3. Synthesis of fluorine disaccharide analogs (**HDF-1 to HDF-7**).
4. NMR Experiments. General Remarks
5. Conformational Analysis
6. Molecular modelling of the interaction between HDF-2, HDF-4 and HD-7 with FGF2.
7. Surface Plasmon Resonance binding kinetics

8. 96-well plate HS mimetic immobilization

9. Cell proliferation

10. MAPK pathway activation assay

11. Glycan microarray assay

12. Spectral data.

EXPERIMENTAL SECTION

1. General Instructions. All chemicals were reagent grade and used as supplied except where noted. Analytical thin layer chromatography (TLC) was performed on Merck silica gel 60 F254 plates (0.25 mmol). Compounds were visualized by UV irradiation or dipping the plate in CAM/ninhydrin solution followed by heating. Column chromatography was carried out using force flow of the indicated solvent on Flukab Kieselgel 60 (230–400 mesh). ^{1}H and ^{13}C NMR spectra were recorded on Jeol 400 MHz, with cryo probe using residual solvents signals as an internal reference ($CDCl_3$ δ_H, 7.26 ppm, δ_C 77.3 ppm and CD_3OD δ_H 3.31 ppm, δ_C 49.0 ppm). The chemical shifts (δ) are reported in ppm and coupling constants (*J*) in Hz. HS disaccharides (**HD-1** to **HD-14**) were synthesized by using previously published procedure .[1]

2. Synthesis of fluorine-glucose building block (**12**)

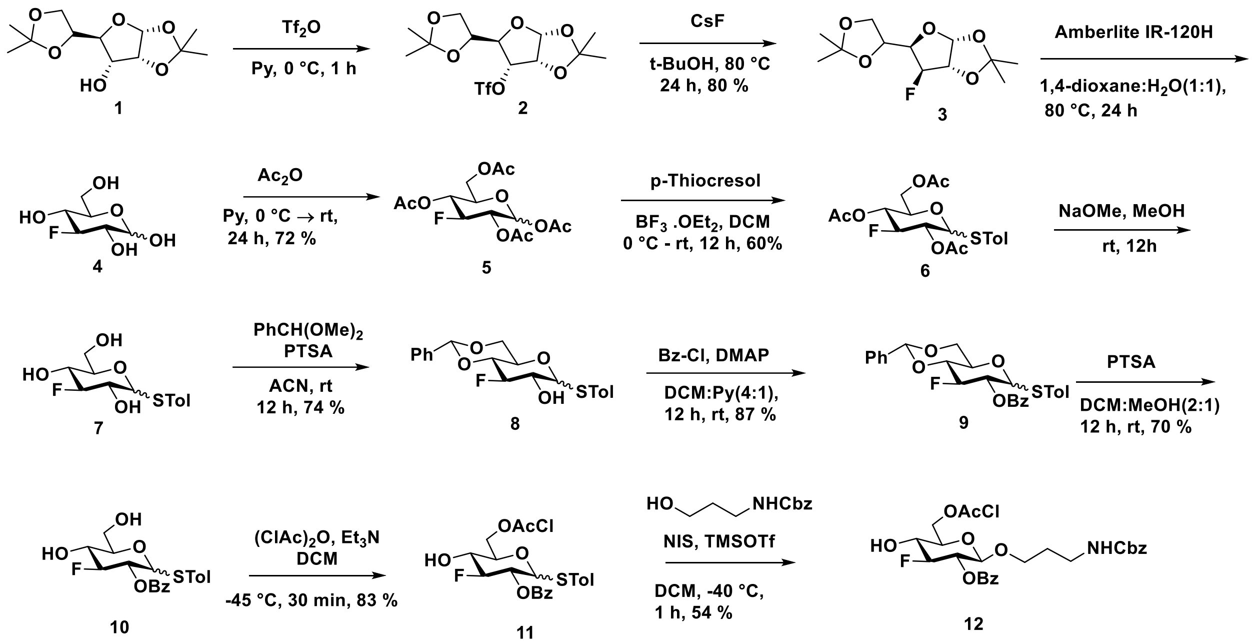

Scheme 1. Synthesis of glucose building block.

3-deoxy-3-fluoro-1,2-5,6-diisopropylidene-α-D-glucopyranose (3):

To a solution of diacetone allofuranose **1** (15.0 g, 57.69 mmol) in pyridine (150 mL), triflic anhydride (11.6 mL, 69.228 mmol) was added over 10 minutes. After stirring in an ice bath for 1 h, the reaction mixture was diluted with ethyl acetate, and the organic layer was washed with half-saturated sodium bicarbonate and brine, then dried over Na_2SO_4 and concentrated under reduced pressure to afford the crude triflate Compound **2**, which was used in the next step without purification. A solution of Compound **2** in tert-butanol (225 mL) was added to cesium fluoride (26 g, 173.07 mmol). The reaction was heated at 80 °C for 22 h, then cooled to room

temperature and diluted with ethyl acetate. The organic layer was washed with saturated sodium bicarbonate and brine, dried over Na_2SO_4, concentrated, and purified by flash silica gel column chromatography to yield Compound **3** (12.6 g, 80%, 2 steps). ^1H NMR (400 MHz, Chloroform-*d*) δ 5.95 (d, *J* = 3.7 Hz, 1H), 5.01 (dd, *J* = 49.9, 2.2 Hz, 1H), 4.70 (dd, *J* = 10.7, 3.7 Hz, 1H), 4.29 (ddd, *J* = 8.3, 6.1, 4.8 Hz, 1H), 4.18 – 4.05 (m, 2H), 4.03 (dd, *J* = 8.7, 4.8 Hz, 1H), 1.50 (s, 3H), 1.45 (s, 3H), 1.37 (s, 3H), 1.33 (s, 3H). ^{19}F NMR (376 MHz, Chloroform-*d*) δ -207.60. ^{13}C NMR (101 MHz, Chloroform-*d*) δ 112.31, 109.45, 105.15, 94.69, 92.87, 82.67, 82.34, 80.72, 80.53, 77.39, 77.08, 76.76, 71.89, 71.82, 67.12, 26.80, 26.65, 26.14, 25.12. HR-ESI-MS *(m/z)*: [M+Na]+ calcd for $C_{12}H_{19}FO_5$, 262.1217; found, 262.1214.

3-deoxy-3-fluoro-1,2,4,6-tetraacetyl-D-glucopyranose (5)

A solution of Compound **3** (13 g) in 1,4-dioxane (150 mL) and water (150 mL) was added to acidic resin (Amberlite IR-120H, 45 g). The reaction mixture was stirred at 80 °C for 24 hours, then cooled to room temperature and filtered through a sintered glass funnel. The resin was washed with additional 80% aqueous acetonitrile, and the combined filtrates were concentrated under reduced pressure. The resulting viscous residue (Comp **4**) was dried under high vacuum for 2 hours and used in the subsequent step without purification. A solution of Compound **4** in pyridine (150 mL) was cooled to 0 °C, and acetic anhydride (50 mL) was added dropwise. The reaction mixture was stirred at room temperature for 24 hours, then concentrated under reduced pressure. The residual oil was redissolved in ethyl acetate, and the organic layer was washed sequentially with half-saturated sodium bicarbonate solution and brine, then dried over Na_2SO_4, concentrated, and purified by flash silica gel column chromatography to afford Compound **5** (13.2 g, 72% , 2 steps). ^1H NMR (400 MHz, Chloroform-*d*) δ 6.33 (t, *J* = 3.7 Hz, 1H), 5.68 (d, *J*

= 8.3 Hz, 1H), 5.40 – 5.05 (m, 4H), 4.75 (ddt, *J* = 71.8, 52.0, 9.3 Hz, 2H), 4.27 (ddd, *J* = 12.4, 5.4, 4.4 Hz, 2H), 4.17 – 4.02 (m, 3H), 3.80 (dddd, *J* = 10.1, 4.6, 2.3, 1.1 Hz, 1H), 2.17 (s, 3H), 2.13 (s, 3H), 2.11 (s, 6H), 2.10 (s, 3H), 2.09 – 2.07 (m, 9H). ^{19}F NMR (376 MHz, Chloroform-*d*) δ -196.06, -199.96 ^{13}C NMR (101 MHz, Chloroform-*d*) δ 170.39, 170.37, 169.48, 169.08, 169.06, 168.89, 168.82, 168.80, 168.48, 92.39, 91.07, 90.96, 90.50, 89.91, 89.14, 89.05, 88.03, 77.56, 77.43, 77.24, 76.92, 71.69, 71.62, 70.27, 70.08, 69.59, 69.55, 69.48, 69.41, 67.73, 67.71, 67.55, 67.52, 61.24, 20.55, 20.52, 20.47, 20.43, 20.41, 20.38, 20.36, 20.27, 20.24. HR-ESI-MS *(m/z)*: [M+Na]+ calcd for $C_{14}H_{19}FO_9$, 373.0911; found, 373.0910.

4-Methylphenyl-[2,4,6-*O*-triacetyl-3-deoxy-3-fluoro-1-thio- α/β-D glucopyranoside] (6)

To a solution of Compound **5** (17 g, 48.57 mmol) in anhydrous CH_2Cl_2 (250 mL), *p*-toluene thiol (30.11 mg, 242.85 mmol) and $BF_3{\cdot}OEt_2$ (53.59 mL, 437.13 mmol) were added portion-wise. The reaction mixture was stirred at room temperature for 4 days under an argon atmosphere. After completion, the reaction mixture was diluted with CH_2Cl_2 (100 mL) and washed twice with saturated aqueous sodium bicarbonate (2 × 100 mL). The organic layer was dried over Na_2SO_4, and the solvent was removed under reduced pressure. The resulting residue was adsorbed onto silica and purified by silica gel column chromatography to afford Compound **6** (11.9 g, 60%). ^{1}H NMR (400 MHz, Chloroform-*d*) δ 7.31 (d, *J* = 8.2 Hz, 2H), 7.17 – 7.05 (m, 2H), 5.85 (dd, *J* = 5.9, 2.5 Hz, 1H), 5.33 – 5.09 (m, 2H), 4.80 (dt, *J* = 52.8, 9.4 Hz, 1H), 4.50 (ddd, *J* = 10.3, 5.3, 2.2 Hz, 1H), 4.25 (dd, *J* = 12.4, 5.3 Hz, 1H), 4.05 (ddd, *J* = 12.3, 2.3, 1.4 Hz, 1H), 2.32 (s, 3H), 2.17 (s, 3H), 2.14 (s, 3H), 2.04 (s, 3H). ^{19}F NMR (376 MHz, Chloroform-*d*) δ -195.60. ^{13}C NMR (101 MHz, Chloroform-*d*) δ 170.60, 169.85, 169.38, 138.55, 138.38, 138.15, 133.53, 132.62, 131.98, 130.02, 129.86, 129.80, 129.67, 128.55, 128.29, 90.50, 88.61, 85.28, 85.20, 77.37, 77.25,

77.05, 76.73, 71.31, 71.13, 68.56, 68.38, 67.88, 67.81, 61.87, 61.85, 29.71, 21.13, 20.91, 20.81, 20.72, 20.68. HR-ESI-MS *(m/z)*: [M+Na]+ calcd for $C_{19}H_{23}FO_7S$, 437.1046; found, 437.1033.

4-Methylphenyl-[4,6-*O*-benzilidine-3-deoxy-3-fluoro-1-thio-α/β-D glucopyranoside] (8)

To a solution of Compound **6** (10.5 g, 28.15 mmol) in methanol (150 mL), sodium methoxide (760 mg, 14.07 mmol) was added, and the reaction mixture was stirred at room temperature for 3 h. After completion, the mixture was neutralized using *Amberlyst*® H^+ resin, filtered, and concentrated under reduced pressure. The resulting residue was adsorbed onto silica and purified by silica gel column chromatography (ethyl acetate: methanol, 10:1) to afford Compound **7** as a colourless solid, which was used in the next step without further purification. Subsequently, Compound **7** was dissolved in dry acetonitrile (120 mL), and *p*-toluenesulfonic acid (PTSA, 484 mg, 2.81 mmol) and benzaldehyde dimethyl acetal (41.99 mL, 281.5 mmol) were added. After completion of the reaction, it was quenched by the addition of triethylamine. The solvent was removed under reduced pressure, and the residue was adsorbed onto silica and purified by silica gel column chromatography to afford Compound **8** (7.09 g, 74%, 2 steps). ^{1}H NMR (400 MHz, Chloroform-*d*) δ 7.51 – 7.40 (m, 4H), 7.36 (dd, *J* = 5.1, 2.1 Hz, 3H), 7.16 (d, *J* = 8.0 Hz, 2H), 5.53 (s, 1H), 4.76 – 4.48 (m, 2H), 4.40 (ddd, *J* = 10.5, 5.0, 2.0 Hz, 1H), 3.83 – 3.68 (m, 2H), 3.68 – 3.55 (m, 1H), 3.48 (tdd, *J* = 9.8, 5.0, 1.4 Hz, 1H), 2.71 (d, *J* = 2.5 Hz, 1H), 2.36 (s, 3H). ^{19}F NMR (376 MHz, Chloroform-*d*) δ -192.75. ^{13}C NMR (101 MHz, Chloroform-*d*) δ 139.26, 136.63, 134.01, 130.02, 129.31, 128.55, 128.35, 126.62, 126.21, 101.64, 94.22, 92.33, 88.42, 88.34, 78.76, 78.59, 77.37, 77.25, 77.05, 76.73, 71.28, 71.10, 69.81, 69.73, 68.45, 68.44, 62.72, 32.22, 22.87, 21.23. HR-ESI-MS *(m/z)*: [M+Na]+ calcd for $C_{20}H_{21}FO_4S$, 399.1042; found, 399.1046.

4-Methylphenyl-[2-*O*-benzoyl-4,6-*O*-benzilidine-3-deoxy-3-fluoro-1-thio-α/β-D glucopyranoside] (9)

A solution of Compound **8** (8.1 g, 20.30 mmol) in CH_2Cl_2 (80 mL) and pyridine (20 mL) was cooled to 0 °C, and DMAP (495 mg, 4.06 mmol) was added, followed by the dropwise addition of benzoyl chloride (7.07 mL, 60.9 mmol). The reaction mixture was stirred for 12 hours at room temperature. After completion, the reaction mixture was diluted with CH_2Cl_2 (20 mL), washed sequentially with 1 N HCl and brine, then dried over Na_2SO_4. The solvent was removed under reduced pressure, and the residue was precipitated using methanol. The precipitate was collected by filtration using a Büchner funnel to afford Compound **9** (8.88 g, 87%). ^{1}H NMR (400 MHz, Chloroform-*d*) δ 8.10 (dd, *J* = 8.1, 1.4 Hz, 2H), 7.65 – 7.56 (m, 1H), 7.53 – 7.41 (m, 4H), 7.40 – 7.32 (m, 5H), 7.11 (d, *J* = 7.9 Hz, 2H), 5.57 (s, 1H), 5.45 – 5.21 (m, 1H), 5.02 – 4.62 (m, 2H), 4.43 (ddd, *J* = 10.5, 5.0, 2.0 Hz, 1H), 4.02 – 3.74 (m, 2H), 3.55 (tdd, *J* = 9.9, 5.0, 1.4 Hz, 1H), 2.33 (s, 3H). ^{19}F NMR (376 MHz, Chloroform-*d*) δ -192.86. ^{13}C NMR (101 MHz, Chloroform-*d*) δ 164.95, 138.97, 136.62, 133.94, 133.52, 133.17, 130.01, 129.83, 129.44, 129.34, 128.53, 128.37, 127.62, 126.23, 101.62, 92.61, 90.69, 86.68, 86.61, 78.78, 78.61, 77.39, 77.28, 77.08, 76.76, 71.22, 71.03, 69.79, 69.71, 68.43, 68.41, 21.23. HR-ESI-MS *(m/z)*: [M+Na]+ calcd for $C_{27}H_{25}FO_5S$, 503.1304; found, 503.1304.

4-Methylphenyl-[2-*O*-benzoyl-3-deoxy-3-fluoro-1-thio-α/β-D glucopyranoside] (10)

To a solution of compound **9** (8.6 g, 17.09 mmol) in CH_2Cl_2 (40 ml) and MeOH (80 ml) was added PTSA (5.88 g, 34.18 mmol) and stirred for 12 h. Upon completion, the reaction mixture was quenched using Et_3N. The solvent was removed under reduced pressure, and the residue was purified through flash silica gel column chromatography to obtain Compound **10** (4.96 gm, 70

%). ^{1}H NMR (400 MHz, Chloroform-*d*) δ 8.16 – 7.93 (m, 2H), 7.60 (td, *J* = 7.4, 1.3 Hz, 1H), 7.46 (td, *J* = 7.6, 1.5 Hz, 2H), 7.37 – 7.27 (m, 2H), 7.08 (dd, *J* = 8.2, 1.9 Hz, 2H), 5.25 (ddd, *J* = 11.9, 10.0, 8.8 Hz, 1H), 4.77 (dd, *J* = 10.1, 1.0 Hz, 1H), 4.63 (dtd, *J* = 52.8, 8.8, 1.4 Hz, 1H), 4.02 – 3.79 (m, 3H), 3.46 (dt, *J* = 7.0, 2.3 Hz, 1H), 2.31 (s, 3H).^{19}F NMR (376 MHz, Chloroform-*d*) δ -192.20. ^{13}C NMR (101 MHz, Chloroform-*d*) δ 165.22, 165.19, 138.77, 138.75, 133.48, 133.46, 133.42, 130.17, 130.00, 129.85, 129.45, 128.50, 128.02, 127.98, 96.61, 94.73, 85.80, 85.72, 78.70, 78.66, 78.59, 77.38, 77.06, 76.75, 70.68, 70.64, 70.50, 70.46, 68.89, 68.83, 68.71, 68.65, 61.86, 21.17. HR-ESI-MS *(m/z)*: [M+Na]+ calcd for $C_{20}H_{21}FO_5S$, 415.0991; found, 415.0987.

4-Methylphenyl-[2-*O*-benzoyl-6-chloroacetyl-3-deoxy-3-fluoro-1-thio-α/β-D glucopyranoside] (11)

A solution of Compound **10** (4.7 g, 11.32 mmol) in CH_2Cl_2 (160 mL) was stirred at -45 °C for 15 minutes under a nitrogen atmosphere before the addition of chloroacetic anhydride (2.3 g, 13.58 mmol) and triethylamine (Et_3N, 1.57 mL, 11.32 mmol). The reaction progress was monitored by TLC and was found to be complete within approximately 15–20 minutes. Upon completion, the reaction mixture was washed sequentially with 1 N HCl and brine. The organic layer was collected, dried over Na_2SO_4, filtered, and concentrated under reduced pressure. The residue was purified by silica gel column chromatography to afford Compound **11** (4.61 g, 83%). ^{1}H NMR (400 MHz, Chloroform-*d*) δ 8.09 (dd, *J* = 8.3, 1.4 Hz, 2H), 7.68 – 7.58 (m, 1H), 7.49 (dd, *J* = 8.3, 7.1 Hz, 2H), 7.38 – 7.32 (m, 2H), 7.16 – 7.02 (m, 2H), 5.34 – 5.13 (m, 1H), 4.83 – 4.62 (m, 2H), 4.61 – 4.54 (m, 1H), 4.53 – 4.46 (m, 1H), 4.11 (s, 2H), 3.88 – 3.76 (m, 1H), 3.63 (dddd, *J* = 10.0, 5.2, 2.4, 1.1 Hz, 1H), 2.76 (d, *J* = 3.5 Hz, 1H), 2.34 (s, 3H). ^{19}F NMR (376 MHz, Chloroform-*d*) δ -192.54. ^{13}C NMR (400 MHz, Chloroform-*d*) δ 167.42, 165.05, 138.90, 133.95,

133.52, 129.98, 129.70, 129.39, 128.52, 127.57, 96.53, 94.65, 85.59, 85.51, 77.36, 77.04, 76.72, 76.40, 76.32, 70.22, 70.04, 68.91, 68.73, 64.29, 64.27, 40.70, 21.20. HR-ESI-MS *(m/z)*: [M+Na]+ calcd for $C_{27}H_{25}FO_5S$, 491.0707; found, 491.0707.

_N_-benzyloxycarbonyl-3-aminopropyl-_O_- [2-_O_-benzoyl-6-chloroacetyl-3-deoxy-3-fluoro-1-thio-β-D glucopyranoside] (12)

A solution of donor **11** (1.0 g, 2.03 mmol) and linker benzyl (3-hydroxypropyl) carbamate (0.766 g, 3.65 mmol) in CH_2Cl_2 (30 mL) was stirred under a nitrogen atmosphere in a round-bottom flask containing freshly dried 4 Å molecular sieves for 2 hours. Next, *N*-iodosuccinimide (NIS, 0.549 g, 2.43 mmol) and trimethylsilyl trifluoromethanesulfonate (TMSOTf, 74 μL, 0.406 mmol) were added at -40 °C. The temperature was gradually increased to -20 °C, and the reaction mixture was stirred until completion, as monitored by TLC. The reaction was quenched by the addition of a few drops of triethylamine (Et_3N). The molecular sieves were removed by filtration through Celite, and the organic layer was washed sequentially with aqueous sodium thiosulphate ($Na_2S_2O_3$) and brine. The organic phase was collected, dried over Na_2SO_4, filtered, and concentrated under reduced pressure. The residue was purified by silica gel column chromatography to afford Compound **12** (608 mg, 54%). ^{1}H NMR (400 MHz, Chloroform-*d*) δ 8.17 – 7.90 (m, 2H), 7.64 – 7.53 (m, 1H), 7.43 (t, *J* = 7.7 Hz, 2H), 7.38 – 7.28 (m, 5H), 5.29 (ddd, *J* = 13.0, 9.3, 8.0 Hz, 1H), 5.04 (s, 2H), 4.72 – 4.55 (m, 1H), 4.57 – 4.44 (m, 2H), 4.09 (s, 2H), 3.93 – 3.78 (m, 2H), 3.58 (m, 2H), 3.24 – 3.05 (m, 3H), 1.72 (m, 2H). ^{19}F NMR (376 MHz, Chloroform-*d*) δ -195.49. ^{13}C NMR (101 MHz, Chloroform-*d*) δ 167.70, 165.32, 156.59, 136.76, 133.62, 129.93, 129.33, 128.63, 128.16, 100.58, 100.47, 95.38, 93.53, 77.45, 77.33, 77.13, 76.81,

72.49, 72.40, 71.85, 71.67, 69.05, 68.86, 67.76, 66.61, 64.17, 40.78, 38.07, 29.52. HR-ESI-MS *(m/z)*: [M+Na]+ calcd for $C_{26}H_{29}ClFNO_9$, 553.1515; found, 553.1515.

3. Synthesis of HDF-1 to HDF-7

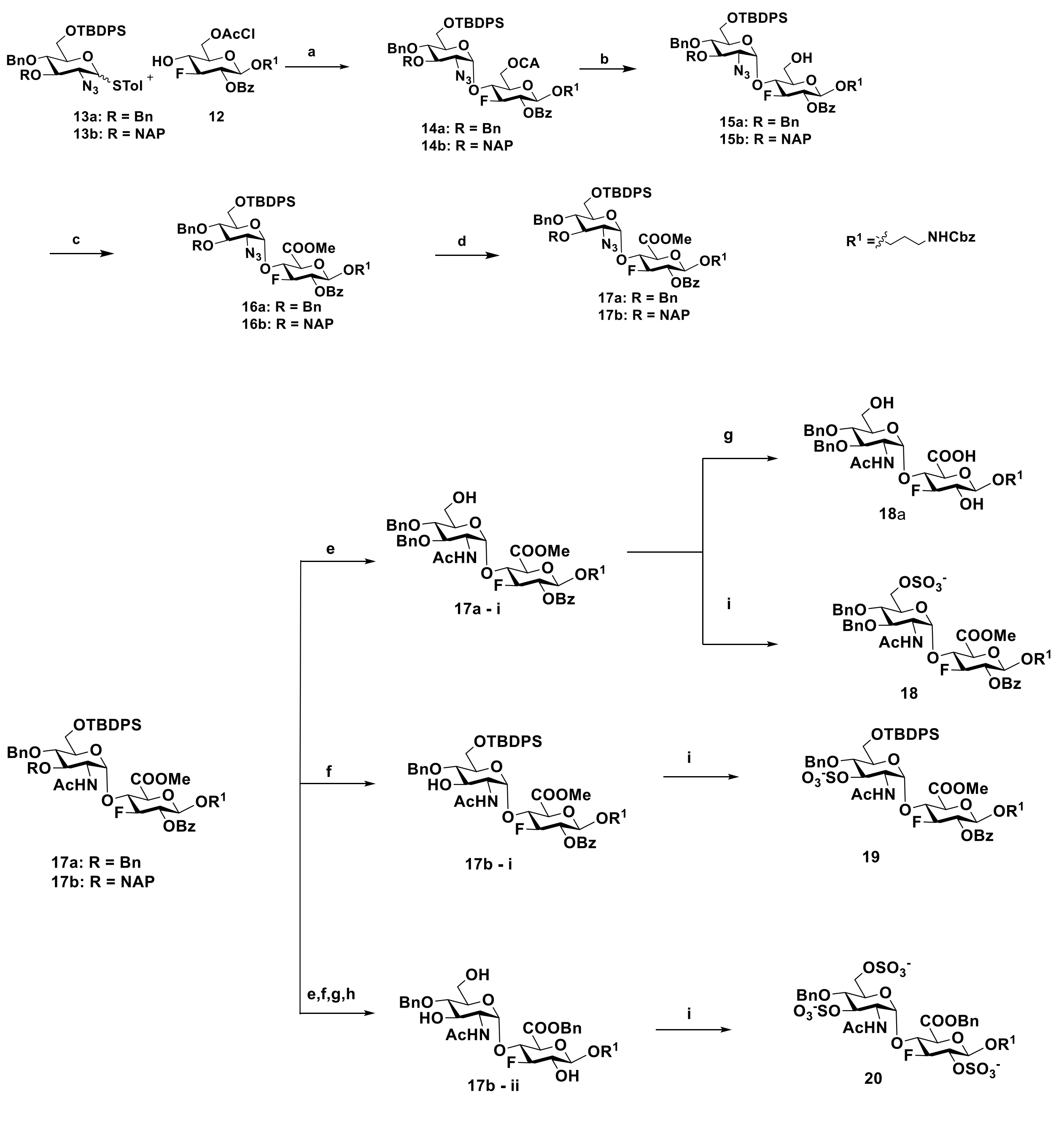

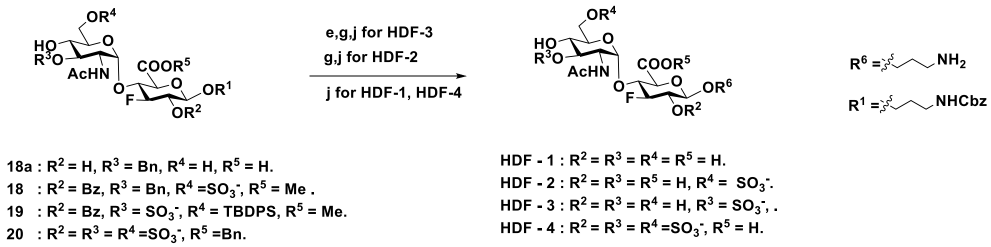

Scheme 2. Synthesis of Glucuronic acid based HS disaccharides: (a) NIS, TMSOTf, DCM, -78 °C, 15 min; (b) Thiourea, Py : MeOH (1 : 1), 80 °C, 2 h; (c) (i) TEMPO, BAIB, DCM:H_2O(1:1), RT ; (ii) MeI, K_2CO_3, DMF, 6 h, RT; (d) Zn dust, THF:AcOH:Ac_2O (3:2:1), RT, 12 h; (e) HF.Py, Py, 0 °C, 12 h; (f) DDQ, DCM:H_2O (18:1), RT, 1h; (g) LiOH, H_2O:THF(1 : 1), RT, 12 h; (h) BnBr, $NaHCO_3$, DMF, 60 °C, 2h; (i) SO_3. Et_3N, DMF, 60 °C, 48-72 h; (j) H_2, $Pd(OH)_2$, H_2O, RT, 48 h.

General procedure for TBDPS deprotection

To a solution of TBDPS protected HS oligosaccharides (1 mmol) in pyridine (5 ml) was added 70% HF.Py (5 mmol) at 0 °C and stirred at RT overnight. After completion, the reaction mixture was diluted with EtOAc and washed with 1 N HCl and brine. The organic layer was dried using anhydrous Na_2SO_4 and concentrated under reduced pressure. The residue was purified by silica column chromatography. or Sephadex LH-20 column depending on the sulfate group in the starting material.

General procedure for NAP deprotection

To a solution of NAP-protected HS oligosaccharides in DCM/H2O (18:1, 10 mL for 0.620 mg) was added DDQ (3 equiv) and stirred under nitrogen atmosphere at 0 °C for 3-4 h. After completion of the reaction, the mixture was diluted with DCM and washed with brine. The

organic layer was concentrated under reduced pressure and the residue was purified by silica gel column chromatography (EtOAc/Hexane).

General procedure for *O*-Sulfation

To a solution of 6-*O* / 3-*O* / 2-*O*-hydroxyl group HS oligosaccharides (1 mmol) in DMF (5 mL) was added $SO_3 \cdot NMe_3$/$SO_3 \cdot NEt_3$ (10 mmol per -OH) and stirred at 60 °C for 72 h. DMF was evaporated and the crude product was purified by column chromatography (MeOH/DCM).

General procedure for Saponification

To a solution of HS oligosaccharides (1 mmol) in a mixture of THF/H_2O (1:1, 4 ml) was added 1 M $LiOH \cdot H_2O$ (3 mmol) and stirred at RT for 15 h. After completion of the reaction, the reaction mixture was diluted with MeOH and neutralized using Dowex 50WX8 H+ resin. The reaction was filtered and concentrated under reduced pressure and purified by bond elute C-18 column (H_2O/ACN).

General procedure for Global deprotection

To a solution of HS oligosaccharides (1 mmol) in H_2O (4 ml) was added $Pd(OH)_2$ (10% by wt.) and stirred for 48 h under hydrogen atmosphere. After completion of the reaction, the reaction mixture was diluted with H_2O, filtered and concentrated, and finally purified by bond elute C-18 column (H_2O).

General procedure for Azide reduction

Trimethyl phosphine (PMe_3, 1.0 M sol in THF, 8 equiv. per azide group) and aq. NaOH (1 M, 2.0 equiv.) solution was added to the solution of starting material in THF (1.0 mL for 0.013 mmol) and the reaction mixture was stirred for 12 h. The progress of reaction was monitored by TLC (EtOAc/MeOH/H2O, 7/2/1, v/v/v). Upon completion, the pH was adjusted to 8.0–8.5 by careful addition of glacial AcOH, and the mixture was concentrated in vacuo. The residue obtained was purified by reverse phase chromatography (C-18 silica gel column). The appropriate fractions were concentrated in vacuo, and the residue was passed through a column DowexR 50 x 8 Na+ resin using MeOH/H_2O as eluent. The appropriate fractions were combined and lyophilized to obtain desired product as sodium salt.

General procedure for *N*-sulfation

6 material in a mixture of MeOH (1.0 mL for 0.006 mmol), Et_3N (0.3 mL) and aq. NaOH solution (1 M, 2.0 equiv.) at 0 ºC was added SO_3.Py complex (5.0 equiv. per amine). The progress of reaction was monitored by TLC (EtOAc/pyridine/H_2O/AcOH 8/5/3/1, v/v/v/v). Two additional portions of SO_3.Py complex were added at 0 ºC after 1h and 2h. After stirring for an additional 8h, the reaction mixture was

concentrated in vacuo and the residue was passed through a column of DowexR 50 x 8 Na+ resin using H_2O/MeOH as eluent to obtain desired product. The appropriate fractions were concentrated in vacuo and further was purified by reverse phase C-18 silica gel column using H_2O/MeOH as eluent. Appropriate fractions were lyophilized to obtain desired product as sodium salt.

***N*-benzyloxycarbonyl-3-aminopropyl-*O*-(2-azido-2-deoxy-3-*O*-naphthylmethyl-4-*O*- benzyl-6-*O*-*tert*-butyldiphenylsilyl-α-D-glucopyranosyl)-(1→4)-*O*-2-*O*-benzoyl-3-deoxy-3-fluoro-β-D glucopyranoside (15b)**

To a solution of Compound **13b** (donor, 2.0 g, 2.56 mmol) and Compound **12** (acceptor, 1.13 g, 2.04 mmol) in CH_2Cl_2 (60 mL), stirring was carried out at room temperature in a round-bottom flask containing freshly dried 4 Å molecular sieves for 1 hour. The reaction mixture was then stirred under a nitrogen atmosphere and cooled to -78 °C, followed by the addition of *N*-iodosuccinimide (NIS, 0.692 g, 3.072 mmol) and trimethylsilyl trifluoromethanesulfonate (TMSOTf, 92 µL, 0.512 mmol). The temperature was gradually increased to -20 °C, and the reaction mixture was stirred for an additional 15 minutes. Completion of the reaction was monitored by TLC, after which it was quenched with a few drops of triethylamine (Et_3N). The molecular sieves were removed by filtration through Celite, and the organic layer was washed sequentially with aqueous sodium thiosulphate ($Na_2S_2O_3$) and brine. The organic phase was collected, dried over anhydrous Na_2SO_4, filtered, and concentrated under reduced pressure. The residue was purified by silica gel column chromatography (ethyl acetate/hexane) to afford Compound **14b** in 63% yield, which was taken for next step. To a solution of Compound **14b** (1.1 g, 0.910 mmol) in a pyridine/methanol mixture (1:1, 15 mL) at 80 °C, thiourea (0.138 g, 1.82 mmol) was added, and the reaction mixture was stirred under a nitrogen atmosphere for 6 hours. Upon completion, the solvent was evaporated under reduced pressure, and the residue was dissolved in ethyl acetate and washed sequentially with 1 N HCl and brine. The organic layer was dried over anhydrous Na_2SO_4, filtered, and concentrated under reduced pressure. The crude product was purified by silica gel column chromatography (ethyl acetate/hexane) to afford Compound **15b** in 82% yield. ^{1}H NMR (400 MHz, Chloroform-*d*) δ 8.10 – 8.00 (m, 2H), 7.87 –

7.76 (m, 4H), 7.69 (ddt, *J* = 13.1, 6.7, 1.5 Hz, 4H), 7.58 (t, *J* = 7.4 Hz, 1H), 7.52 (dd, *J* = 8.4, 1.7 Hz, 1H), 7.49 – 7.26 (m, 18H), 7.19 (dd, *J* = 6.6, 3.1 Hz, 2H), 5.43 (d, *J* = 3.8 Hz, 1H), 5.39 – 5.26 (m, 1H), 5.12 – 5.00 (m, 5H), 5.01 – 4.89 (m, 1H), 4.91 – 4.79 (m, 1H), 4.72 (d, *J* = 10.8 Hz, 1H), 4.57 (d, *J* = 8.1 Hz, 1H), 4.07 (dt, *J* = 12.6, 9.1 Hz, 1H), 4.01 – 3.90 (m, 3H), 3.91 – 3.79 (m, 3H), 3.74 (m, 2H), 3.61 (m, 1H), 3.52 – 3.40 (m, 2H), 3.21 (m, 2H), 1.71 (m, 2H), 1.06 (s, 9H). ^{19}F NMR (376 MHz, $CDCl_3$) δ -190.18. ^{13}C NMR (101 MHz, $CDCl_3$) δ 165.18, 156.46, 137.94, 136.59, 135.90, 135.65, 135.31, 133.48, 133.35, 133.13, 132.89, 129.91, 129.80, 129.76, 129.38, 128.52, 128.33, 128.13, 128.11, 128.03, 127.89, 127.85, 127.79, 127.71, 126.99, 126.12, 126.09, 126.02, 100.44, 100.33, 97.75, 96.45, 94.59, 79.99, 78.06, 77.38, 77.26, 77.06, 76.74, 75.72, 75.30, 73.76, 72.99, 72.49, 72.33, 72.07, 71.88, 67.57, 66.60, 63.64, 62.29, 61.41, 37.93, 29.56, 26.89, 19.34. HR-ESI-MS *(m/z)*: [M+H]+ calcd for $C_{64}H_{69}FN_4O_{12}Si$, 1133.4744; found, 1133.4743.

***N*-benzyloxycarbonyl-3-aminopropyl-*O*-[Methyl(2-azido-2-deoxy-3,4-*O*- benzyl-6-*O*-*tert*-butyldiphenylsilyl-α-D-glucopyranosyl)-(1→4)-*O*-2-*O*-benzoyl-3-*deoxy-3*-fluoro-β-D-gluco pyranosyluronate] (16a)**

A solution of Compound **13a** (donor, 1.5 g, 2.05 mmol) and Compound **12** (acceptor, 0.906 g, 1.64 mmol) in CH_2Cl_2 (50 mL) was stirred at room temperature in a round-bottom flask containing freshly dried 4 Å molecular sieves for 1 hour. The reaction mixture was then stirred under a nitrogen atmosphere and cooled to -78 °C, followed by the addition of NIS (0.553 g, 2.46 mmol) and TMSOTf (74 µL, 0.41 mmol). The temperature was gradually increased to -20 °C, and the reaction mixture was stirred for a further 15 minutes. Completion of the reaction was

monitored by TLC, after which it was quenched with a few drops of triethylamine (Et_3N). The molecular sieves were removed by filtration through Celite, and the organic layer was washed sequentially with aqueous sodium thiosulphate ($Na_2S_2O_3$) and brine. The organic phase was collected, dried over anhydrous Na_2SO_4, filtered, and concentrated under reduced pressure. The residue was purified by silica gel column chromatography (ethyl acetate/hexane) to afford Compound **14a** in 59% yield, which was taken as such for next step. A solution of Compound **14a** (1.0 g, 0.863 mmol) in a 1:1 mixture of pyridine and methanol (20 mL) was heated to 80 °C, and thiourea (0.131 g, 1.72 mmol) was added. The reaction mixture was stirred under a nitrogen atmosphere for 6 hours. Upon completion, the solvent was evaporated under reduced pressure, and the residue was dissolved in ethyl acetate and washed sequentially with 1 N HCl and brine. The organic layer was dried over anhydrous Na_2SO_4, filtered, and concentrated under reduced pressure. The crude product was purified by silica gel column chromatography (EtOAc/hexane, 1:6, v/v) to afford Compound **15a** in 79% yield, which was taken as such for next step. A solution of Compound **15a** (0.936 g, 0.847 mmol) in a 2:1 mixture of dichloromethane (DCM) and water (15 mL) was treated with TEMPO (0.079 g, 0.508 mmol) and BAIB (1.09 g, 3.388 mmol). The reaction mixture was stirred at room temperature for 10 hours. Upon completion, the organic layer was washed with saturated ammonium chloride (NH_4Cl) solution and concentrated under reduced pressure. The resulting crude product was dissolved in dimethylformamide (DMF, 10 mL) and treated with K_2CO_3 (0.350 g, 2.54 mmol) and MeI (0.482 mL, 7.62 mmol). The reaction mixture was stirred overnight at room temperature. After completion, the reaction mixture was extracted with ethyl acetate and brine. The organic layer was concentrated under reduced pressure, and the residue was purified by silica gel column chromatography (ethyl acetate/hexane) to afford Compound **16a** in 61% yield. ^{1}H NMR (400 MHz, Chloroform-*d*) δ

7.70 – 7.66 (m, 2H), 7.63 (m, 4H), 7.44 – 7.26 (m, 22H), 7.21 (m, 2H), 5.95 (d, *J* = 5.0 Hz, 1H), 5.12 (m, 2H), 5.05 (m, 2H), 4.92 – 4.79 (m, 4H), 4.73 (dd, *J* = 11.1, 5.4 Hz, 2H), 4.27 (dddd, *J* = 20.7, 8.5, 2.5, 1.1 Hz, 1H), 4.01 (d, *J* = 8.5 Hz, 1H), 3.91 – 3.78 (m, 3H), 3.69 (dd, *J* = 11.7, 1.7 Hz, 1H), 3.45 (s, 3H), 3.43 (m, 2H), 3.38 – 3.29 (m, 2H), 3.25 (q, *J* = 6.2 Hz, 2H), 1.72 (p, *J* = 6.5 Hz, 2H), 1.04 (s, 9H). ^{19}F NMR (376 MHz, $CDCl_3$) δ -184.56. ^{13}C NMR (101 MHz, $CDCl_3$) δ 168.68, 156.32, 138.18, 137.71, 136.56, 135.86, 135.58, 134.89, 133.48, 132.93, 129.71, 128.56, 128.53, 128.51, 128.42, 128.22, 128.11, 128.03, 127.75, 127.73, 127.61, 127.60, 126.11, 121.42, 97.67, 96.86, 89.29, 87.54, 79.95, 77.73, 77.34, 77.23, 77.02, 76.71, 75.66, 75.03, 73.52, 73.26, 73.08, 72.77, 69.29, 66.63, 63.42, 61.81, 52.61, 38.49, 29.36, 26.84, 19.34. HR-ESI-MS *(m/z)*: [M+Na]+ calcd for $C_{61}H_{67}ClN_4O_{13}Si$, 1110.4458; found, 1110.4653.

***N*-benzyloxycarbonyl-3-aminopropyl-*O*-[Methyl(2-azido-2-deoxy-3-*O*-naphthylmethyl-4-*O*-benzyl-6-*O*-*tert*-butyldiphenylsilyl-α-D-glucopyranosyl)-(1→4)-*O*-2-*O*-benzoyl-3-*deoxy-3-* -β-D-gluco pyranosyluronate] (16b)**

To a solution of compound **15b** (1.3 g, 1.14 mmol) in DCM/H_2O (2:1, 21 mL) were added 2,2,6,6-tetramethyl-1-piperidinyloxyl free radical (TEMPO, 0.106 g, 0.684 mmol) and [Bis(acetoxy)iodo] benzene (BAIB) (1.46 g, 4.56 mmol), and stirred at RT for 10 h. The organic layer was washed with saturated Brine solution and concentrated. Then, the crude product was dissolved in DMF (10 mL), added K_2CO_3 (0.471 g, 3.42 mmol), MeI (0.640 mL, 10.26 mmol), at 0 °C and stirred overnight at RT. After completion of the reaction, the reaction mixture was extracted with EtOAc and brine. The organic layer was concentrated and purified through silica gel column chromatography (EtOAc/Hexane) to obtain compound **16b** (65%, 882mg). ^{1}H NMR (400 MHz, Chloroform-*d*) δ 8.10 – 8.03 (m, 2H), 7.85 – 7.74 (m, 4H), 7.68 (ddt, *J* = 19.7, 6.7, 1.5 Hz, 4H), 7.62 – 7.53 (m, 1H), 7.54 – 7.36 (m, 9H), 7.38 – 7.26 (m, 10H), 7.21 (dd, *J* = 6.7,

3.3 Hz, 2H), 5.44 – 5.30 (m, 2H), 5.03 (s, 4H), 5.00 – 4.90 (m, 2H), 4.81 (q, *J* = 10.7 Hz, 2H), 4.60 (d, *J* = 7.6 Hz, 1H), 4.30 (ddd, *J* = 12.4, 9.7, 8.3 Hz, 1H), 4.05 – 3.97 (m, 2H), 3.96 – 3.91 (m, 2H), 3.90 – 3.81 (m, 2H), 3.53 (s, 3H), 3.49 (d, *J* = 8.6 Hz, 1H), 3.42 (dp, *J* = 6.9, 5.4 Hz, 1H), 3.19 (m, 1H), 3.09 (m, 1H), 1.72 – 1.64 (m, 2H), 1.06 (s, 9H). ^{19}F NMR (376 MHz, $CDCl_3$) δ -190.06. ^{13}C NMR (101 MHz, $CDCl_3$) δ 167.81, 167.78, 165.10, 156.47, 138.29, 136.77, 135.95, 135.63, 135.29, 133.61, 133.56, 133.36, 133.15, 132.89, 129.93, 129.83, 129.75, 129.18, 128.59, 128.51, 128.49, 128.35, 128.03, 127.79, 127.75, 127.73, 127.70, 127.48, 127.06, 126.14, 126.05, 100.50, 100.40, 97.85, 97.80, 95.53, 93.66, 79.76, 77.75, 77.42, 77.31, 77.10, 76.78, 75.68, 75.09, 73.95, 73.78, 73.18, 73.09, 72.71, 71.73, 71.53, 67.58, 66.48, 63.59, 61.80, 52.87, 37.82, 29.74, 29.37, 26.92, 19.41. HR-ESI-MS *(m/z)*: [M+H]+ calcd for $C_{65}H_{69}FN_4O_{13}Si$, 1183.4512; found, 1183.4512.

N-benzyloxycarbonyl-3-aminopropyl-*O*-[methyl(2-acetamido-2-deoxy-3,4-*O*-benzyl-6-*O*-*tert*-butyldiphenylsilyl-*α*-D-glucopyranosyl)]-(1→4)-*O*-2-*O*-benzoyl-3-deoxy-3-fluoro-β-D-glucopyranosiduronate (17a)

To a solution of compound **16a** (1.2 g, 1.03 mmol) in THF (6 mL), AcOH (4 mL) and Ac_2O (2 mL) was added Zn dust (1.3 g, 20.6 mmol) and stirred for 12 h at room temperature. The reaction mixture was filtered through celite, and the solvent was evaporated upon completion. The remaining residue was extracted with ethyl acetate, saturated $NaHCO_3$, and washed with brine. The collected organic layer was dried over Na_2SO_4, filtered, concentrated, and purified through silica gel column chromatography (ethyl acetate/ hexane) to obtain compound **17a** (858 mg, 70.09 %). ^{1}H NMR (400 MHz, Chloroform-*d*) δ 8.06 – 8.00 (m, 2H), 7.68 (m, 4H), 7.59 (t, *J* = 7.4 Hz, 1H), 7.48 – 7.29 (m, 21H), 7.21 (m, 2H), 5.41 – 5.27 (m, 2H), 5.07 – 5.01 (m, 3H), 4.94 – 4.86 (m, 2H), 4.84 – 4.73 (m, 2H), 4.67 – 4.60 (m, 1H), 4.56 (d, *J* = 7.5 Hz, 1H), 4.30 – 4.12

(m, 2H), 4.07 – 3.99 (m, 2H), 3.89 – 3.77 (m, 3H), 3.69 – 3.61 (m, 1H), 3.58 – 3.47 (m, 2H), 3.40 (s, 3H), 3.24 – 3.04 (m, 2H), 1.83 (s, 3H), 1.76 – 1.65 (m, 2H), 1.07 (s, 9H). ^{19}F NMR (376 MHz, $CDCl_3$) δ -192.60. ^{13}C NMR (101 MHz, $CDCl_3$) δ 170.02, 166.98, 164.94, 156.42, 138.37, 136.69, 135.96, 135.69, 133.64, 133.49, 132.93, 129.85, 129.74, 129.70, 129.07, 128.59, 128.52, 128.49, 128.45, 128.19, 128.04, 127.88, 127.75, 127.71, 127.66, 100.47, 100.26, 92.53, 79.82, 77.35, 77.04, 76.72, 74.99, 74.94, 74.16, 73.22, 71.55, 67.61, 66.49, 61.90, 52.86, 52.77, 37.78, 29.36, 26.92, 23.34, 19.34. HR-ESI-MS *(m/z)*: [M+Na]+ calcd for $C_{61}H_{67}FN_4O_{13}Si$, 1133.4656; found, 1133.4652.

N-benzyloxycarbonyl-3-aminopropyl-*O*-[methyl(2-acetamido-2-deoxy-3-*O*-naphthylmethyl-4-*O*-benzyl-6-*O*-*tert*-butyldiphenylsilyl)-*α*-D-glucopyranosyl)]-(1→4)-*O*-2-*O*-benzoyl-3-deoxy-3-fluoro-β-D-glucopyranosiduronate (17b)

To a solution of compound **16b** (1.6 g, 1.35 mmol) in THF (9 mL), AcOH (6 mL) and Ac_2O (3 mL) was added Zn dust (1.7 g, 27 mmol) and stirred for 12 h at room temperature. The reaction mixture was filtered through celite, and the solvent was evaporated upon completion. The remaining residue was extracted with ethyl acetate, saturated $NaHCO_3$, and washed with brine. The collected organic layer was dried over Na_2SO_4, filtered, concentrated, and purified through silica gel column chromatography (ethyl acetate/ hexane) to obtain compound **17b** (1.17 mg, 73.52 %). ^{1}H NMR (400 MHz, Chloroform-*d*) δ 8.08 – 7.98 (m, 2H), 7.81 (m, 3H), 7.76 – 7.65 (m, 5H), 7.59 (t, *J* = 7.4 Hz, 1H), 7.47 – 7.30 (m, 19H), 7.24 (m, 2H), 5.37 – 5.27 (m, 2H), 5.11 – 5.02 (m, 3H), 4.96 (m, 2H), 4.87 – 4.76 (m, 2H), 4.55 (d, *J* = 7.4 Hz, 2H), 4.34 – 4.25 (m, 1H), 4.22 – 4.12 (m, 1H), 4.10 – 4.02 (m, 2H), 3.88 – 3.81 (m, 2H), 3.71 (dd, *J* = 10.7, 9.0 Hz, 1H), 3.62 – 3.48 (m, 2H), 3.41 (s, 3H), 3.11 (m, 2H), 1.76 (s, 3H), 1.69 (q, *J* = 6.4 Hz, 2H), 1.09 (s, 9H). ^{19}F NMR (376 MHz, $CDCl_3$) δ -192.58. ^{13}C NMR (101 MHz, $CDCl_3$) δ 170.06, 167.05,

167.02, 164.92, 156.43, 138.38, 136.71, 135.98, 135.83, 135.70, 133.64, 133.50, 133.27, 133.00, 132.94, 129.86, 129.76, 129.72, 129.09, 128.58, 128.50, 128.47, 128.28, 128.04, 128.02, 127.93, 127.76, 127.73, 127.70, 127.68, 127.38, 126.90, 126.25, 126.22, 126.05, 100.56, 100.45, 100.23, 94.41, 92.54, 79.61, 77.57, 77.39, 77.28, 77.07, 76.76, 75.02, 74.94, 74.10, 74.02, 73.24, 71.72, 71.53, 67.61, 66.49, 61.92, 52.85, 52.79, 37.79, 36.67, 29.72, 29.37, 26.94, 23.33, 19.36. HR-ESI-MS *(m/z)*: [M+H]+ calcd for $C_{67}H_{73}FN_2O_{14}Si$, 1177.4893; found, 1177.4893.

***N*-benzyloxycarbonyl-3-aminopropyl-*O*-[methyl(2-acetamido-2-deoxy-3,4-di-*O*-benzyl)-*α*-D-glucopyranosyl)]-(1→4)-*O*-2-*O*-benzoyl-3-deoxy-3-fluoro-1-β-D-glucopyranosiduronate (17a-i)**

Disaccharide **17a** (360 mg, 0.324 mmol) was subjected to the TBDPS deprotection described in general procedures to obtain disaccharide **17a-i** (200 mg, 71 %).

^{1}H NMR (400 MHz, Chloroform-*d*) δ 8.11 – 7.98 (m, 2H), 7.59 (t, *J* = 7.5 Hz, 1H), 7.45 (t, *J* = 7.7 Hz, 2H), 7.32 (m, 15H), 5.39 – 5.27 (m, 2H), 5.05 (s, 2H), 5.02 (d, *J* = 3.7 Hz, 1H), 4.90 (m, 1H), 4.87 – 4.72 (m, 2H), 4.70 – 4.57 (m, 3H), 4.29 – 4.15 (m, 2H), 3.94 (d, *J* = 9.7 Hz, 1H), 3.89 (m, 1H), 3.79 (s, 3H), 3.72 – 3.65 (m, 1H), 3.64 – 3.60 (m, 2H), 3.54 (m, 1H), 3.20 (p, *J* = 6.6 Hz, 1H), 3.12 (p, *J* = 6.5 Hz, 1H), 1.96 (t, *J* = 6.7 Hz, 1H), 1.80 (s, 3H), 1.71 (q, *J* = 6.2 Hz, 2H). ^{19}F NMR (376 MHz, Chloroform-*d*) δ -192.92. ^{13}C NMR (101 MHz, Chloroform-*d*) δ 170.02, 167.43, 164.95, 156.35, 138.19, 137.85, 133.68, 129.85, 129.02, 128.60, 128.55, 128.54, 128.50, 128.14, 128.07, 128.03, 127.94, 100.68, 100.57, 99.99, 79.40, 77.65, 77.35, 77.03, 76.71, 75.13, 74.82, 74.19, 74.12, 73.22, 71.83, 71.64, 67.75, 66.53, 61.56, 53.23, 52.62, 29.37, 23.24,. HR-ESI-MS *(m/z)*: [M+H]+ calcd for $C_{47}H_{53}FN_2O_{14}$, 888.3559; found, 888.3559.

***N*-benzyloxycarbonyl-3-aminopropyl-*O*-[methyl(2-acetamido-2-deoxy-4-*O*-benzyl-6-*O*-*tert*-butyldiphenylsilyl-*α*-D-glucopyranosyl)]-(1→4)-*O*-2-*O*-benzoyl-3-deoxy-3-fluoro-β-D-glucopyranosiduronate(17b-i)**

Disaccharide **17b** (325 mg, 0.27 mmol) was subjected to the NAP deprotection described in general procedures to give disaccharide **17b-i** (208 mg, 72.7 %).

^{1}H NMR (400 MHz, Chloroform-*d*) δ 8.10 – 7.87 (m, 2H), 7.68 (m, 4H), 7.61 – 7.54 (m, 1H), 7.48 – 7.36 (m, 6H), 7.36 – 7.25 (m, 12H), 6.06 (dd, *J* = 8.9, 2.7 Hz, 1H), 5.34 (m, 1H), 5.09 (d, *J* = 3.7 Hz, 1H), 5.03 (s, 2H), 4.98 (m, 2H), 4.87 – 4.64 (m, 2H), 4.54 (d, *J* = 7.6 Hz, 1H), 4.29 – 4.09 (m, 2H), 4.03 (dd, *J* = 11.6, 2.6 Hz, 1H), 3.93 – 3.71 (m, 5H), 3.61 – 3.47 (m, 2H), 3.41 (s, 3H), 3.31 (d, *J* = 4.5 Hz, 1H), 3.13 (m, 2H), 2.01 (s, 3H), 1.75 – 1.60 (m, 2H), 1.08 (s, 9H). ^{19}F NMR (376 MHz, $CDCl_3$) δ -192.20. ^{13}C NMR (101 MHz, $CDCl_3$) δ 171.89, 167.23, 165.00, 156.47, 138.68, 136.70, 135.93, 135.68, 133.70, 133.48, 133.08, 129.88, 129.77, 129.73, 129.04, 128.62, 128.51, 128.45, 128.06, 128.01, 127.76, 127.69, 127.66, 127.63, 100.55, 100.44, 94.72, 92.85, 77.82, 77.44, 77.33, 77.13, 76.81, 74.99, 73.97, 73.89, 72.80, 71.70, 71.51, 67.64, 66.49, 62.09, 54.33, 52.92, 37.79, 29.72, 29.38, 26.93, 23.22, 19.38. HR-ESI-MS *(m/z)*: [M+H]+ calcd for $C_{56}H_{65}FN_2O_{14}Si$, 1037.4267; found, 1037.4267.

***N*-benzyloxycarbonyl-3-aminopropyl-*O*-[Benzyl(2-acetamido-2-deoxy-4-*O*-benzyl-*α*-D-glucopyranosyl)]-(1→4)-*O*-3-deoxy-3-fluoro-β-D-glucopyranosiduronate(17b-ii)**

Disaccharide **17b** (800 mg, 0.68 mmol) was subjected to NAP deprotection, TBDPS deprotection, and saponification according to the general procedures to give crude disaccharide. Then, the crude product was dissolved in DMF (10 mL), mixed with $NaHCO_3$ (142 mg, 1.7 mmol), BnBr (0.201 mL, 1.7 mmol), TBAI (153 mg, 0.47 mmol), and stirred for 2 h at 60 °C.

After completion of the reaction, the reaction mixture was extracted with EtOAc and brine. The organic layer was concentrated and purified through silica gel column chromatography (EtOAc/Hexane) and yielded compound **17b-ii** (235 mg, 44.81 %, four steps).

^{1}H NMR (400 MHz, Chloroform-*d*) δ 7.43 – 7.11 (m, 15H), 5.31 (t, *J* = 6.3 Hz, 1H), 5.17 (d, *J* = 12.1 Hz, 1H), 5.14 – 5.06 (m, 2H), 5.03 (s, 2H), 4.90 (d, *J* = 11.2 Hz, 1H), 4.67 (d, *J* = 11.2 Hz, 1H), 4.53 (dt, *J* = 52.0, 8.7 Hz, 1H), 4.25 (d, *J* = 7.6 Hz, 1H), 4.17 – 3.97 (m, 2H), 3.86 (d, *J* = 9.4 Hz, 2H), 3.83 – 3.71 (m, 2H), 3.65 (dd, *J* = 14.2, 8.9 Hz, 2H), 3.52 (d, *J* = 4.8 Hz, 3H), 3.37 (dq, *J* = 13.5, 6.7 Hz, 1H), 3.18 (dq, *J* = 12.0, 5.8 Hz, 1H), 1.96 (s, 3H), 1.79 – 1.60 (m, 2H). ^{19}F NMR (376 MHz, Chloroform-*d*) δ -192.09. ^{13}C NMR (101 MHz, Chloroform-*d*) δ 172.36, 167.53, 156.94, 139.31, 138.38, 136.50, 134.54, 128.68, 128.63, 128.61, 128.55, 128.45, 128.16, 128.10, 127.83, 114.11, 102.61, 102.49, 98.78, 97.10, 95.27, 77.81, 77.42, 77.31, 77.10, 76.79, 76.49, 76.32, 74.93, 73.60, 72.77, 72.45, 71.80, 71.62, 68.12, 67.37, 66.76, 61.35, 54.23, 37.58, 33.85, 31.95, 31.67, 29.71, 29.68, 29.64, 29.55, 29.38, 29.18, 28.97, 22.97, 22.71, 14.16.

HR-ESI-MS *(m/z)*: [M+H]+ calcd for $C_{39}H_{47}FN_2O_{13}$, 771.3140; found, 771.3137.

N-benzyloxycarbonyl-3-aminopropyl-*O*-[methyl(2-acetamido-2-deoxy-3,4-di-*O*-benzyl-6-*O*-sulfonate)-*α*-D-glucopyranosyl)]-(1→4)-*O*-2-*O*-benzoyl-3-deoxy-3-fluoro-β-D-glucopyranosiduronate (18)

Disaccharide **17a-i** (145 mg, 0.16 mmol) was subjected to *O*-sulfation as described in general procedures to obtain disaccharide **18** (103 mg, 65.18 %).

^{1}H NMR (400 MHz, Methanol-d_4) δ 8.12 (d, *J* = 7.8 Hz, 2H), 7.62 (t, *J* = 7.4 Hz, 1H), 7.49 (t, *J* = 7.6 Hz, 2H), 7.43 – 7.14 (m, 15H), 5.40 – 5.24 (m, 1H), 5.18 (d, *J* = 3.7 Hz, 1H), 5.05 (s, 2H), 4.98 – 4.91 (m, 2H), 4.81 (d, *J* = 11.1 Hz, 2H), 4.48 (d, *J* = 10.5 Hz, 1H), 4.43 – 4.16 (m, 4H),

3.94 – 3.83 (m, 2H), 3.82 (s, 3H), 3.76 (d, *J* = 6.9 Hz, 2H), 3.62 (m, 1H), 3.18 (q, *J* = 7.3 Hz, 2H), 3.09 (t, *J* = 6.8 Hz, 2H), 1.99 (s, 3H), 1.73 – 1.61 (m, 2H). ^{19}F NMR (376 MHz, Methanol-d_4) δ -192.92. ^{13}C NMR (101 MHz, Methanol-d_4) δ 176.13, 172.55, 169.40, 161.26, 142.59, 142.25, 140.92, 137.47, 133.45, 133.07, 132.42, 132.12, 131.98, 131.92, 131.60, 131.38, 131.33, 131.30, 131.26, 104.22, 104.11, 102.49, 99.18, 97.33, 83.61, 81.54, 79.93, 79.75, 78.75, 78.65, 76.85, 76.77, 75.92, 75.74, 74.49, 71.49, 69.90, 69.44, 57.03, 56.40, 52.38, 52.17, 51.96, 51.75, 51.53, 51.32, 51.11, 50.55, 41.15, 35.63, 33.59, 33.36, 33.03, 26.32, 25.48, 12.02. HR-ESI-MS *(m/z)*: [M]+ calcd for $C_{47}H_{52}FN_2O_{17}S^-$, 967.2976; found, 967.2995

N-benzyloxycarbonyl-3-aminopropyl-*O*-[methyl(2-acetamido-2-deoxy-3-*O*-sulfonate-4-*O*-benzyl-6-*O*-*tert*-butyldiphenylsilyl)-*α*-D-glucopyranosyl)]-(1→4)-*O*-2-*O*-benzoyl-3-deoxy-3-fluoro-β-D-glucopyranosiduronate (19)

Disaccharide **17b-i** (187 mg, 0.18 mmol) was subjected to *O*-sulfation as described in general procedures to obtain disaccharide **19** (107 mg, 54.31%).

^{1}H NMR (400 MHz, Methanol-d_4) δ 7.98 – 7.92 (m, 2H), 7.69 – 7.57 (m, 3H), 7.57 – 7.45 (m, 3H), 7.38 – 7.17 (m, 12H), 7.17 – 7.09 (m, 5H), 5.31 (d, *J* = 3.3 Hz, 1H), 5.15 (dd, *J* = 12.4, 8.5 Hz, 2H), 4.87 (s, 2H), 4.70 – 4.56 (m, 2H), 4.46 (d, *J* = 10.7 Hz, 1H), 4.07 (m, 2H), 3.92 – 3.80 (m, 2H), 3.76 (m, 1H), 3.72 – 3.65 (m, 2H), 3.47 (m, 1H), 3.43 (s, 3H), 3.42 – 3.37 (m, 1H), 2.91 (q, *J* = 6.7 Hz, 2H), 1.87 (s, 3H), 1.50 (p, *J* = 7.0, 5.4 Hz, 2H), 0.96 (s, 9H). ^{19}F NMR (376 MHz, Methanol-d_4) δ -192.97. ^{13}C NMR (101 MHz, Methanol-d_4) δ 172.54, 168.43, 165.37, 157.33, 138.57, 136.94, 135.67, 135.40, 133.47, 133.32, 132.88, 129.58, 129.46, 129.15, 128.42, 128.12,

128.01, 127.85, 127.63, 127.54, 127.45, 127.38, 127.20, 100.18, 100.07, 97.67, 95.08, 93.23, 77.46, 76.12, 75.52, 75.35, 74.78, 72.89, 72.78, 71.94, 71.76, 67.34, 65.96, 62.08, 54.05, 52.24, 48.59, 48.38, 48.16, 47.95, 47.74, 47.53, 47.31, 47.10, 37.14, 29.35, 29.08, 28.70, 26.12, 23.56, 22.67, 21.62, 18.80. HR-ESI-MS *(m/z)*: [M+Na]+ calcd for $C_{56}H_{64}FN_2O_{17}SSi^-$, 1115.3684; found, 1115.3745

N-benzyloxycarbonyl-3-aminopropyl-*O*-[Benzyl(2-acetamido-2-deoxy-3-*O*-sulfonate-4-*O*-benzyl-6-*O*-sulfonate)-*α*-D-glucopyranosyl)]-(1→4)-*O*-2-*O*-sulfonate-3-deoxy-3-fluoro-β-D-glucopyranosiduronate (20)

Disaccharide **17b-ii** (158 mg, 0.206 mmol) was subjected to *O*-sulfation as described in general procedures to obtain disaccharide **20** (86 mg, 41.26 %).

^{1}H NMR (400 MHz, Methanol-d_4) δ 7.58 – 7.52 (m, 2H), 7.36 – 7.26 (m, 9H), 7.22 (dd, *J* = 5.1, 2.1 Hz, 3H), 5.31 (d, *J* = 3.5 Hz, 1H), 5.23 – 5.10 (m, 3H), 5.05 (s, 1H), 4.90 (t, *J* = 8.0 Hz, 1H), 4.69 – 4.62 (m, 2H), 4.33 (dd, *J* = 10.9, 2.8 Hz, 3H), 4.22 – 4.15 (m, 2H), 4.03 (dt, *J* = 11.2, 3.0 Hz, 1H), 3.77 (ddd, *J* = 18.7, 10.1, 6.8 Hz, 2H), 3.70 – 3.54 (m, 2H), 3.21 (t, *J* = 6.1 Hz, 2H), 2.04 (s, 3H), 1.80 – 1.63 (m, 2H). ^{19}F NMR (376 MHz, Methanol-d_4) δ -191.20. ^{13}C NMR (101 MHz, Methanol-d_4) δ 173.22, 168.75, 157.71, 138.13, 136.81, 134.73, 129.21, 128.42, 128.41, 128.34, 128.27, 127.94, 127.79, 127.56, 127.47, 100.78, 100.68, 97.83, 95.40, 93.55, 77.67, 77.49, 77.30, 75.61, 75.13, 74.95, 74.85, 72.70, 72.62, 70.48, 67.98, 67.40, 66.18, 65.53, 53.25, 48.52, 48.31, 48.10, 47.88, 47.67, 47.46, 47.24, 37.19, 29.77, 29.03, 21.84, 21.80. HR-ESI-MS *(m/z)*: [M-3H] $^{-3}$ calcd for $C_{39}H_{44}FN_2O_{22}S_3^{3-}$, 335.3183; found, 335.3186.

3-aminopropyl-*O*-[2-acetamido-2-deoxy-*α*-D-glucopyranosyl)]-(1→4)-*O*-3-deoxy-3-fluoro-β-D-glucopyranosiduronate (HDF-1)

Disaccharide **17a-i** (67 mg, 0.07 mmol) was subjected to Saponification to obtain compound 18a. Compound 18a was followed by Global deprotection as described in the General procedure to yield disaccharide **HDF-1** (16 mg, 47.05 %, two steps).

^{1}H NMR (400 MHz, D_2O) δ 5.20 (d, *J* = 3.7 Hz, 1H), 4.74 – 4.51 (m, 2H), 4.15 – 3.92 (m, 2H), 3.90 – 3.79 (m, 5H), 3.78 – 3.60 (m, 3H), 3.51 (t, *J* = 9.6 Hz, 1H), 3.16 (td, *J* = 6.8, 2.3 Hz, 2H), 2.05 (s, 3H), 2.00 (q, *J* = 6.3 Hz, 2H). ^{19}F NMR (376 MHz, D_2O) δ -192.88. ^{13}C NMR (101 MHz, D_2O) δ 174.53, 174.28, 101.50, 101.38, 98.09, 97.02, 96.97, 96.28, 75.01, 74.97, 74.90, 74.86, 72.01, 71.45, 71.27, 70.34, 69.51, 68.09, 64.86, 59.90, 53.64, 37.54, 26.69, 21.74, 18.45. HR-ESI-MS *(m/z)*: [M]+ calcd for $C_{17}H_{29}FN_2O_{11}$, 456.1755; found, 456.1753.

3-aminopropyl-*O*-[2-acetamido-2-deoxy-6-*O*-sulfonate-*α*-D-glucopyranosyl)]-(1→4)-*O*-3-deoxy-3-fluoro-β-D-glucopyranosiduronate (HDF-2)

Disaccharide **18** (67 mg, 0.069 mmol) was subjected to Saponification followed by Global deprotection as described in the General procedure to yield disaccharide **HDF-2** (13 mg, 52.9 %, two steps).

^{1}H NMR (600 MHz, Deuterium Oxide) δ 5.11 (d, *J* = 3.8 Hz, 1H), 4.62 – 4.48 (m, 1H), 4.46 (dd, *J* = 8.0, 1.2 Hz, 1H), 4.31 – 4.22 (m, 1H), 4.08 (dt, *J* = 11.1, 1.7 Hz, 1H), 3.91 (m, 2H), 3.86 – 3.72 (m, 4H), 3.68 – 3.56 (m, 2H), 3.55 – 3.47 (m, 1H), 3.08 (p, *J* = 6.3 Hz, 2H), 1.99 – 1.91 (s, 3H), 1.94 – 1.86 (m, 2H). ^{19}F NMR (564 MHz, Deuterium Oxide) δ -194.09. ^{13}C NMR (151 MHz, Deuterium Oxide) δ 174.48, 173.93, 101.46, 101.38, 97.64, 97.01, 96.97, 96.43, 74.94,

74.83, 74.61, 74.56, 71.36, 71.24, 70.23, 69.00, 68.09, 66.24, 64.86, 53.45, 37.56, 26.67, 21.72, 18.43. HR-ESI-MS *(m/z)*: [M]+ calcd for $C_{17}H_{28}FN_2O_{14}S$-, 535.1251; found, 535.1243

3-aminopropyl-*O*-[2-acetamido-2-deoxy-3-*O*-sulfonate-*α*-D-glucopyranosyl]-(1→4)-*O*-3-deoxy-3-fluoro-β-D-glucopyranosiduronate (HDF-3)

Disaccharide **19** (134 mg, 0.035 mmol) was subjected to TBDPS deprotetion followed by Saponification, Global deprotection as described in the General procedure to yield disaccharide **HDF-3** (17 mg, 26.3%, three steps).

^{1}H NMR (600 MHz, Deuterium Oxide) δ 5.15 (d, *J* = 3.8 Hz, 1H), 4.61 (dt, *J* = 52.8, 8.8 Hz, 1H), 4.47 (d, *J* = 8.0 Hz, 1H), 4.39 (dd, *J* = 10.7, 9.0 Hz, 1H), 3.98 (dd, *J* = 10.8, 3.9 Hz, 1H), 3.95 – 3.87 (m, 2H), 3.80 – 3.74 (m, 3H), 3.71 (m, 2H), 3.60 (m, 2H), 3.07 (m, 2H), 1.95 (s, 3H), 1.92 (p, *J* = 6.5 Hz, 2H). ^{19}F NMR (564 MHz, Deuterium Oxide) δ -192.84. ^{13}C NMR (151 MHz, Deuterium Oxide) δ 174.44, 174.21, 101.42, 101.34, 97.60, 97.29, 97.25, 96.39, 79.23, 75.30, 75.19, 74.90, 74.85, 71.84, 71.42, 71.30, 68.08, 68.04, 64.86, 59.76, 52.21, 37.51, 26.67, 21.90, 18.43. HR-ESI-MS *(m/z)*: [M]+ calcd for $C_{17}H_{28}FN_2O_{14}S^-$, 535.1251; found, 535.1251.

3-aminopropyl-*O*-[2-acetamido-2-deoxy-3-*O*-sulfonate-6-*O*-sulfonate-*α*-D-glucopyranosyl]-(1→4)-*O*-2-*O*-sulfonate-3-deoxy-3-fluoro-β-D-glucopyranosiduronate (HDF-4)

Disaccharide **20** (56 mg, 0.05 mmol) was subjected to Global deprotection as described in General procedure to yield disaccharide **HDF-4** (23 mg, 60.52%).

^{1}H NMR (400 MHz, D_2O) δ 5.16 (d, *J* = 3.8 Hz, 1H), 4.86 (t, *J* = 8.7 Hz, 1H), 4.63 (d, *J* = 7.8 Hz, 1H), 4.39 (dd, *J* = 10.7, 9.0 Hz, 1H), 4.36 – 4.22 (m, 2H), 4.11 (dd, *J* = 11.5, 1.6 Hz, 1H), 4.07 – 3.96 (m, 3H), 3.89 (d, *J* = 10.2 Hz, 1H), 3.83 – 3.64 (m, 3H), 3.11 (t, *J* = 6.4 Hz, 2H), 1.96 (s, 3H), 1.91 (m,2H). ^{19}F NMR (376 MHz, D_2O) δ -190.94. ^{13}C NMR (101 MHz, D_2O) δ 174.53,

99.85, 99.78, 97.46, 97.43, 95.62, 94.38, 79.04, 77.71, 77.59, 75.43, 75.33, 70.12, 68.65, 67.56, 66.09, 52.01, 38.08, 26.40, 21.94, 18.40. HR-ESI-MS *(m/z)*: $[M\text{-}3H]^{-3}$ calcd $C_{17}H_{26}FN_2O_{20}S_3^{3-}$, 231.0080; found, 231.0087.

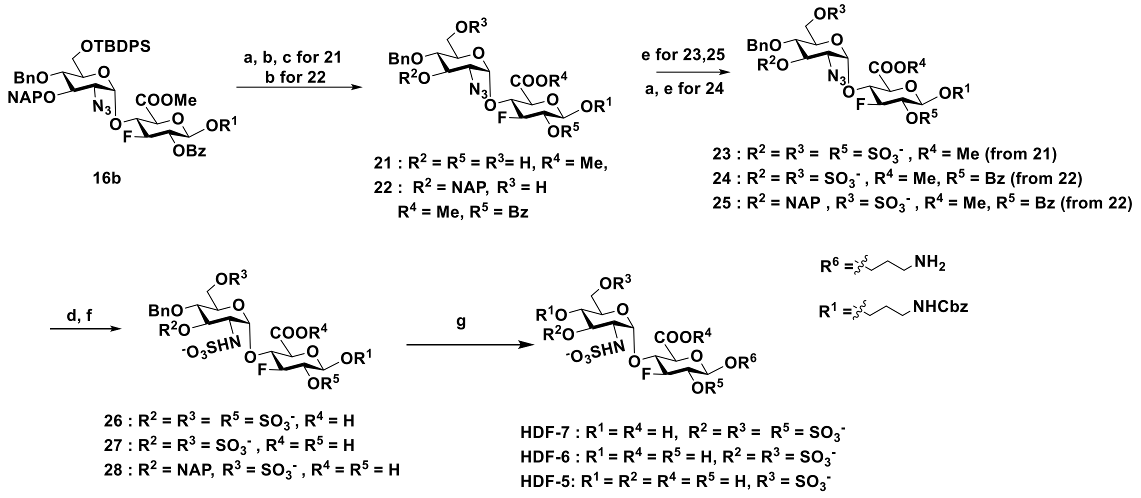

Scheme 3. Synthesis of fluorinated *N*-sulfated HS disaccharides: (a) DDQ, DCM:H_2O (18:1), RT, 1 h; (b) HF.Py, Py, 0 °C, 12 h; (c) NaOMe, MeOH, RT, 6h; (d) LiOH, H_2O:THF(1:1), RT, 12 h; (e) SO_3. Et_3N, DMF, 60 °C, 48-60 h; (f) (i) PMe_3.THF, RT, 24 h; (ii) SO_3.Py. MeOH, 1 M NaOH, 0 °C, 48 h; (g) H_2, $Pd(OH)_2$, H_2O, RT, 48 h.

***N*-benzyloxycarbonyl-3-aminopropyl-*O*-[methyl(2-azido-2-deoxy-4-*O*-Benzyl-α-D-glucopyranosyl)-(1→4)-*O*-3-deoxy-3-fluoro-β-D-gluco pyranosyluronate] (21)**

Disaccharide **16b** (600 mg, 0.517 mmol) was subjected to TBDPS deprotection followed by NAP deprotection as described in general procedures. Then, the crude product was subjected to Bz deprotection done by NaOMe (2 eq) in MeOH/DCM (1:1) to obtain disaccharide **21** (230 mg, 52.61 %, three steps).

^{1}H NMR (400 MHz, Chloroform-*d*) δ 7.30 – 6.88 (m, 10H), 5.14 (d, *J* = 3.6 Hz, 2H), 4.87 (s, 2H), 4.64 (s, 1H), 4.58 – 4.34 (m, 2H), 4.18 (s, 1H), 4.09 – 3.90 (m, 2H), 3.84 – 3.75 (m, 1H), 3.70 (d, *J* = 9.7 Hz,2), 3.66 – 3.55 (m, 2H), 3.54 (s, 3H), 3.52 – 3.42 (m, 2H), 3.39 – 3.31 (m, 2H), 3.29 (dd, *J* = 9.9, 8.3 Hz, 1H), 3.26 – 3.16 (m, 3H), 3.07 – 2.93 (m, 2H), 1.53 (ddd, *J* = 20.0, 14.9, 8.7 Hz, 2H). ^{19}F NMR (376 MHz, $CDCl_3$) δ -190.47. ^{13}C NMR (101 MHz, $CDCl_3$) δ 168.25, 168.22, 156.90, 137.97, 136.45, 128.70, 128.57, 128.21, 128.18, 128.02, 102.53, 102.41, 97.65, 97.61, 95.78, 77.60, 77.37, 77.25, 77.05, 76.73, 74.95, 74.26, 74.08, 73.03, 72.94, 71.94, 71.84, 71.76, 71.28, 67.32, 66.90, 63.02, 61.32, 60.45, 53.04, 37.47, 29.61, 21.07, 14.21.

HR-ESI-MS *(m/z)*: [M+Na]+ calcd for $C_{31}H_{39}FN_4O_{12}$, 701.2446; found, 701.2446.

***N*-benzyloxycarbonyl-3-aminopropyl-*O*-[Methyl(2-azido-2-deoxy-3-*O*-naphthylmethyl-4-*O*-benzyl-α-D-glucopyranosyl)-(1→4)-*O*-2-*O*-benzoyl-3-deoxy-3-fluoro-β-D-gluco pyranosyluronate] (22)**

Disaccharide **16b** (700 mg, 0.603 mmol) was subjected to the TBDPS deprotection described in general procedures to obtain disaccharide **22** (345 mg, 62 %).

^{1}H NMR (400 MHz, Chloroform-*d*) δ 8.06 – 7.99 (m, 2H), 7.79 (m, 4H), 7.58 – 7.37 (m, 6H), 7.34 – 7.24 (m, 10H), 5.48 (d, *J* = 3.7 Hz, 1H), 5.40 (m,2H), 5.03 (d, *J* = 2.4 Hz, 2H), 5.01 (d, *J*

= 1.5 Hz, 1H), 4.99 – 4.77 (m, 2H), 4.71 (d, *J* = 11.1 Hz, 1H), 4.64 (d, *J* = 7.6 Hz, 1H), 4.53 – 4.38 (m, 1H), 4.06 (m, 1H), 4.01 – 3.85 (m, 3H), 3.78 (s, 3H), 3.75 – 3.63 (m, 2H), 3.57 – 3.49 (m, 1H), 3.45 (m, 1H), 3.24 (m, 1H), 3.18 – 3.07 (m, 1H), 2.52 (m, 1H), 1.74 – 1.61 (m, 2H). ^{19}F NMR (376 MHz, $CDCl_3$) δ -190.28. ^{13}C NMR (101 MHz, $CDCl_3$) δ 168.23, 165.05, 156.59, 138.11, 136.83, 135.35, 133.59, 133.34, 133.11, 129.95, 129.05, 128.53, 128.50, 128.47, 128.27, 128.02, 127.96, 127.85, 127.72, 127.70, 126.96, 126.13, 126.11, 126.00, 100.56, 100.45, 97.72, 97.66, 95.70, 93.83, 79.82, 77.62, 77.42, 77.31, 77.10, 76.79, 75.58, 75.08, 73.53, 73.35, 72.89, 72.80, 72.72, 71.54, 71.35, 67.52, 66.40, 63.39, 60.66, 53.18, 37.64, 29.74, 29.20, 14.24. HR-ESI-MS *(m/z)*: [M+H]+ calcd for $C_{49}H_{51}FN_4O_{13}$, 923.3515; found, 923.3515.

***N*-benzyloxycarbonyl-3-aminopropyl-*O*-[Methyl(2-azido-2-deoxy-4-*O*-Benzyl-3-*O*-sulfonate-6-*O*-sulfonate α-D-glucopyranoside)-(1→4)-*O*-2-*O*-sulfonate-3-deoxy-3-fluoro-β-D-gluco pyranosyluronate](23)**

Disaccharide **21** (210 mg, 0.299 mmol) was subjected to *O*-sulfation as described in general procedures to obtain disaccharide **23** (195 mg, 71.2 %).

^{1}H NMR (400 MHz, Methanol-d_4) δ 7.49 – 7.22 (m, 10H), 5.29 (d, *J* = 3.7 Hz, 1H), 5.08 (s, 2H), 4.80 (d, *J* = 10.4 Hz, 2H), 4.53 (t, *J* = 8.6 Hz, 1H), 4.40 (d, *J* = 7.8 Hz, 1H), 4.32 (dd, *J* = 10.6, 2.0 Hz, 1H), 4.19 (d, *J* = 10.6 Hz, 1H), 4.15 – 4.07 (m, 1H), 4.04 (d, *J* = 9.8 Hz, 1H), 3.95 – 3.81 (m, 2H), 3.75 (s, 3H), 3.65 – 3.47 (m, 4H), 3.28 – 3.16 (m, 3H), 1.79 (p, *J* = 6.4 Hz, 2H). ^{19}F NMR (376 MHz, Methanol-d_4) δ -192.27. ^{13}C NMR (101 MHz, Methanol-d_4) δ 169.17, 169.15, 157.64, 157.58, 138.42, 136.99, 128.16, 128.00, 127.93, 127.84, 127.78, 127.67, 127.47, 127.43,

127.37, 102.28, 102.20, 97.85, 97.81, 97.42, 96.20, 77.57, 74.84, 74.73, 74.59, 74.48, 72.63, 72.57, 71.74, 71.62, 71.21, 70.28, 67.37, 66.42, 66.25, 66.07, 65.65, 65.35, 63.45, 63.11, 54.00, 52.39, 48.64, 48.22, 48.08, 47.94, 47.79, 47.65, 47.51, 47.37, 38.41, 38.09, 37.38, 37.25, 29.56, 29.39, 29.25. HR-ESI-MS *(m/z)*: $[M-3H]^{-3}$ calcd for $C_{31}H_{36}FN_4O_{21}S_3^{3-}$, 305.0345 found, 305.0342.

***N*-benzyloxycarbonyl-3-aminopropyl-*O*-[Methyl(2-azido-2-deoxy-4-*O*-Benzyl-3-*O*-sulfonate-6-*O*-sulfonate α-D-glucopyranoside)-(1→4)-*O*-2-*O*-benzoyl-3-deoxy-3-fluoro-β-D-gluco pyranosyluronate(24)**

Disaccharide **22** (232 mg, 0.25 mmol) was subjected to the sequence of NAP deprotection and *O*-sulfation described in general procedures to obtain disaccharide **24**(184 mg, 78.15%, two steps).

^{1}H NMR (400 MHz, Methanol-d_4) δ 8.12 – 8.03 (m, 2H), 7.68 – 7.44 (m, 5H), 7.38 – 7.17 (m, 8H), 5.39 (d, *J* = 3.6 Hz, 1H), 5.35 – 5.25 (m, 1H), 5.19 (d, *J* = 9.5 Hz, 1H), 5.02 (m, 3H), 4.85 (d, *J* = 7.9 Hz, 1H), 4.66 (d, *J* = 9.5 Hz, 1H), 4.39 – 4.14 (m, 4H), 3.85 (dt, *J* = 10.1, 6.0 Hz, 1H), 3.78 (s, 3H), 3.75 – 3.61 (m, 2H), 3.62 – 3.52 (m, 1H), 3.40 – 3.29 (m, 2H), 3.05 (t, *J* = 6.7 Hz, 2H), 1.66 (m, 2H). ^{19}F NMR (376 MHz, MeOD) δ -192.20. ^{13}C NMR (101 MHz, MeOD) δ 168.76, 165.47, 157.37, 138.28, 136.97, 133.54, 129.48, 129.06, 129.03, 128.45, 128.14, 127.74, 127.63, 127.39, 127.32, 100.27, 100.16, 98.35, 98.30, 95.27, 93.42, 78.13, 75.95, 74.74, 74.58, 72.61, 72.51, 71.89, 71.70, 70.39, 67.56, 65.97, 65.42, 62.16, 52.55, 48.61, 48.40, 48.19, 47.97,

47.76, 47.55, 47.34, 47.12, 37.13, 29.40. HR-ESI-MS *(m/z)*: $[M-2H]^{-2}$ calcd for $C_{31}H_{36}FN_4O_{21}S_3^{3-}$, 470.0900 found, 470.0906.

***N*-benzyloxycarbonyl-3-aminopropyl-*O*-[Methyl(2-azido-2-deoxy-3-*O*-naphthylmethyl-4-*O*-benzyl -6-O-sulfonate-α-D-glucopyranosyl)-(1→4)-*O*-2-*O*-benzoyl-3-deoxy-3-fluoro-β-D-gluco pyranosyluronate](25)**

Disaccharide **22** (208 mg, 0.225 mmol) was subjected to *O*-sulfation and, as described in general procedures, to obtain disaccharide **25** (157 mg, 69.64 %).

^{1}H NMR (600 MHz, Methanol-d_4) δ 8.13 – 7.94 (m, 2H), 7.84 – 7.70 (m, 4H), 7.65 – 7.53 (m, 1H), 7.50 – 7.40 (m, 5H), 7.36 – 7.23 (m, 10H), 5.32 (d, *J* = 3.7 Hz, 2H), 5.01 (d, *J* = 7.2 Hz, 4H), 4.88 (d, *J* = 10.5 Hz, 2H), 4.80 (d, *J* = 10.6 Hz, 1H), 4.42 – 4.30 (m, 2H), 4.27 (dd, *J* = 10.9, 2.0 Hz, 1H), 4.12 (d, *J* = 9.6 Hz, 1H), 3.98 – 3.83 (m, 2H), 3.8(s,3H), 3.79 – 3.62 (m, 2H), 3.57 (dt, *J* = 10.1, 6.0 Hz, 1H), 3.44 (dd, *J* = 10.4, 3.6 Hz, 1H), 3.33 (q, *J* = 1.7 Hz, 1H), 3.09 (ddd, *J* = 29.1, 13.8, 7.1 Hz, 2H), 1.69 (h, *J* = 7.1 Hz, 2H). ^{19}F NMR (564 MHz, Methanol-d_4) δ -187.37. ^{13}C NMR (151 MHz, Methanol-d_4) δ 168.38, 168.37, 165.48, 157.22, 137.96, 136.65, 135.30, 133.58, 133.27, 133.05, 129.69, 128.99, 128.48, 128.30, 128.22, 128.00, 127.97, 127.84, 127.80, 127.63, 127.51, 126.54, 125.94, 125.81, 125.76, 100.39, 100.32, 97.96, 97.92, 94.83, 93.59, 79.34, 77.71, 77.50, 77.28, 75.13, 74.97, 74.78, 74.66, 72.91, 72.86, 71.80, 71.67, 70.68, 67.72, 67.41, 66.60, 66.31, 65.34, 63.17, 52.93, 48.78, 48.76, 48.63, 48.61, 48.49, 48.47, 48.35, 48.33, 48.21, 48.19, 48.07, 48.05, 47.92, 47.90, 37.98, 37.50, 37.36, 29.65, 29.34. HR-ESI-MS *(m/z)*: [M]+ calcd for $C_{49}H_{50}FN_4O_{16}S^-$, 1001.2932; found, 1001.2938.

***N*-benzyloxycarbonyl-3-aminopropyl-*O*-[2-deoxy-2-sulfamino-3-*O*-sulfonate-4-*O*-benzyl-6-*O*-sulfonate-α-D-glucopyranoside-(1→4)-*O*-2-*O*-sulfonate-3-deoxy-3-fluoro-β-D-glucopyranosyluronate](26)**

Disaccharide **23** (123 mg, 0.13 mmol) was subjected to the sequence of Saponification, azide reduction, and *N*-sulfation according to the general procedures to get sulfated disaccharide **26** (75 mg, 58.13 % over three steps).

^{1}H NMR (600 MHz, Deuterium Oxide) δ 8.23 – 6.54 (m, 10H), 5.47 (d, *J* = 3.6 Hz, 1H), 5.02 (s, 2zzH), 4.80 (d, *J* = 10.2 Hz, 1H), 4.64 (t, *J* = 8.6 Hz, 1H), 4.40 (d, *J* = 8.0 Hz, 1H), 4.30 (dd, *J* = 11.1, 2.5 Hz, 1H), 4.17 – 4.08 (m, 1H), 3.94 (dd, *J* = 11.5, 8.8 Hz, 1H), 3.82 (dd, *J* = 10.7, 5.8 Hz, 1H), 3.74 (dd, *J* = 10.3, 3.0 Hz, 1H), 3.66 (s, 1H), 3.62 (t, *J* = 9.7 Hz, 1H), 3.60 – 3.49 (m, 3H), 3.21 (dd, *J* = 10.3, 3.6 Hz, 1H), 3.18 – 3.09 (m, 2H), 3.03 (q, *J* = 7.2 Hz, 1H), 2.92 – 2.84 (m, 1H), 1.71 (p, *J* = 6.6 Hz, 2H). ^{19}F NMR (564 MHz, Deuterium Oxide) δ -189.99. ^{13}C NMR (151 MHz, Deuterium Oxide) δ 158.40, 137.04, 136.59, 129.03, 128.77, 128.71, 128.51, 128.32, 127.58, 101.28, 101.21, 97.49, 96.52, 96.48, 96.28, 77.15, 75.09, 74.78, 73.43, 73.32, 71.52, 71.39, 71.20, 69.08, 67.88, 66.77, 66.12, 57.75, 55.73, 55.45, 49.83, 44.18, 43.29, 37.25, 28.72, 26.84, 20.81, 19.63, 12.27, 10.73. HR-ESI-MS *(m/z)*: $[M-4H]^{-4}$ calcd for $C_{30}H_{35}FN_2O_{25}S_4^{4-}$, 242.5104; found, 242.5110.

***N*-benzyloxycarbonyl-3-aminopropyl-*O*-[2-deoxy-2-sulfamino-3-*O*-sulfonate-4-*O*-benzyl-6-*O*-sulfonate-α-D-glucopyranoside-(1→4)-*O*-3deoxy-3-fluoro-β-D-glucopyranosyluronate](27)**

Disaccharide **24** (140 mg, 0.14 mmol) was subjected to the sequence of Saponification, azide reduction, and *N*-sulfation according to the general procedures to get sulfated disaccharide **27** (60 mg, 45.67 % over three steps).

^{1}H NMR (400 MHz, Deuterium Oxide) δ 7.54 (dd, *J* = 7.9, 1.7 Hz, 2H), 7.49 – 7.37 (m, 8H), 5.60 (d, *J* = 3.5 Hz, 1H), 5.11 (s, 2H), 5.02 (d, *J* = 9.6 Hz, 1H), 4.68 (d, *J* = 9.6 Hz, 1H), 4.53 – 4.31 (m, 3H), 4.19 (dd, *J* = 11.0, 1.9 Hz, 1H), 4.14 – 3.98 (m, 1H), 3.96 – 3.74 (m, 5H), 3.64 (m, 2H), 3.46 (dd, *J* = 10.7, 3.4 Hz, 1H), 3.32 – 3.12 (m, 2H), 1.80 (t, *J* = 6.4 Hz, 2H). ^{19}F NMR (376 MHz, Deuterium Oxide) δ -186.43. ^{13}C NMR (101 MHz, Deuterium Oxide) δ 160.96, 139.52, 139.15, 132.00, 131.33, 131.18, 131.03, 130.89, 130.15, 103.83, 103.71, 100.32, 99.41, 99.35, 98.51, 80.55, 78.11, 77.71, 77.23, 76.30, 76.14, 74.13, 73.95, 71.97, 70.44, 69.33, 68.49, 59.21, 45.82, 39.80, 31.26, 18.67. HR-ESI-MS *(m/z)*: $[M-3H]^{-3}$ calcd for $C_{30}H_{36}FN_2O_{22}S_3^{3-}$, 297.0307; found, 297.0303.

***N*-benzyloxycarbonyl-3-aminopropyl-*O*-(2-deoxy-2-sulfamido-3-*O*-naphthylmethyl-4-*O*-benzyl-6-O-sulfonate-α-D-glucopyranosyl)-(1→4)-*O*-3-deoxy-3-fluoro-β-D-gluco pyranosyluronate(28)**

Disaccharide **25** (115 mg, 0.114 mmol) was subjected to the sequence of Saponification, azide reduction, and *N*-sulfation according to the general procedures to get sulfated disaccharide **28** (42 mg, 39.2 % over three steps).

^{1}H NMR (400 MHz, Deuterium Oxide) δ 7.92 – 7.80 (m, 4H), 7.54 (m, 3H), 7.40 – 7.08 (m, 10H), 5.50 (d, *J* = 3.6 Hz, 1H), 5.12 (d, *J* = 11.1 Hz, 1H), 5.02 (s, 2H), 4.91 – 4.74 (m, 2H), 4.67 – 4.52 (m, 2H), 4.47 – 4.25 (m, 2H), 4.15 (dd, *J* = 10.9, 1.9 Hz, 1H), 4.05 – 3.93 (m, 1H), 3.91 – 3.49 (m, 7H), 3.40 (dd, *J* = 10.3, 3.6 Hz, 1H), 3.15 (q, *J* = 6.4 Hz, 2H), 1.72 (p, *J* = 6.5 Hz, 2H). ^{19}F NMR (376 MHz, Deuterium Oxide) δ -189.06. ^{13}C NMR (101 MHz, Deuterium Oxide) δ 174.20, 158.41, 136.78, 136.60, 135.46, 133.03, 132.88, 128.82, 128.78, 128.62, 128.44, 128.32,

128.06, 127.70, 127.63, 127.60, 127.05, 126.45, 126.43, 101.33, 101.21, 97.88, 96.86, 96.07, 79.73, 76.59, 75.63, 75.27, 75.04, 73.69, 73.52, 71.60, 71.41, 69.34, 67.88, 66.79, 66.06, 57.73, 37.26, 28.75. HR-ESI-MS *(m/z)*: $[M-2H]^{-2}$ calcd for $C_{41}H_{45}FN_2O_{18}S_2^{2-}$, 468.1052; found, 468.1107.

3-aminopropyl-*O*-2-deoxy-2-sulfamino-3-*O*-sulfonate-6-*O*-sulfonate-α-D-glucopyranoside-(1→4)-*O*-3-deoxy-3-fluoro-2-*O*-sulfonate-β-D-glucopyranosyluronate. (HDF-7)

Disaccharide **26** (35mg, 0.036 mmol) was subjected to Global deprotection as described in the General procedure to yield disaccharide **HDF-7** (14 mg, 53.88%).

^{1}H NMR (400 MHz, Deuterium Oxide) δ 5.51 (d, *J* = 3.6 Hz, 1H), 4.50 (d, *J* = 8.1 Hz, 0H), 4.24 (dd, *J* = 11.1, 3.3 Hz, 1H), 4.12 (dd, *J* = 11.0, 2.2 Hz, 1H), 4.03 – 3.89 (m, 1H), 3.84 – 3.75 (m, 1H), 3.72 (d, *J* = 9.2 Hz, 1H), 3.67 – 3.56 (m, 0H), 3.57 – 3.44 (m, 1H), 3.18 (dd, *J* = 9.9, 3.5 Hz, 1H), 3.09 (td, *J* = 6.7, 2.2 Hz, 1H), 1.93 (p, *J* = 6.3 Hz, 1H). ^{19}F NMR (376 MHz, Deuterium Oxide) δ -188.57.^{13}C NMR (101 MHz, Deuterium Oxide) δ 175.24, 101.32, 96.15, 71.53, 71.41, 70.92, 69.98, 69.08, 68.09, 66.40, 57.57, 37.62, 26.64. HR-ESI-MS *(m/z)*: $[M-4H]^{-4}$ calcd for $C_{15}H_{23}FN_2O_{23}S_4^{4-}$, 186.4895; found, 186.4892.

3-aminopropyl-*O*-2-deoxy-2-sulfamino-3-*O*-sulfonate-6-*O*-sulfonate-α-D-glucopyranoside-(1→4)- *O*-3-deoxy-3-fluoro-β-D-glucopyranosyluronate (HDF-6)

Disaccharide **27** (45 mg, 0.05 mmol) was subjected to Global deprotection as described in the General procedure to yield disaccharide **HDF-6** (13 mg, 40.09 %).

^{1}H NMR (600 MHz, Deuterium Oxide) δ 5.53 (d, *J* = 3.4 Hz, 1H), 4.52 (dd, *J* = 8.1, 1.3 Hz, 1H), 4.28 – 4.20 (m, 2H), 4.13 (m, 1H), 4.02 (dddd, *J* = 11.9, 9.8, 8.1, 1.7 Hz, 1H), 3.98 – 3.83 (m, 3H), 3.82 – 3.74 (m, 2H), 3.68 – 3.53 (m, 2H), 3.32 (dt, *J* = 10.8, 2.8 Hz, 1H), 3.08 (m, 2H), 1.98 – 1.85 (m, 2H). ^{19}F NMR (564 MHz, Deuterium Oxide) δ -189.86. ^{13}C NMR (151 MHz, Deuterium Oxide) δ 173.81, 101.32, 101.24, 97.45, 96.62, 96.58, 96.24, 78.31, 73.27, 73.16, 71.50, 71.38, 70.18, 68.11, 68.01, 67.95, 66.32, 66.25, 64.90, 56.21, 37.58, 26.64, 18.43, 15.99, 15.52. HR-ESI-MS *(m/z)*: $[M-3H]^{-3}$ calcd for $C_{15}H_{24}FN_2O_{20}S_3^{3-}$, 222.3362; found, 222.3367.

3-aminopropyl-*O*-2-deoxy-2-sulfamino-6-*O*-sulfonate-α-D-glucopyranoside-(1→4)-*O*-3-deoxy-3-fluoro-β-D-glucopyranosyluronate (HDF-5)

Disaccharide **28** (39 mg, 0.04 mmol) was subjected to Global deprotection as described in the General procedure to yield disaccharide **HDF-5** (11 mg, 45 %).

^{1}H NMR (600 MHz, Deuterium Oxide) δ 5.59 (d, *J* = 3.6 Hz, 1H), 4.86 -4.76 (m, 1H), 4.58 (d, *J* = 8.1 Hz, 1H), 4.32 (dt, *J* = 11.0, 2.8 Hz, 1H), 4.20 (dd, *J* = 11.1, 2.1 Hz, 1H), 4.09 – 3.98 (m, 2H), 3.92 – 3.84 (m, 2H), 3.82 – 3.77 (m, 1H), 3.69 (dt, *J* = 14.9, 8.3 Hz, 1H), 3.58 (tt, *J* = 11.1, 8.4 Hz, 2H), 3.26 (dd, *J* = 9.9, 3.6 Hz, 1H), 3.21 – 3.11 (m, 2H), 2.13 – 1.93 (m, 2H). ^{19}F NMR (564 MHz, Deuterium Oxide) δ -189.60. ^{13}C NMR (151 MHz, Deuterium Oxide) δ 174.42, 101.33, 101.25, 97.67, 96.47, 96.18, 96.14, 74.93, 74.88, 72.77, 72.67, 71.54, 71.42, 70.92, 69.99, 69.09, 68.47, 68.09, 66.39, 57.58, 55.44, 37.61, 26.66, 20.02, 18.43. HR-ESI-MS *(m/z)*: $[M-2H]^{-2}$ calcd for $C_{15}H_{25}FN_2O_{17}S_2^{2-}$, 294.0295; found, 294.0292.

3-aminopropyl-*O*-[(2-acetamido-2-deoxy-*α*-D-glucopyranosyl)]-(1→4)-*O*-β-D-gluco pyranosiduronate (HD-1)

^{1}H NMR (400 MHz, Deuterium Oxide) δ 5.32 (d, J = 3.7 Hz, 1H), 4.38 (d, J = 8.0 Hz, 1H), 3.93 – 3.84 (m, 1H), 3.82 – 3.74 (m, 2H), 3.73 – 3.67 (m, 4H), 3.66 – 3.59 (m, 3H), 3.39 (dd, J = 10.1, 9.1 Hz, 1H), 3.28 – 3.19 (m, 1H), 2.01 – 1.84 (m, 5H). ^{13}C NMR (101 MHz, D_2O) δ 175.2, 174.4, 100.4, 94.4, 73.1, 71.9, 71.1, 69.6, 68.7, 68.4, 67.3, 66.4, 60.1, 53.5, 38.2, 26.4, 21.9. HR-ESI-MS *(m/z)*: $[M]^-$ calcd for $C_{17}H_{30}N_2O_{12}$, 477.1699; found, 477.1632.

3-aminopropyl-*O*-[(2-acetamido-2-deoxy-6-*O*-sulfonate-*α*-D-glucopyranosyl)]-(1→4)-*O*-β-D-glucopyranosiduronate (HD-2)

^{1}H NMR (600 MHz, Deuterium Oxide) δ 5.43 (d, *J* = 3.8 Hz, 1H), 4.48 (dd, *J* = 8.0, 1.3 Hz, 1H), 4.37 – 4.30 (m, 1H), 4.18 (dt, *J* = 11.1, 1.8 Hz, 1H), 4.01 – 3.95 (m, 1H), 3.94 – 3.82 (m, 3H), 3.81 – 3.66 (m, 4H), 3.58 (dd, *J* = 10.4, 9.1, 1.3 Hz, 1H), 3.37 – 3.32 (m, 1H), 3.16 (q, *J* = 6.7 Hz, 2H), 2.05 (s, 3H), 1.99 (p, *J* = 6.4 Hz, 2H). ^{13}C NMR (151 MHz, D_2O) δ 175.0, 174.3, 102.2, 96.8, 76.6, 76.5, 75.5, 73.4, 70.6, 70.0, 69.0, 67.9, 66.3, 53.5, 37.6, 26.7, 21.9. HR-ESI-MS *(m/z)*: $[M]^-$ calcd for $C_{17}H_{29}N_2O_{15}S^-$, 556.1181; found, 556..1136.

3-aminopropyl-*O*-[(2-acetamido-2-deoxy-3-*O*-sulfonate-*α*-D-glucopyranosyl)]-(1→4)-*O*-β-D-glucopyranosiduronate (HD-3)

^{1}H NMR (400 MHz, Deuterium Oxide) δ 5.36 (d, J = 3.6 Hz, 1H), 4.52 – 4.36 (m, 2H), 4.04 (dd, J = 10.6, 3.6 Hz, 1H), 3.94 (dt, J = 11.2, 5.8 Hz, 1H), 3.88 (d, J = 8.4 Hz, 1H), 3.84 – 3.77 (m, 2H), 3.74 (dd, J = 8.0, 5.6 Hz, 3H), 3.64 (t, J = 9.3 Hz, 1H), 3.43 – 3.25 (m, 2H), 3.15 (t, J = 7.2 Hz, 2H), 2.05 – 1.91 (m, 5H). ^{13}C NMR (151 MHz, D_2O) δ 174.8, 174.3, 102.2, 102.2, 97.5, 79.6, 76.9, 76.8, 76.7, 76.2, 76.2, 73.3, 71.8, 68.2, 67.9, 67.7, 59.9, 52.3, 50.8, 42.5, 37.5, 26.7,

26.0, 22.1, 18.1, 18.1. HR-ESI-MS *(m/z)*: $[M]^-$ calcd for $C_{17}H_{29}N_2O_{15}S^-$, 556.1194; found, 556.1146.

3-aminopropyl-*O*-(2-deoxy-2-sulfamido-6-O-sulfonate-α-D-glucopyranosyl)-(1→4)-β-D-gluco pyranosyluronate (HD-4)

^{1}H NMR (600 MHz, Deuterium Oxide) δ 5.56 (d, *J* = 3.9 Hz, 1H), 4.45 (dt, *J* = 8.0, 1.7 Hz, 1H), 4.25 (dt, *J* = 10.7, 3.0 Hz, 1H), 4.09 (dt, *J* = 11.2, 2.5 Hz, 1H), 3.95 – 3.89 (m, 1H), 3.77 (dddd, *J* = 24.4, 16.1, 9.2, 3.0 Hz, 5H), 3.54 – 3.46 (m, 2H), 3.34 – 3.27 (m, 1H), 3.22 – 3.17 (m, 1H), 3.08 (q, *J* = 6.0, 5.3 Hz, 2H), 1.92 (t, *J* = 6.7 Hz, 2H).^{13}C NMR (400 MHz, Deuterium Oxide) δ 174.32, 102.19, 97.37, 76.12, 75.93, 72.64, 71.03, 69.86, 68.98, 67.98, 66.36, 57.82, 37.64, 26.66. HR-ESI-MS *(m/z)*: $[M\text{-}2H]^{2-}$ calcd for $C_{15}H_{26}N_2O_{17}S_2^{2-}$, 285.0342; found, $^-$ 285.0345.

3-aminopropyl-*O*-(2-deoxy-2-sulfamino-3-*O*-sulfonate-α-D-glucopyranoside)-(1→4)-β-D-glucopyranosyluronate (HD-5)

^{1}H NMR (400 MHz, Deuterium Oxide) δ 5.61 (d, *J* = 3.5 Hz, 1H), 4.54 (d, *J* = 8.0 Hz, 1H), 4.38 (dd, *J* = 10.7, 8.9 Hz, 1H), 4.02 (dt, *J* = 11.5, 5.9 Hz, 1H), 3.92 – 3.77 (m, 7H), 3.69 (t, *J* = 9.3 Hz, 1H), 3.43 – 3.36 (m, 2H), 3.18 (t, *J* = 6.9 Hz, 2H), 2.02 (p, *J* = 6.6 Hz, 2H). ^{13}C NMR (400 MHz, Chloroform-*d*) δ 104.73, 100.75, 81.17, 80.52, 79.19, 75.06, 74.27, 70.88, 70.53, 62.46, 59.34, 40.17, 29.19. HR-ESI-MS *(m/z)*: $[M\text{-}2H]^{2-}$, calcd for $C_{15}H_{26}N_2O_{17}S_2^{2-}$ is 285.0342; found, 285.0341.

3-aminopropyl-*O*-2-deoxy-2-sulfamino-3-*O*-sulfonate-6-*O*-sulfonate-α-D-glucopyrano side-(1→4)-β-D-glucopyranosyluronate (HD-6)

^{1}H NMR (400 MHz, Deuterium Oxide) δ 5.54 (d, *J* = 3.6 Hz, 1H), 4.48 (d, *J* = 8.0 Hz, 1H), 4.32 – 4.24 (m, 2H), 4.11 (dd, *J* = 11.1, 2.1 Hz, 1H), 3.97 – 3.87 (m, 2H), 3.86 – 3.72 (m, 4H), 3.67 (t, *J* = 9.6 Hz, 1H), 3.37 – 3.34 (m, 1H), 3.34 – 3.30 (m, 1H), 3.09 (t, *J* = 6.8 Hz, 2H), 1.93 (p, *J* = 6.6 Hz, 2H). ^{13}C NMR (400 MHz, Deuterium Oxide) δ 173.80, 102.22, 98.26, 78.28, 77.76, 75.93, 75.53, 72.47, 70.15, 68.03, 67.83, 66.26, 56.67, 37.64, 26.67. HR-ESI-MS *(m/z)*: $[M-3H]^{3-}$, calcd for $C_{15}H_{25}N_{2}O_{20}S_{3}^{3-}$ is 216.3393; found, 216.3348.

3-aminopropyl-*O*-2-deoxy-2-sulfamino-3-*O*-sulfonate-6-*O*-sulfonate-α-D-glucopyrano side-(1→4)-*O*-2-*O*-sulfonate-β-D-glucopyranosyluronate (HD-7)

^{1}H NMR (600 MHz, Deuterium Oxide) δ 5.68 (d, *J* = 3.5 Hz, 1H), 4.71 (d, *J* = 7.6 Hz, 1H), 4.39 – 4.31 (m, 2H), 4.24 – 4.14 (m, 3H), 4.11 – 4.02 (m, 3H), 3.97 – 3.93 (m, 1H), 3.92 – 3.86 (m, 1H), 3.82 (dt, *J* = 11.1, 5.6 Hz, 1H), 3.75 (dd, *J* = 10.1, 9.0 Hz, 1H), 3.41 (dd, *J* = 10.7, 3.5 Hz, 1H), 3.20 (t, *J* = 6.3 Hz, 3H), 2.01 (q, *J* = 6.1 Hz, 2H). ^{3}C NMR (600 MHz, Deuterium Oxide) δ 173.34, 100.54, 98.15, 79.76, 78.32, 77.03, 75.13, 74.30, 70.23, 68.58, 67.91, 66.28, 56.51, 38.23, 26.34. HR-ESI-MS *(m/z)*: $[M-4H]^{4-}$, calcd for $C_{15}H_{24}N_{2}O_{23}S_{4}^{4-}$ 181.9918; found, 181.9975.

3-aminopropyl-*O*-[(2-acetamido-*α*-D-glucopyranosyl)-(1→4)-*O*-*α*-L-idopyranosid uronate (HD-8)

^{1}H NMR (400 MHz, Deuterium Oxide) δ 5.04 (d, J = 3.7 Hz, 1H), 4.79 (d, J = 2.6 Hz, 1H), 4.40 (d, J = 2.7 Hz, 1H), 3.95 (t, J = 3.1 Hz, 1H), 3.89 (t, J = 4.1 Hz, 1H), 3.81 (td, J = 9.9, 9.1, 2.9 Hz, 2H), 3.72 (s, 2H), 3.69 (dd, J = 2.9, 1.3 Hz, 1H), 3.66 – 3.60 (m, 2H), 3.58 – 3.55 (m, 1H),

3.39 (t, J = 9.3 Hz, 1H), 3.04 (td, J = 6.7, 1.9 Hz, 2H), 1.91 (s, 5H). ^{13}C NMR (101 MHz, D2O) δ 175.2, 174.4, 100.4, 94.4, 73.1, 71.9, 71.1, 69.6, 68.7, 68.4, 67.3, 66.4, 60.1, 53.5, 38.2, 26.4, 21.9. HR-ESI-MS *(m/z)*: $[M]^-$ calcd for $C_{17}H_{30}N_2O_{12}$, 454.1799; found, 454.1788.

3-aminopropyl-*O*-[(2-acetamido-6-*O*-sulfonato-*α*-D-glucopyranosyl)-(1→4)-*O*-*α*-L-idopyranosiduronate (HD-9)

. ^{1}H NMR (400 MHz, Deuterium Oxide) δ 5.06 (d, J = 3.7 Hz, 1H), 4.81 (d, J = 2.6 Hz, 1H), 4.44 (d, J = 2.6 Hz, 1H), 4.25 (dd, J = 11.1, 3.4 Hz, 1H), 4.12 (dd, J = 11.0, 2.1 Hz, 1H), 3.98 (t, J = 2.9 Hz, 1H), 3.92 (t, J = 4.0 Hz, 1H), 3.89 (d, J = 3.5 Hz, 1H), 3.86 (d, J = 3.6 Hz, 1H), 3.84 – 3.78 (m, 1H), 3.66 – 3.60 (m, 2H), 3.58 (dd, J = 4.7, 2.6 Hz, 1H), 3.48 (t, J = 9.6 Hz, 1H), 3.10 – 3.02 (m, 2H), 1.95 – 1.87 (m, 5H). ^{13}C NMR (101 MHz, D_2O) δ 175.0, 174.3, 100.4, 94.3, 72.9, 71.0, 70.1, 69.1, 68.5, 68.2, 67.0, 66.5, 53.3, 38.3, 26.3, 21.9. HR-ESI-MS *(m/z)*: $[M]^-$ calcd for $C_{17}H_{29}N_2O_{15}S^-$, 533.1294; found, 533.1291.

3-aminopropyl-*O*-[(2-acetamido-3-*O*-sulfonato-*α*-D-glucopyranosyl)-(1→4)-*O*-*α*-L-idopyranosiduronate (HD-10)

^{1}H NMR (400 MHz, Deuterium Oxide) δ 5.17 (d, J = 3.7 Hz, 1H), 4.91 (d, J = 2.7 Hz, 1H), 4.51 (d, J = 2.6 Hz, 1H), 4.44 (dd, J = 10.5, 9.0 Hz, 1H), 4.13 – 4.05 (m, 2H), 4.02 (t, J = 4.1 Hz, 1H), 3.95 – 3.89 (m, 1H), 3.89 – 3.82 (m, 3H), 3.73 (dt, J = 10.6, 5.6 Hz, 1H), 3.69 – 3.62 (m, 2H), 3.15 (td, J = 6.7, 1.4 Hz, 2H), 2.00 (s, 5H). ^{13}C NMR (151 MHz, D_2O) δ 175.1, 174.3, 100.4, 94.9, 79.8, 73.7, 71.8, 68.7, 68.3, 68.2, 67.5, 66.4, 59.9, 52.1, 38.1, 26.3, 22.0. HR-ESI-MS *(m/z)*: $[M]^-$ calcd for $C_{17}H_{29}N_2O_{15}S^-$, 533.1294; found, 533.1262.

3-aminopropyl-*O*-[(2-sulfonatomido-6-*O*-sulfonato-*α*-D-glucopyranosyl)-(1→4)-*O*-*α*-L-idopyranosiduronate (HD-11)

. ^{1}H NMR (400 MHz, Deuterium Oxide) δ 5.34 (d, J = 3.7 Hz, 1H), 4.97 – 4.85 (m, 1H), 4.60 (d, J = 2.4 Hz, 1H), 4.34 (dd, J = 11.0, 3.1 Hz, 1H), 4.27 – 4.11 (m, 2H), 4.08 (t, J = 2.8 Hz, 1H), 3.97 – 3.83 (m, 2H), 3.78 – 3.68 (m, 2H), 3.66 – 3.54 (m, 2H), 3.25 (dd, J = 10.0, 3.6 Hz, 1H), 3.21 – 3.11 (m, 2H), 2.04 – 1.98 (m, 2H). ^{13}C NMR (151 MHz, D_2O) δ 174.6, 100.4, 95.8, 74.2, 74.1, 71.1, 70.1, 69.1, 68.1, 68.0, 67.5, 67.5, 66.9, 66.8, 66.6, 66.5, 66.3, 57.6, 50.8, 43.2, 38.3, 26.2, 25.6. HR-ESI-MS (m/z): [M]2^{-} calcd for C15H26N2O17S2-, 285.0684; found, 285.0671.

3-aminopropyl-*O*-[(2-sulfonatomido-3-*O*-sulfonato-*α*-D-glucopyranosyl)-(1→4)-*O*-*α*-L-idopyranosiduronate (HD-12)

^{1}H NMR (600 MHz, Deuterium Oxide) δ 5.33 (d, J = 3.7 Hz, 1H), 4.93 (s, 1H), 4.66 (d, J = 2.5 Hz, 1H), 4.20 (t, J = 3.5 Hz, 1H), 4.07 (d, J = 2.6 Hz, 1H), 3.95 – 3.88 (m, 1H), 3.84 – 3.74 (3, 2H), 3.75 (d, J = 3.7 Hz, 1H), 3.74 – 3.65 (m, 2H), 3.63 – 3.60 (m, 1H), 3.51 (t, J = 9.6 Hz, 1H), 3.24 – 3.12 (m, 3H), 2.03 – 1.96 (m, 2H). ^{13}C NMR (151 MHz, D_2O) δ 174.2, 100.5, 96.1, 74.1, 72.0, 71.2, 69.4, 67.8, 67.2, 66.6, 66.6, 60.0, 57.7, 55.4, 38.3, 26.2. HR-ESI-MS *(m/z)*: $[M]^{2-}$ calcd for $C_{15}H_{26}N_2O_{17}S^{2-}$, 285.0684; found, 285.0673.

3-aminopropyl-*O*-[(2-sulfonatomido-6-*O*-sulfonato-*α*-D-glucopyranosyl)-(1→4)-*O*-2-*O*-sulfonato *O*-*α*-L-idopyranosiduronate (HD-13)

^{1}H NMR (400 MHz, Deuterium Oxide) δ 5.32 (d, J = 3.5 Hz, 1H), 5.08 (d, J = 2.6 Hz, 1H), 4.48 (d, J = 2.7 Hz, 1H), 4.28 (dd, J = 11.0, 2.8 Hz, 1H), 4.24 – 4.11 (m, 3H), 4.03 (t, J = 3.1 Hz, 1H), 3.91 – 3.84 (m, 2H), 3.67 – 3.60 (m, 1H), 3.59 – 3.46 (m, 2H), 3.19 (dd, J = 10.2, 3.6 Hz, 1H), 3.10 (t, J = 6.5 Hz, 2H), 1.97 – 1.90 (m, 2H). ^{13}C NMR (101 MHz, D_2O) δ 176.9, 101.2, 99.7, 78.4, 78.2, 73.5, 72.6, 71.7, 70.9, 70.8, 69.2, 69.0, 60.4, 40.9, 28.7.HR-ESI-MS *(m/z)*: $[M]^{2-}$ calcd for $C_{16}H_{27}N_2O_{20}S_3^{3-}$, 221.0112; found, 221.0195.

3-aminopropyl-*O*-[(2-sulfonatomido-6-*O*-sulfonato-3-*O*-sulfonato-*α*-D-glucopyranosyl)-(1→4)-*O*-2-*O*-sulfonato *O*-*α*-L-idopyranosiduronate (HD-14)

^{1}H NMR (400 MHz, Deuterium Oxide) δ 5.34 (d, J = 3.7 Hz, 1H), 4.97 – 4.85 (m, 1H), 4.60 (d, J = 2.4 Hz, 1H), 4.34 (dd, J = 11.0, 3.1 Hz, 1H), 4.27 – 4.11 (m, 2H), 4.08 (t, J = 2.8 Hz, 1H), 3.97 – 3.83 (m, 2H), 3.78 – 3.68 (m, 2H), 3.66 – 3.54 (m, 2H), 3.25 (dd, J = 10.0, 3.6 Hz, 1H), 3.21 – 3.11 (m, 2H), 2.04 – 1.98 (m, 2H). ^{13}C NMR (151 MHz, D_2O) δ 174.6, 100.4, 95.8, 74.2, 74.1, 71.1, 70.1, 69.1, 68.1, 68.0, 67.5, 67.5, 66.9, 66.8, 66.6, 66.5, 66.3, 57.6, 50.8, 43.2, 38.3, 26.2, 25.6. HR-ESI-MS *(m/z)*: $[M]^{2-}$ calcd for $C_{15}H_{26}N_2O_{17}S^{2-}$, 285.0684; found, 285.0671.

4. NMR Experiments. General Remarks

NMR spectra of compounds HDF-1 – HDF-3 and HDF-5 – HDF-7 were acquired with a Bruker AVANCE 2 600 MHz spectrometer using 5 mm tubes and D_2O as solvent.^{19}F NMR experiments were acquired using a Bruker AVANCE 400 MHz or 600 MHz spectrometer using 5mm tubes and D_2O as solvent. Experiments were acquired using disaccharide solutions at 2 mM concentration at 298K. ^{1}H NMR resonances were assigned through standard TOCSY (30 and 80 ms mixing times) and NOESY (400 and 500 ms mixing times) experiments. ROESY experiments were performed to rule out, if any, slow exchange conformational equilibrium processes.

^{1}H and ^{13}C chemical shifts of disaccharides HDF-1 – HDF-3 and HDF-5 – HDF-7 are provided in tables S1-S6.

Table S1. Chemical shifts (^{1}H and ^{13}C respectively) of **HDF-1**.

**HDF-1**					
GlcNAc		GlcA		Linker	
H1'/C1'	5.12/96.96	H1/C1	4.46/101.41	Ha	3.92,3.77/68.07/68.07
H2'/C2'	3.79/53.68	H2/C2	3.58/71.32	Hb	3.08/37.53
H3'/C3'	3.66/70.34	H3/C3	4.60,4.50/96.54,97.79	Hc	1.92/26.73
H4'/C4'	3.44/69.50	H4/C4	3.90/74.93		
H5'/C5'	3.64/72.00	H5/C5	3.73/74.92		

H6'/C6'	3.72/59.87
NHAc	1.97/21.80

Table S2. Chemical shifts (^{1}H and ^{13}C respectively) of HDF-3.

**HDF-2**					
GlcNAc		GlcA		Linker	
H1'/C1'	5.12/97.03	H1/C1	4.48/101.46	Ha	3.92,3.79/68.15,68.15
H2'/C2'	3.85/53.61	H2/C2	3.61/71.36	Hb	3.09/37.65
H3'/C3'	3.66/70.36	H3/C3	4.61,4.51/96.54,97.74	Hc	1.92/26.83
H4'/C4'	3.51/69.08	H4/C4	3.92/74.92		
H5'/C5'	3.80/70.28	H5/C5	3.74/74.79		
H6'/C6'	4.27,4.09/66.31,66.31				
NHAc	1.98/21.93				

Table S3. Chemical shifts (^{1}H and ^{13}C respectively) of HDF-2.

**HDF-3**					
GlcNAc		GlcA		Linker	
H1'/C1'	5.17/97.13	H1/C1	4.49/101.38	Ha	3.93,3.79/68.02,68.02
H2'/C2'	4.00/52.20	H2/C2	3.60/68.01	Hb	3.09/37.47
H3'/C3'	4.40/79.16	H3/C3	4.68,4.59/96.36,97.58	Hc	1.94/26.65
H4'/C4'	3.59/71.27	H4/C4	3.93/75.25		
H5'/C5'	3.71/71.77	H5/C5	3.76/74.81		
H6'/C6'	3.76/59.69				
NHAc	1.96/21.92				

Table S4. Chemical shifts (^{1}H and ^{13}C respectively) of HDF-5.

**HDF-5**					
GlcNAc		GlcA		Linker	
H1'/C1'	5.52/96.09	H1/C1	4.51/101.24	Ha	3.93,3.80/68.08,68.08
H2'/C2'	3.16/57.61	H2/C2	3.61/71.48	Hb	3.09/37.63
H3'/C3'	3.50/70.93	H3/C3	4.78,4.69/96.45,97.69	Hc	1.93/26.69
H4'/C4'	3.48/69.13	H4/C4	3.97/72.68		
H5'/C5'	3.72/69.99	H5/C5	3.78/74.91		
H6'/C6'	4.24,4.13/66.37,66.37				

Table S5. Chemical shifts (^{1}H and ^{13}C respectively) of HDF-6.

**HDF-6**					
GlcNAc		GlcA		Linker	
H1'/C1'	5.54/96.49	H1/C1	4.52/101.22	Ha	3.93,3.80/68.14,68.14
H2'/C2'	3.32/56.27	H2/C2	3.62/71.44	Hb	3.10/37.63

H3'/C3'	4.25/78.33	H3/C3	4.80,4.71/96.26,97.46	Hc	1.93/26.67
H4'/C4'	3.65/68.05	H4/C4	4.03/73.2		
H5'/C5'	3.78/70.13	H5/C5	3.88/74.58		
H6'/C6'	4.25,4.13/66.31,66.31				

Table S6. Chemical shifts (^{1}H and ^{13}C respectively) of HDF-7 (**6**).

**HDF-7**					
GlcNAc		GlcA		Linker	
H1'/C1'	5.52/96.19	H1/C1	4.51/101.27	Ha	3.93,3.80/68.08,68.08
H2'/C2'	3.62/71.46	H2/C2	3.18/57.64	Hb	3.09/37.61
H3'/C3'	3.50/70.91	H3/C3	4.77,4.68/96.39,97.52	Hc	1.93/26.68
H4'/C4'	3.48/69.13	H4/C4	3.98/72.81		
H5'/C5'	3.70/70.06	H5/C5	3.81/74.70		
H6'/C6'	4.24,4.12/66.36,66.36				

5. Conformational Analysis

Analysis of vicinal coupling constants allowed determining the ring conformation of the individual monosaccharides (see Tables S7-S12). In addition, NOE-derived distances for proton-proton pairs were approximately estimated following the isolated spin pair approximation and compared with those measured in the structures obtained by molecular modelling (Tables S13-S17). This approach was employed to determine the main global conformations of all disaccharides studied. 2D-NOESY spectra of compounds HDF-1 – HDF-3 and HDF-5 – HDF-7 were acquired at 600 MHz (mixing times varying between 400-500 ms). Molecular models were obtained using the carbohydrate building module available in the GLYCAM web portal. The disaccharide structures were then modified using MAESTRO suite of programs to include a fluorine atom at position C3 and to display the corresponding sulfation pattern. Then, they were submitted to an energy minimization process with a low gradient convergence threshold (0.05) in 2500 steps, employing the AMBER force field.

Table 1. Relevant coupling constants of **HDF-1** to determine the ring conformation.

**HDF-1**			
GlcA		GlcNAc	
	Hz		Hz
$^3J_{1,2}$	7,99	$^3J_{1,2}$	3,50
$^3J_{2,3}$	8,97	$^3J_{2,3}$	10,85
$^3J_{3,4}$	8,83	$^3J_{3,4}$	9,73
$^3J_{4,5}$	9,95	$^3J_{4,5}$	9,93
$^3J_{3,F}$	53,18	$^3J_{5,6}$	2,11

Table S8. Relevant coupling constants of **HDF-2** to determine the ring conformation.

**HDF-2**			
GlcA		GlcNAc	
	Hz		Hz
$^3J_{1,2}$	8,04	$^3J_{1,2}$	3,50
$^3J_{2,3}$	8,88	$^3J_{2,3}$	10,68
$^3J_{3,4}$	8,70	$^3J_{3,4}$	9,76
$^3J_{4,5}$	9,94	$^3J_{4,5}$	9,48
$^3J_{3,F}$	53,09	$^3J_{5,6}$	2,35

Table S9. Relevant coupling constants of **HDF-3** to determine the ring conformation.

**HDF-3**			
GlcA		GlcNAc	
	Hz		Hz
$^3J_{1,2}$	8,07	$^3J_{1,2}$	3,74
$^3J_{2,3}$	8,83	$^3J_{2,3}$	10,02
$^3J_{3,4}$	8,80	$^3J_{3,4}$	9,71
$^3J_{4,5}$	10,01	$^3J_{4,5}$	9,49
$^3J_{3,F}$	52,79	$^3J_{5,6}$	2,26

Table S10. Relevant coupling constants of **HDF-5** to determine the ring conformation.

**HDF-5**			
GlcA		GlcNAc	
	Hz		Hz
$^3J_{1,2}$	8,26	$^3J_{1,2}$	3,68
$^3J_{2,3}$	8,85	$^3J_{2,3}$	9,96
$^3J_{3,4}$	8,69	$^3J_{3,4}$	9,73
$^3J_{4,5}$	10,11	$^3J_{4,5}$	9,56
$^3J_{3,F}$	52,82	$^3J_{5,6}$	2,37

Table S11. Relevant coupling constants of HDF-6 to determine the ring conformation.

**HDF-6**			
GlcA		GlcNAc	
	Hz		Hz
$^3J_{1,2}$	8,07	$^3J_{1,2}$	3,43
$^3J_{2,3}$	8,62	$^3J_{2,3}$	10,58
$^3J_{3,4}$	8,53	$^3J_{3,4}$	9,87
$^3J_{4,5}$	10,06	$^3J_{4,5}$	9,52
$^3J_{3,F}$	51,06	$^3J_{5,6}$	2,91

Table S12. Relevant coupling constants of **HDF-7** to determine the ring conformation.

**HDF-7**			
GlcA		GlcNAc	
	Hz		Hz
$^3J_{1,2}$	8,08	$^3J_{1,2}$	3,67
$^3J_{2,3}$	8,75	$^3J_{2,3}$	9,61
$^3J_{3,4}$	8,55	$^3J_{3,4}$	9,72
$^3J_{4,5}$	9,92	$^3J_{4,5}$	9,46

$^{3}J_{3,F}$	53,31	$^{3}J_{5,6}$	2,86

Table S13. Comparison between NOE-derived distances and those measured in the model for HDF-1. Key data for the conformational analysis of the molecule have been highlighted.

**HDF-1**			
	**Rel. NOE cross-peak volume**	**NOE derived distance (Å)**	**Predicted distance (Å)**
H1´-H2´ (ref)	1.00	2.40	2.40
**H1´-H4**	**1.257**	**2.31**	**2.38**
H2´-H4´	0.751	2.52	2.71
H3´-H5´	0.59	2.62	2.73
**H4-H2**	**0.581**	**2.63**	**2.80**
H3-H5	0.997	2.40	2.71
H3-H1	0.513	2.68	2.80
**H1-H5**	**1.957**	**2.15**	**2.56**

Table S14. Comparison between NOE-derived distances and those measured in the model for HDF-2. Key data for the conformational analysis of the molecule have been highlighted.

**HDF-2**			
	**Rel. NOE cross-peak volume**	**NOE derived distance (Å)**	**Predicted distance (Å)**
H1´-H2´ (ref)	1.00	2.40	2.40
**H1´-H4**	**1.283**	**2.30**	**2.38**
H2´-H4´	1.387	2.27	2.71
H3´-H5´	0.269	2.99	2.73
**H4-H2**	**0.496**	**2.70**	**2.80**
H3-H5	1.073	2.37	2.71
H3-H1	0.502	2.69	2.80
**H1-H5**	**2.101**	**2.12**	**2.56**

Table S15. Comparison between NOE-derived distances and those measured in the model for **HDF-3**. Key data for the conformational analysis of the molecule have been highlighted.

**HDF-3**			
	**Rel. NOE cross-peak volume**	**NOE derived distance (Å)**	**Predicted distance (Å)**
H1´-H2´ (ref)	1.00	2.40	2.40
**H1´-H4**	**1.32**	**2.29**	**2.38**
H2´-H4´	1.267	2.31	2.71
H3´-H5´	0.71	2.54	2.73
**H4-H2**	**0.809**	**2.49**	**2.80**
H3-H5	0.289	2.95	2.71
H3-H1	0.416	2.78	2.80
**H1-H5**	**1.944**	**2.15**	**2.56**

Table S14. Comparison between NOE-derived distances and those measured in the model for HDF-2. Key data for the conformational analysis of the molecule have been highlighted.

**HDF-2**			
	**Rel. NOE cross-peak volume**	**NOE derived distance (Å)**	**Predicted distance (Å)**
H1´-H2´ (ref)	1.00	2.40	2.40
**H1´-H4**	**1.283**	**2.30**	**2.38**
H2´-H4´	1.387	2.27	2.71
H3´-H5´	0.269	2.99	2.73
**H4-H2**	**0.496**	**2.70**	**2.80**
H3-H5	1.073	2.37	2.71
H3-H1	0.502	2.69	2.80
**H1-H5**	**2.101**	**2.12**	**2.56**

Table S15. Comparison between NOE-derived distances and those measured in the model for HDF-5. Key data for the conformational analysis of the molecule have been highlighted.

**HDF-5**			
	**Rel. NOE cross-peak volume**	**NOE derived distance (Å)**	**Predicted distance (Å)**
H1´-H2´ (ref.)	1.00	2.40	2.40
**H1´-H4**	**0.774**	**2.50**	**2.38**
H2´-H4´	1.015	2.39	2.71
H3´-H5´	0.354	2.85	2.73
**H4-H2**	**0.703**	**2.55**	**2.80**
H3-H5	0.074	3.70	2.71
H3-H1	---	---	2.80
**H1-H5**	**1.878**	**2.16**	**2.56**

Table S16. Comparison between NOE-derived distances and those measured in the model for **HDF-6**. Key data for the conformational analysis of the molecule have been highlighted.

**HDF-6**			
	**Rel. NOE cross-peak volume**	**NOE derived distance (Å)**	**Predicted distance (Å)**
H1´-H2´ (ref.)	1.00	2.40	2.40
**H1´-H4**	**0.951**	**2.42**	**2.38**
H2´-H4´	1.127	2.35	2.71
H3´-H5´	1.747	2.19	2.73
**H4-H2**	**0.545**	**2.66**	**2.80**
H3-H5	0.032	4.26	2.71
H3-H1	0.213	3.11	2.80
**H1-H5**	**1.029**	**2.39**	**2.56**

Table 2. Comparison between NOE-derived distances and those measured in the model for **HDF-7**. Key data for the conformational analysis of the molecule have been highlighted.

**HDF-7**			
	**Rel. NOE cross-peak volume**	**NOE derived distance (Å)**	**Predicted distance (Å)**
H1´-H2´ (ref.)	1.00	2.40	2.40
**H1´-H4**	**0.61**	**2.61**	**2.38**
H2´-H4´	0.636	2.59	2.71
H3´-H5´	0.467	2.72	2.73
**H4-H2**	**0.358**	**2.85**	**2.80**
H3-H5	---	---	2.71
H3-H1	---	---	2.80
**H1-H5**	**1.298**	**2.30**	**2.56**

6. Molecular modelling of the interaction between HDF-2, HDF-4 and HD-7 with FGF2.

The interaction of the selected disaccharides with the basic fibroblast growth factor (FGF2) was investigated by molecular modelling approaches. To this aim, the X-ray crystallographic structure of the complex between FGF2 and GlcNS(3S,6S)1α→4IdoA(2S) (PDB ID 4OEG) was used as a template. As a first step, the natural disaccharide GlcNS(3S,6S)1α→4GlcA(2S) (**HD-7**) was superimposed in the binding site, employing the most populated conformation found for the free ligand according to MD studies. This initial 3D model was submitted to MD simulations to further analyse the molecular recognition process. The complex between FGF2 and GlcNS(3S,6S)1α→4GlcA(2S) resulted stable along the 100 ns run and the ligand remained in the binding site along the entire trajectory. No changes were detected either in ring puckering or geometries around the glycosidic linkage. Indeed, MD simulations predicted an almost 100% $^4C_1$

pucker for both the GlcNAc and GlcA rings (100% $^4C_1$ and 96.4% $^4C_{1,}$ respectively), as well as the existence of a single conformation around the O-glycosidic linkage, which exhibited rather poor flexibility along the dynamics. The values obtained for ϕ and ψ fluctuate between 0 to -60 and 60 to 120, respectively, and agreed with the conformation predicted for the free ligand (Figure S1A-B).

A

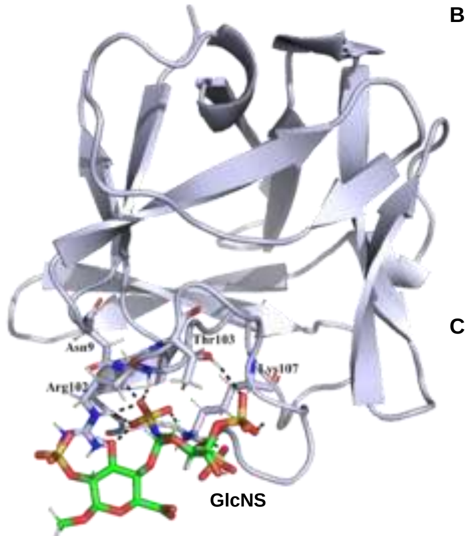

B

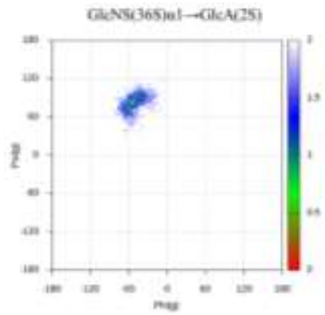

C

Intermolecular hydrogen bond contacts

Acceptor	Donor	Fraction (%)
SO3 (N) (GlcNS)	Lys 107 (HZ)	56
SO3 (N) (GlcNS)	Arg 102 (H)	55
SO3 (N) (GlcNS)	Asn 9 (HD21)	43
SO3 (6) (GlcNS)	Thr 103 (HG1)	31
SO3 (6) (GlcNS)	Arg 102 (HH21)	15
SO3 (N) (GlcNS)	Arg 102 (HH21)	12
Asn 9 (OD1)	OH (3) (GlcA)	10
OH (3) (GlcA)	Lys 117 (HZ)	10
SO3 (3) (GlcNS)	Lys 107 (HZ)	9
COO^- (GlcA)	Arg 102 (HH21)	4

Figure S1. 3D model of the interaction between FGF2 and **HD-7**. A) Representative geometry of the complex obtained by molecular modelling approaches. B) ϕ/ψ plot of the GlcNS(3S,6S)1α→4GlcA(2S) linkage as predicted by MD simulations. C) Intermolecular hydrogen bonds in the complex between FGF2 and HD-7. Atoms are named by using Amber nomenclature. The time-duration of each interaction was calculated as the fraction of the total frames during the MD simulation in which a given intermolecular hydrogen bond was present.

Next, intermolecular hydrogen bond contacts between the ligand and the protein were analysed. The results are gathered in Figure S1C, while Figure S1A shows a single frame of the MD

simulation, where key amino acids interacting with the disaccharide have been highlighted. A set of basic and polar residues, including Asn9, Arg102, Lys107, Gln116, and Lys117 comprised the binding site and create a patch of positive electrostatic potential. Indeed, the interactions of the ligand are mainly sulfate mediated, especially with those groups located at position 6 and N of the GlcNS ring. In contrast, only short-lived interactions were predicted for the GlcA residue, in particular via the hydroxyl group present at position C3.

The complexes between FGF2 and the fluorinated disaccharides **HDF-2** and **HDF-4** were built using similar procedures and the intermolecular interactions established between protein and ligand were analyzed. In particular, the 3D model of the interaction was obtained after energy minimization of the complex with a low gradient convergence threshold (0.05) in 2500 steps employing the AMBER force field and using MAESTRO suite of programs (Figure S2). The incorporation of fluorine at C3 only disrupted the short-lived interactions with Asn9 and Arg102, while the remaining contacts in the FGF2-natural disaccharide complex were preserved. These observations suggested fluorinated and non-fluorinated disaccharides would display similar binding affinities. However, counteracting effects sauch as hydrophobicity could potentially benefit the interaction in the case of fluorinated molecules.

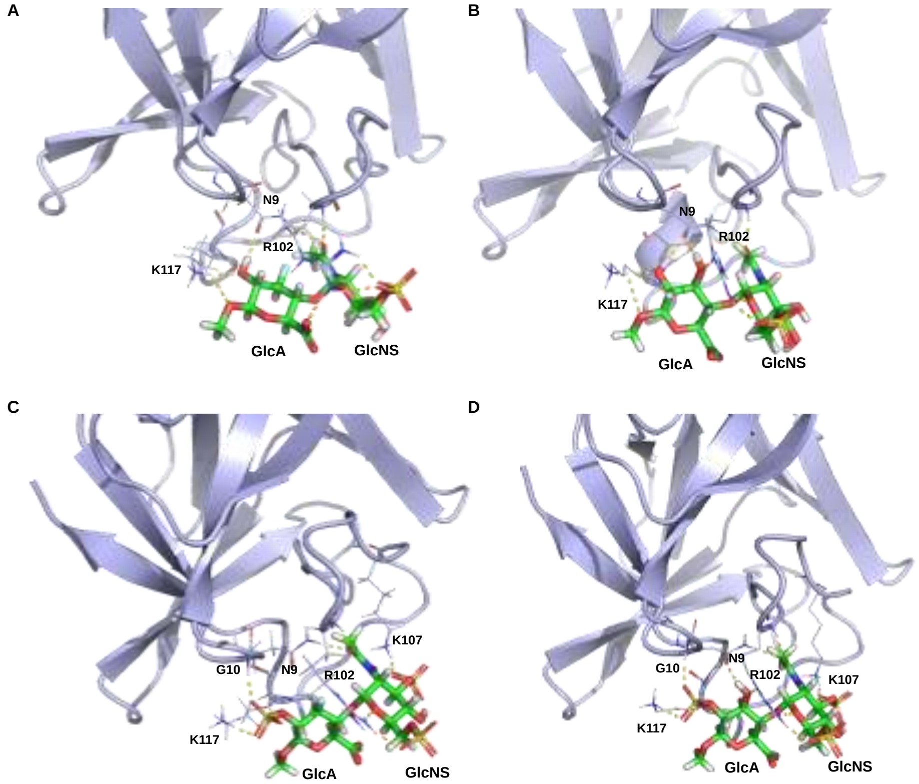

Figure S2. Molecular modelling structures for the interaction between non-fluorinated A) HD-2 and C) HD-4 and fluorinated B) HDF-2 and D) HDF-4 molecules with FGFR2.

7. Surface Plasmon Resonance binding kinetics.

Substrate	K_D(μM)	$K_{on}(M^{-1}S^{-1})$	K_{off} (S^{-1})
**HD-14**	64 ± 0.12	$7.35 \pm 0.15 \times 10^3$	$4.71 \pm 0.13 \times 10^{-1}$
**HDF-2**	72 ± 0.63	$6.16 \pm 0.19 \times 10^3$	$4.41 \pm 0.32 \times 10^{-1}$
**HDF-4**	71 ± 0.61	$6.21 \pm 0.19 \times 10^3$	$4.44 \pm 0.32 \times 10^{-1}$
**HDF-6**	89 ± 0.36	$4.21 \pm 0.19 \times 10^3$	$3.75 \pm 0.17 \times 10^{-1}$

Table S7. SPR analysis of kinetic rate constants and equilibrium affinities for HS ligands to FGF2.

HS disaccharide ligands (0.1 mM) were immobilized onto a CM5 sensor chip via amine coupling. Briefly, the CM5 chip surface was activated using a mixture of N-hydroxysuccinimide (NHS, 0.05 mM) and 1-ethyl-3-(3-dimethylaminopropyl)carbodiimide (EDC, 0.1 mM) in water. Subsequently, 0.1 mM of HS ligands were injected at a flow rate of 100 µL/min until a response of approximately 400 response units (RU) was achieved. Unreacted active esters were then quenched by injecting ethanolamine (0.1 mM) prepared in HBS-EP buffer. A control flow cell was similarly activated with NHS and EDC, followed by blocking with ethanolamine, but without ligand immobilization. To study binding kinetics, fibroblast growth factor 2 (FGF2) at concentrations ranging from 0.1 to 4 µM was injected over the HS-functionalized surface in HBS-EP buffer (10 mM HEPES, 150 mM NaCl, 3 mM EDTA, 0.005% surfactant P20, pH 7.4) at a flow rate of 50 µL/min and a temperature of 25 °C. The association phase was monitored for 300 seconds, followed by dissociation in running buffer (HBS-EP without analyte) for an additional 300 seconds. Regeneration of the sensor surface was carried out using 0.1% SDS and 0.085% phosphoric acid (H_3PO_4), injected at a flow rate of 100 µL/min for 3 minutes. Kinetic parameters were determined using BIAevaluation software (version specific for the Biacore T100 system).

8. 96-well plate HS mimetic immobilization

An epoxy-coated 96-well plate (purchased from **PolyAn**) was functionalized with heparan sulfate (HS) ligands (1 mM in 100 µL of phosphate-buffered saline [PBS], pH 7.5) on a slow-speed plate shaker for 24 hours to allow covalent immobilization. Following incubation, the wells were gently tapped to remove the unbound solution and washed three times with PBS (pH 7.5) to remove non-specifically bound ligands. The functionalized plate was subsequently used for cell proliferation assays and mitogen-activated protein kinase (MAPK) activity studies.

9. Cell proliferation assay

NIH-3T3 cells (approximately 5,000 cells per well) were seeded onto HS ligand-coated 96-well plates in Dulbecco's Modified Eagle Medium (DMEM) supplemented with 1% fetal bovine serum (FBS) and no additional growth factors. After 4 h of incubation, Fibroblast growth factor 2 (FGF2) was added to each well at final concentrations of 10 ng/mL. After 48 hours of incubation at 37 °C in a humidified atmosphere with 5% CO_2, the cells were washed with PBS and fixed with 4% paraformaldehyde. Cell proliferation was assessed using the WST-1 reagent, 2-(4-iodophenyl)-3-(4-nitrophenyl)-5-(2,4-disulfophenyl)-2H-tetrazolium monosodium salt. The absorbance was measured at 450 nm using a microplate reader. The absorbance value from the control group (lacking both HS ligands and FGF2) was set as 100%, and the relative proliferation of other treatment groups was quantified accordingly.

10. MAPK pathway activation assay

NIH-3T3 cells (approximately 5,000 cells per well) were seeded onto HS ligand-coated 96-well plates and incubated for 6 hours at 37 °C to allow cell adhesion. The cells were then stimulated with fibroblast growth factor 2 (FGF2) at a final concentration of 50 ng/mL for 1 hour. Following stimulation, cells were fixed with 4% paraformaldehyde and washed with phosphate-buffered saline (PBS). For detection of MAPK signaling, cells were incubated overnight at 4 °C with either anti-phospho-p44/42 MAPK (ERK1/2) (Thr202/Tyr204) antibody (Cell Signaling Technology) to detect activated pERK1/2, or total p44/42 MAPK (ERK1/2) antibody. After primary antibody incubation, the cells were washed three times with PBS and incubated with Alexa Fluor 555-conjugated anti-mouse IgG Fab fragment secondary antibody. Fluorescence intensity was measured by exciting at 555 nm and recording emission at 565 nm to quantify ERK1/2 activation.

11. Glycan microarray

Materials and Methods

Materials. PBSx10 was purchased from Hy-labs (BP 507/500D), Ethanol Amine from Fisher (FE/0700/08), Ovalbumin (Grade V, A5503) Sodium phosphate monobasic monohydrate

(S9638), Sodium phosphate dibasic heptahydrate (S9390), Tween-20 (D7949) and Tris/HCl (T3253) were purchased from Sigma-Aldrich. Alexa Fluor555 hydrazide, tris(triethylammonium) salt (A20501MP) from Invitrogen. Growth factors, chemokines and their antibodies were purchased from Peprotech: Human FGF1 (100-17A), Human FGF2 (100-18B), Human VEGF-165 (AF-100-20), Human HB-EGF (100-47), Human Amphiregulin (100-55B), Human BMP2 (AF-120-02), Human EGF (AF-100-15), Human MCP-4 CCL13 (300-24-20), Human IP-10 CXCL10 (300-12-5), Human MCP-3 CCL7 (300-17-10), Human MCP-1/MCAF CCL2 (300-04), Human SD1α CXCL12 (300-28A), Human RANTES CCL5 (300-06-5), Human MEC CCL28 (300-57-5), Human Exodus-2 CCL21 (300-35), Human CXCL13 (300-47), Biotinylated-Rabbit αHuman FGF acidic (500-P17BT), Biotinylated-Rabbit-FGF2 polyclonal antibody (500-P18BT), Biotinylated-Rabbit αHuman VEGF-165 (500-P10BT), Biotinylated-Rabbit αHuman HB-EGF (500-P329BT), Biotinylated-Rabbit αHuman Amphiregulin (500-P322BT), Human BMP-2 Detection Antibody (500-P195BT), Biotinylated-Rabbit αHuman EGF (500-P45BT), Biotinylated Anti-H-MCP-4 (500-P04BT), Rabbit Biotinylated Anti-Human IP-10 (500-P93BT), Goat Biotinylated Anti- Human MCP-3 (500-P37Gbt), Rabbit Biotinylated Anti-Human MCAF/MCP-1 (500-P34BT), Biotinilated Antigen Affinity Purified G-Anti Murin SDF-1α (500-P164G), Biotinylated Rabbit Anti-Rantes (CCL5) (500-P36BT), Biotinylated Anti-Human MEC (500-P297Bt), Biotinylated-Rabbit αHuman Exodus-2 CCL21 (500-P109BT). ChromPure Human IgG, whole molecule (009-000-003), Cy3-Sterptavidin (016-160-0848) and Alexa Fluor 647 Donkey Anti-Rabbit IgG (H+L) (711-605-152) were purchased from Jackson ImmunoResearch.

HS mimetics glycan microarray fabrication. Nano-printing of HS mimetics glycan microarray were fabricated as previously described.[2] Array (Version VRH2) were fabricated with NanoPrint

LM-60 Microarray Printer (Arrayit) on epoxide-derivatized slides (PolyAn 2D) with 16 sub-array blocks on each slide, each sub-array containing 380 spots arraigned in 20×19 matrix. Glycoconjugates were distributed into 384-well source plates using 4 replicate wells per sample and 8 µl per well. Each glycoconjugate diluted into 100 µM and 50 µM in an optimized printing buffer (300 mM phosphate buffer, pH 8.4). To monitor printing, ChromPure Human IgG whole molecule (at 20 ng/µl in PBS pH 7.4 + 10% glycerol) and AlexaFlour-555-Hydraside (at 2 ng/µl in 178 mM phosphate buffer, pH 5.5) were used for each sub-array. The arrays were printed with four 946MP3 pins (5 µm tip, 0.25 µl sample channel, ~100 µm spot diameter; Arrayit). The humidity level in the arraying chamber was maintained at about 70% during printing. Printed slides were left on arrayer deck over-night, allowing humidity to drop to ambient levels (40–45%). Next, slides were packed, vacuum-sealed and stored at room temperature (RT) until used.

HS mimetics glycan microarray binding assay. Slides were developed and analyzed as previously described[2b] with some modifications. Slides were rehydrated with dH_2O and incubated for 30 min in a staining dish with 50°C pre-warmed ethanolamine (0.05 M) in Tris-HCl (0.1 M, pH 9.0), then washed with 50°C pre-warmed dH_2O. Slides were centrifuged at 200×g for five min then fitted with ProPlate™ Multi-Array 16-well slide module (Grace Bio-lab) to divide into the sub-arrays (blocks). Slides were washed with PBST (0.1% Tween 20), aspirated and blocked with 200 µl/sub-array of blocking buffer (PBS pH 7.3 + 1% w/v ovalbumin) for 1 hour at RT with gentle shaking. Next, the blocking solution was aspirated and each protein was applied into 3 separate sub-arrays, each at different concentration- 10, 3.3 or 1.3 ng/µl in 100 µl / sub-array (all but CXCL13 which was used at 3.7, 1.2 and 0.49 ng/µl), diluted in blocking buffer, and incubated with gentle shaking for 2 hours at RT. Binding was

detected with biotinylated secondary detection (1 ng/µl, 100 µl/sub-array) diluted in PBS for 1 hour at RT with gentle shaking, followed by Cy3 or Alexa 647 labeled final (as listed in table S18) detection diluted in PBS at 200 µl/sub-array, for 1 hour at RT with gentle shaking. Slides were washed 4 times with PBST, then with PBS (without Tween-20) for 10 min followed by removal from ProPlate™ Multi-Array slide module and immediately dipping in a staining dish with dH_2O for 10 min with shaking. Slide then were centrifuged at 200×g for 5 minutes and the dry slides immediately scanned.

Array slide processing. Processed slides were scanned and analyzed at 10 µm resolution with a Genepix 4000B microarray scanner (Molecular Devices) using 350 PMT gain (for Cy3) or 600 PMT gain (for Alexa 647). Image analysis was carried out with Genepix Pro 7.0 analysis software (Molecular Devices). Spots were defined as circular features with a variable radius as determined by the Genepix scanning software. Local background subtraction was performed, and relative fluorescence units (RFU) were calculated for each spot. Next, the maximum RFU in each sub-array was used as 100% (maximum between both 50 and 100µM in the same sub-array) where all other RFU's from this sub-array were ranked according to the maximum. For each protein tested, rank results for each glycan were averaged (n=6, 2 different glycan concentration printed, and 3 different protein concentration used) and SEM was calculated (Supplementary data array file).

Table S18. Microarray detection details

Protein	Catalog number	Concentration used µg/ml	2nd detecting Antibody, 1µg/ml	Catalog number	Final detection	Cat No
Human FGF1 (acidic)	100-17A Pepeotech	10, 3.3, 1.3	Biotinylated-Rabbit αHuman FGF acidic	500-P17BT Peprotech	Cy3-SA, 1.5µg/ml	Jackson Immunoresearch 016-160-0848
Human FGF2 (basic recombinant)	100-18B Pepeotech	10, 3.3, 1.3	Biotinylated-Rabbit-FGF2 polyclonal antibody	500-P18BT Peprotech	Cy3-SA, 1.5µg/ml	Jackson Immunoresearch 016-160-0848
Human VEGF-165	AF-100-20 Pepeotech	10, 3.3, 1.3	Biotinylated-Rabbit αHuman VEGF-165	500-P10BT Peprotech	Cy3-SA, 1.5µg/ml	Jackson Immunoresearch 016-160-0848
Human HB-EGF	100-47 Pepeotech	10, 3.3, 1.3	Biotinylated-Rabbit αHuman HB-EGF	500-P329BT Peprotech	Cy3-SA, 1.5µg/ml	Jackson Immunoresearch 016-160-0848
Human Amphiregulin	100-55B Pepeotech	10, 3.3, 1.3	Biotinylated-Rabbit αHuman Amphiregulin	500-P322BT Peprotech	Cy3-SA, 1.5µg/ml	Jackson Immunoresearch 016-160-0848
Human BMP2	AF-120-02 Pepeotech	10, 3.3, 1.3	Human BMP-2 Detection Antibody	500-P195BT Peprotech	Alexa 647 anti Rabbit IgG (H+L), 5µg/ml	Jackson Immunoresearch 711-605-152
Human EGF	AF-100-15 Pepeotech	10, 3.3, 1.3	Biotinylated-Rabbit αHuman EGF	500-P45BT Peprotech	Cy3-SA, 1.5µg/ml	Jackson Immunoresearch 016-160-0848
Human MCP-4 (CCL13)	300-24-20 Pepeotech	10, 3.3, 1.3	Biotinylated Anti-H-MCP-4	500-P04BT-25	Cy3-SA, 1.5µg/ml	Jackson Immunoresearch 016-160-0848
Human IP-10 (CXCL10)	300-12-5 Pepeotech	10, 3.3, 1.3	Rabbit Biotinylated Anti-Human IP-10	500-P93BT-25	Cy3-SA, 1.5µg/ml	Jackson Immunoresearch 016-160-0848
Human MCP-3 (CCL7)	300-17-10 Pepeotech	10, 3.3, 1.3	Goat Biotinylated Anti-Human MCP-3 (CCL7)	500-P37Gbt-25 Peprotech	Cy3-SA, 1.5µg/ml	Jackson Immunoresearch 016-160-0848
Human MCP-1/MCAF (CCL2)	300-04 Pepeotech	10, 3.3, 1.3	Rabbit Biotinylated Anti-Human MCAF/MCP-1 (CCL2)	500-P34BT Peprotech	Cy3-SA, 1.5µg/ml	Jackson Immunoresearch 016-160-0848
Human SD1α (CXCL12)	300-28A Pepeotech	10, 3.3, 1.3	Biotinilated Antigen Affinity Purified G-Anti Murin SDF-1α	500-P164G Peprotech	Cy3-SA, 1.5µg/ml	Jackson Immunoresearch 016-160-0848
Human RANTES (CCL5)	300-06-5 Pepeotech	10, 3.3, 1.3	Biotinylated Rabbit Anti-Rantes (CCL5)	500-P56BT Peprotech	Cy3-SA, 1.5µg/ml	Jackson Immunoresearch 016-160-0848
Human MEC (CCL28)	300-57-5 Pepeotech	10, 3.3, 1.3	Biotinylated Anti-Human MEC	500-P297Bt-25	Cy3-SA, 1.5µg/ml	Jackson Immunoresearch 016-160-0848
Human Exodus-2 (CCL21)	300-35 Pepeotech	10, 3.3, 1.3	Biotinylated-Rabbit αHuman Exodus-2 (CCL21)	500-P109BT Peprotech	Cy3-SA, 1.5µg/ml	Jackson Immunoresearch 016-160-0848
Human BCA-1 (CXCL13)	300-47-5 Pepeotech	3.7, 1.2, 0.49	Rabbit Biotinylated Anti-Human BCA-1	500-P141BT-25	Cy3-SA, 0.1µg/ml	Jackson Immunoresearch 016-160-0848

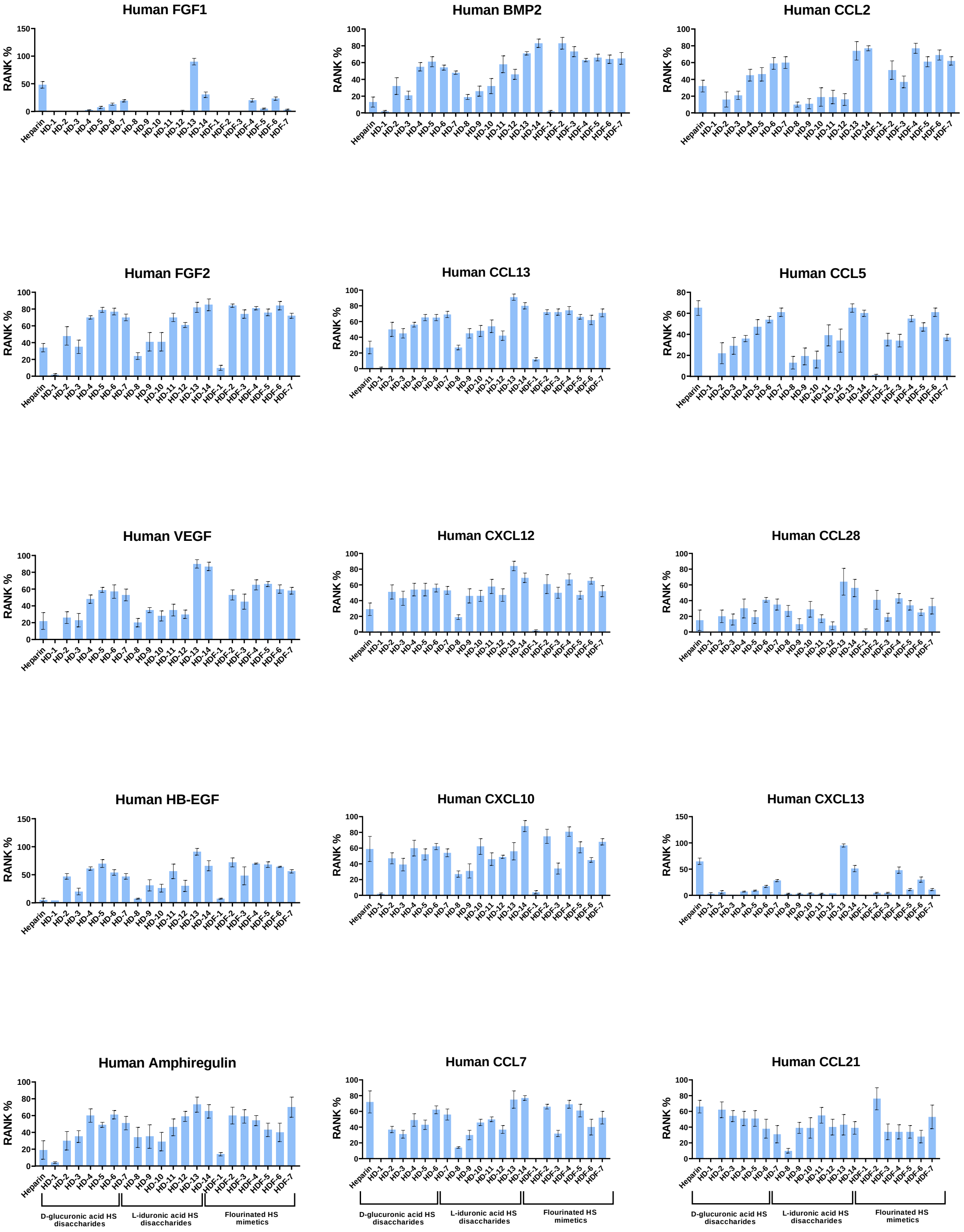

Figure S3. Microarray analysis of signaling proteins with **HD-1** to **HD-14** and **HDF-1** to **HDF-7**.

12. References.

1. (a) Anand, S.; Bhoge, P. R.; Raigawali, R.; Saladi, S. V.; Kikkeri, R. *Chem. Sci*. **2024**, 15, 19962-19969; (b) Jain. P.; Shanthamurthy, C. D.; Chaudhary, P. M.; Kikkeri, R. *Chem. Sci*. **2021**, 12, 4021-4027; (c) Jain. P.; Shanthamurthy, C. D.; Leviatan Ben-Arye, S.; Woods, R. J.; Kikkeri, R.; Padler-Karavani, V. *Chem. Sci*. **2021**, 12, 3674-2681; (d) Jain, P.; Shanthamurthy, C. D.; Leviatan Ben-Arye, S.; Yehuda, S.; Nandikol, S. S.; Thulasiram, H. V.; Palder-karavani, V.; Kikkeri. R. *Chem. Commun*. **2021**, 57, 3516-3519; (e) Anand, S.; Mardhekar, S.; Raigawali, R.; Mohanta, N.; Jain, P. D. Shanthamurthy, C.; Gnanaprakasam, B.; Kikkeri. R. *Org. Lett*. **2020**, 22, 3402-3406.

2. (a) Leviatan Ben-Arye, S.; Yu, H.; Chen, X.; Padler-Karavani, V.; *J. Vis. Chem*. **2017**, 125, 56094; (b) Padler-Karavani, V.; Sung, X.; Yu, H.; Hurtado-Ziola, N.; Huang, S.; Muthana, S.; Chokhawala, H. A.; Cheng, J.; Verhagan, A.; Langereis, M. A.; Kleene, R.; Schachner, M.; de Groot, R. J.; Lasanajak, Y.; Matsuda, H.; Schwad, R.; Chen, X.; Smith, D. F.; Cummings, R. D.; Vark, A. *J. Bio. Chem.* **2012**, 287, 22593-22608.

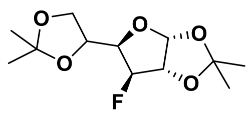
O
O
O
O
O
F

3

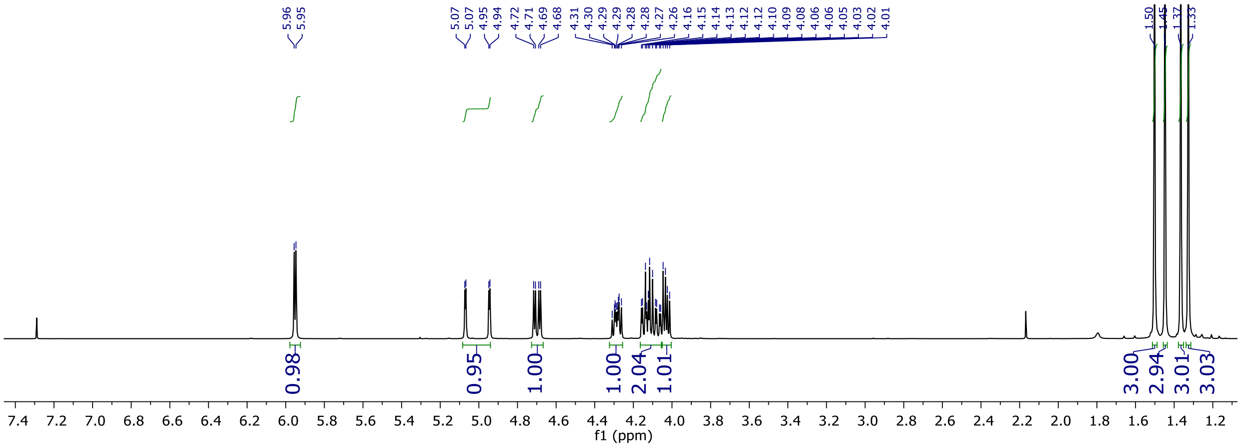
5.96
5.95
5.07
5.07
4.95
4.94
4.72
4.71
4.69
4.68
4.31
4.30
4.29
4.29
4.28
4.28
4.27
4.26
4.16
4.15
4.14
4.13
4.12
4.12
4.10
4.09
4.08
4.06
4.06
4.05
4.03
4.02
4.01
1.50
1.45
1.37
1.33
0.98
0.95
1.00
1.00
2.04
1.01
3.00
2.94
3.01
3.03
7.4 7.2 7.0 6.8 6.6 6.4 6.2 6.0 5.8 5.6 5.4 5.2 5.0 4.8 4.6 4.4 4.2 4.0 3.8 3.6 3.4 3.2 3.0 2.8 2.6 2.4 2.2 2.0 1.8 1.6 1.4 1.2
f1 (ppm)

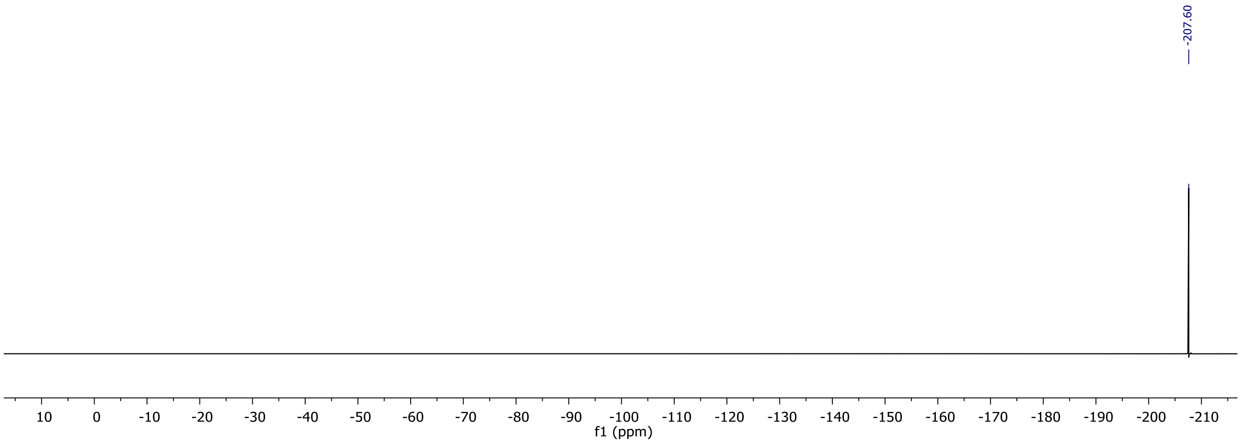
-207.60
10 0 -10 -20 -30 -40 -50 -60 -70 -80 -90 -100 -110 -120 -130 -140 -150 -160 -170 -180 -190 -200 -210
f1 (ppm)

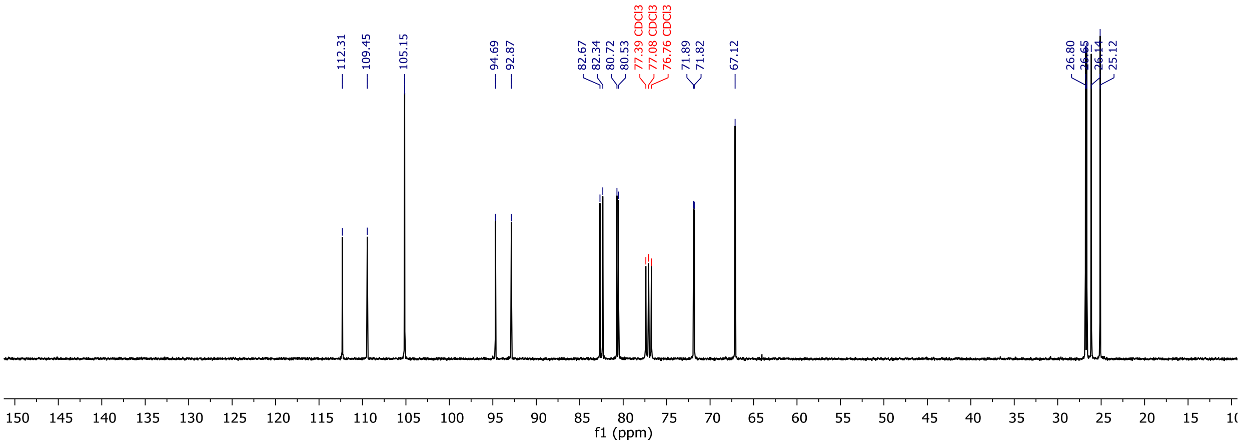

112.31
109.45
105.15
94.69
92.87
82.67
82.34
80.72
80.53
77.39 CDCl3
77.08 CDCl3
76.76 CDCl3
71.89
71.82
67.12
26.80
26.65
26.14
25.12
f1 (ppm)

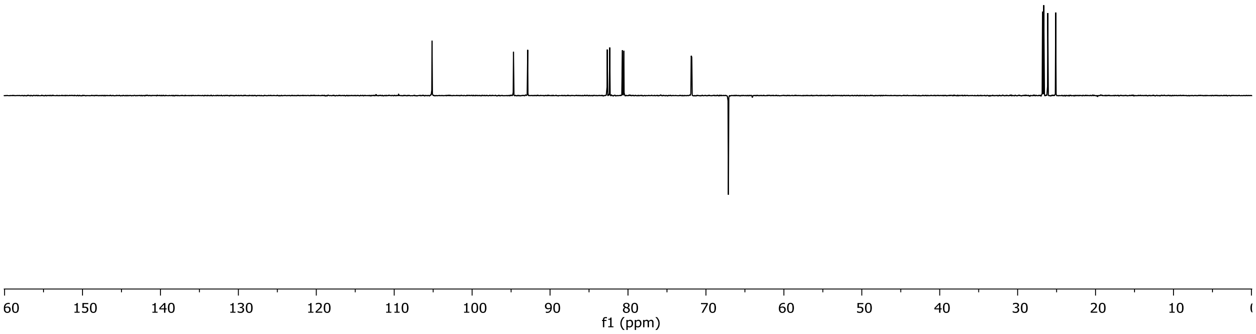

f1 (ppm)

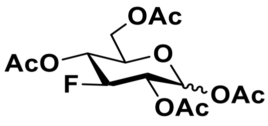
OAc
AcO
O
F
OAc
OAc

5

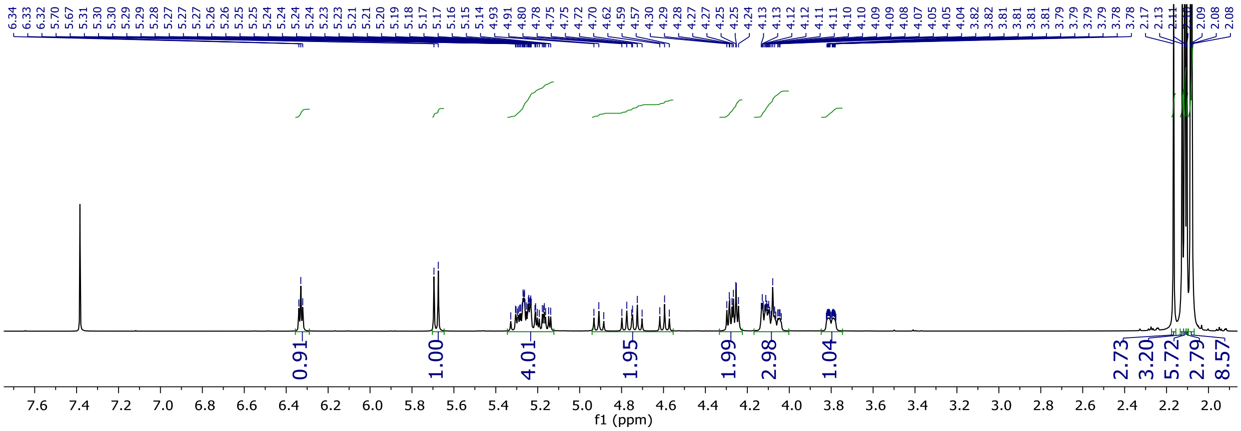
0.91
1.00
4.01
1.95
1.99
2.98
1.04
2.73
3.20
5.72
2.79
8.57
f1 (ppm)

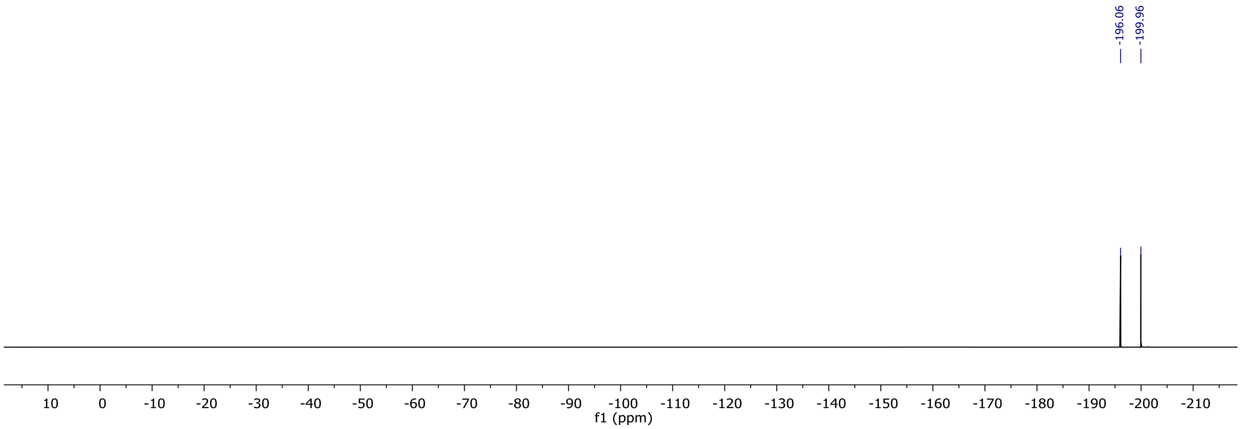
-196.06
-199.96
f1 (ppm)

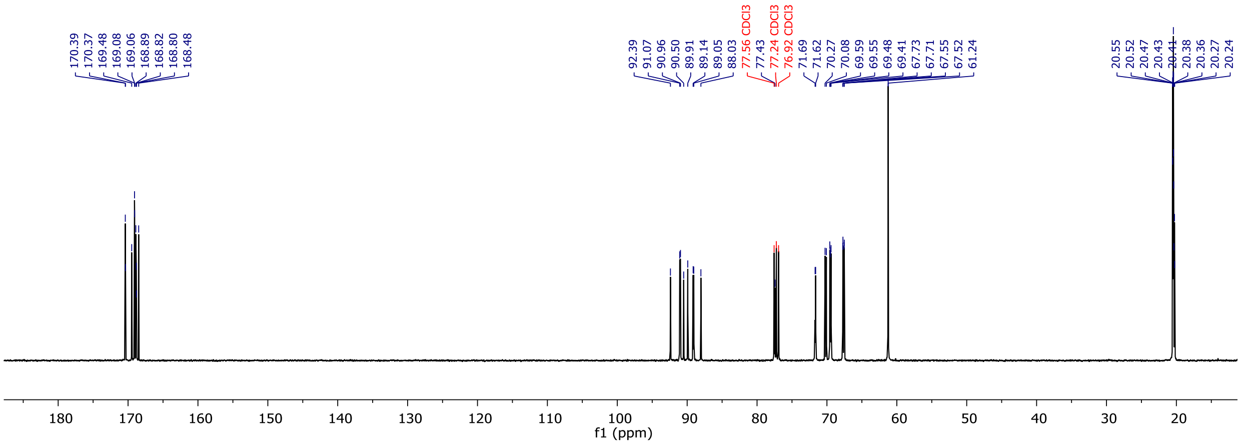

170.39
170.37
169.48
169.08
169.06
168.89
168.82
168.80
168.48
92.39
91.07
90.96
90.50
89.91
89.14
89.05
88.03
77.56 CDCl3
77.43
77.24 CDCl3
76.92 CDCl3
71.69
71.62
70.27
70.08
69.59
69.55
69.48
69.41
67.73
67.71
67.55
67.52
61.24
20.55
20.52
20.47
20.43
20.41
20.38
20.36
20.27
20.24
f1 (ppm)

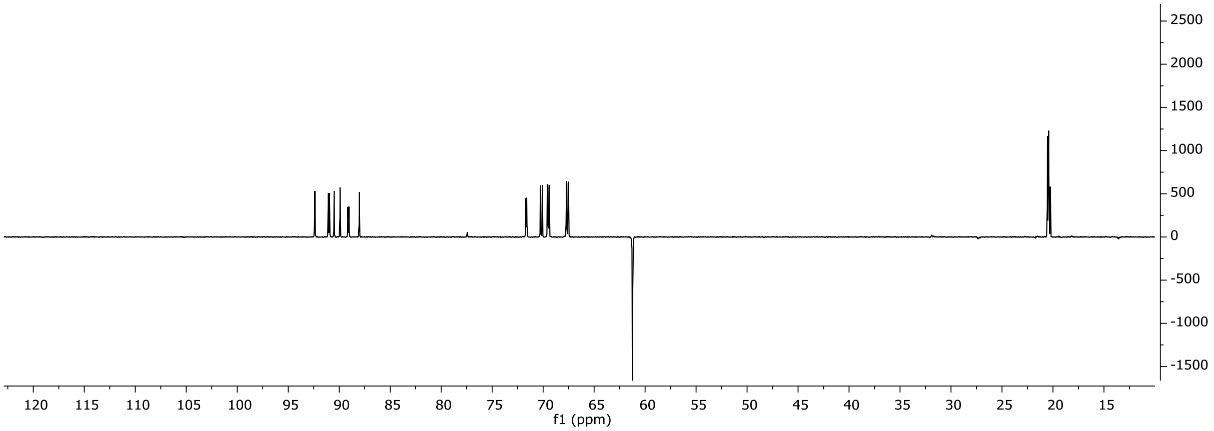

f1 (ppm)

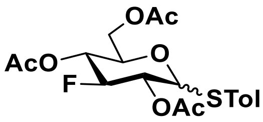
OAc
AcO
O
F
OAc
STol

6

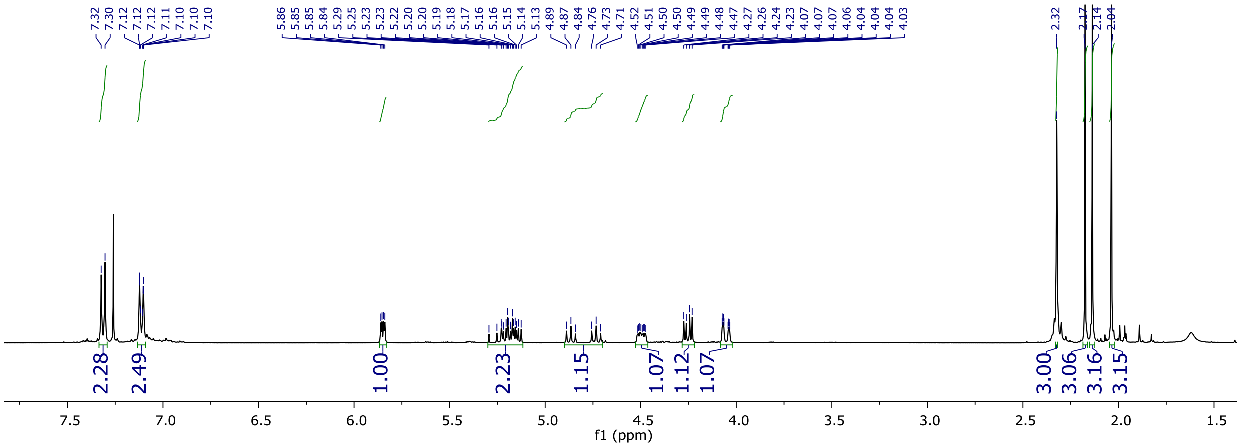
2.28
2.49
1.00
2.23
1.15
1.07
1.12
1.07
3.00
3.06
3.16
3.15
f1 (ppm)

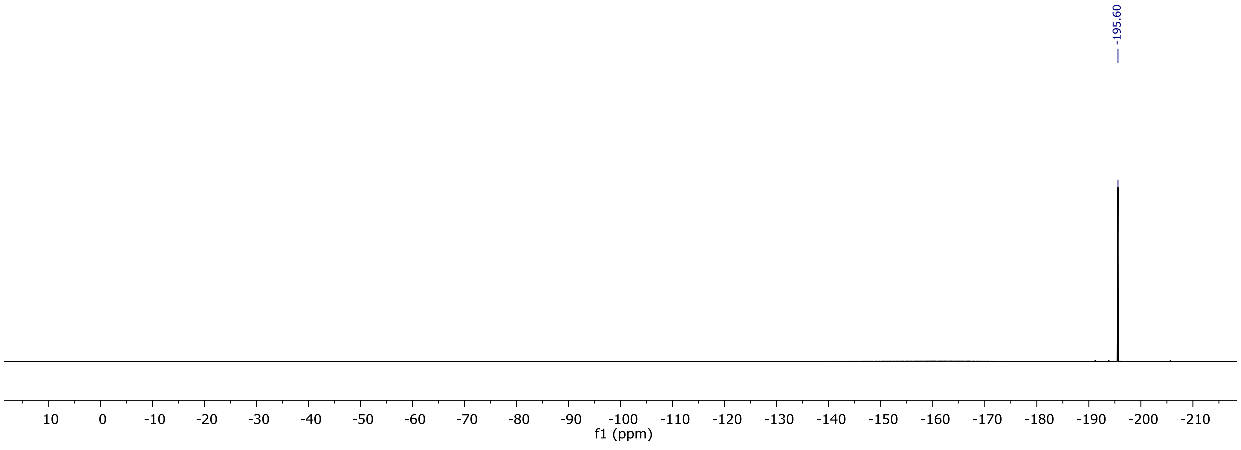
-195.60
f1 (ppm)

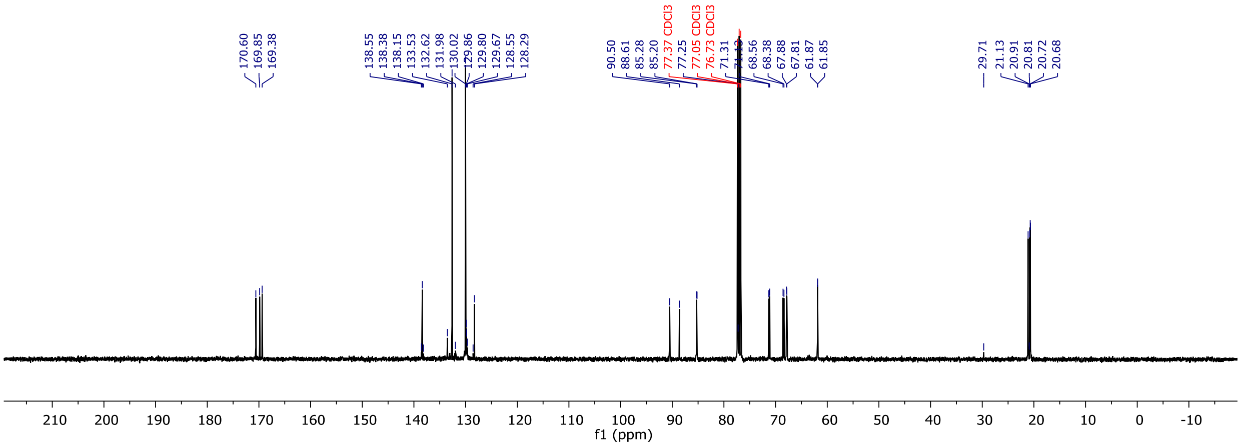
170.60
169.85
169.38
138.55
138.38
138.15
133.53
132.62
131.98
130.02
129.86
129.80
129.67
128.55
128.29
90.50
88.61
85.28
85.20
77.37 CDCl3
77.25
77.05 CDCl3
76.73 CDCl3
71.31
68.56
68.38
67.88
67.81
61.87
61.85
29.71
21.13
20.91
20.81
20.72
20.68
210 200 190 180 170 160 150 140 130 120 110 100 90 80 70 60 50 40 30 20 10 0 -10
f1 (ppm)

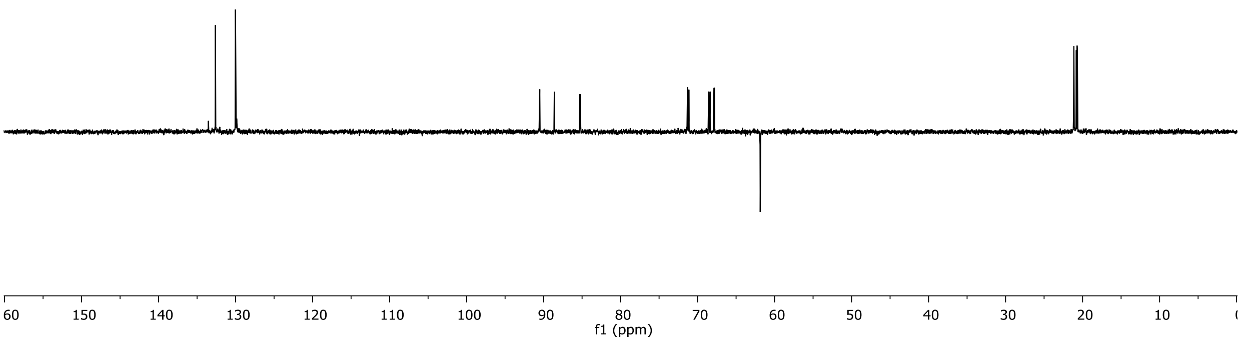
150 140 130 120 110 100 90 80 70 60 50 40 30 20 10
f1 (ppm)

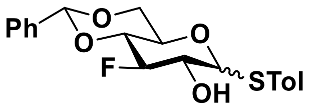
Ph
O
O
O
F
OH
STol

8

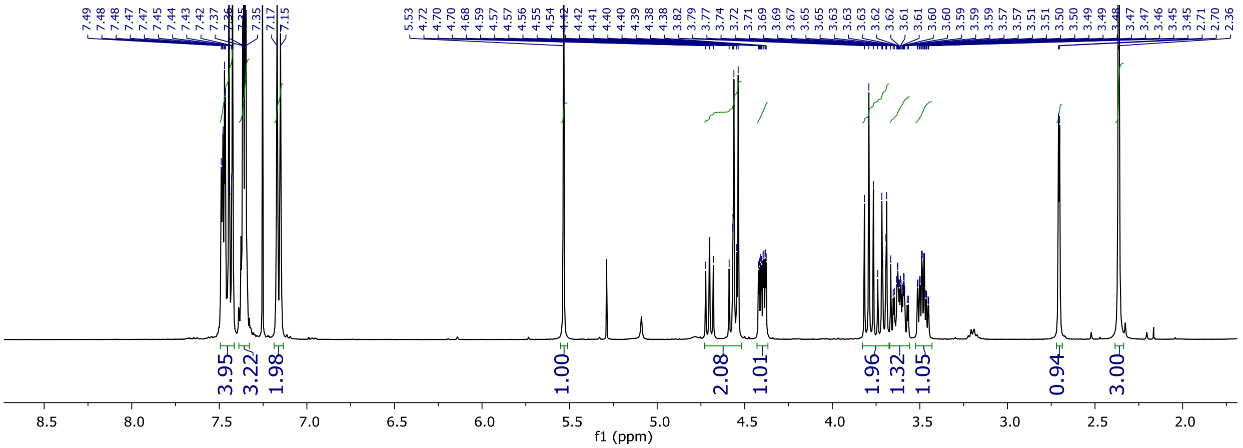
3.95
3.22
1.98
1.00
2.08
1.01
1.96
1.32
1.05
0.94
3.00
8.5
8.0
7.5
7.0
6.5
6.0
5.5
5.0
4.5
4.0
3.5
3.0
2.5
2.0
f1 (ppm)

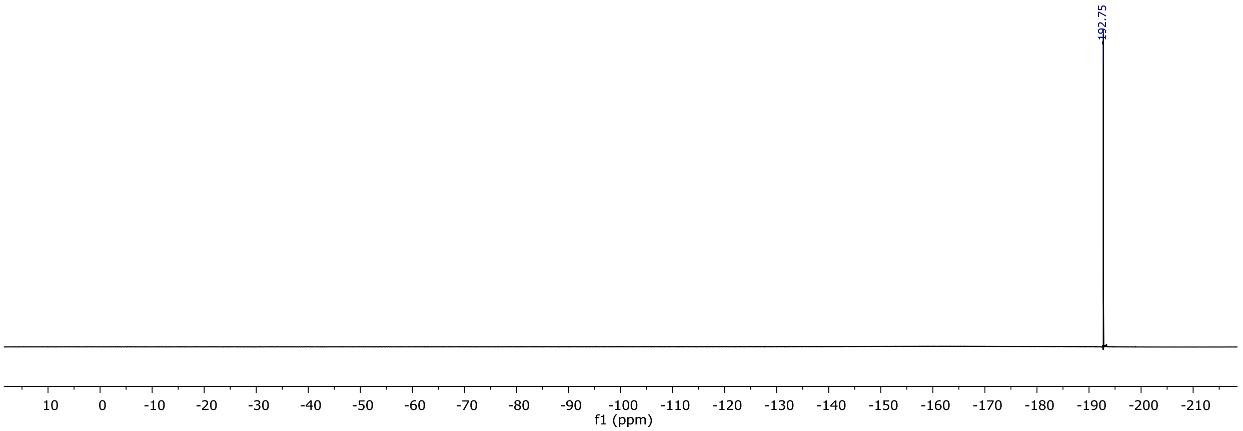
-192.75
10
0
-10
-20
-30
-40
-50
-60
-70
-80
-90
-100
-110
-120
-130
-140
-150
-160
-170
-180
-190
-200
-210
f1 (ppm)

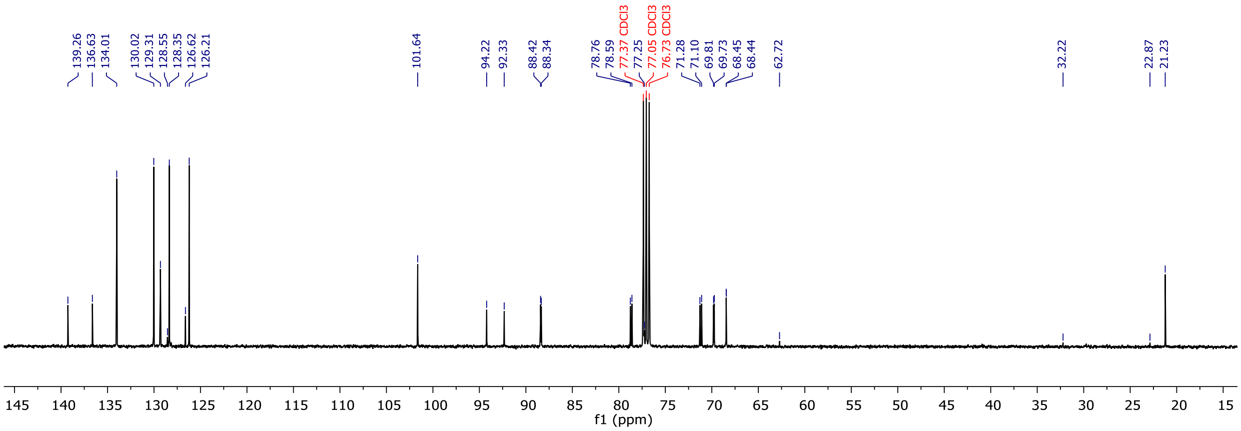

139.26
136.63
134.01
130.02
129.31
128.55
128.35
126.62
126.21
101.64
94.22
92.33
88.42
88.34
78.76
78.59
77.37 CDCl3
77.25
77.05 CDCl3
76.73 CDCl3
71.28
71.10
69.81
69.73
68.45
68.44
62.72
32.22
22.87
21.23
f1 (ppm)

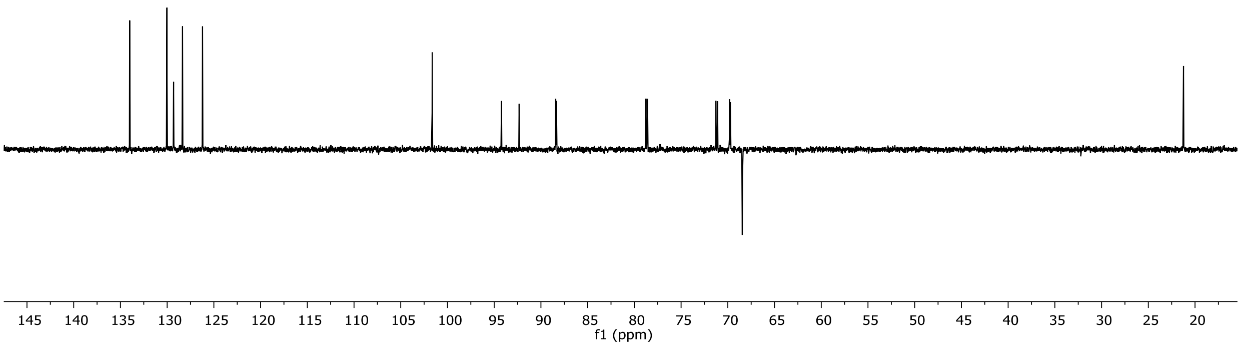

f1 (ppm)

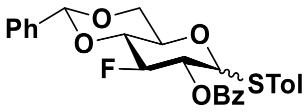
Ph
O
O
O
F
OBz
STol

9

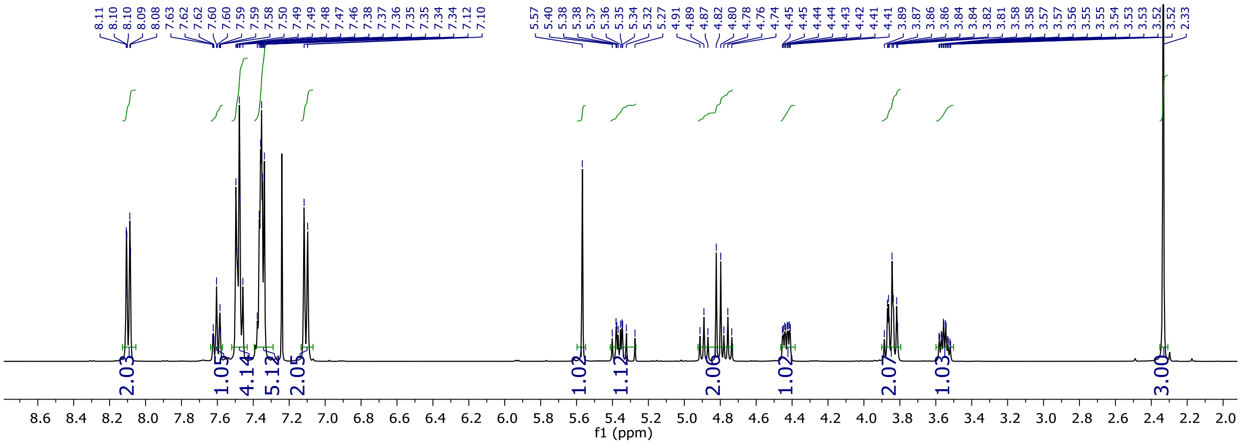
8.11
8.10
8.10
8.09
8.08
7.63
7.62
7.62
7.60
7.59
7.59
7.58
7.50
7.49
7.49
7.48
7.47
7.46
7.38
7.37
7.36
7.35
7.35
7.34
7.34
7.12
7.10
5.57
5.40
5.38
5.38
5.37
5.36
5.35
5.34
5.32
5.27
4.91
4.89
4.87
4.82
4.80
4.78
4.76
4.74
4.45
4.45
4.44
4.44
4.43
4.42
4.41
4.41
3.89
3.87
3.86
3.86
3.84
3.84
3.82
3.81
3.58
3.58
3.58
3.57
3.57
3.56
3.55
3.55
3.54
3.53
3.53
3.52
3.52
2.33
2.03
1.05
4.14
5.12
2.05
1.02
1.12
2.06
1.02
2.07
1.03
3.00
8.6 8.4 8.2 8.0 7.8 7.6 7.4 7.2 7.0 6.8 6.6 6.4 6.2 6.0 5.8 5.6 5.4 5.2 5.0 4.8 4.6 4.4 4.2 4.0 3.8 3.6 3.4 3.2 3.0 2.8 2.6 2.4 2.2 2.0
f1 (ppm)

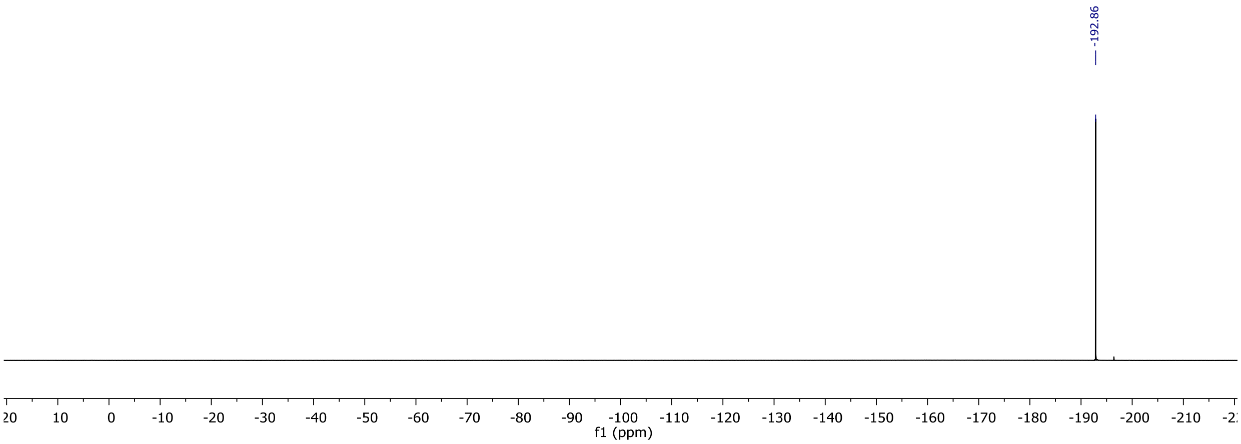
-192.86
10 0 -10 -20 -30 -40 -50 -60 -70 -80 -90 -100 -110 -120 -130 -140 -150 -160 -170 -180 -190 -200 -210
f1 (ppm)

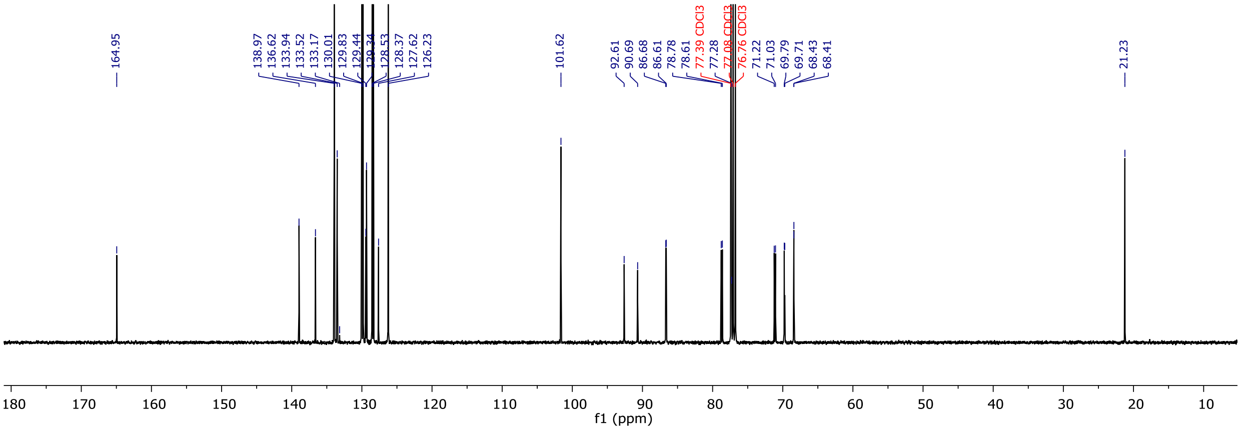
164.95
138.97
136.62
133.94
133.52
133.17
130.01
129.83
129.44
129.34
128.53
128.37
127.62
126.23
101.62
92.61
90.69
86.68
86.61
78.78
78.61
77.39 CDCl3
77.28
77.08 CDCl3
76.76 CDCl3
71.22
71.03
69.79
69.71
68.43
68.41
21.23
180 170 160 150 140 130 120 110 100 90 80 70 60 50 40 30 20 10
f1 (ppm)

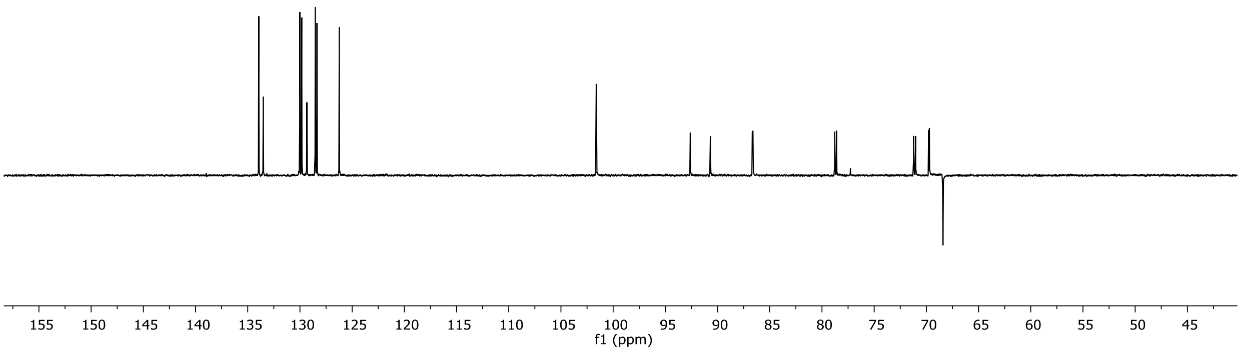
155 150 145 140 135 130 125 120 115 110 105 100 95 90 85 80 75 70 65 60 55 50 45
f1 (ppm)

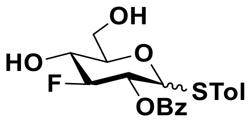
OH
O
HO
F
STol
OBz

10

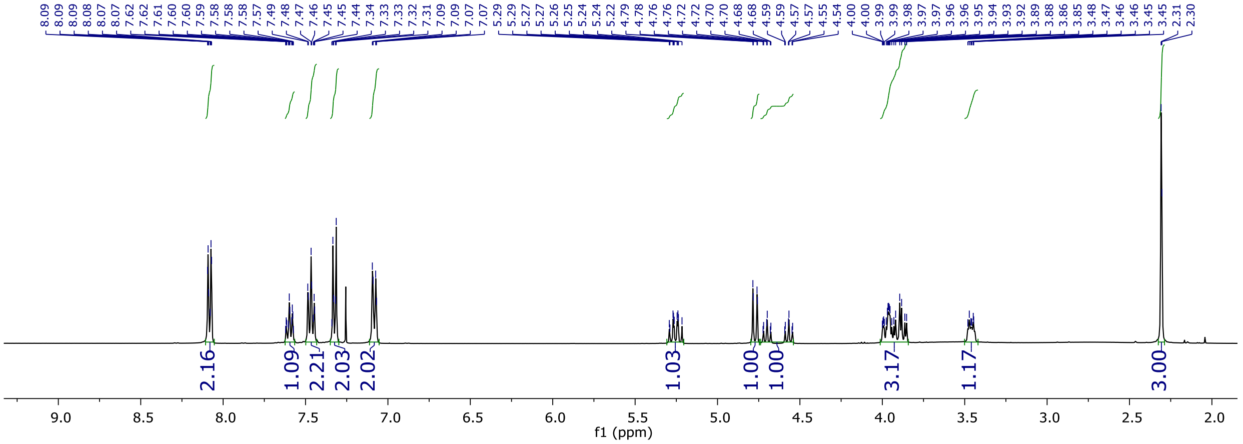
8.09
8.09
8.09
8.08
8.07
8.07
7.62
7.62
7.61
7.60
7.60
7.59
7.58
7.58
7.58
7.57
7.49
7.48
7.47
7.46
7.45
7.45
7.44
7.34
7.33
7.33
7.32
7.31
7.09
7.09
7.07
5.29
5.29
5.27
5.27
5.26
5.25
5.24
5.24
5.22
4.79
4.78
4.76
4.76
4.72
4.72
4.70
4.70
4.68
4.68
4.59
4.59
4.57
4.57
4.55
4.54
4.00
4.00
3.99
3.99
3.98
3.97
3.97
3.96
3.96
3.95
3.94
3.93
3.92
3.89
3.88
3.86
3.85
3.48
3.47
3.46
3.46
3.45
3.45
2.31
2.30
2.16
1.09
2.21
2.03
2.02
1.03
1.00
1.00
3.17
1.17
3.00
9.0
8.5
8.0
7.5
7.0
6.5
6.0
5.5
5.0
4.5
4.0
3.5
3.0
2.5
2.0
f1 (ppm)

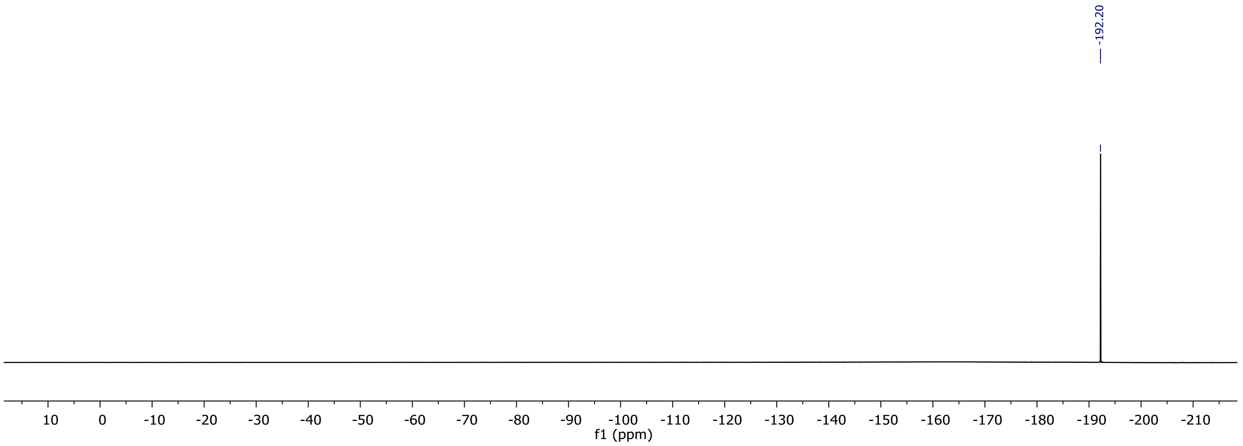
-192.20
10
0
-10
-20
-30
-40
-50
-60
-70
-80
-90
-100
-110
-120
-130
-140
-150
-160
-170
-180
-190
-200
-210
f1 (ppm)

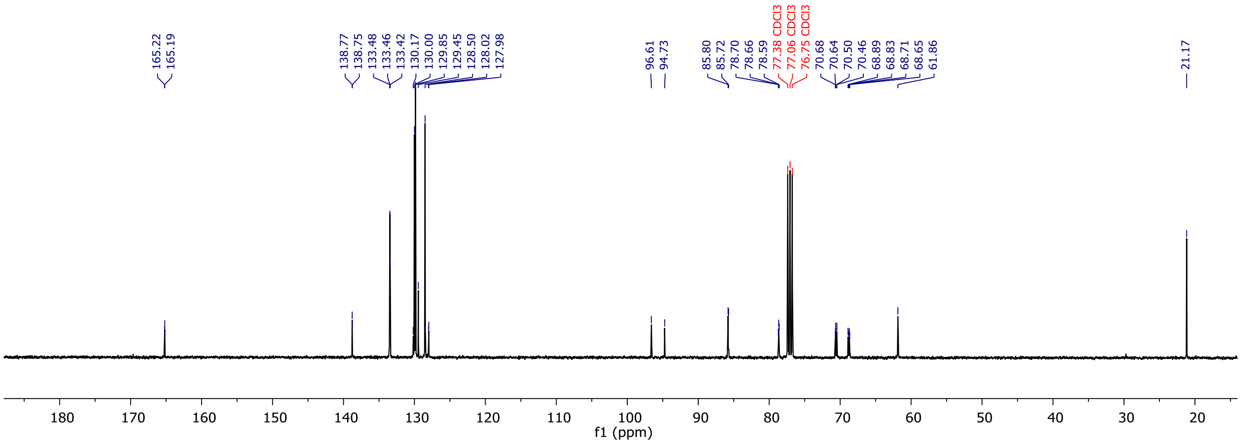
165.22
165.19
138.77
138.75
133.48
133.46
133.42
130.17
130.00
129.85
129.45
128.50
128.02
127.98
96.61
94.73
85.80
85.72
78.70
78.66
78.59
77.38 CDCl3
77.06 CDCl3
76.75 CDCl3
70.68
70.64
70.50
70.46
68.89
68.83
68.71
68.65
61.86
21.17
f1 (ppm)

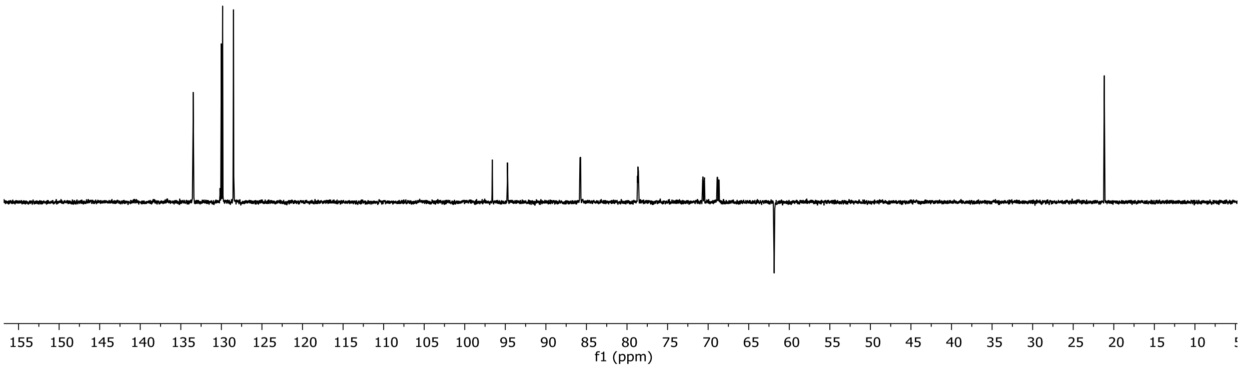
f1 (ppm)

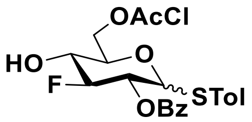
OAcCl
HO
O
F
OBz
STol

11

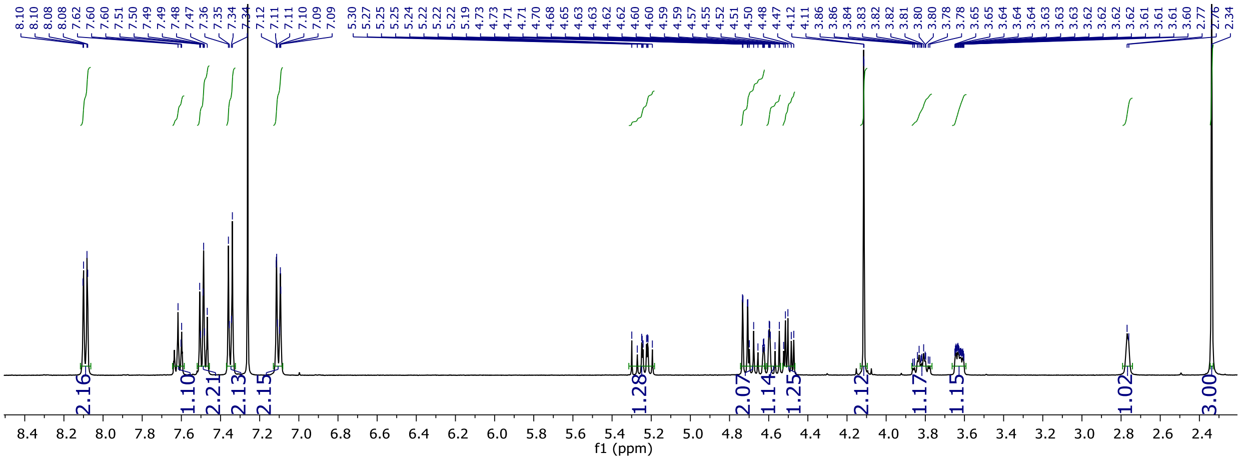
2.16
1.10
2.21
2.13
2.15
1.28
2.07
1.14
1.25
2.12
1.17
1.15
1.02
3.00
8.4 8.2 8.0 7.8 7.6 7.4 7.2 7.0 6.8 6.6 6.4 6.2 6.0 5.8 5.6 5.4 5.2 5.0 4.8 4.6 4.4 4.2 4.0 3.8 3.6 3.4 3.2 3.0 2.8 2.6 2.4
f1 (ppm)

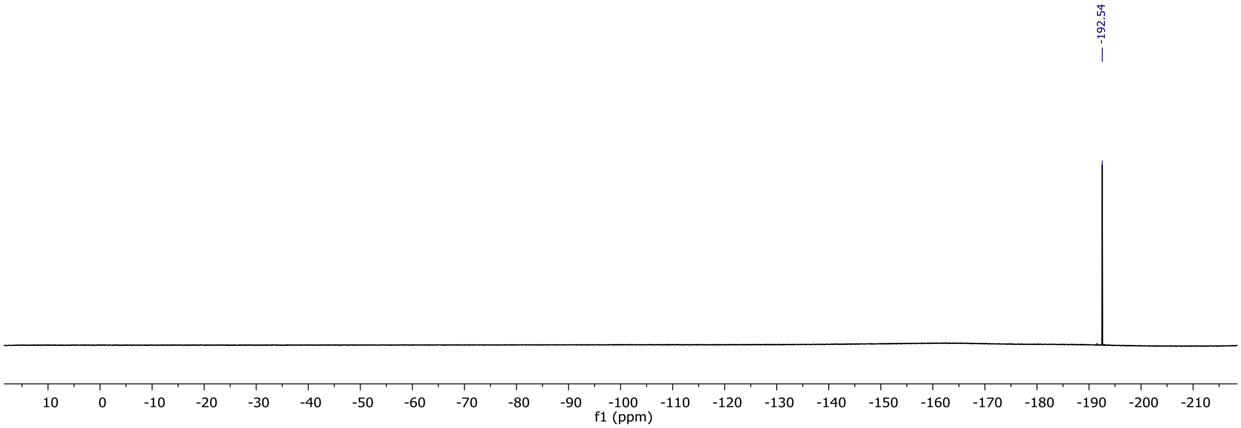
-192.54
10 0 -10 -20 -30 -40 -50 -60 -70 -80 -90 -100 -110 -120 -130 -140 -150 -160 -170 -180 -190 -200 -210
f1 (ppm)

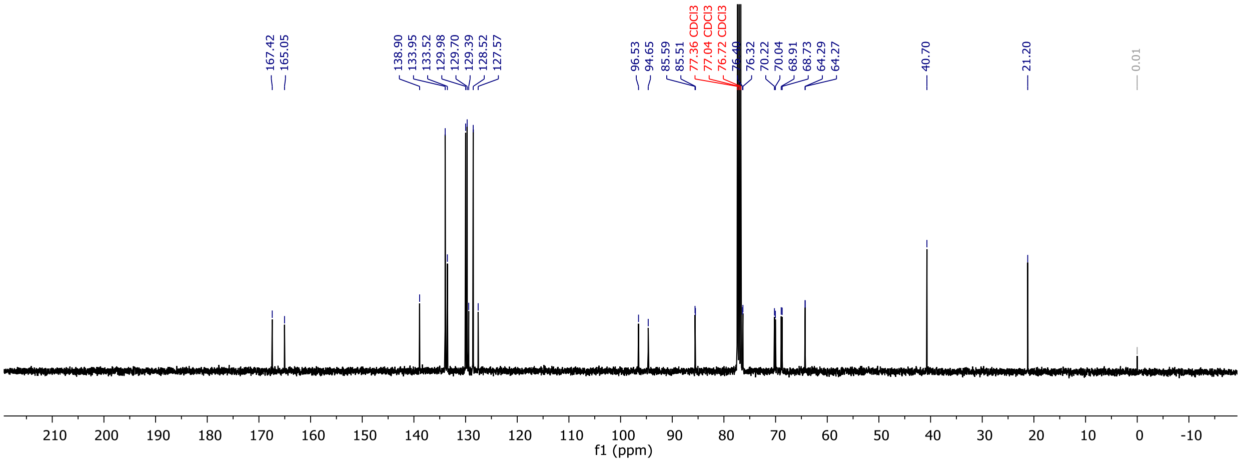
167.42
165.05
138.90
133.95
133.52
129.98
129.70
129.39
128.52
127.57
96.53
94.65
85.59
85.51
77.36 CDCl3
77.04 CDCl3
76.72 CDCl3
76.40
76.32
70.22
70.04
68.91
68.73
64.29
64.27
40.70
21.20
0.01
210 200 190 180 170 160 150 140 130 120 110 100 90 80 70 60 50 40 30 20 10 0 -10
f1 (ppm)

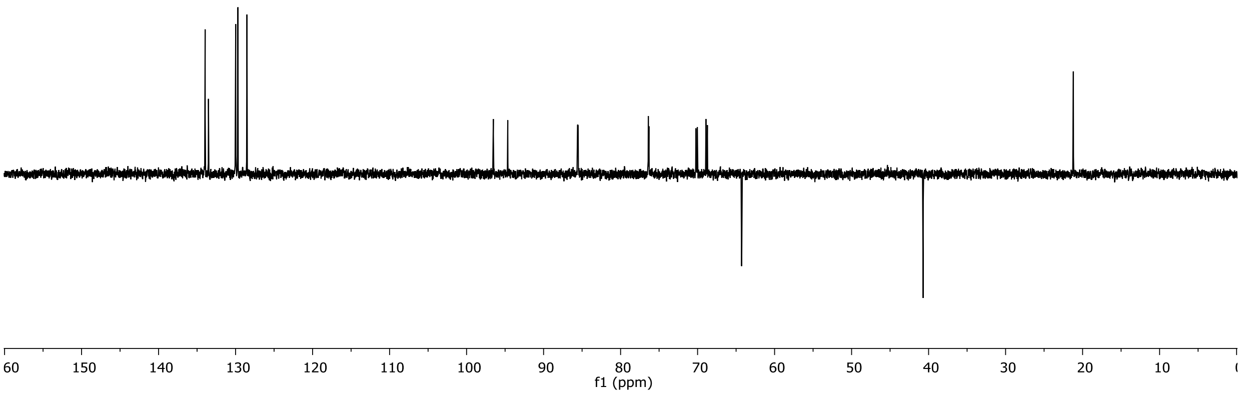
60 150 140 130 120 110 100 90 80 70 60 50 40 30 20 10
f1 (ppm)

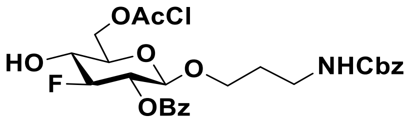
OAcCl
HO
O
O
NHCbz
F
OBz

12

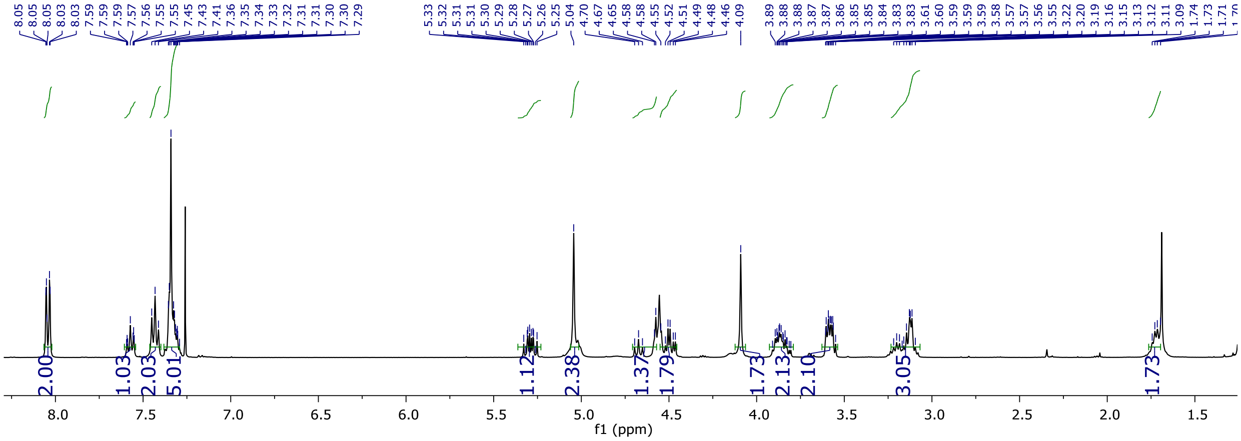
2.00
1.03
2.03
5.01
1.12
2.38
1.37
1.79
1.73
2.13
2.10
3.05
1.73
8.0
7.5
7.0
6.5
6.0
5.5
5.0
4.5
4.0
3.5
3.0
2.5
2.0
1.5
f1 (ppm)

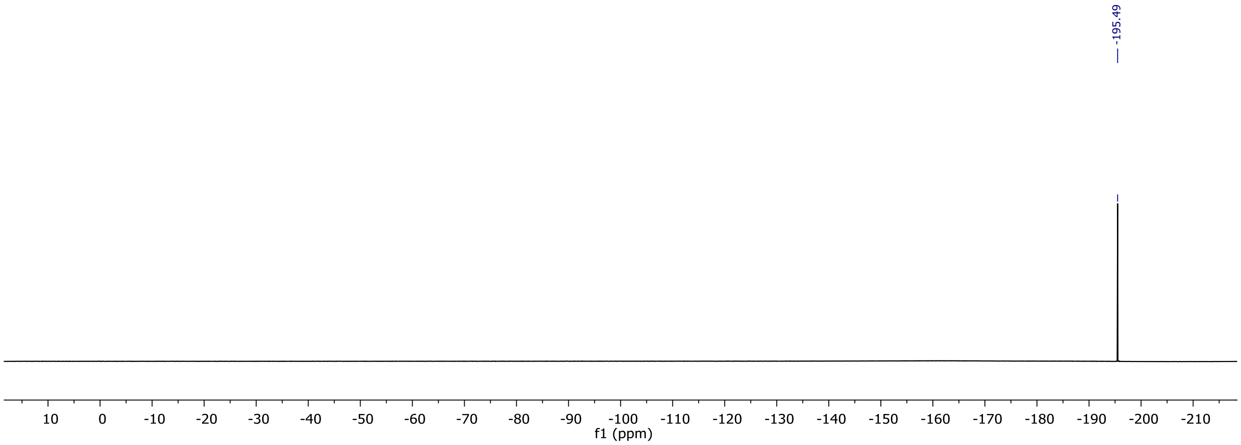
-195.49
10
0
-10
-20
-30
-40
-50
-60
-70
-80
-90
-100
-110
-120
-130
-140
-150
-160
-170
-180
-190
-200
-210
f1 (ppm)

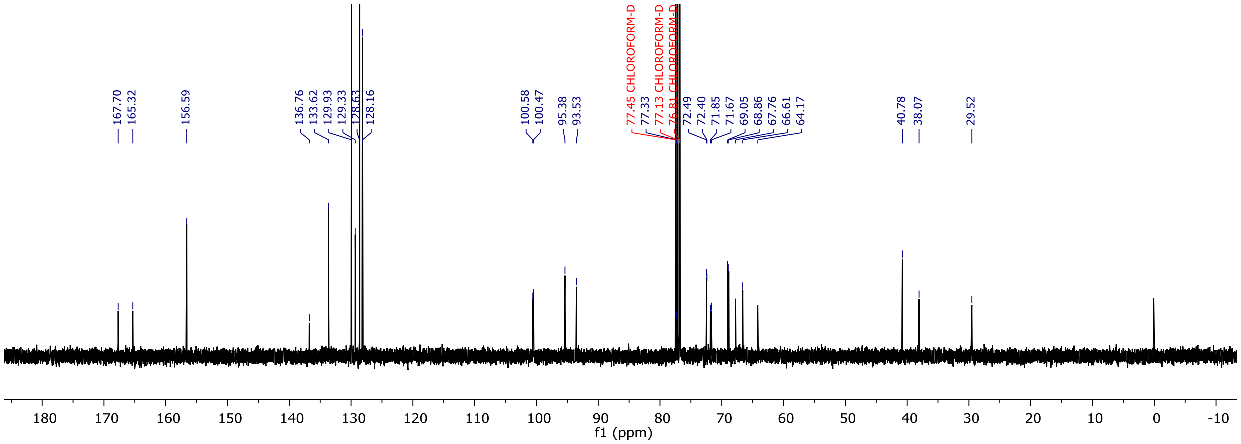
167.70
165.32
156.59
136.76
133.62
129.93
129.33
128.63
128.16
100.58
100.47
95.38
93.53
77.45 CHLOROFORM-D
77.33
77.13 CHLOROFORM-D
76.81 CHLOROFORM-D
72.49
72.40
71.85
71.67
69.05
68.86
67.76
66.61
64.17
40.78
38.07
29.52
f1 (ppm)

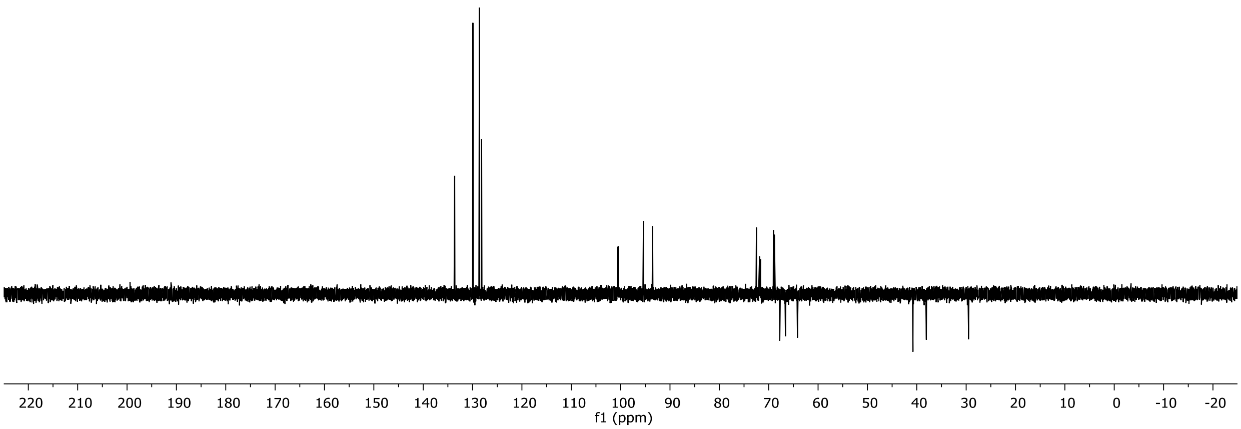
f1 (ppm)

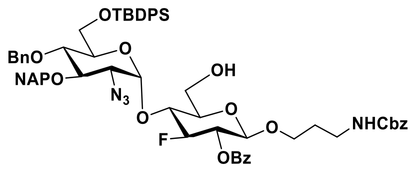

15b

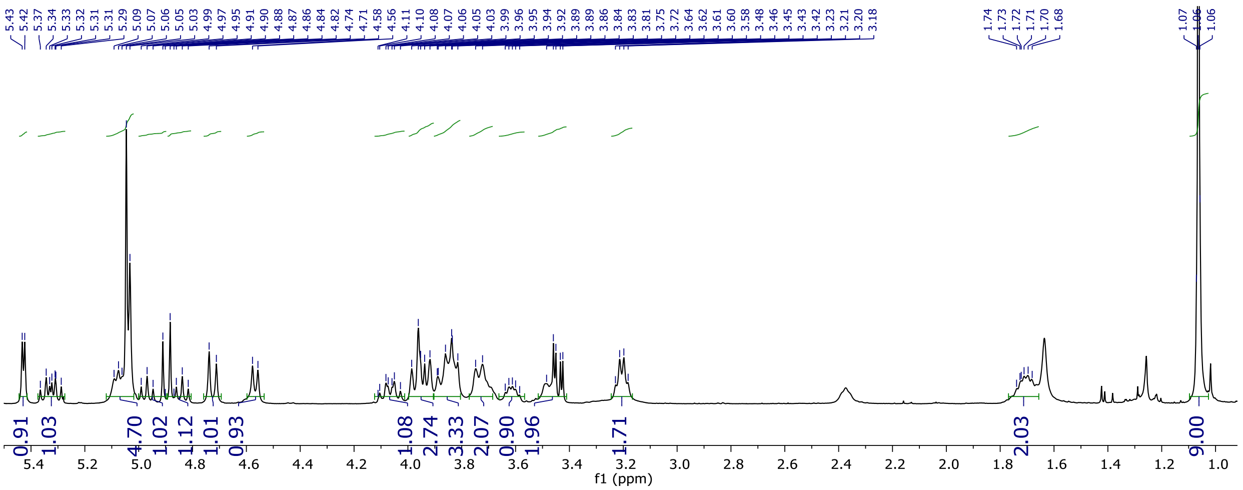

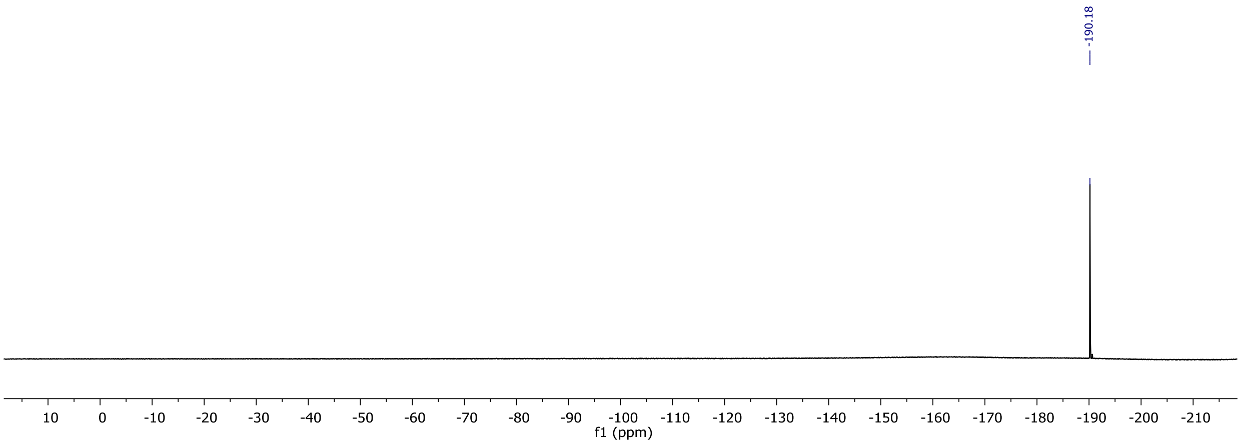

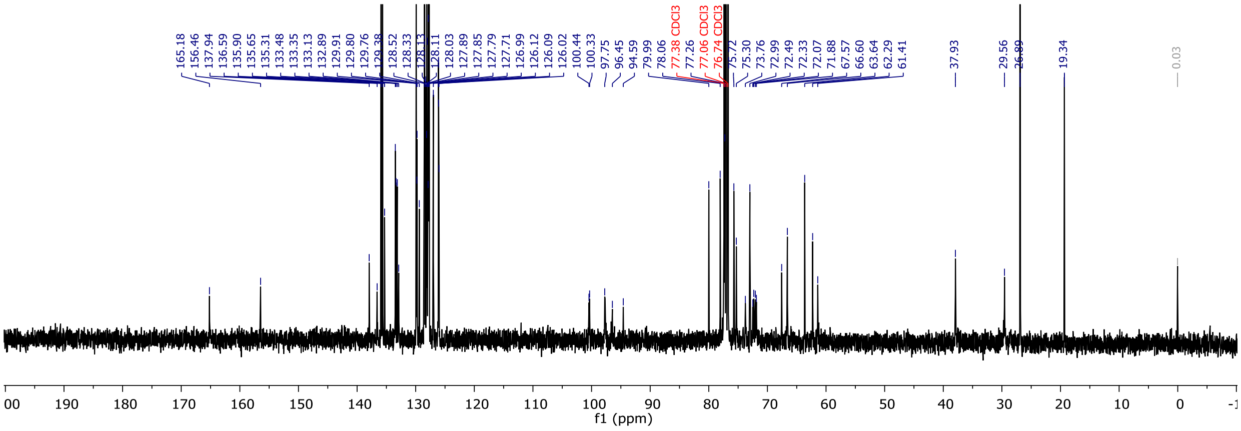
165.18
156.46
137.94
136.59
135.90
135.65
135.31
133.48
133.35
133.13
132.89
129.91
129.80
129.76
129.38
128.52
128.33
128.13
128.11
128.03
127.89
127.85
127.79
127.71
126.99
126.12
126.09
126.02
100.44
100.33
97.75
96.45
94.59
79.99
78.06
77.38 CDCl3
77.26
77.06 CDCl3
76.74 CDCl3
75.72
75.30
73.76
72.99
72.49
72.33
72.07
71.88
67.57
66.60
63.64
62.29
61.41
37.93
29.56
26.89
19.34
0.03
f1 (ppm)

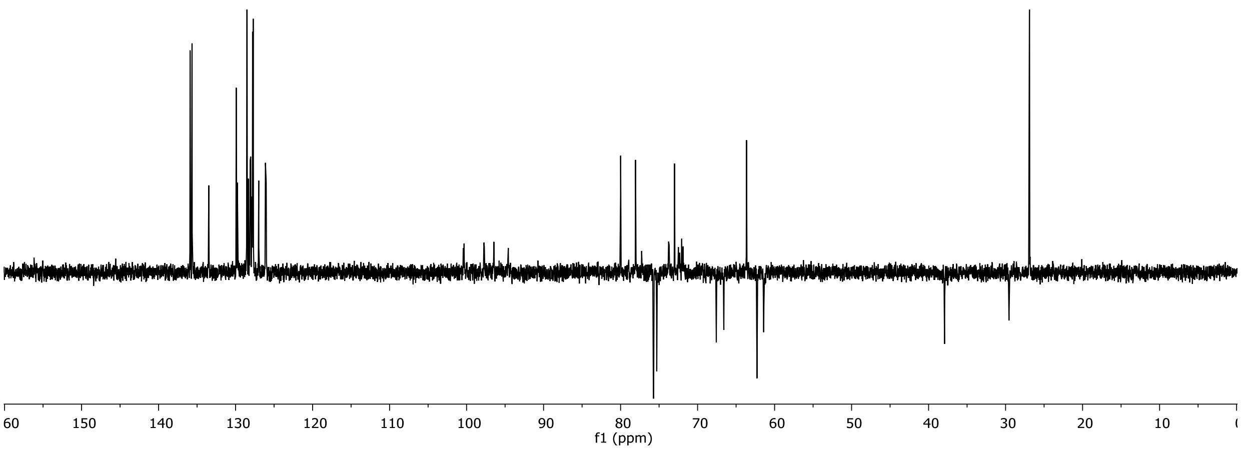
f1 (ppm)

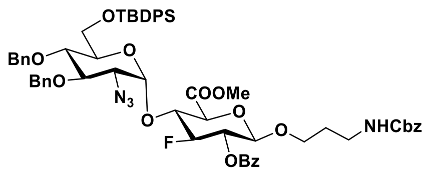

16a

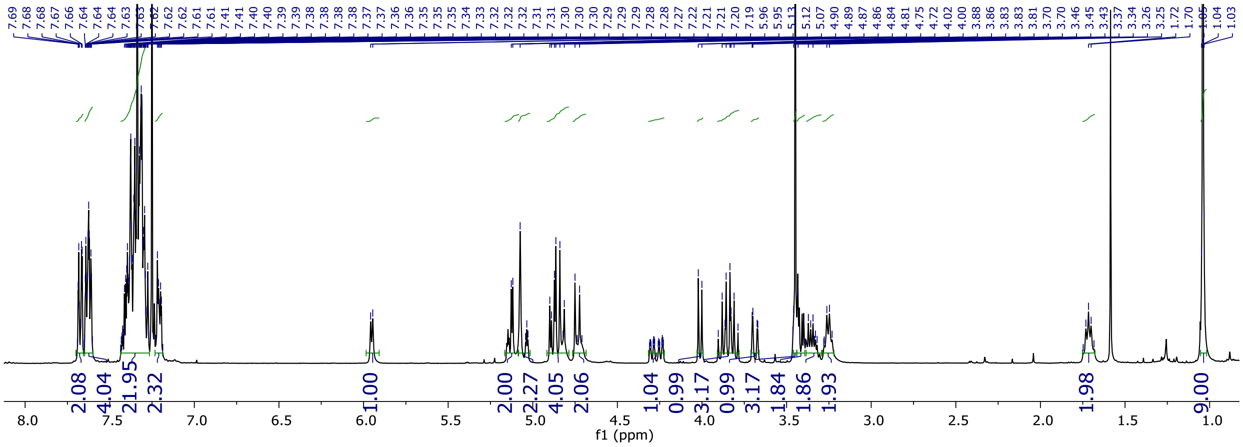

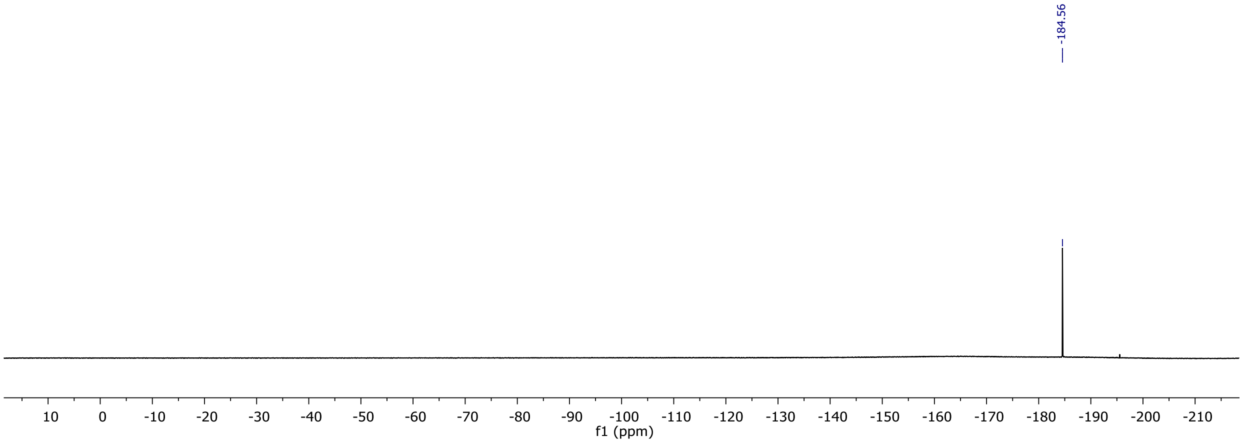

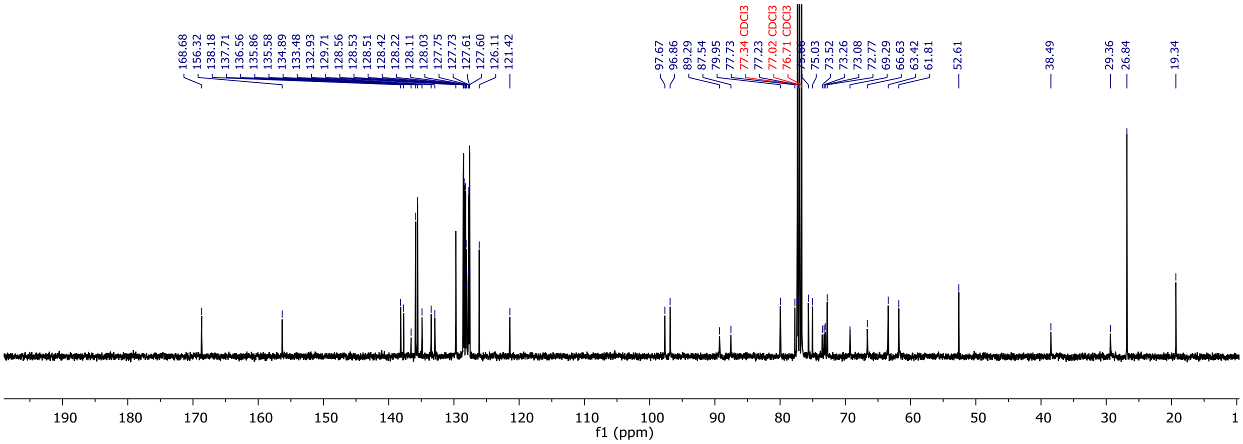
168.68
156.32
138.18
137.71
136.56
135.86
135.58
134.89
133.48
132.93
129.71
128.56
128.53
128.51
128.42
128.22
128.11
128.03
127.75
127.73
127.61
127.60
126.11
121.42
97.67
96.86
89.29
87.54
79.95
77.73
77.34 CDCl3
77.23
77.02 CDCl3
76.71 CDCl3
75.66
75.03
73.52
73.26
73.08
72.77
69.29
66.63
63.42
61.81
52.61
38.49
29.36
26.84
19.34
f1 (ppm)

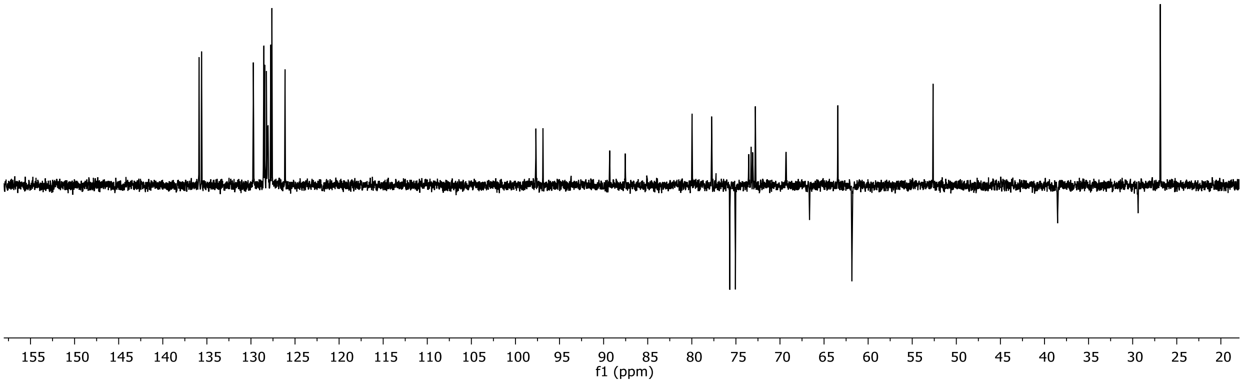
f1 (ppm)

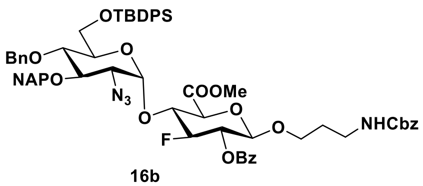

16b

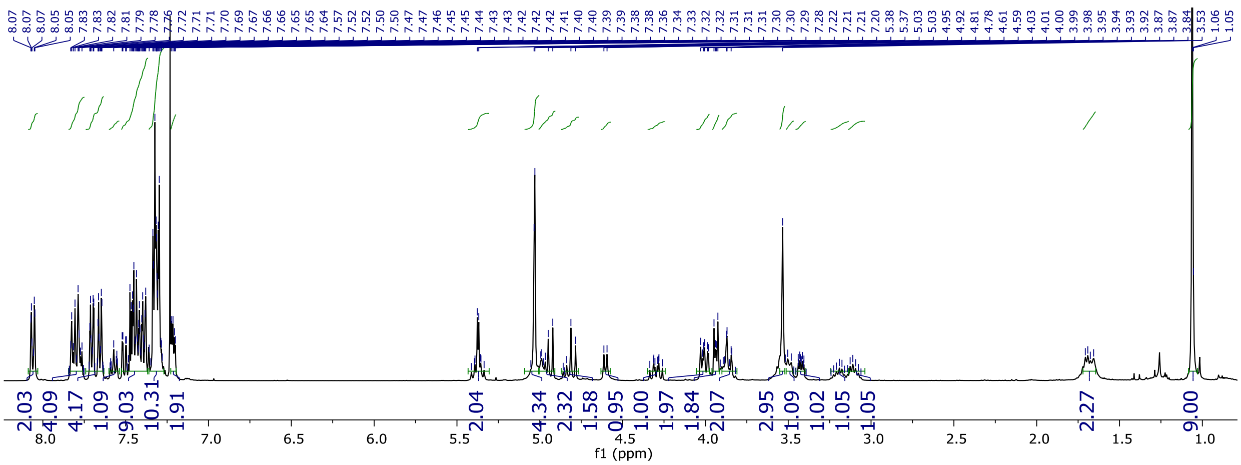

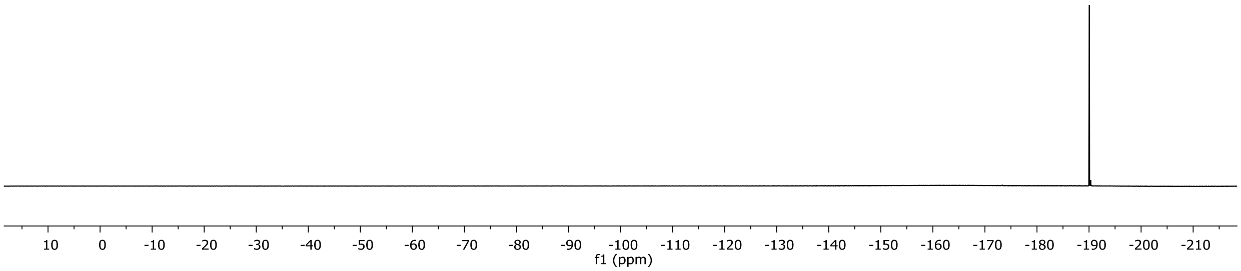

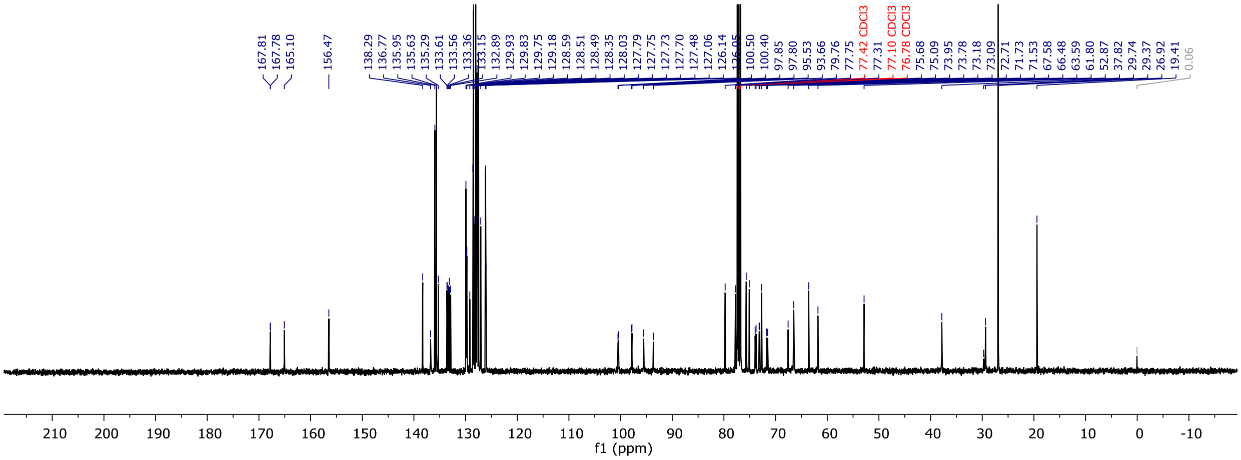
167.81
167.78
165.10
156.47
138.29
136.77
135.95
135.63
135.29
133.61
133.56
133.36
133.15
132.89
129.93
129.83
129.75
129.18
128.59
128.51
128.49
128.35
128.03
127.79
127.75
127.73
127.70
127.48
127.06
126.14
126.05
100.50
100.40
97.85
97.80
95.53
93.66
79.76
77.75
77.42 CDCl3
77.31
77.10 CDCl3
76.78 CDCl3
75.68
75.09
73.95
73.78
73.18
73.09
72.71
71.73
71.53
67.58
66.48
63.59
61.80
52.87
37.82
29.74
29.37
26.92
19.41
0.06
f1 (ppm)

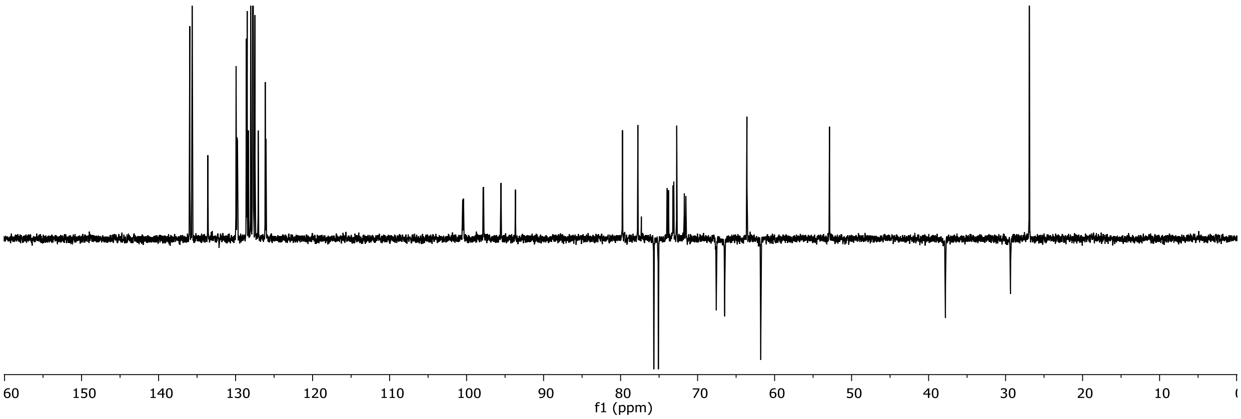
f1 (ppm)

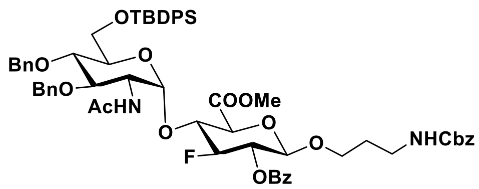
OTBDPS
BnO
BnO
AcHN
O
COOMe
O
O
O
F
OBz
NHCbz

17a

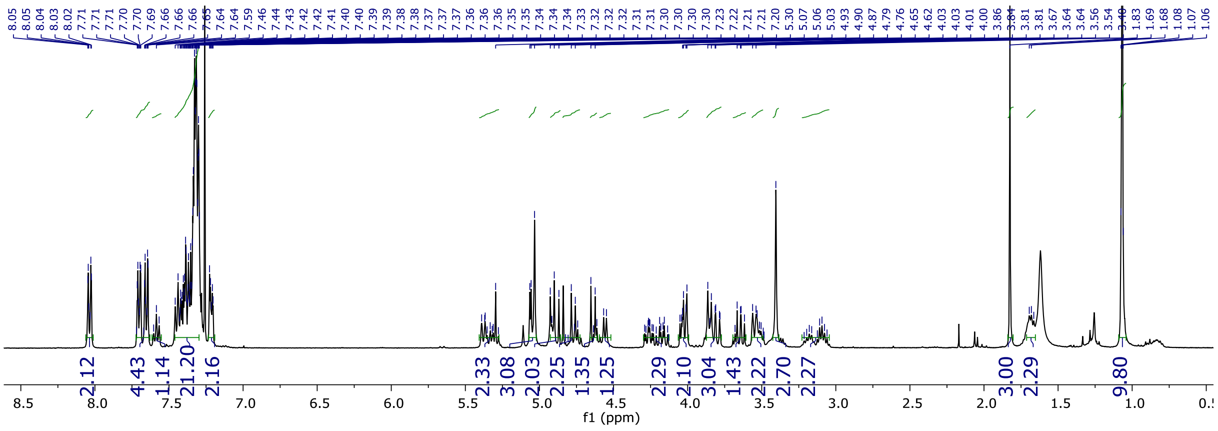
8.05 8.05 8.04 8.03 8.02 7.71 7.71 7.71 7.70 7.69 7.66 7.66 7.66 7.65 7.64 7.59 7.46 7.44 7.43 7.42 7.42 7.41 7.40 7.39 7.39 7.38 7.38 7.37 7.37 7.36 7.36 7.35 7.34 7.34 7.33 7.32 7.32 7.32 7.31 7.30 7.30 7.30 7.30 7.23 7.22 7.22 7.21 7.20 5.30 5.07 5.06 5.03 4.93 4.90 4.87 4.79 4.76 4.65 4.62 4.03 4.01 4.00 3.86 3.84 3.81 3.67 3.64 3.64 3.56 3.54 3.40 1.83 1.69 1.68 1.08 1.07 1.06
2.12 4.43 1.14 21.20 2.16 2.33 3.08 2.03 2.25 1.35 1.25 2.29 2.10 3.04 1.43 2.22 2.70 2.27 3.00 2.29 9.80
8.5 8.0 7.5 7.0 6.5 6.0 5.5 5.0 4.5 4.0 3.5 3.0 2.5 2.0 1.5 1.0 0.5
f1 (ppm)

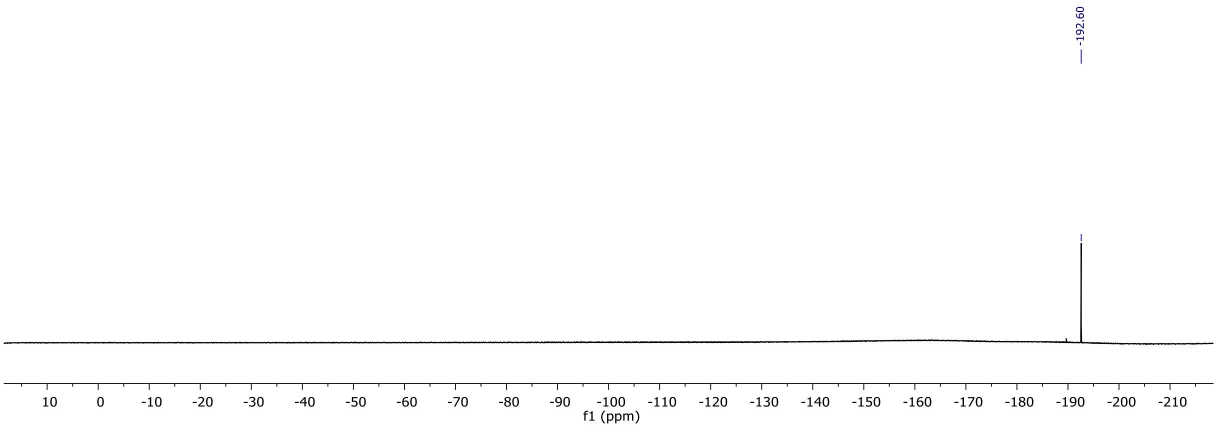
-192.60
10 0 -10 -20 -30 -40 -50 -60 -70 -80 -90 -100 -110 -120 -130 -140 -150 -160 -170 -180 -190 -200 -210
f1 (ppm)

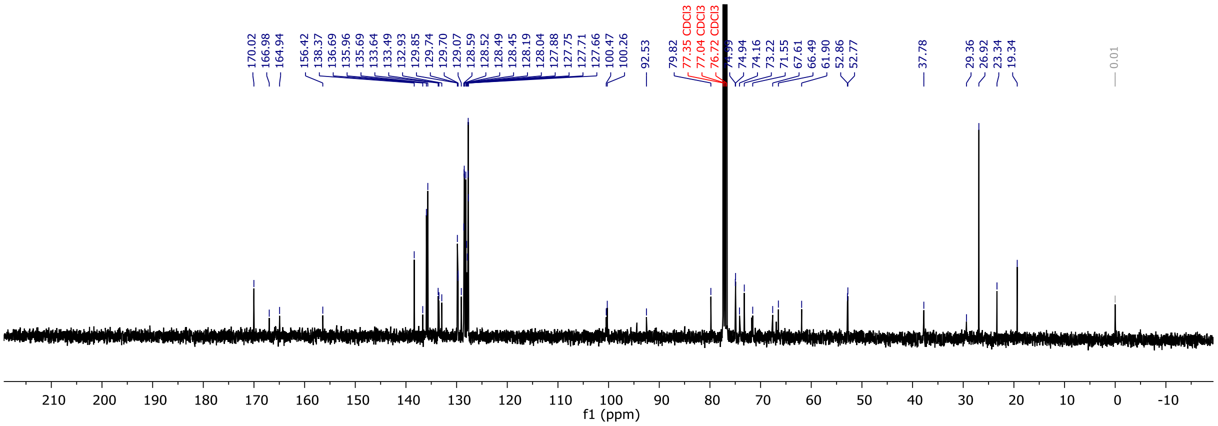
170.02
166.98
164.94
156.42
138.37
136.69
135.96
135.69
133.64
133.49
132.93
129.85
129.74
129.70
129.07
128.59
128.52
128.49
128.45
128.19
128.04
127.88
127.75
127.71
127.66
100.47
100.26
92.53
79.82
77.35 CDCl3
77.04 CDCl3
76.72 CDCl3
74.99
74.94
74.16
73.22
71.55
67.61
66.49
61.90
52.86
52.77
37.78
29.36
26.92
23.34
19.34
0.01
f1 (ppm)

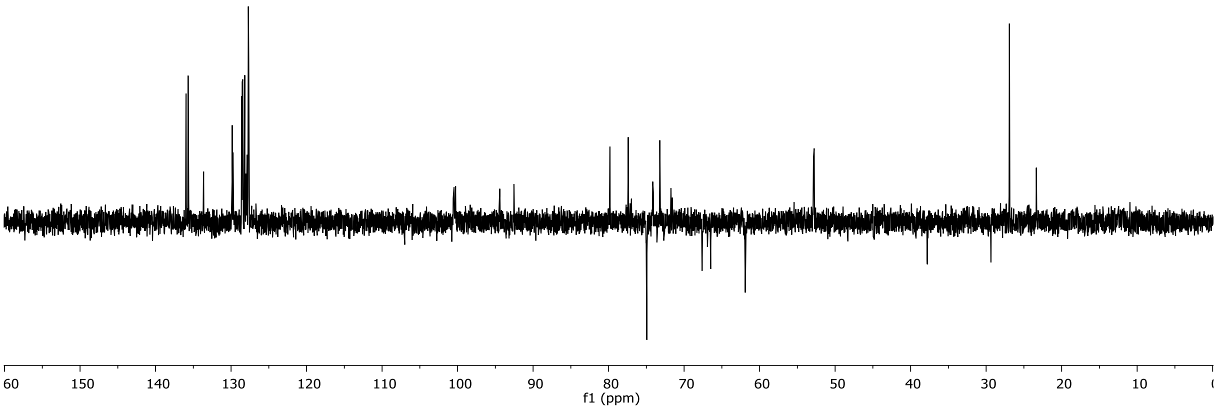
f1 (ppm)

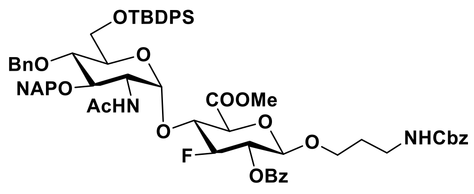

17b

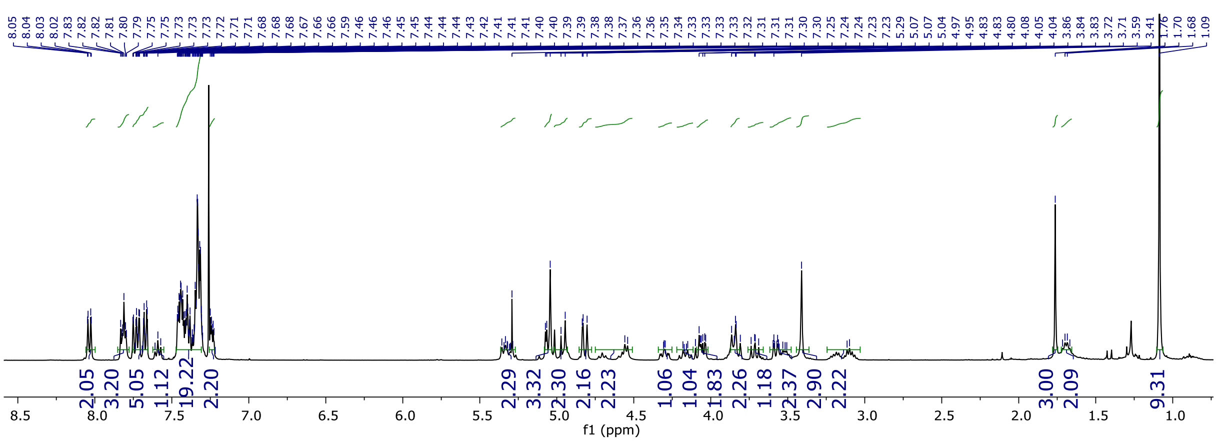

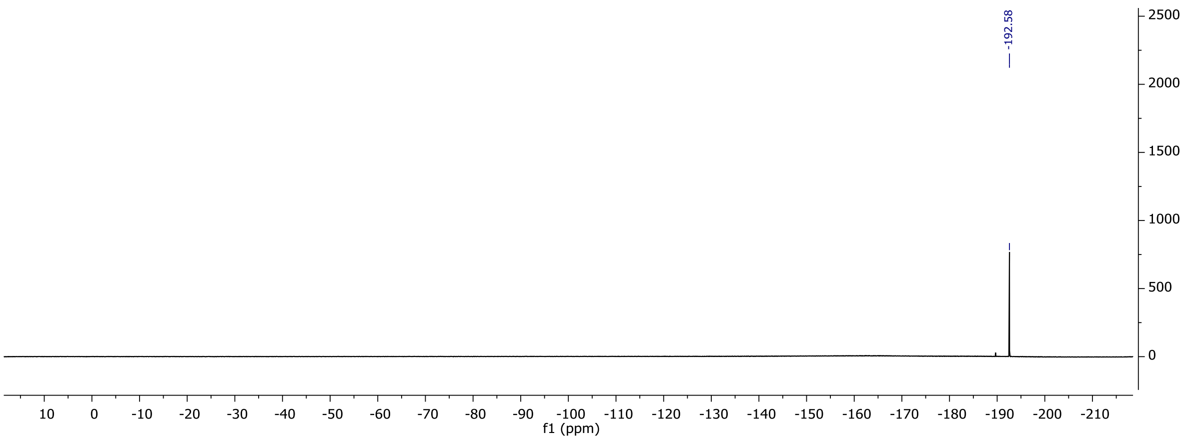

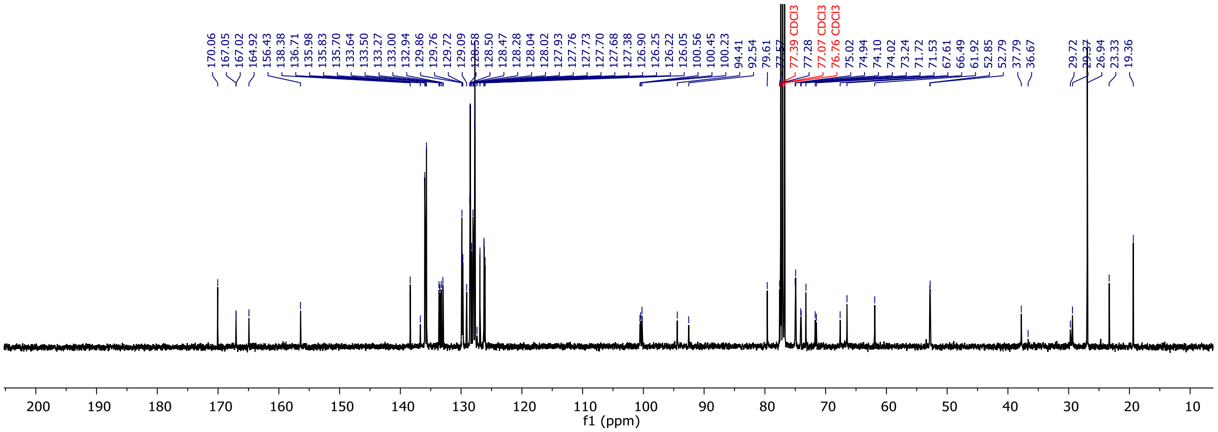

f1 (ppm)

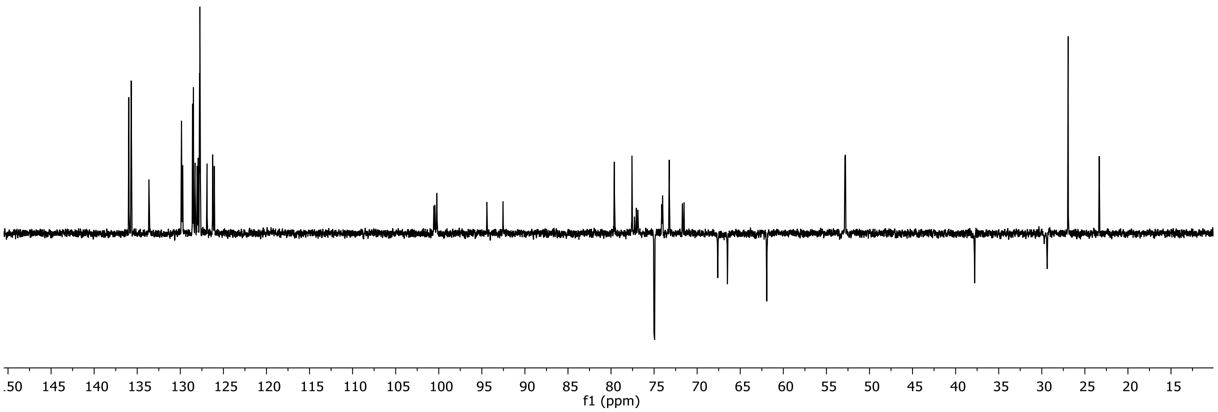

f1 (ppm)

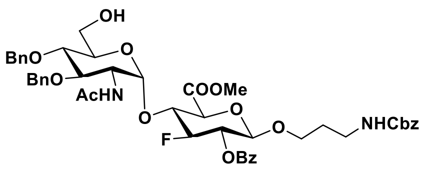
OH
BnO
BnO
AcHN
COOMe
O
F
OBz
NHCbz

17a - i

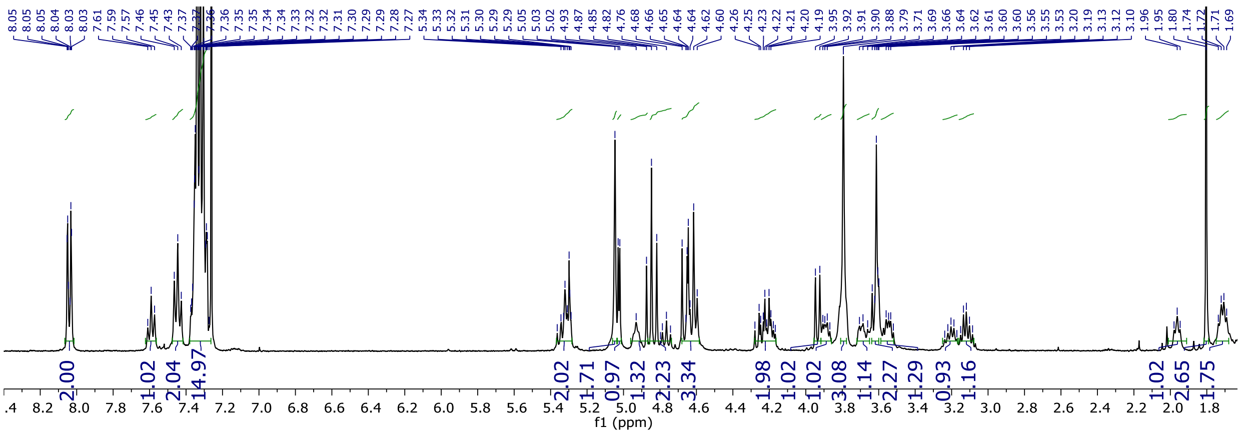
2.00
1.02
2.04
14.97
2.02
1.71
0.97
1.32
2.23
3.34
1.98
1.02
1.02
3.08
1.14
2.27
1.29
0.93
1.16
1.02
2.65
1.75
f1 (ppm)

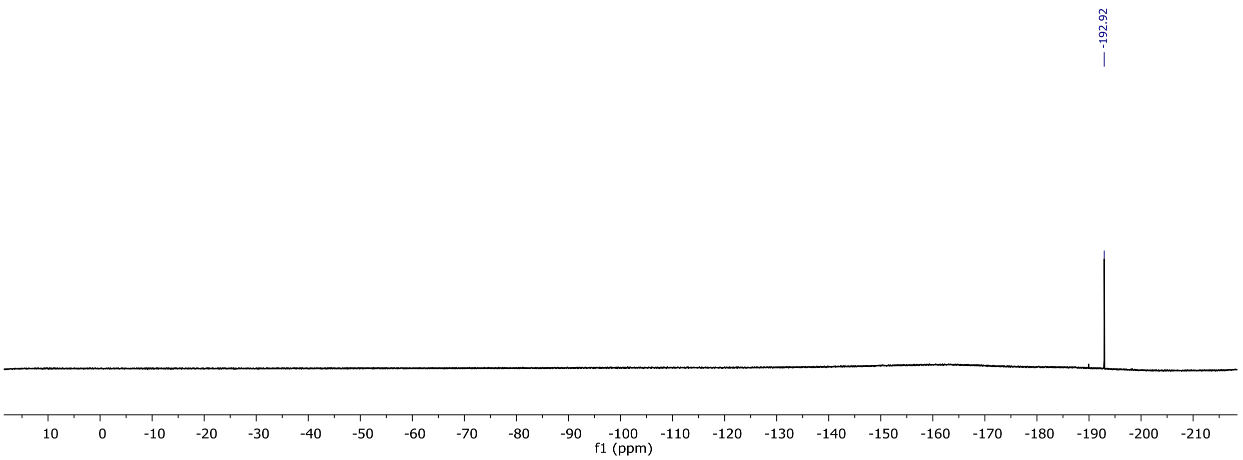
-192.92
f1 (ppm)

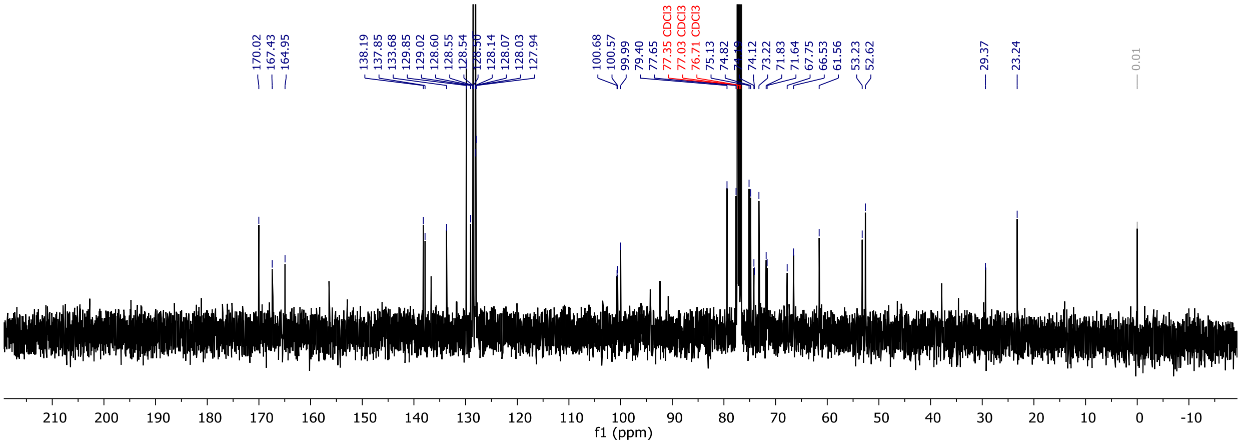

170.02
167.43
164.95
138.19
137.85
133.68
129.85
129.02
128.60
128.55
128.54
128.14
128.07
128.03
127.94
100.68
100.57
99.99
79.40
77.65
77.35 CDCl3
77.03 CDCl3
76.71 CDCl3
75.13
74.82
74.12
73.22
71.83
71.64
67.75
66.53
61.56
53.23
52.62
29.37
23.24
0.01
f1 (ppm)

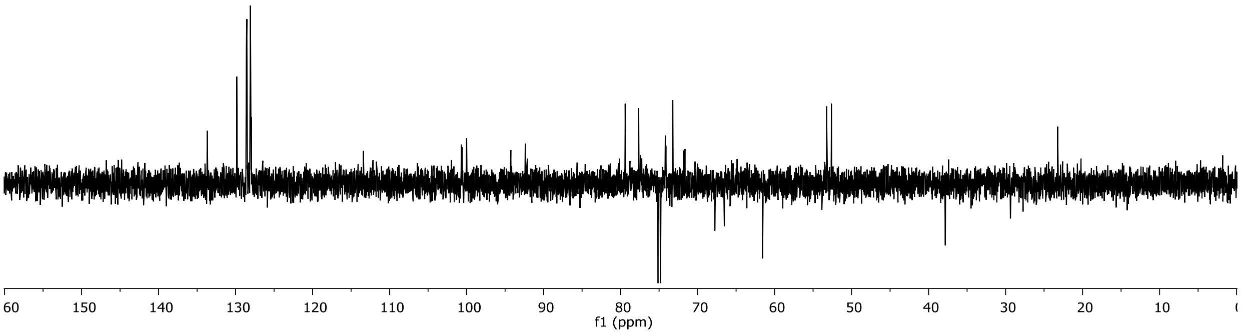

f1 (ppm)

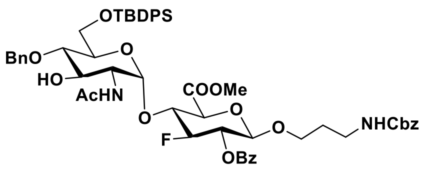

17b - i

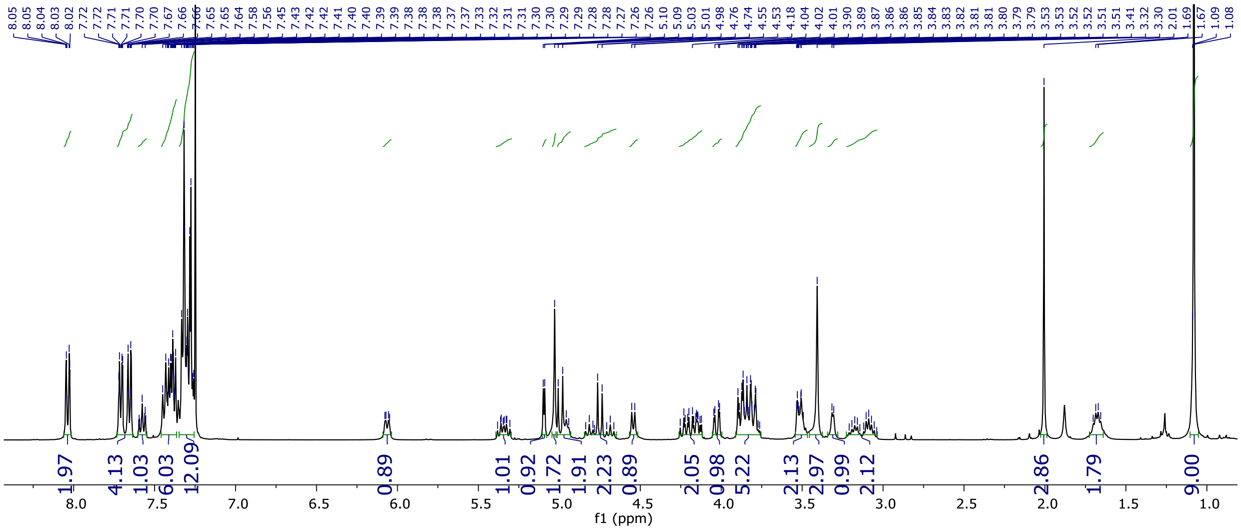

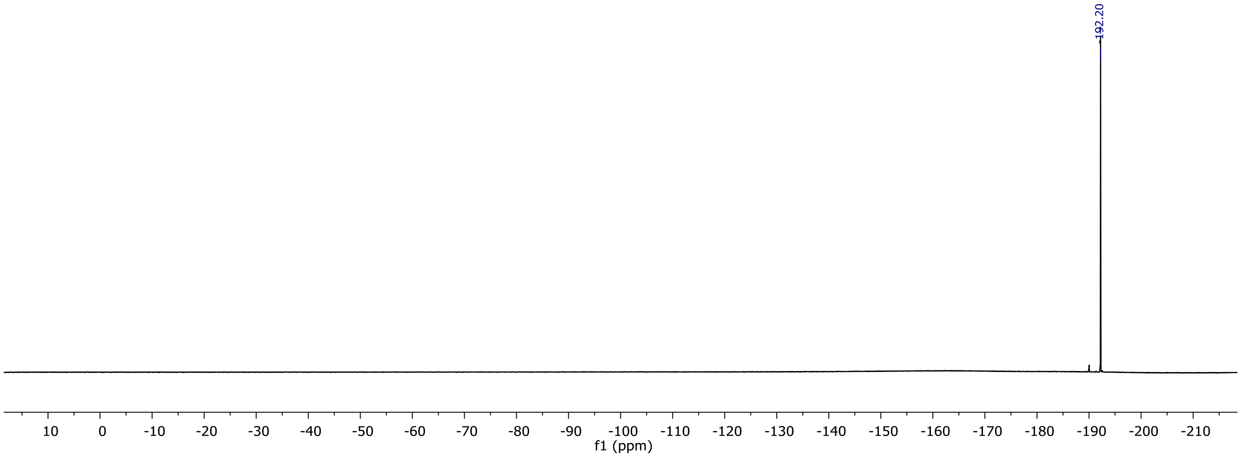

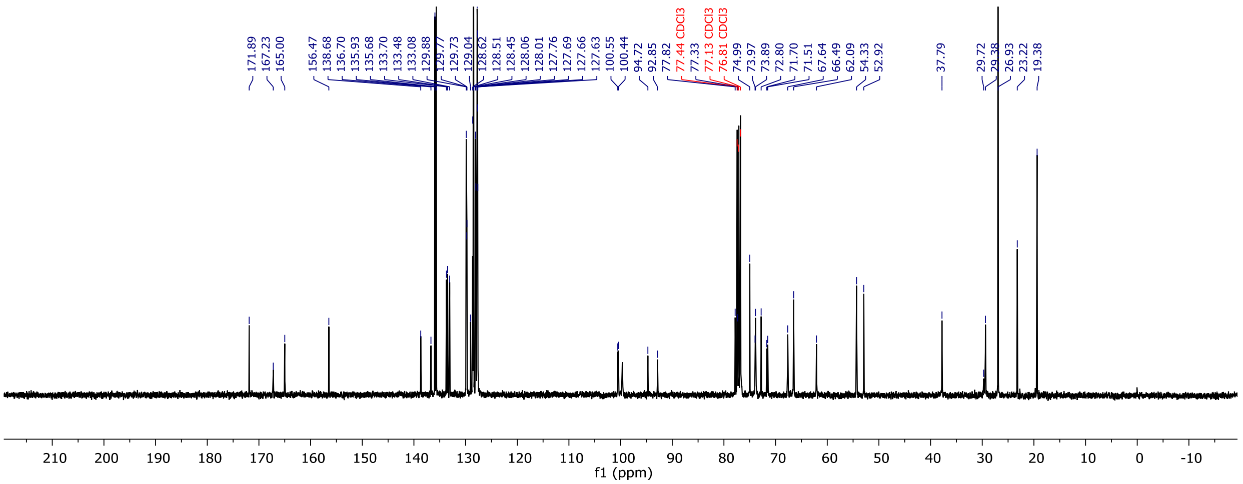
171.89
167.23
165.00
156.47
138.68
136.70
135.93
135.68
133.70
133.48
133.08
129.88
129.77
129.73
129.04
128.62
128.51
128.45
128.06
128.01
127.76
127.69
127.66
127.63
100.55
100.44
94.72
92.85
77.82
77.44 CDCl3
77.33
77.13 CDCl3
76.81 CDCl3
74.99
73.97
73.89
72.80
71.70
71.51
67.64
66.49
62.09
54.33
52.92
37.79
29.72
29.38
26.93
23.22
19.38
f1 (ppm)

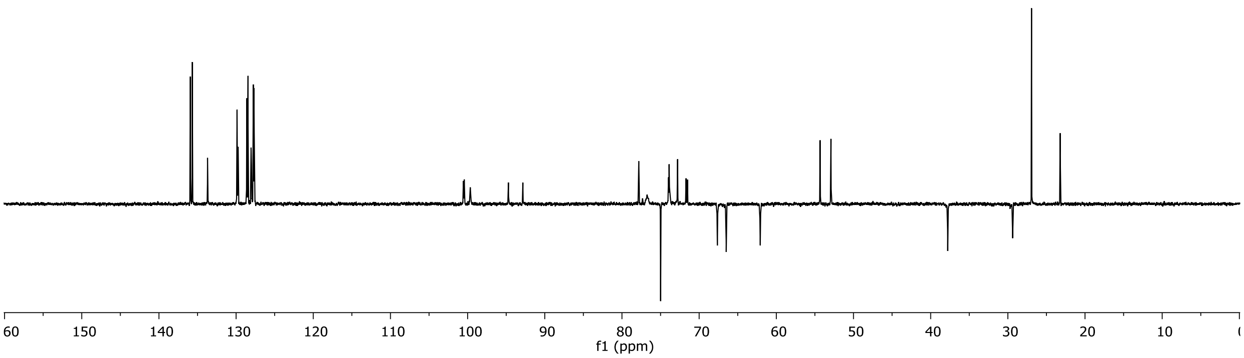
f1 (ppm)

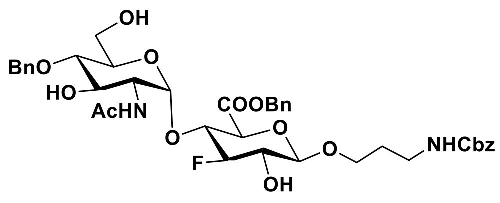
OH
BnO
HO
AcHN
COOBn
O
F
OH
NHCbz

17b - ii

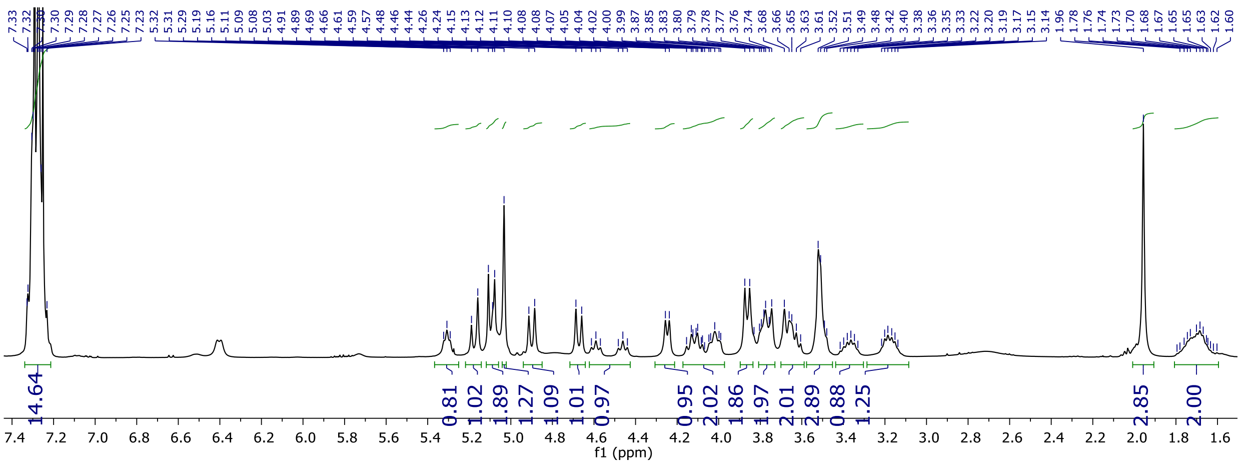
14.64
0.81
1.02
1.89
1.27
1.09
1.01
0.97
0.95
2.02
1.86
1.97
2.01
2.89
0.88
1.25
2.85
2.00
f1 (ppm)

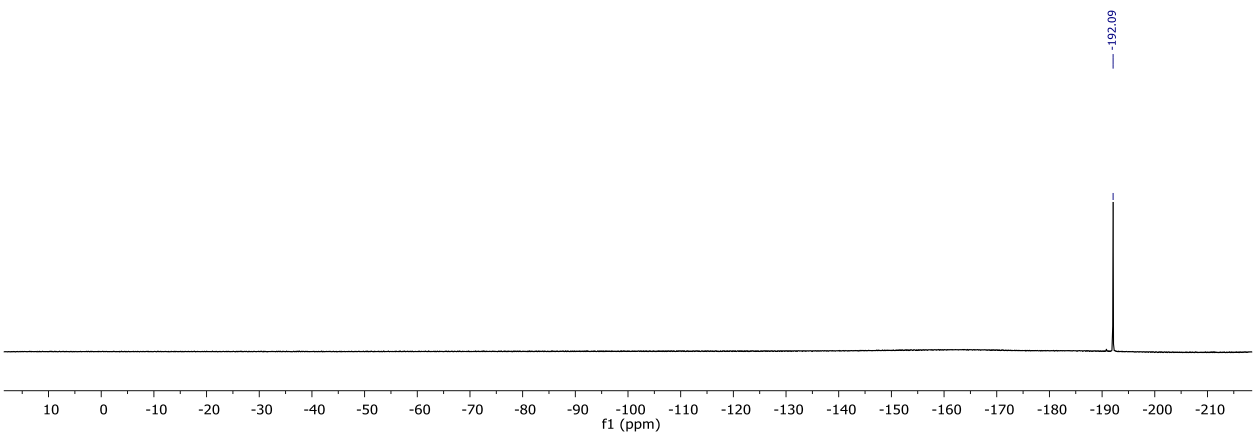
-192.09
f1 (ppm)

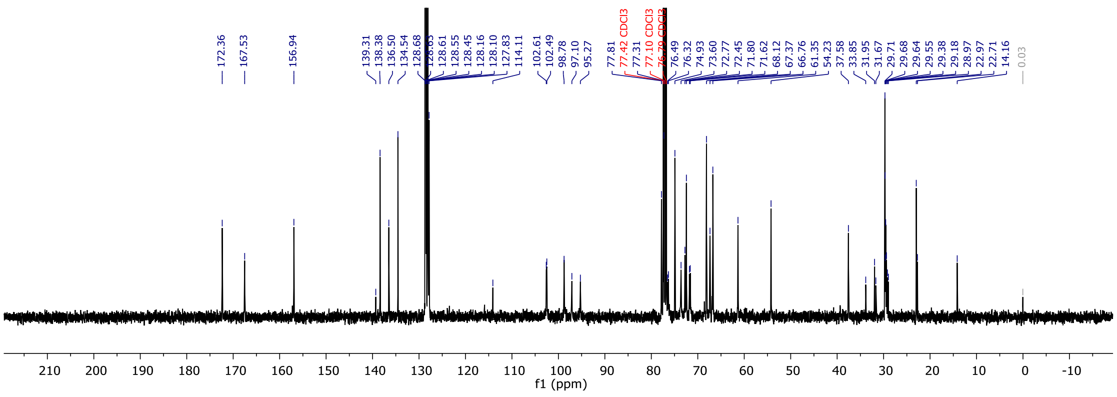

f1 (ppm)

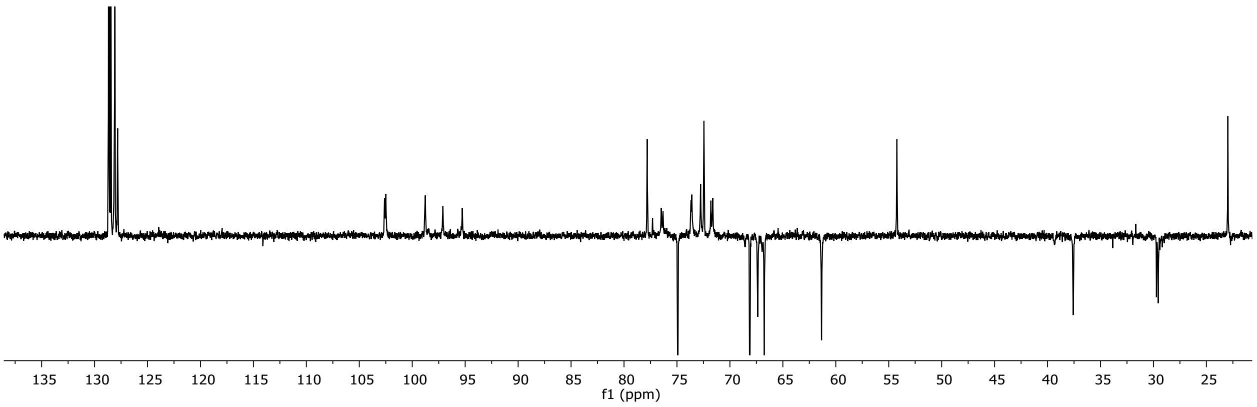

f1 (ppm)

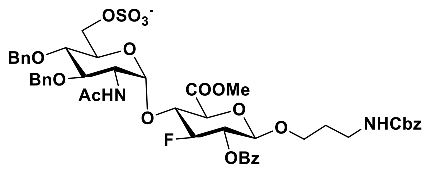

18

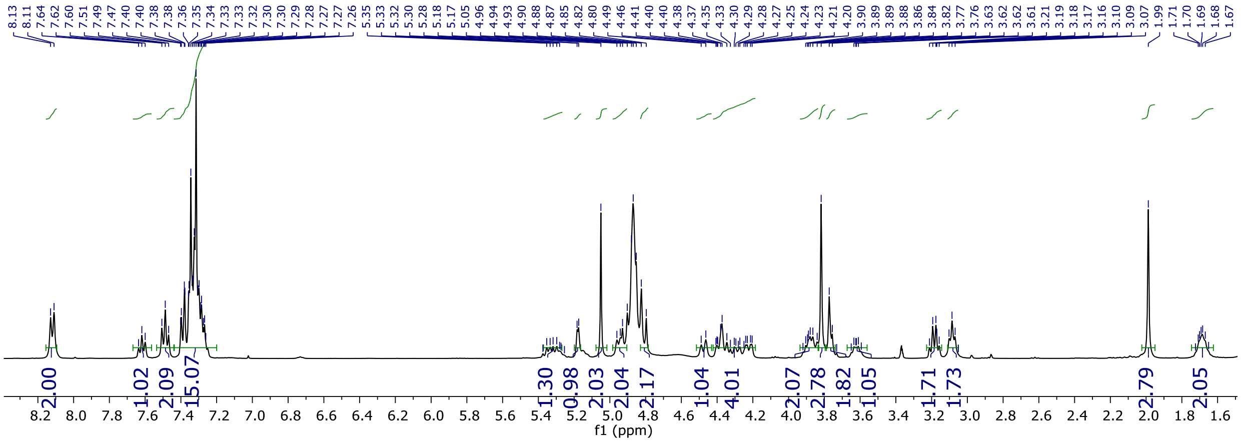

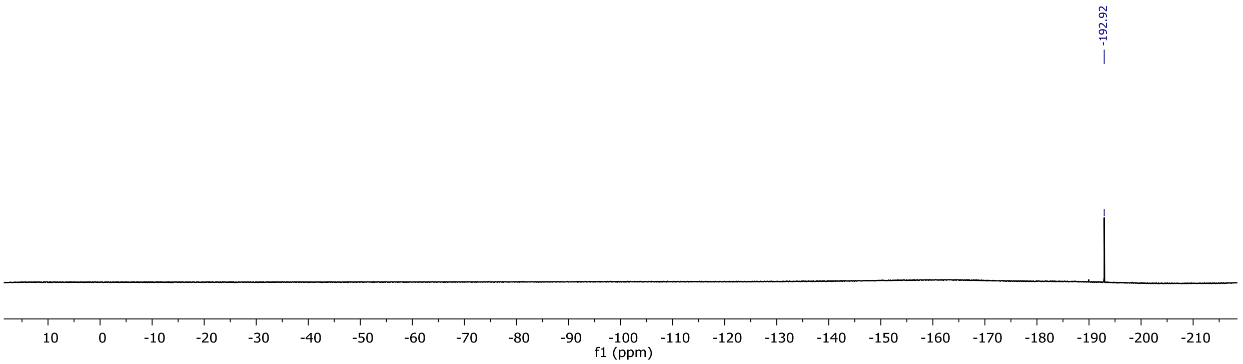
-192.92
f1 (ppm)

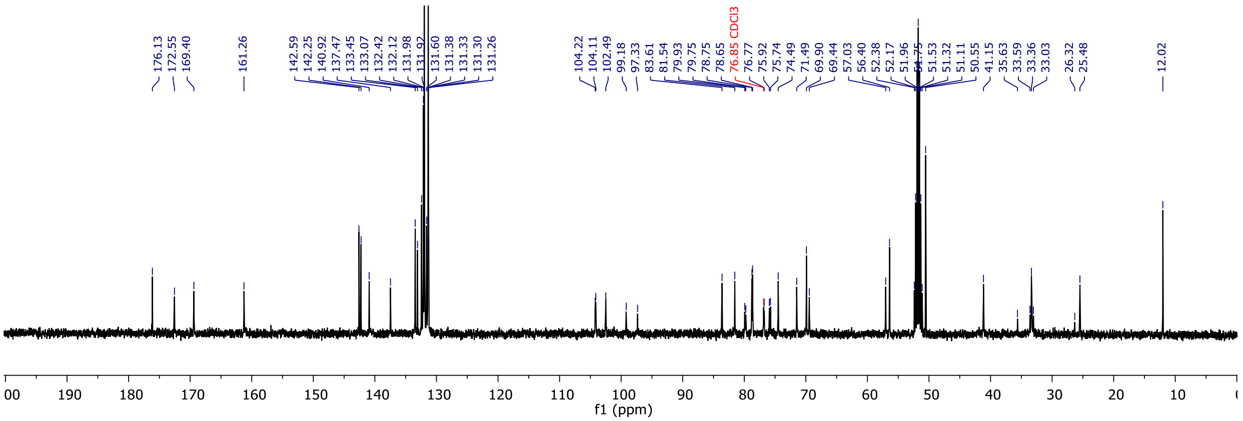
176.13
172.55
169.40
161.26
142.59
142.25
140.92
137.47
133.45
133.07
132.42
132.12
131.98
131.92
131.60
131.38
131.33
131.30
131.26
104.22
104.11
102.49
99.18
97.33
83.61
81.54
79.93
79.75
78.75
78.65
76.85 CDCl3
76.77
75.92
75.74
74.49
71.49
69.90
69.44
57.03
56.40
52.38
52.17
51.96
51.75
51.53
51.32
51.11
50.55
41.15
35.63
33.59
33.36
33.03
26.32
25.48
12.02
f1 (ppm)

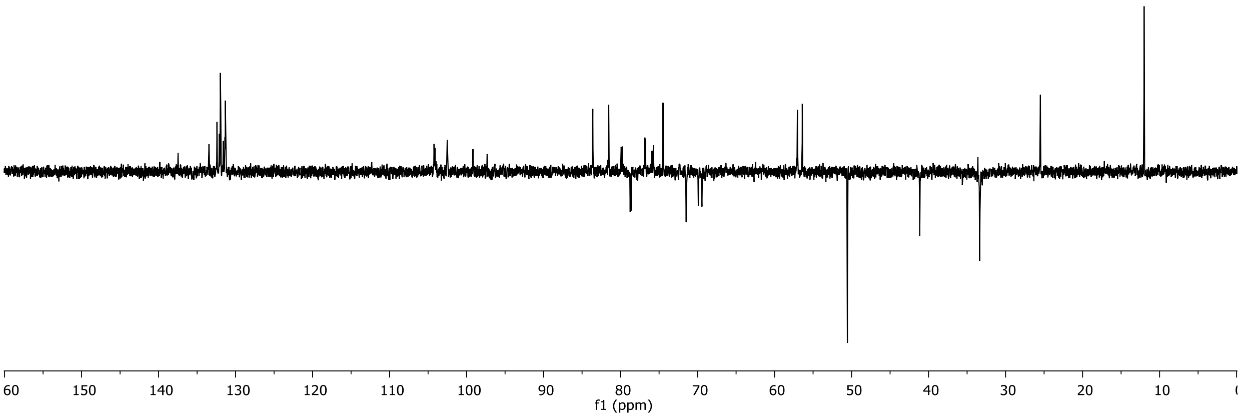
f1 (ppm)

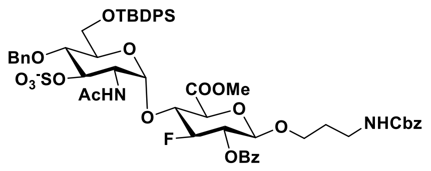
OTBDPS
BnO
O_3^-SO
AcHN
O
COOMe
O
O
F
OBz
O
NHCbz

19

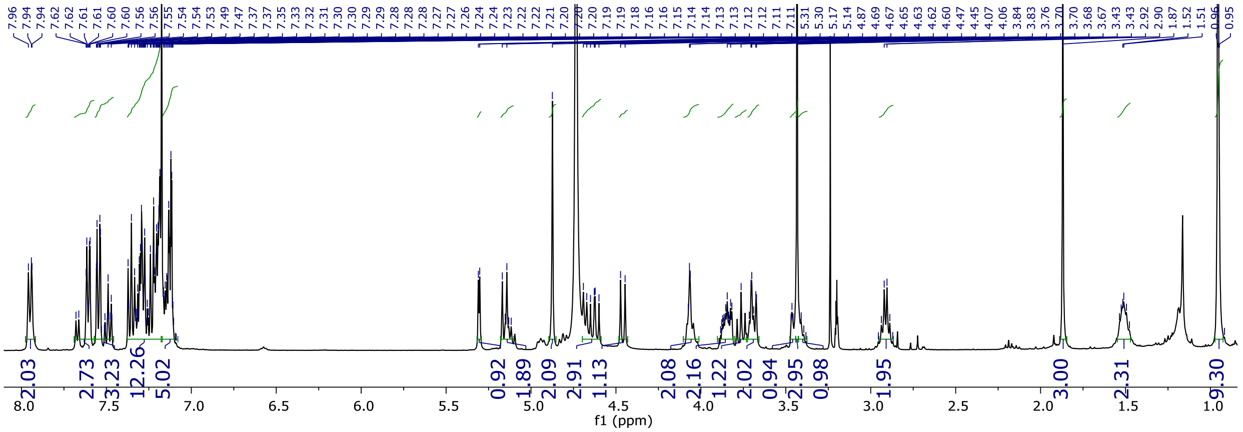
7.96 7.94 7.94 7.62 7.62 7.61 7.61 7.60 7.60 7.56 7.55 7.54 7.54 7.53 7.49 7.47 7.37 7.37 7.35 7.33 7.32 7.31 7.30 7.30 7.29 7.29 7.28 7.28 7.28 7.27 7.27 7.26 7.24 7.24 7.23 7.22 7.22 7.21 7.20 7.20 7.20 7.19 7.19 7.18 7.16 7.15 7.14 7.14 7.13 7.13 7.12 7.12 7.11 7.11 5.31 5.30 5.17 5.14 4.87 4.69 4.67 4.65 4.63 4.62 4.60 4.47 4.45 4.07 4.06 3.84 3.83 3.76 3.70 3.70 3.68 3.67 3.43 3.43 2.92 2.90 1.87 1.52 1.51 0.96 0.95
2.03 2.73 3.23 12.26 5.02 0.92 1.89 2.09 2.91 1.13 2.08 2.16 1.22 2.02 0.94 2.95 0.98 1.95 3.00 2.31 9.30
8.0 7.5 7.0 6.5 6.0 5.5 5.0 4.5 4.0 3.5 3.0 2.5 2.0 1.5 1.0
f1 (ppm)

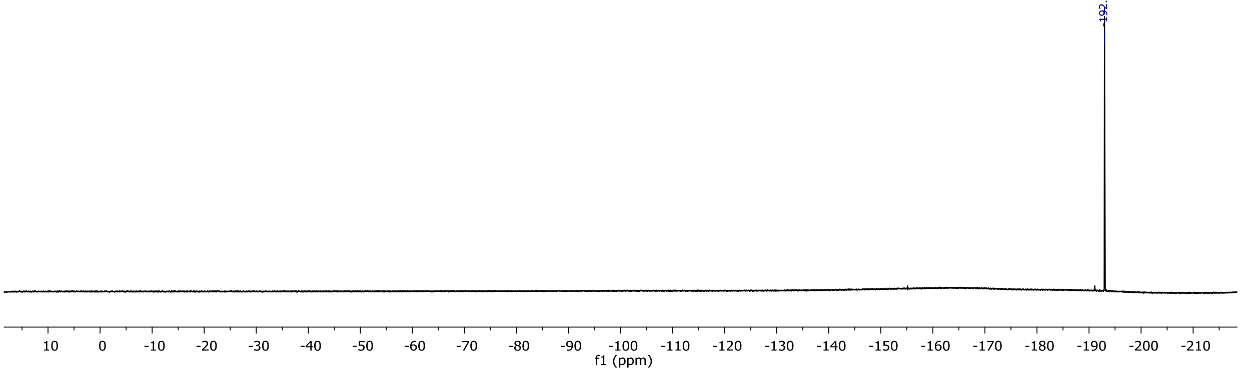
-192.97
10 0 -10 -20 -30 -40 -50 -60 -70 -80 -90 -100 -110 -120 -130 -140 -150 -160 -170 -180 -190 -200 -210
f1 (ppm)

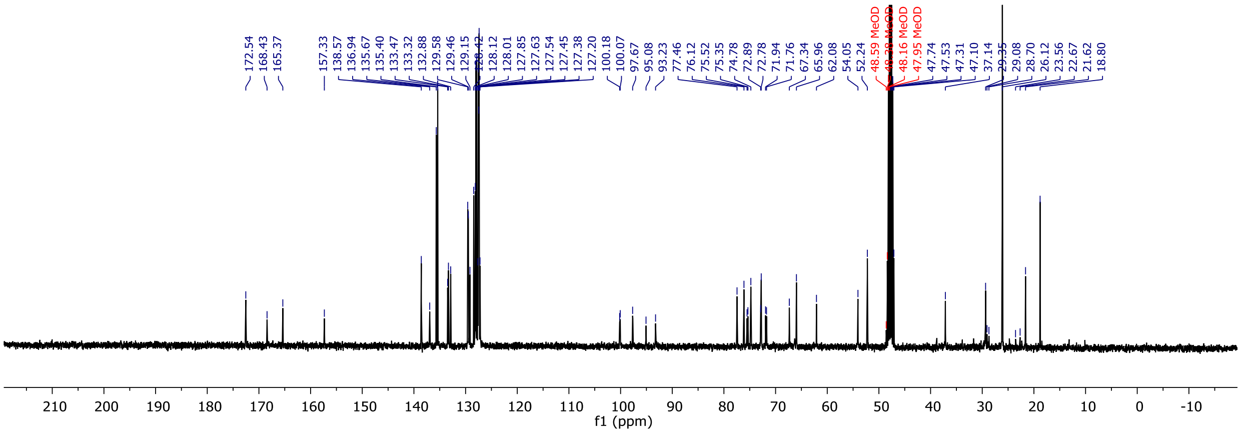
172.54
168.43
165.37
157.33
138.57
136.94
135.67
135.40
133.47
133.32
132.88
129.58
129.46
129.15
128.42
128.12
128.01
127.85
127.63
127.54
127.45
127.38
127.20
100.18
100.07
97.67
95.08
93.23
77.46
76.12
75.52
75.35
74.78
72.89
72.78
71.94
71.76
67.34
65.96
62.08
54.05
52.24
48.59 MeOD
48.38 MeOD
48.16 MeOD
47.95 MeOD
47.74
47.53
47.31
47.10
37.14
29.35
29.08
28.70
26.12
23.56
22.67
21.62
18.80
f1 (ppm)

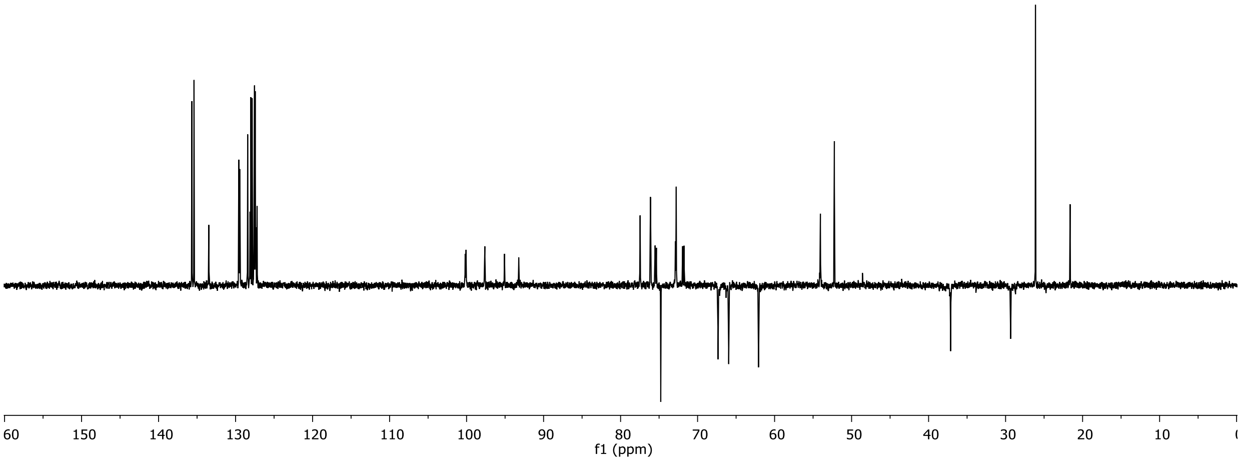
f1 (ppm)

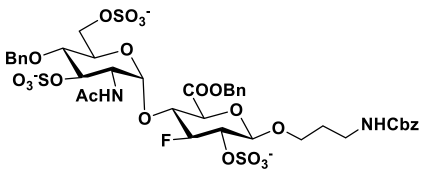
OSO3-
BnO
O3-SO
AcHN
COOBn
NHCbz
F
OSO3-

20

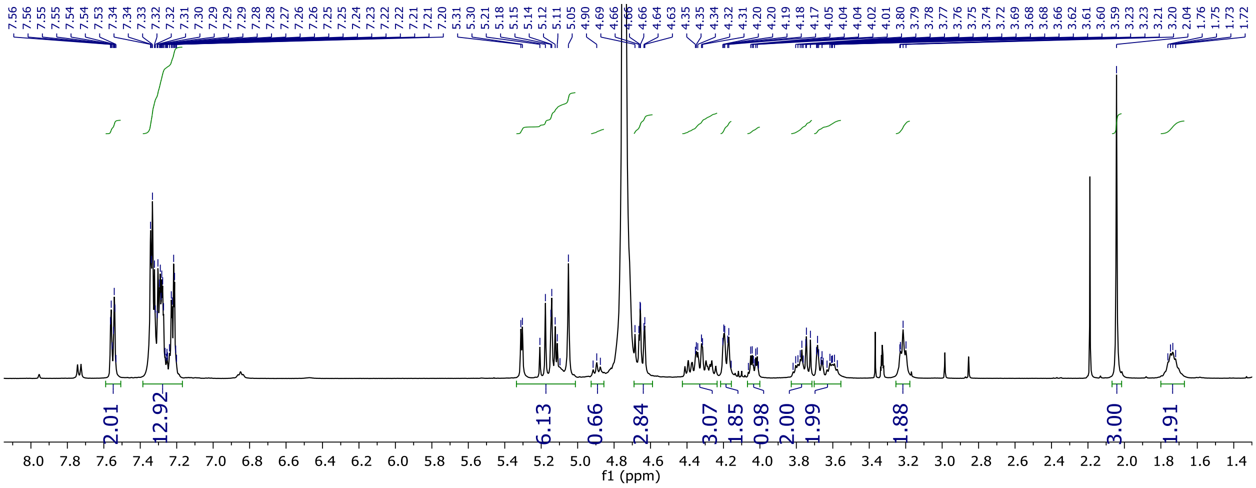
2.01
12.92
6.13
0.66
2.84
3.07
1.85
0.98
2.00
1.99
1.88
3.00
1.91
f1 (ppm)

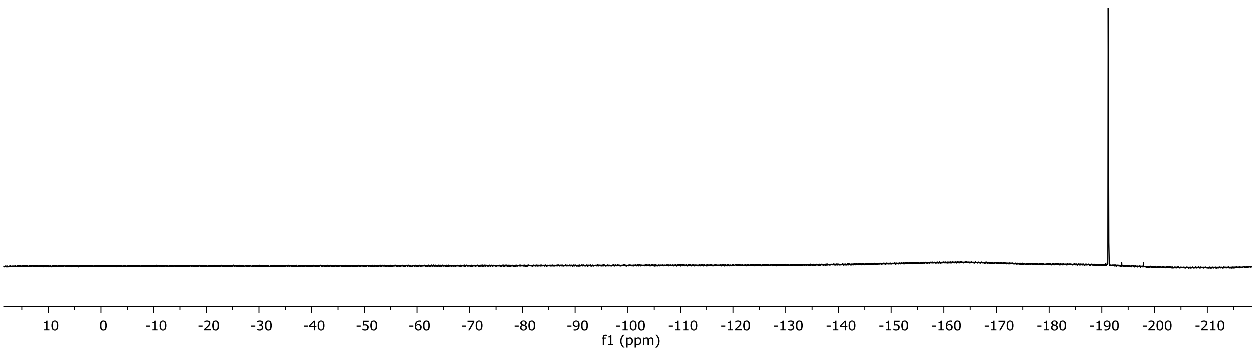
f1 (ppm)

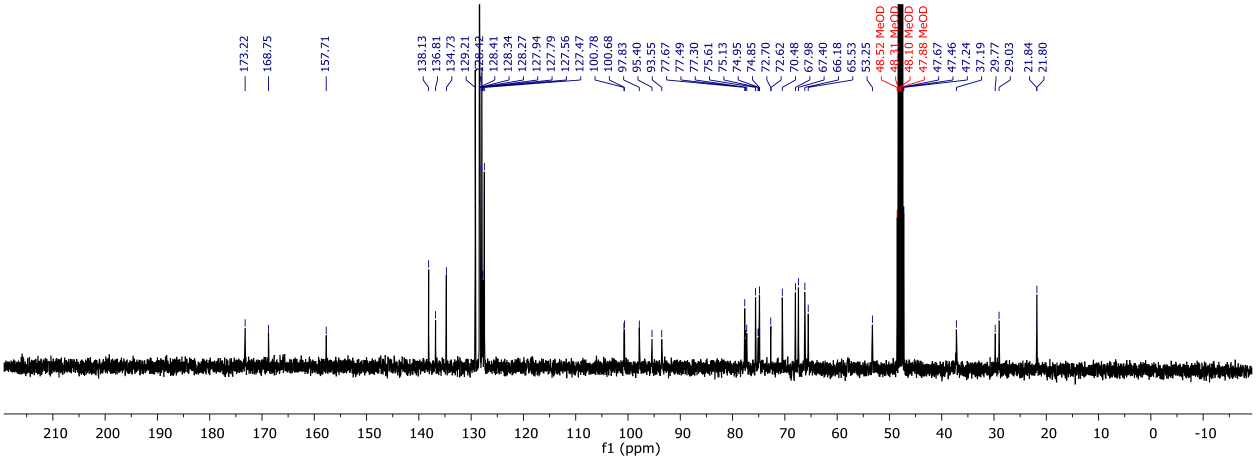

173.22
168.75
157.71
138.13
136.81
134.73
129.21
128.42
128.41
128.34
128.27
127.94
127.79
127.56
127.47
100.78
100.68
97.83
95.40
93.55
77.67
77.49
77.30
75.61
75.13
74.95
74.85
72.70
72.62
70.48
67.98
67.40
66.18
65.53
53.25
48.52 MeOD
48.31 MeOD
48.10 MeOD
47.88 MeOD
47.67
47.46
47.24
37.19
29.77
29.03
21.84
21.80
f1 (ppm)

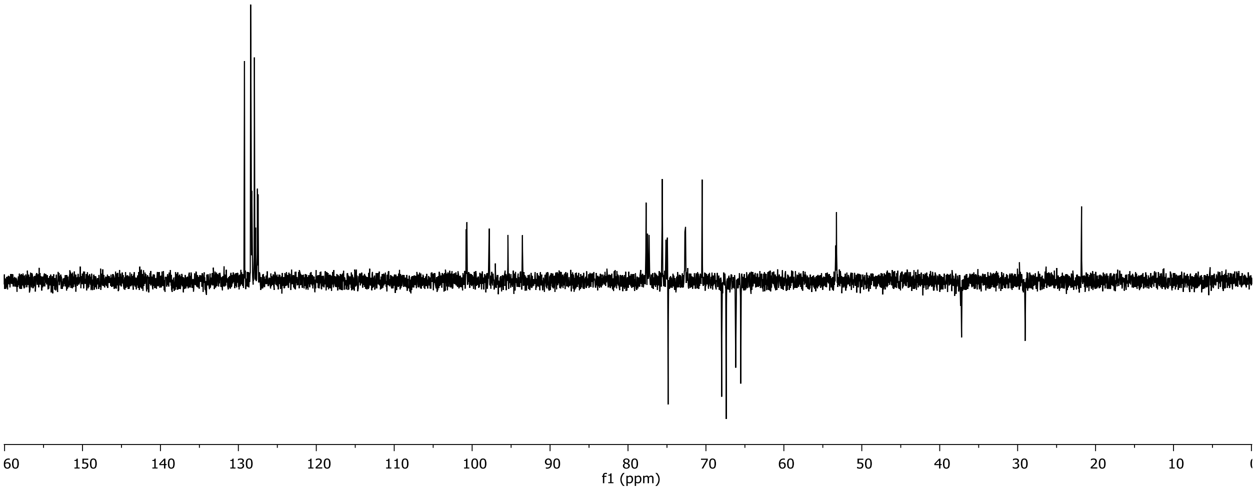

f1 (ppm)

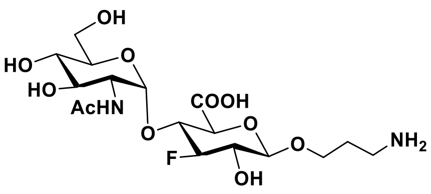
OH
HO
O
HO
AcHN
COOH
O
O
O
F
OH
NH2

HDF-1

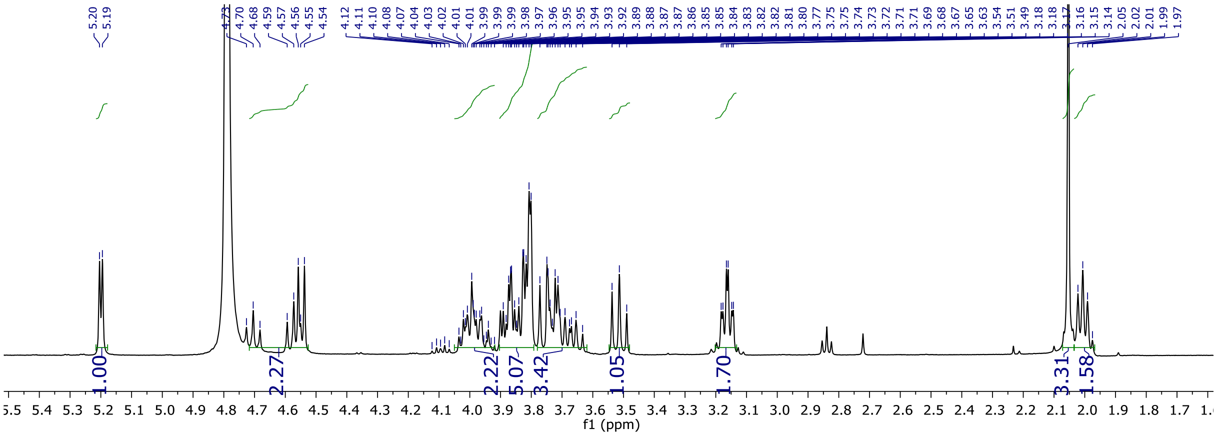
1.00
2.27
2.22
5.07
3.42
1.05
1.70
3.31
1.58
f1 (ppm)

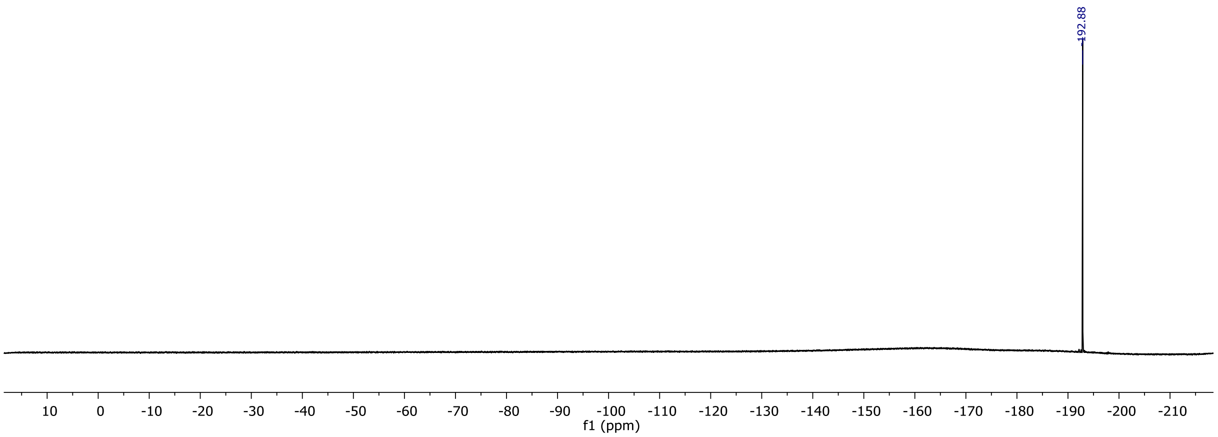
-192.88
f1 (ppm)

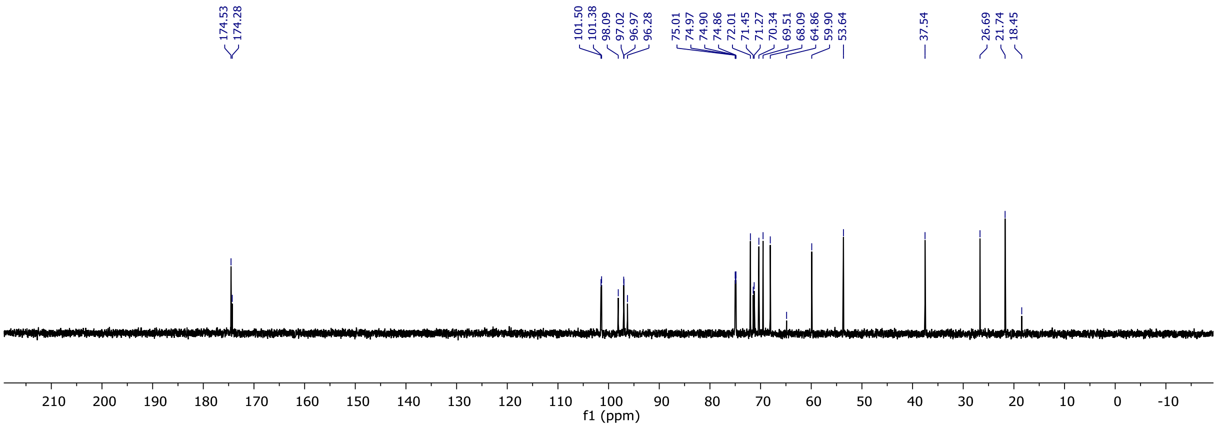
174.53
174.28
101.50
101.38
98.09
97.02
96.97
96.28
75.01
74.97
74.90
74.86
72.01
71.45
71.27
70.34
69.51
68.09
64.86
59.90
53.64
37.54
26.69
21.74
18.45
210 200 190 180 170 160 150 140 130 120 110 100 90 80 70 60 50 40 30 20 10 0 -10
f1 (ppm)

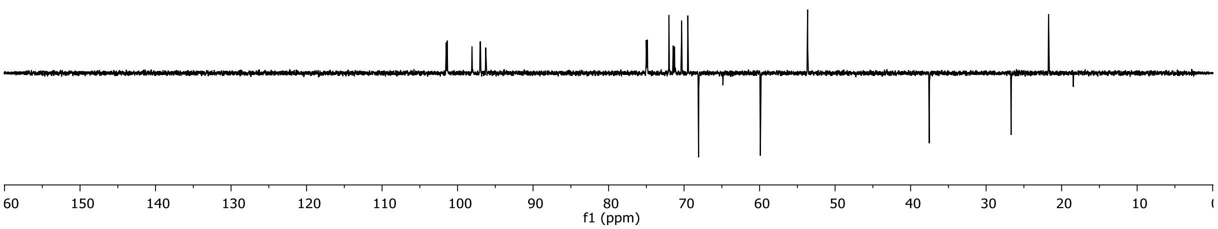
150 140 130 120 110 100 90 80 70 60 50 40 30 20 10
f1 (ppm)

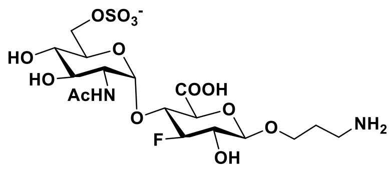
OSO3-
HO
O
HO
AcHN
O
COOH
O
O
NH2
F
OH

HDF-2

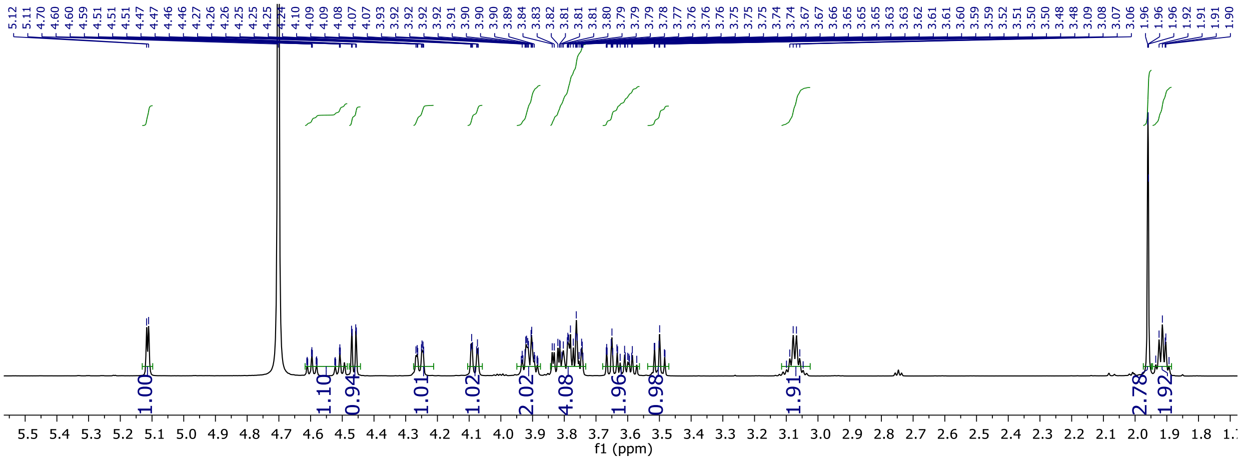
5.12
5.11
4.70
4.60
4.60
4.59
4.51
4.51
4.51
4.51
4.47
4.47
4.46
4.46
4.27
4.26
4.26
4.25
4.25
4.25
4.24
4.10
4.09
4.09
4.08
4.07
4.07
3.93
3.92
3.92
3.92
3.92
3.91
3.90
3.90
3.90
3.89
3.84
3.83
3.82
3.81
3.81
3.81
3.80
3.79
3.79
3.79
3.78
3.77
3.76
3.76
3.76
3.75
3.75
3.75
3.74
3.74
3.67
3.67
3.66
3.65
3.65
3.63
3.63
3.62
3.61
3.61
3.60
3.59
3.59
3.52
3.51
3.50
3.50
3.48
3.48
3.09
3.08
3.07
3.06
1.96
1.96
1.96
1.92
1.91
1.91
1.90
1.00
1.10
0.94
1.01
1.02
2.02
4.08
1.96
0.98
1.91
2.78
1.92
5.5 5.4 5.3 5.2 5.1 5.0 4.9 4.8 4.7 4.6 4.5 4.4 4.3 4.2 4.1 4.0 3.9 3.8 3.7 3.6 3.5 3.4 3.3 3.2 3.1 3.0 2.9 2.8 2.7 2.6 2.5 2.4 2.3 2.2 2.1 2.0 1.9 1.8
f1 (ppm)

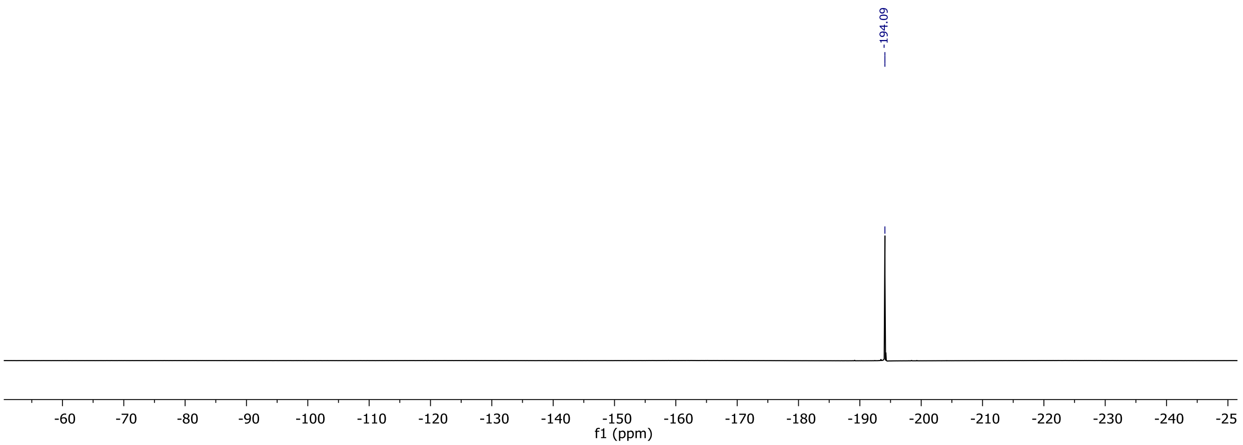
-194.09
-60 -70 -80 -90 -100 -110 -120 -130 -140 -150 -160 -170 -180 -190 -200 -210 -220 -230 -240
f1 (ppm)

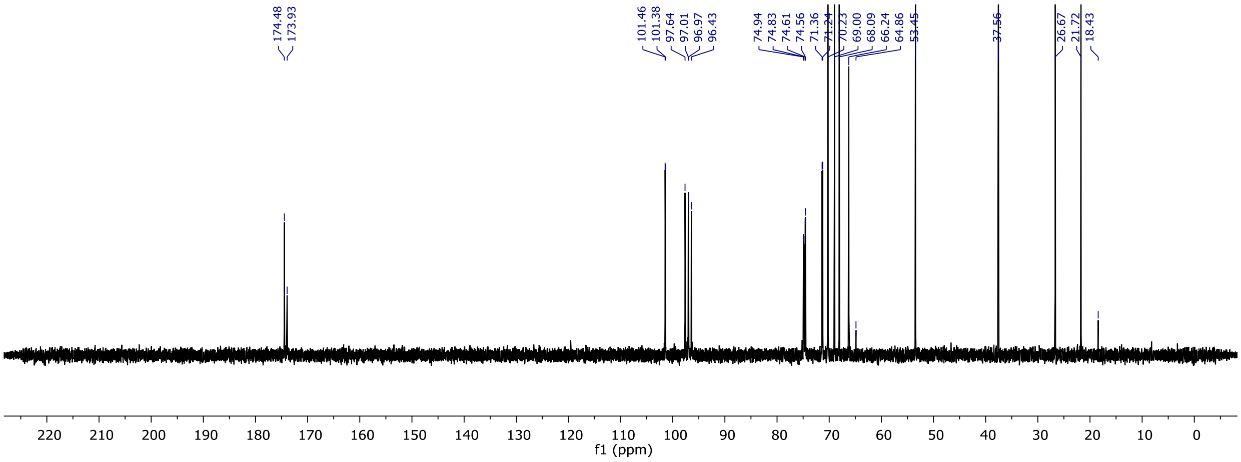

174.48
173.93
101.46
101.38
97.64
97.01
96.97
96.43
74.94
74.83
74.61
74.56
71.36
71.24
70.23
69.00
68.09
66.24
64.86
53.45
37.56
26.67
21.72
18.43
220 210 200 190 180 170 160 150 140 130 120 110 100 90 80 70 60 50 40 30 20 10 0
f1 (ppm)

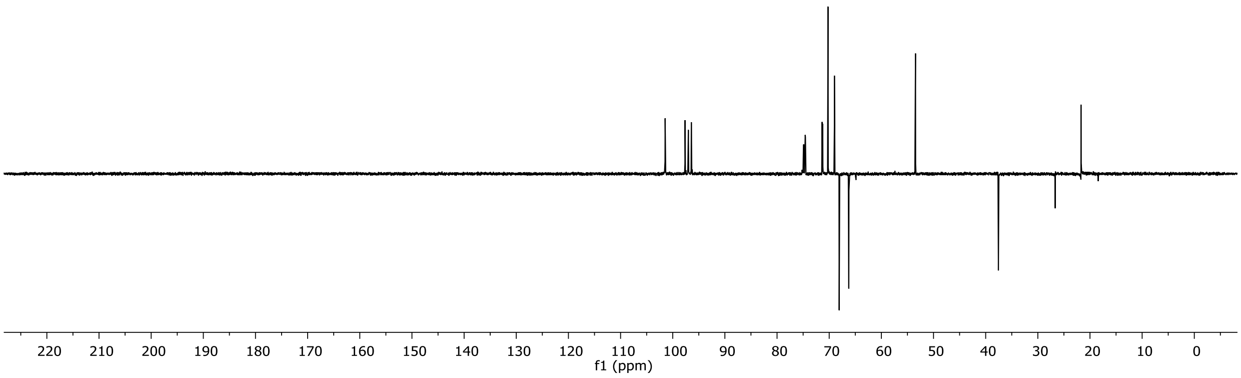

220 210 200 190 180 170 160 150 140 130 120 110 100 90 80 70 60 50 40 30 20 10 0
f1 (ppm)

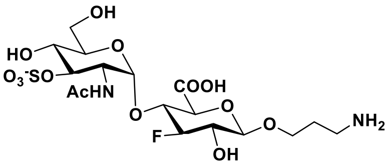

HDF-3

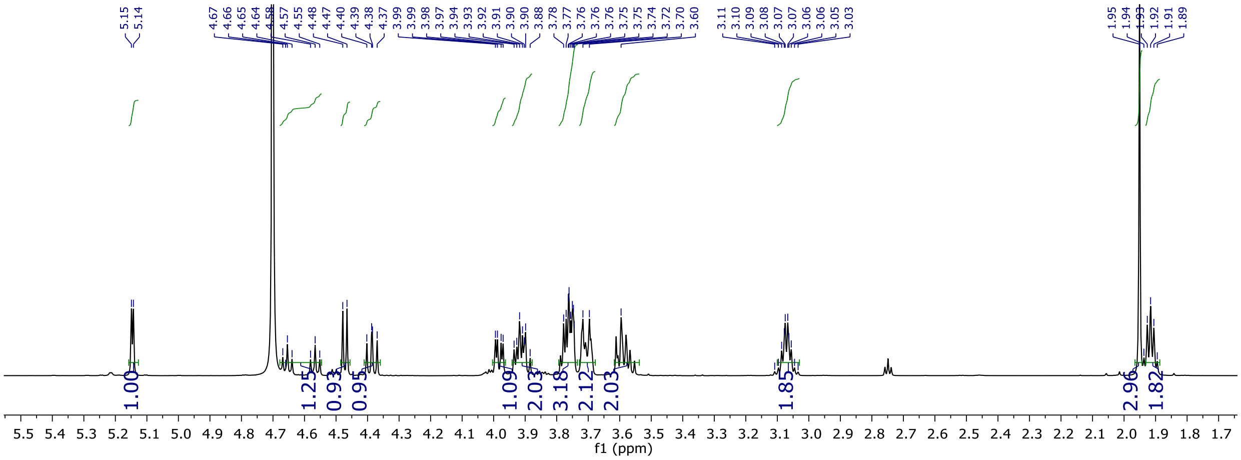

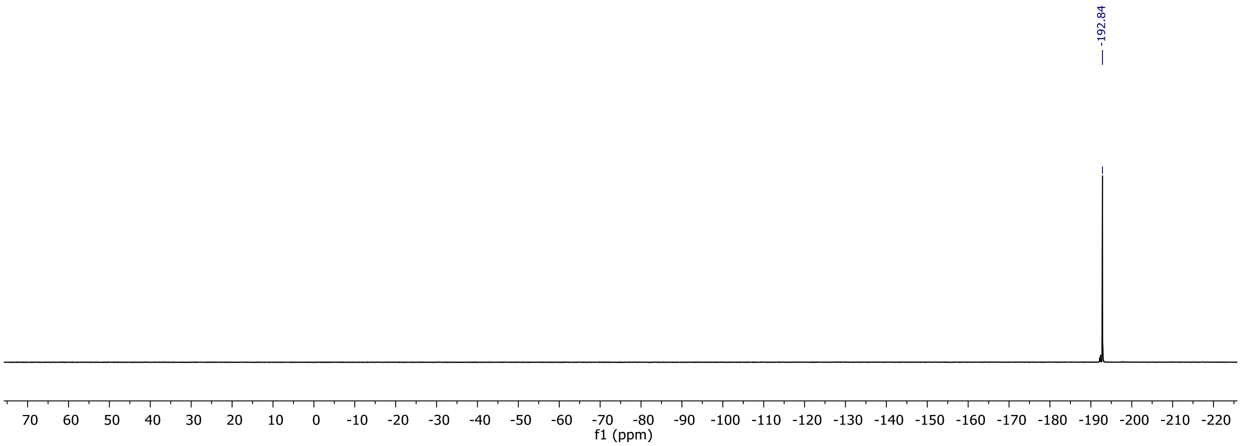

-192.84
70 60 50 40 30 20 10 0 -10 -20 -30 -40 -50 -60 -70 -80 -90 -100 -110 -120 -130 -140 -150 -160 -170 -180 -190 -200 -210 -220
f1 (ppm)

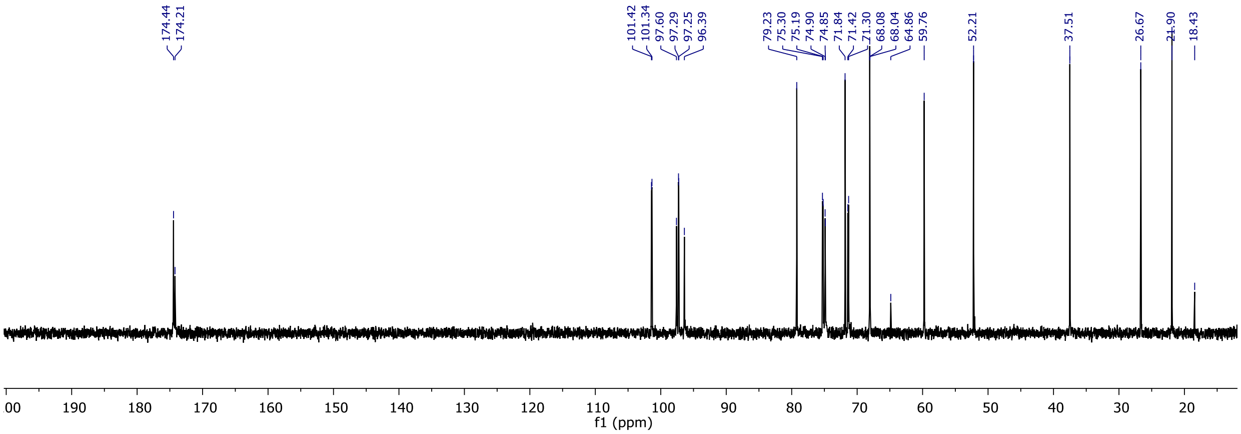

174.44
174.21
101.42
101.34
97.60
97.29
97.25
96.39
79.23
75.30
75.19
74.90
74.85
71.84
71.42
71.30
68.08
68.04
64.86
59.76
52.21
37.51
26.67
21.90
18.43
00 190 180 170 160 150 140 130 120 110 100 90 80 70 60 50 40 30 20
f1 (ppm)

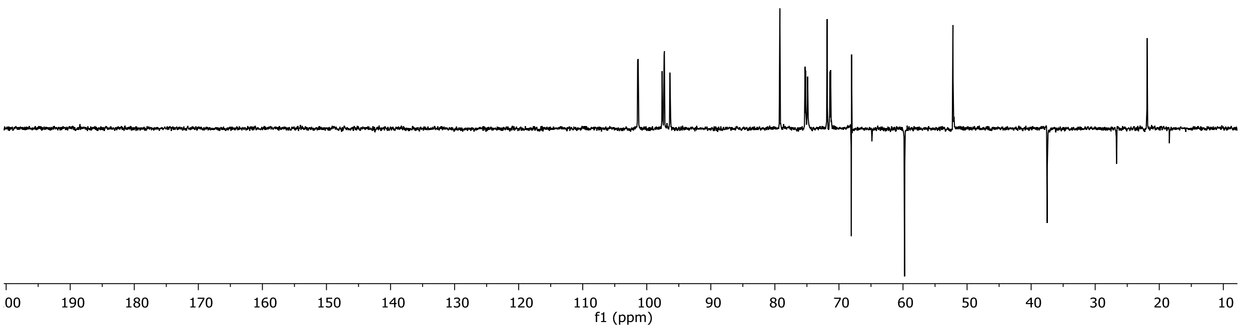

00 190 180 170 160 150 140 130 120 110 100 90 80 70 60 50 40 30 20 10
f1 (ppm)

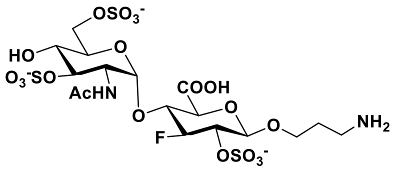

HDF-4

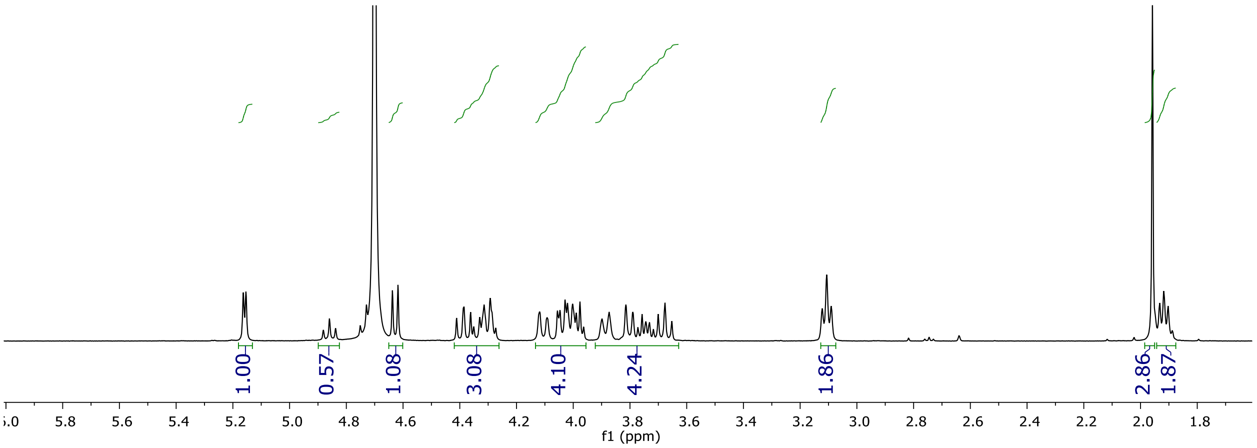

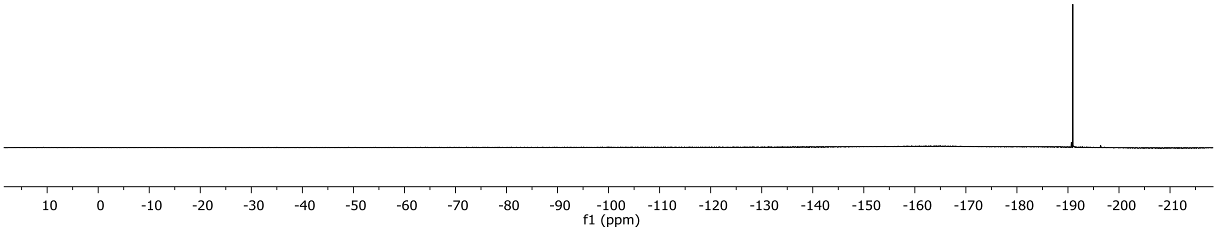

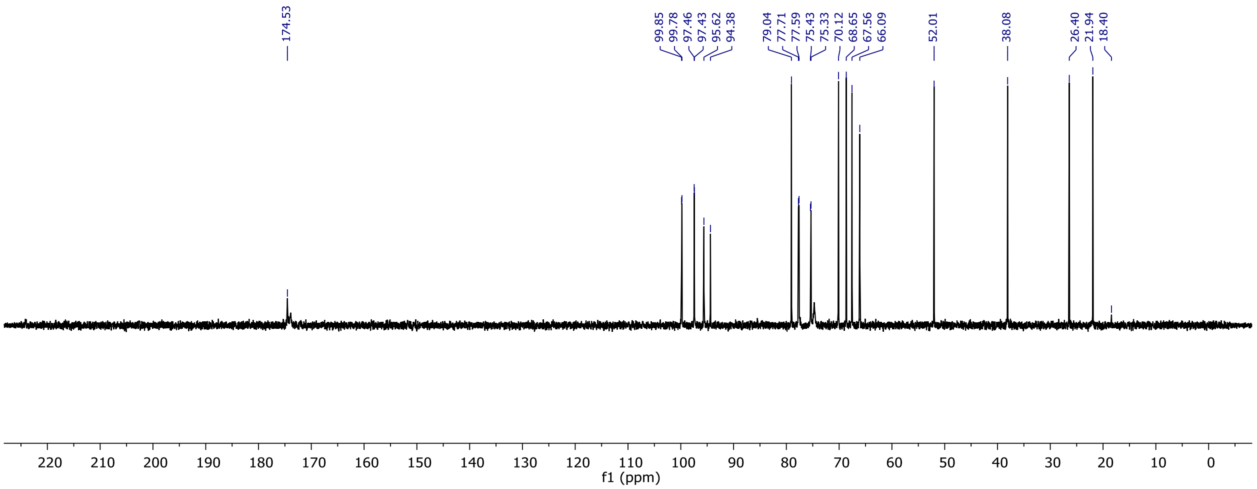
174.53
99.85
99.78
97.46
97.43
95.62
94.38
79.04
77.71
77.59
75.43
75.33
70.12
68.65
67.56
66.09
52.01
38.08
26.40
21.94
18.40
220 210 200 190 180 170 160 150 140 130 120 110 100 90 80 70 60 50 40 30 20 10 0
f1 (ppm)

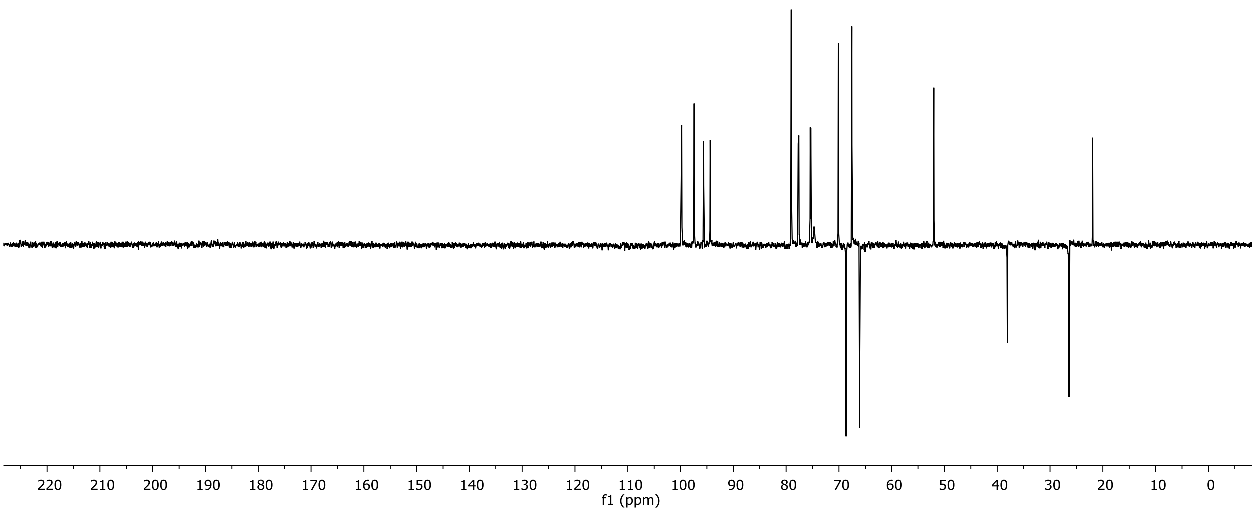
220 210 200 190 180 170 160 150 140 130 120 110 100 90 80 70 60 50 40 30 20 10 0
f1 (ppm)

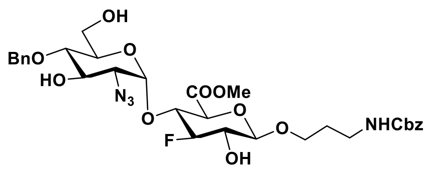

21

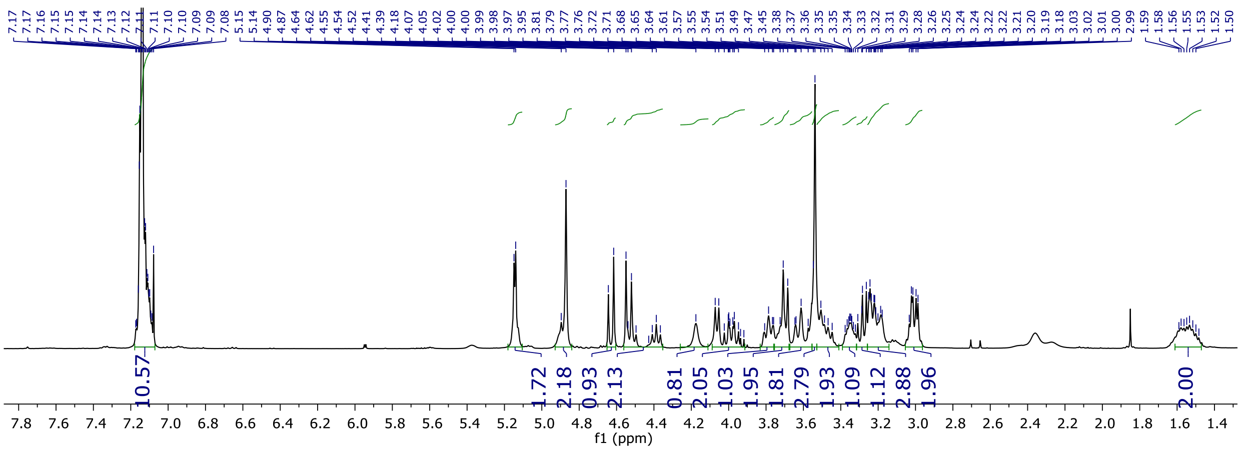

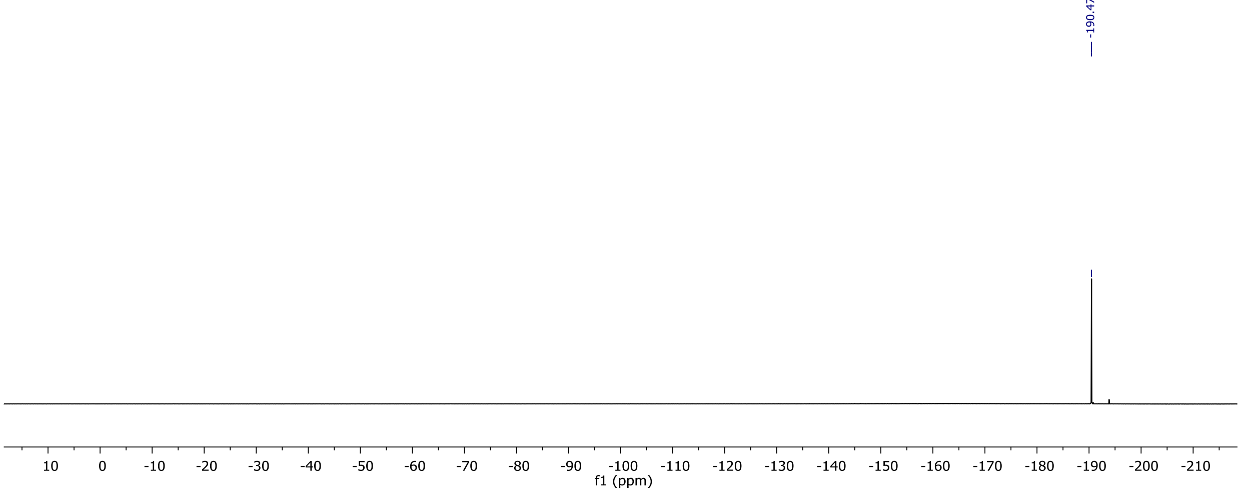

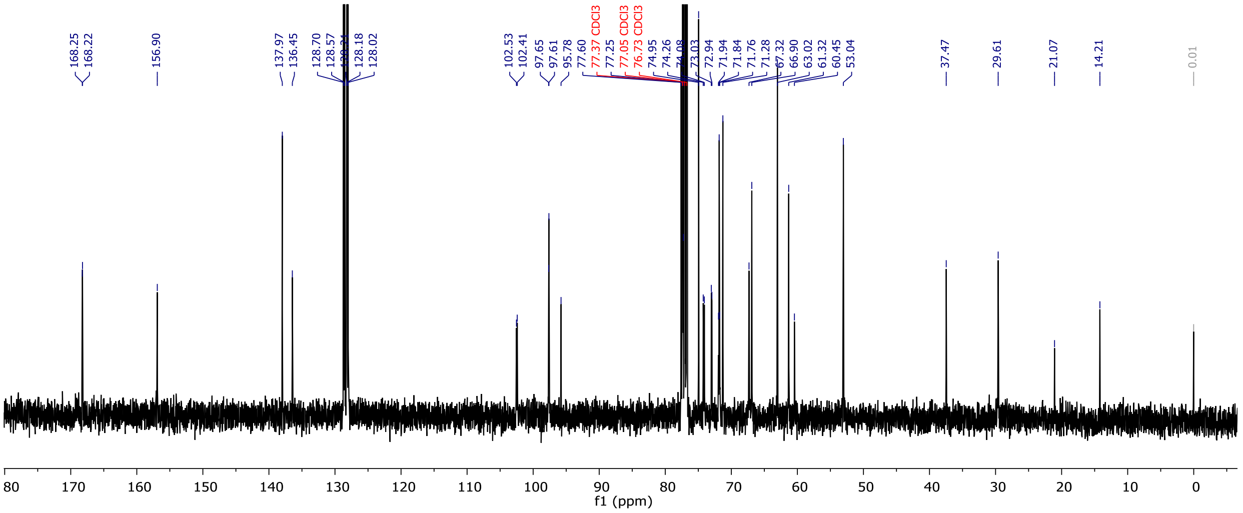

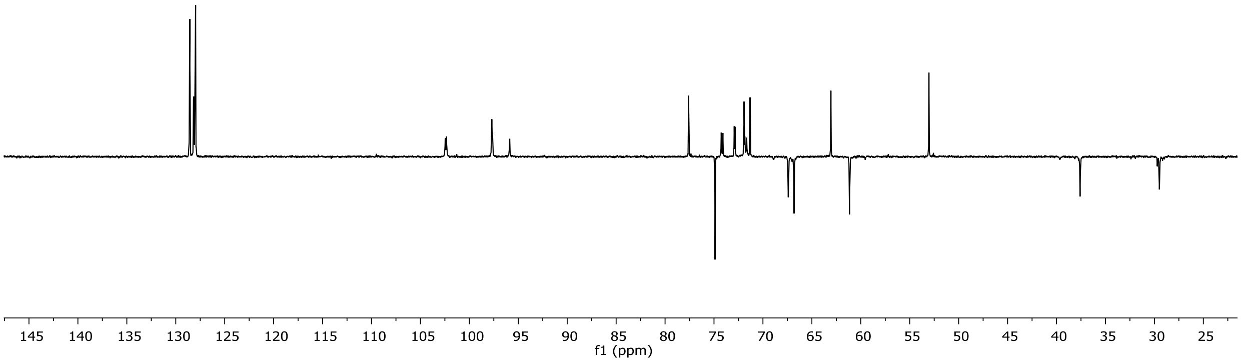

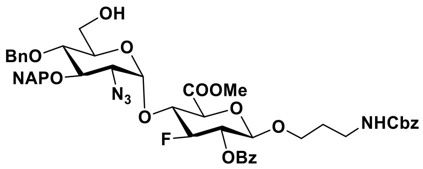

22

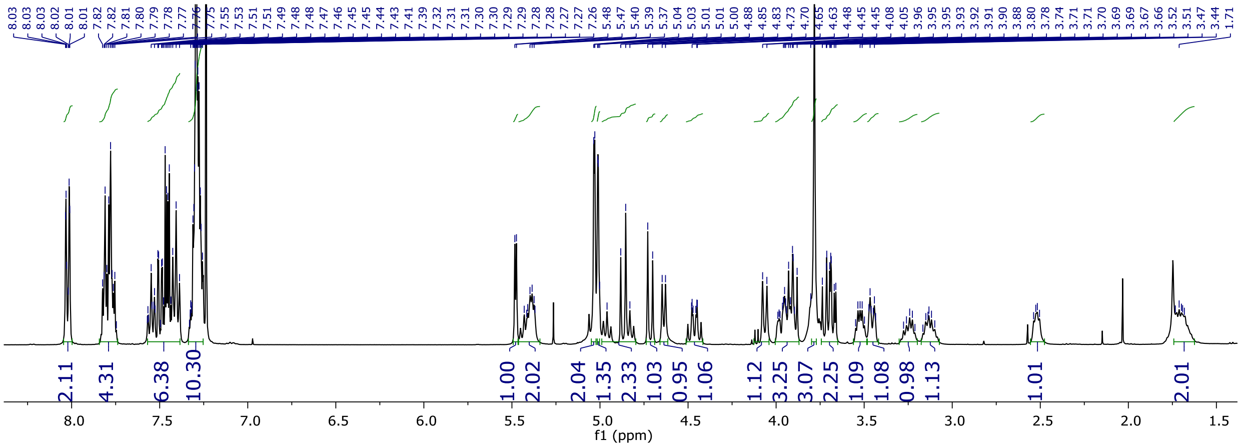

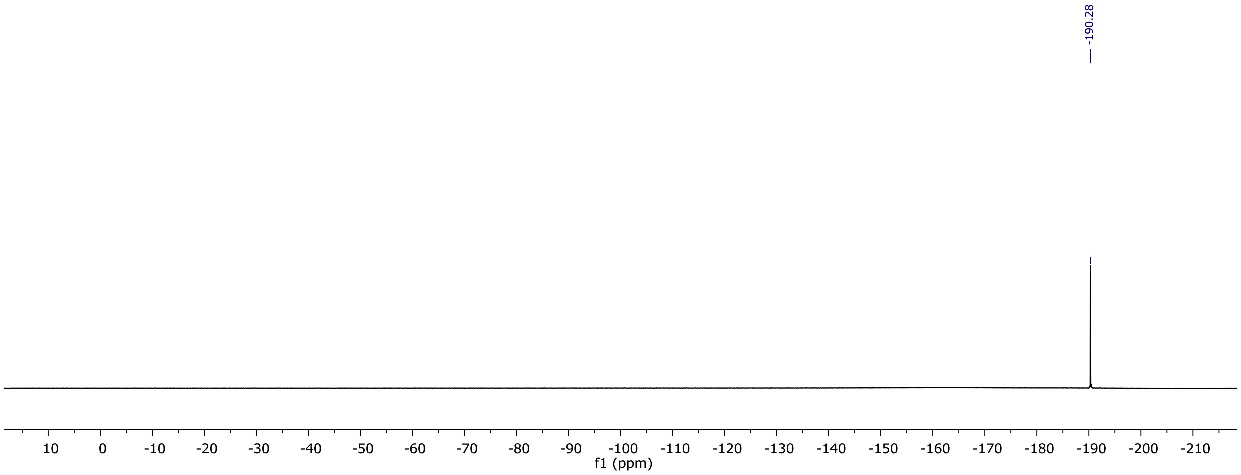

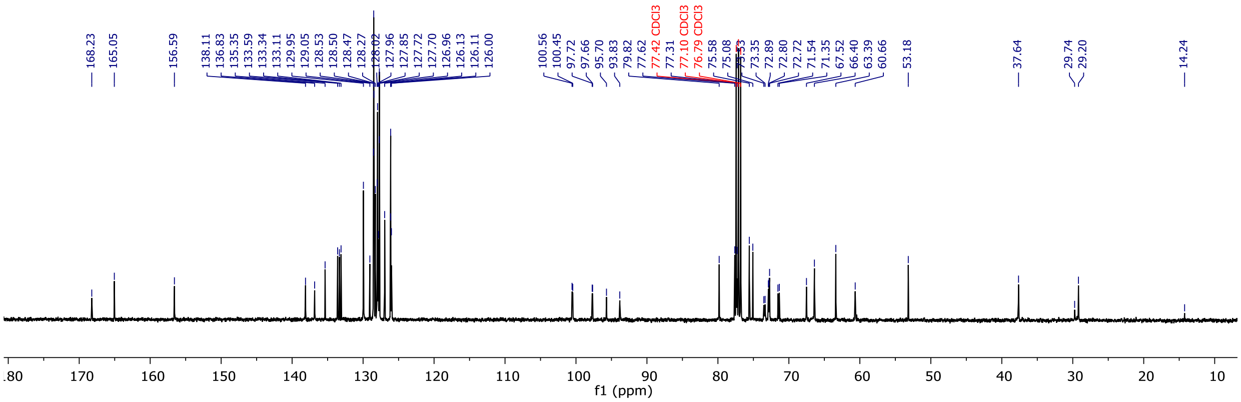
168.23
165.05
156.59
138.11
136.83
135.35
133.59
133.34
133.11
129.95
129.05
128.53
128.50
128.47
128.27
128.02
127.96
127.85
127.72
127.70
126.96
126.13
126.11
126.00
100.56
100.45
97.72
97.66
95.70
93.83
79.82
77.62
77.42 CDCl3
77.31
77.10 CDCl3
76.79 CDCl3
75.58
75.08
73.35
72.89
72.80
72.72
71.54
71.35
67.52
66.40
63.39
60.66
53.18
37.64
29.74
29.20
14.24
f1 (ppm)

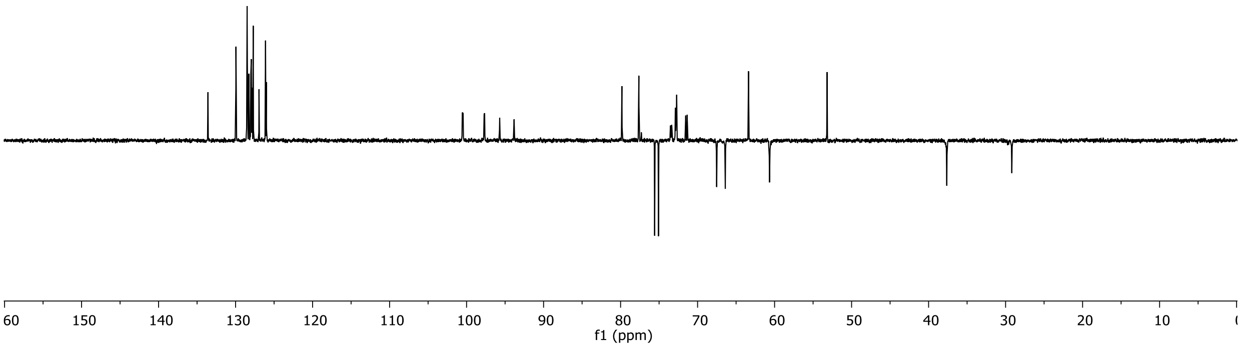
f1 (ppm)

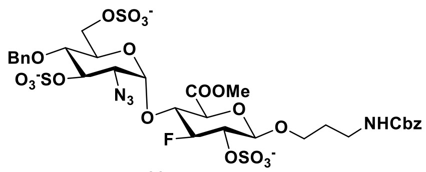

23

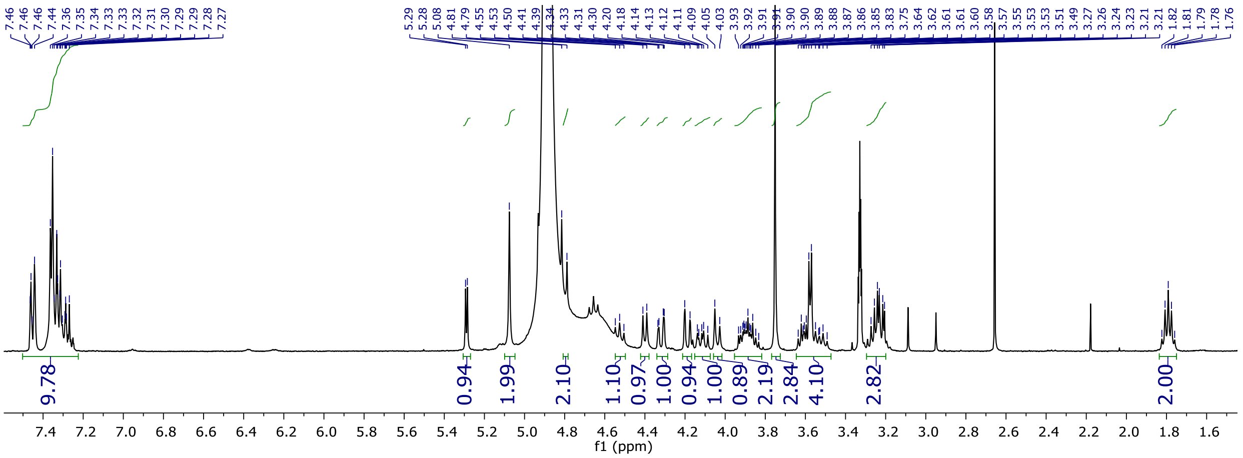

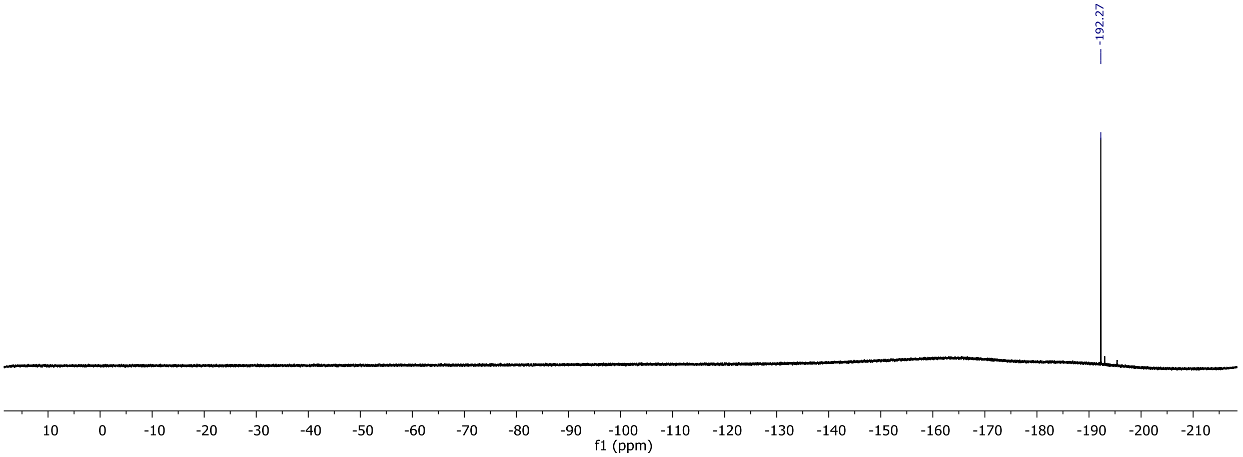

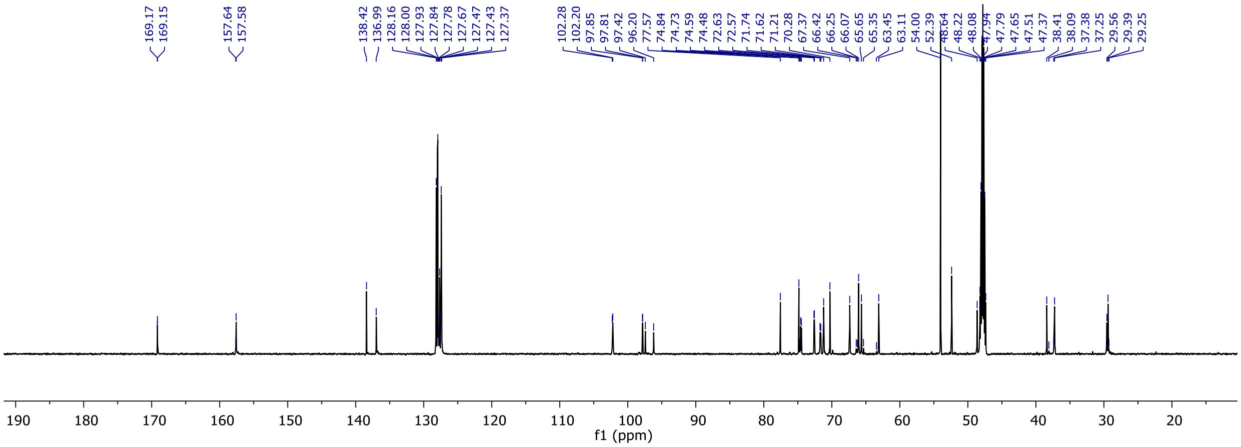
169.17
169.15
157.64
157.58
138.42
136.99
128.16
128.00
127.93
127.84
127.78
127.67
127.47
127.43
127.37
102.28
102.20
97.85
97.81
97.42
96.20
77.57
74.84
74.73
74.59
74.48
72.63
72.57
71.74
71.62
71.21
70.28
67.37
66.42
66.25
66.07
65.65
65.35
63.45
63.11
54.00
52.39
48.64
48.22
48.08
47.94
47.79
47.65
47.51
47.37
38.41
38.09
37.38
37.25
29.56
29.39
29.25
f1 (ppm)

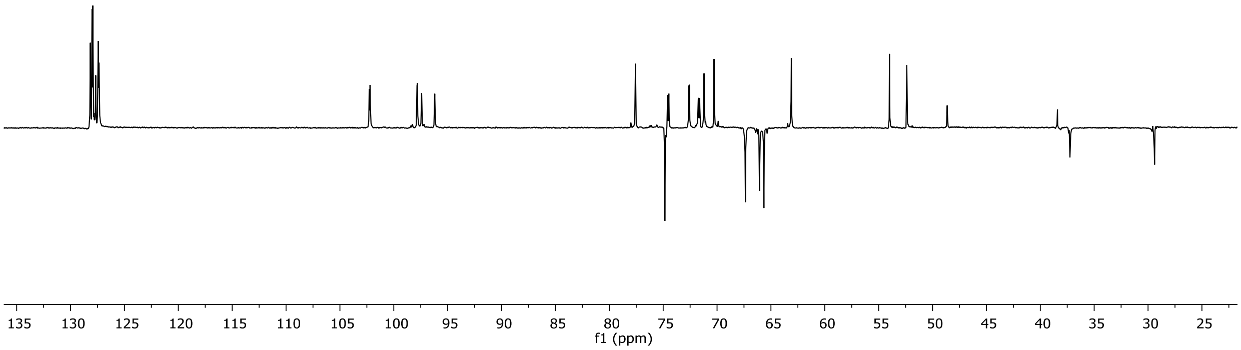
f1 (ppm)

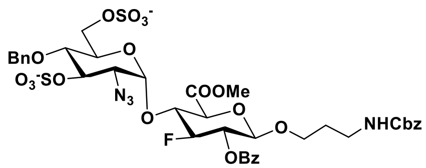
OSO3-
BnO
O3-SO
N3
COOMe
O
F
OBz
NHCbz

24

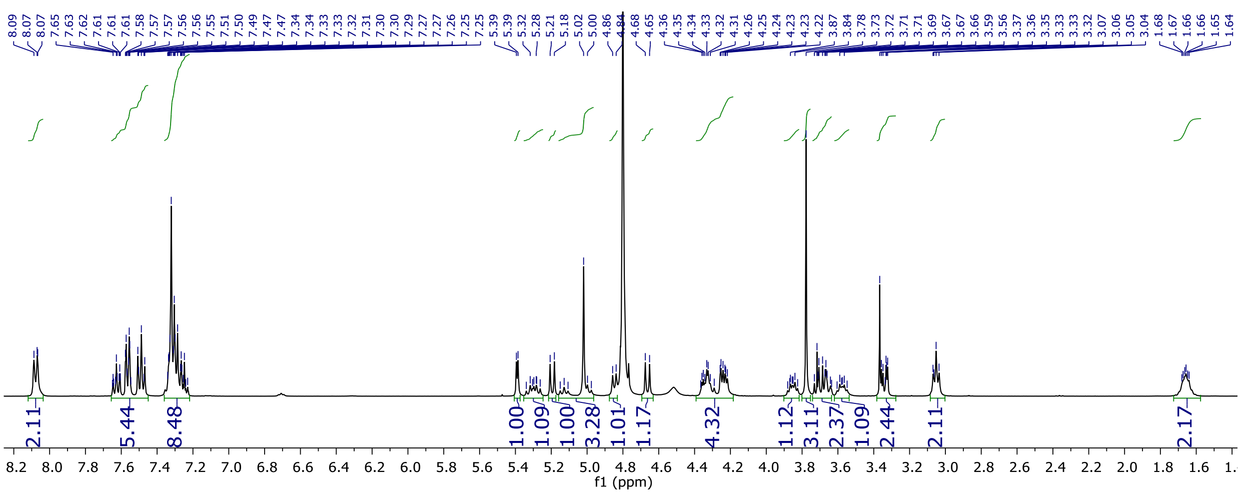
2.11
5.44
8.48
1.00
1.09
1.00
3.28
1.01
1.17
4.32
1.12
3.11
2.37
1.09
2.44
2.11
2.17
f1 (ppm)

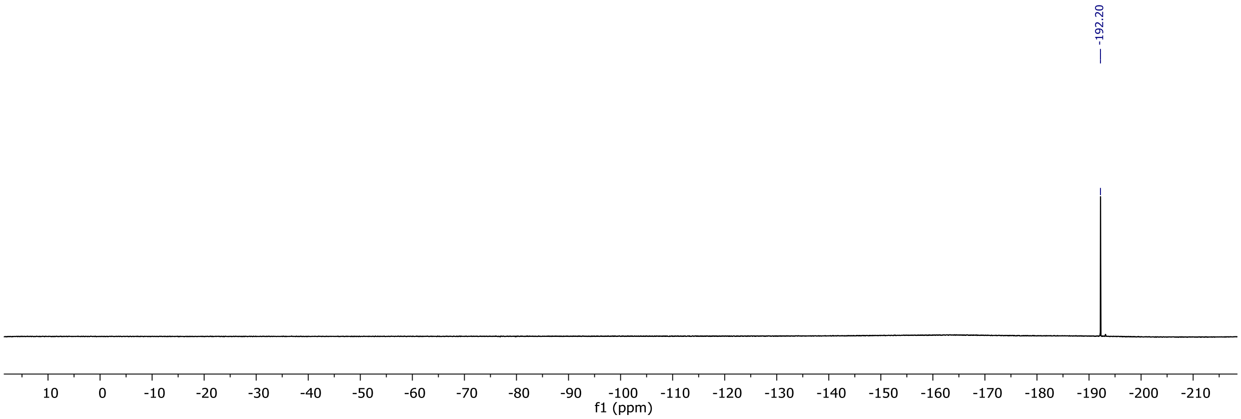
-192.20
f1 (ppm)

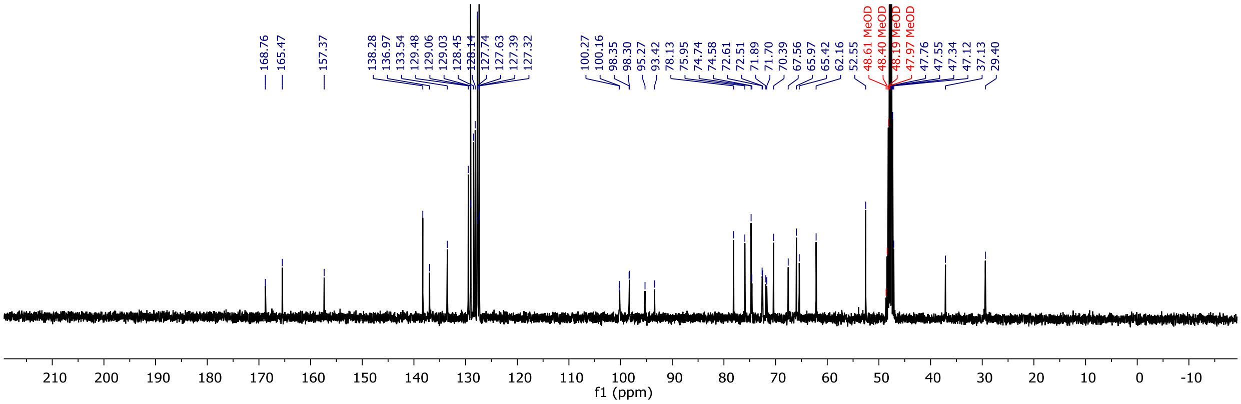

168.76
165.47
157.37
138.28
136.97
133.54
129.48
129.06
129.03
128.45
128.14
127.74
127.63
127.39
127.32
100.27
100.16
98.35
98.30
95.27
93.42
78.13
75.95
74.74
74.58
72.61
72.51
71.89
71.70
70.39
67.56
65.97
65.42
62.16
52.55
48.61 MeOD
48.40 MeOD
48.19 MeOD
47.97 MeOD
47.76
47.55
47.34
47.12
37.13
29.40
f1 (ppm)

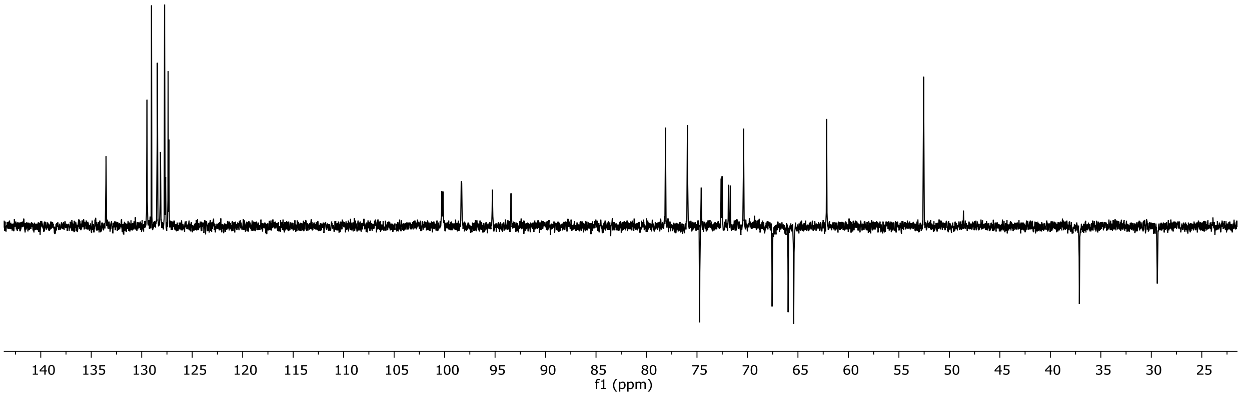

f1 (ppm)

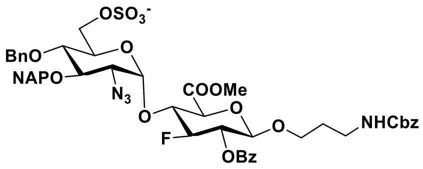
OSO3-
BnO
NAPO
O
N3
COOMe
O
O
F
OBz
O
NHCbz

25

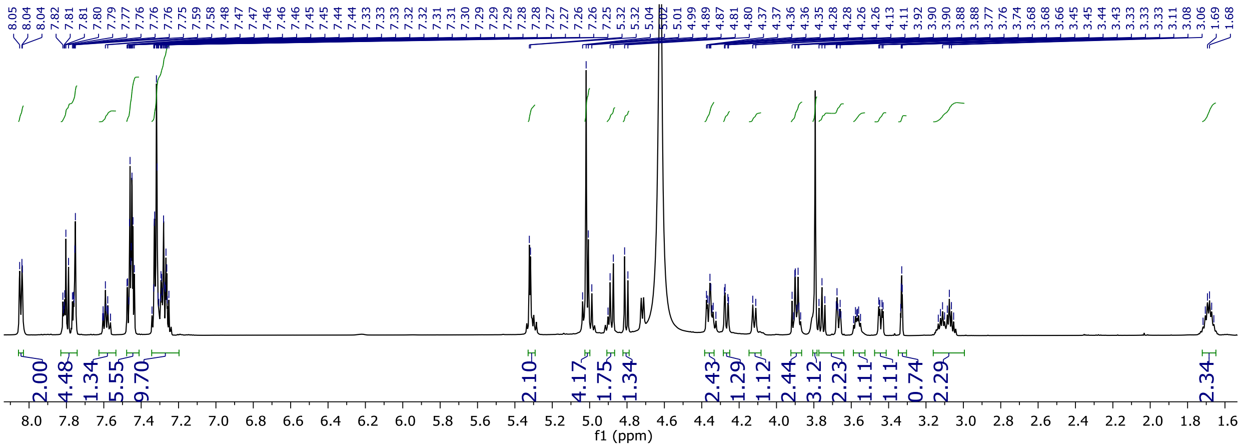
2.00
4.48
1.34
5.55
9.70
2.10
4.17
1.75
1.34
2.43
1.29
1.12
2.44
3.12
2.23
1.11
1.11
0.74
2.29
2.34
f1 (ppm)

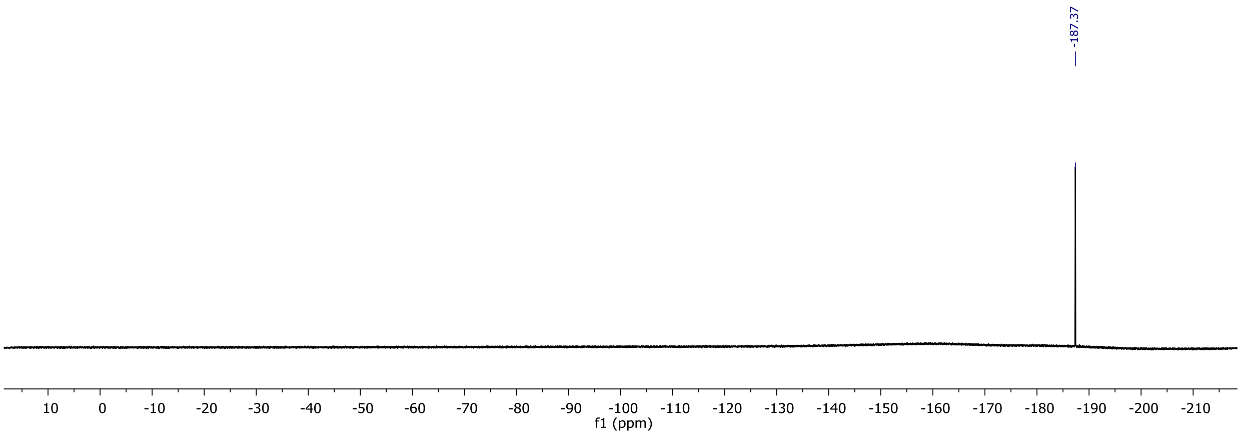
-187.37
f1 (ppm)

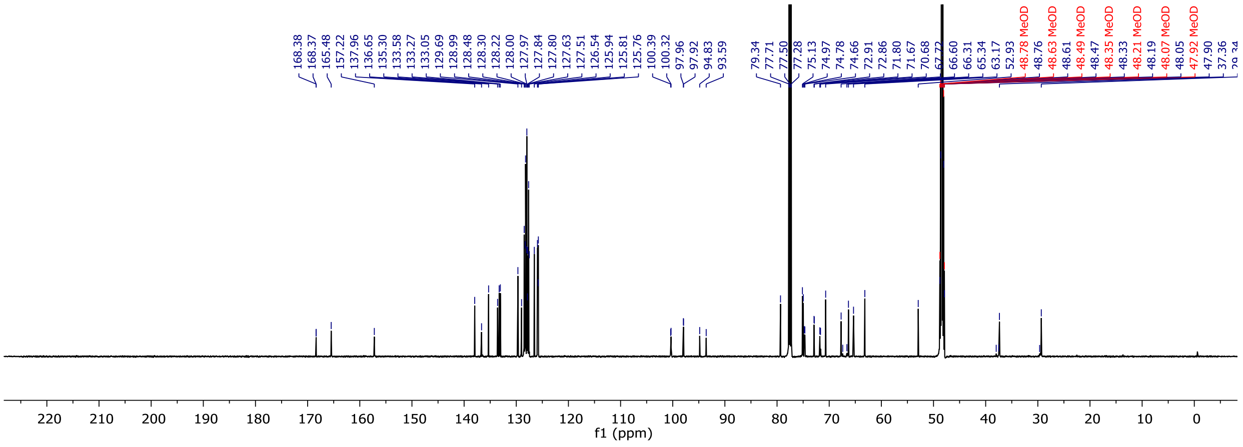
168.38
168.37
165.48
157.22
137.96
136.65
135.30
133.58
133.27
133.05
129.69
128.99
128.48
128.30
128.22
128.00
127.97
127.84
127.80
127.63
127.51
126.54
125.94
125.81
125.76
100.39
100.32
97.96
97.92
94.83
93.59
79.34
77.71
77.50
77.28
75.13
74.97
74.78
74.66
72.91
72.86
71.80
71.67
70.68
67.72
66.60
66.31
65.34
63.17
52.93
48.78 MeOD
48.76
48.63 MeOD
48.61
48.49 MeOD
48.47
48.35 MeOD
48.33
48.21 MeOD
48.19
48.07 MeOD
48.05
47.92 MeOD
47.90
37.36
f1 (ppm)

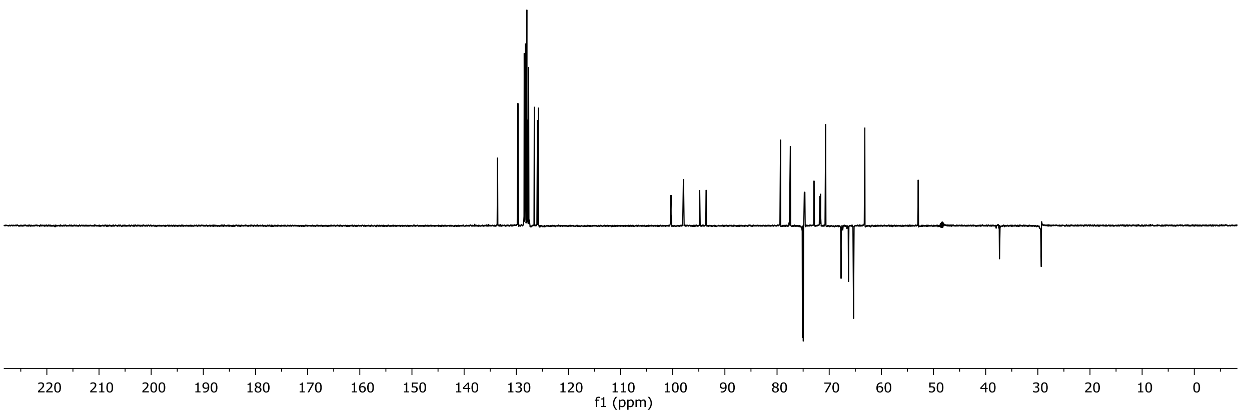
f1 (ppm)

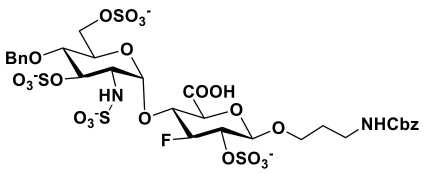

26

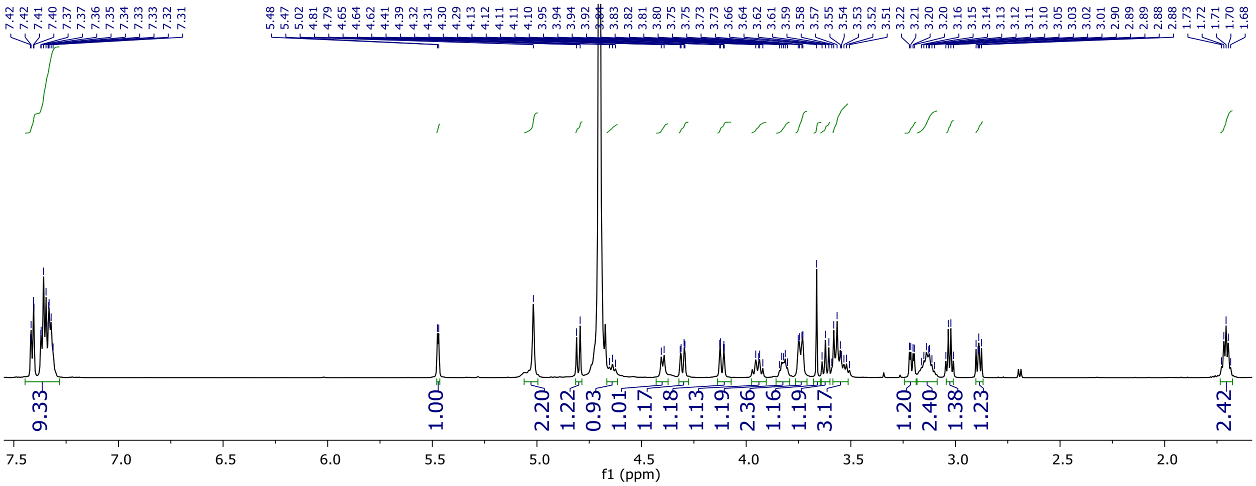

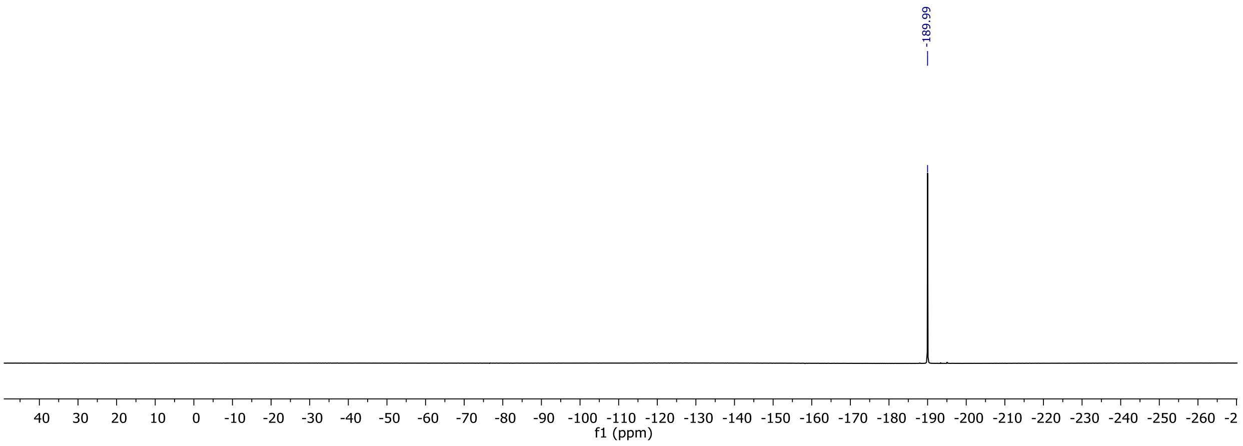

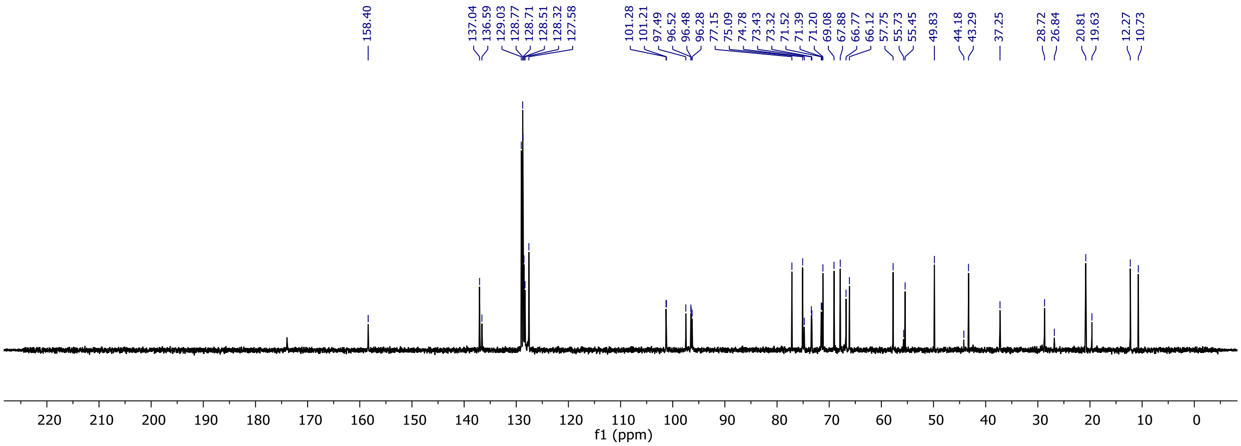

158.40
137.04
136.59
129.03
128.77
128.71
128.51
128.32
127.58
101.28
101.21
97.49
96.52
96.48
96.28
77.15
75.09
74.78
73.43
73.32
71.52
71.39
71.20
69.08
67.88
66.77
66.12
57.75
55.73
55.45
49.83
44.18
43.29
37.25
28.72
26.84
20.81
19.63
12.27
10.73
f1 (ppm)

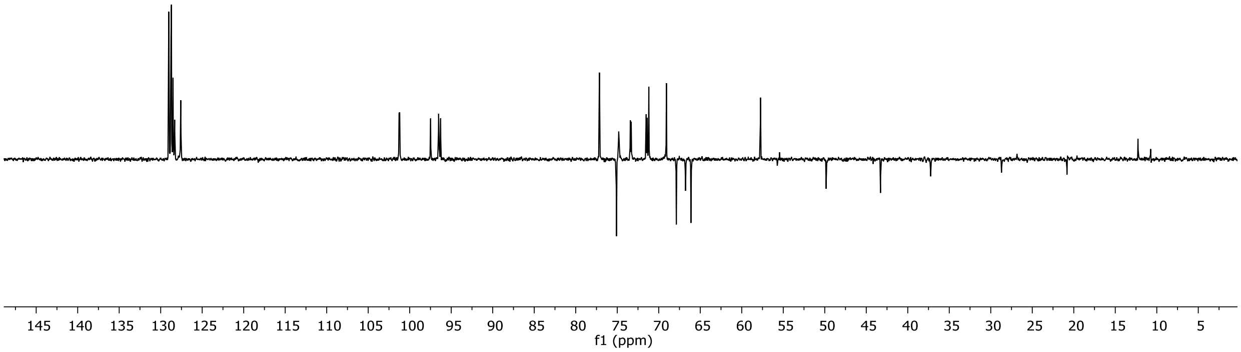

f1 (ppm)

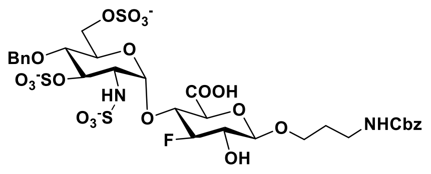

27

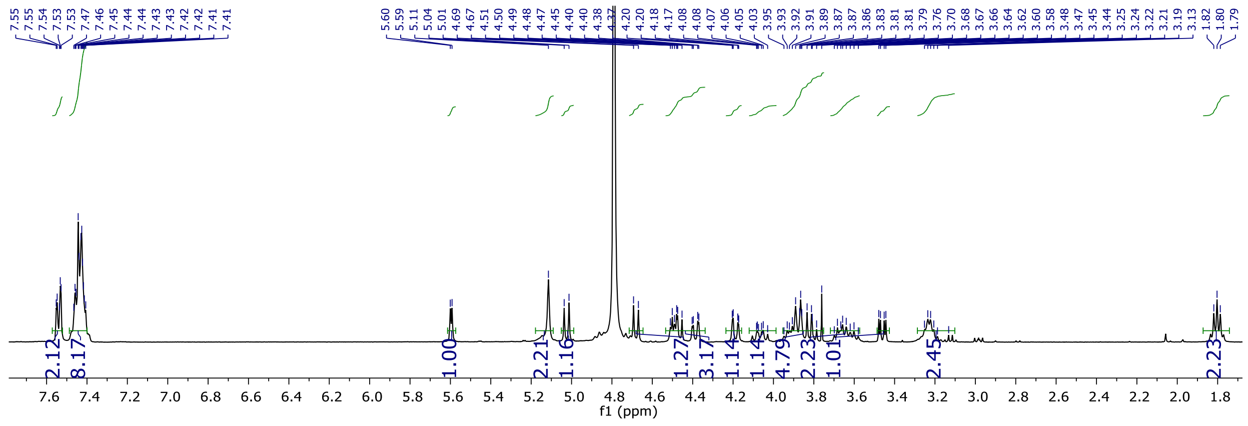

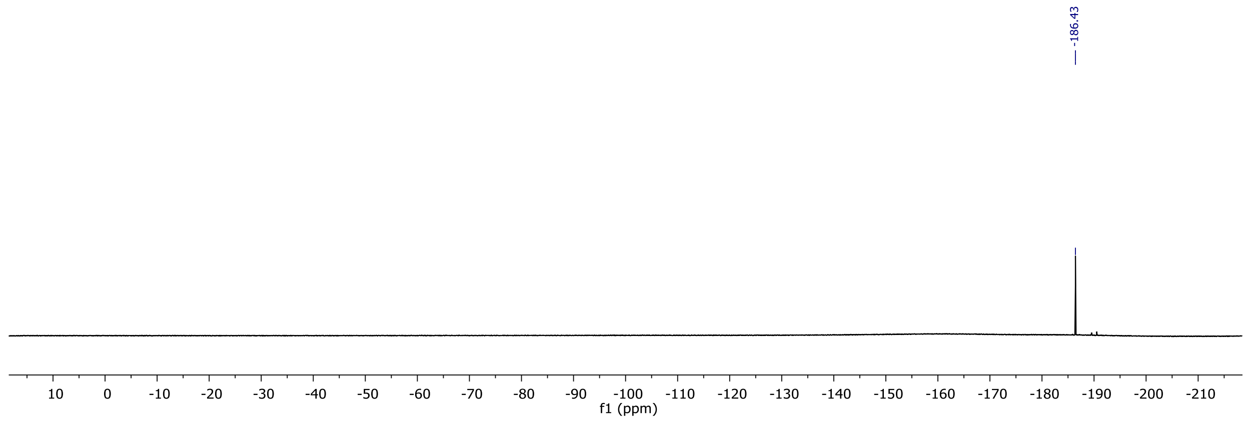

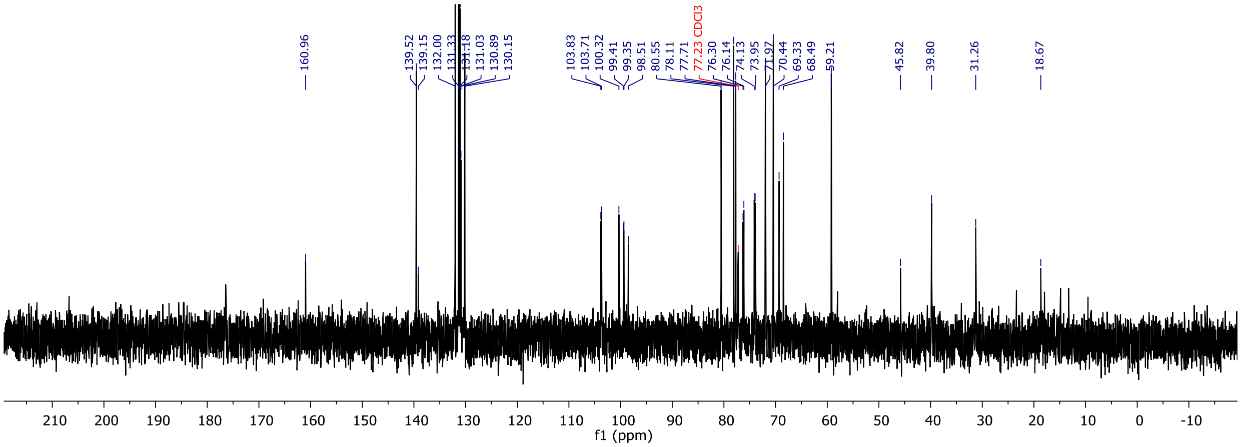
160.96
139.52
139.15
132.00
131.33
131.18
131.03
130.89
130.15
103.83
103.71
100.32
99.41
99.35
98.51
80.55
78.11
77.71
77.23 CDCl3
76.30
76.14
74.13
73.95
71.97
70.44
69.33
68.49
59.21
45.82
39.80
31.26
18.67
f1 (ppm)

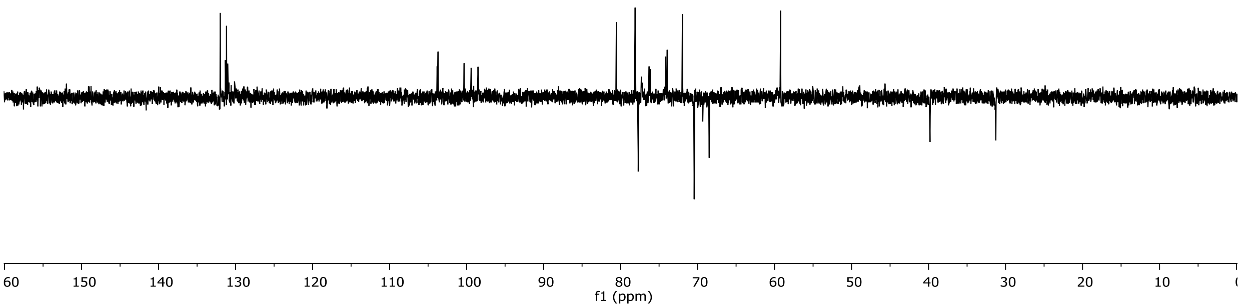
f1 (ppm)

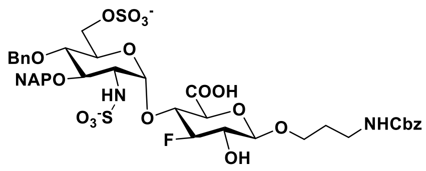
OSO3-
BnO
NAPO
HN
O3-S
COOH
F
OH
NHCbz

28

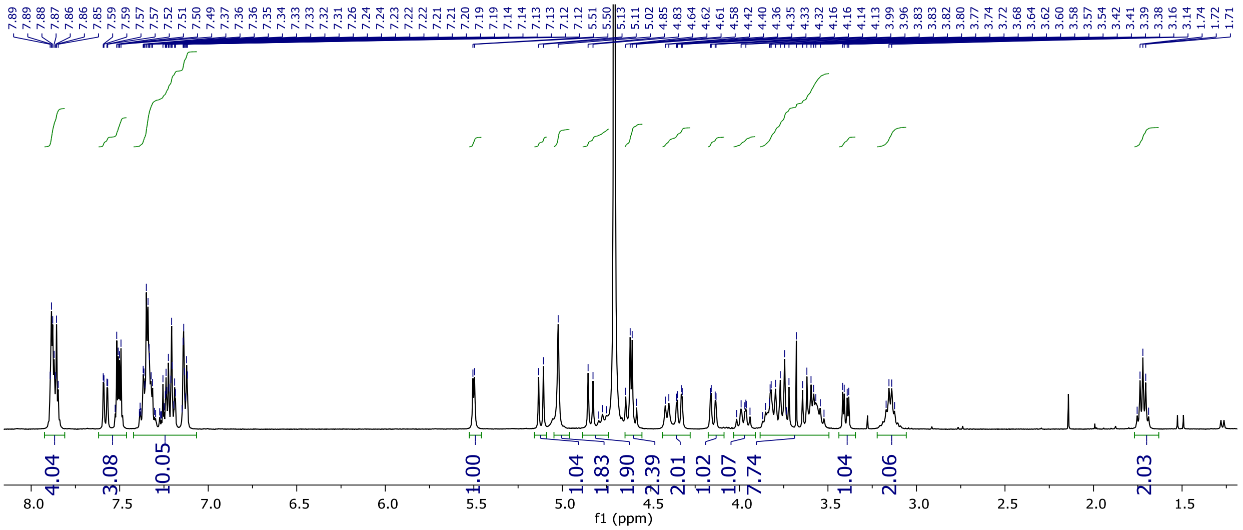
4.04
3.08
10.05
1.00
1.04
1.83
1.90
2.39
2.01
1.02
1.07
7.74
1.04
2.06
2.03
8.0 7.5 7.0 6.5 6.0 5.5 5.0 4.5 4.0 3.5 3.0 2.5 2.0 1.5
f1 (ppm)

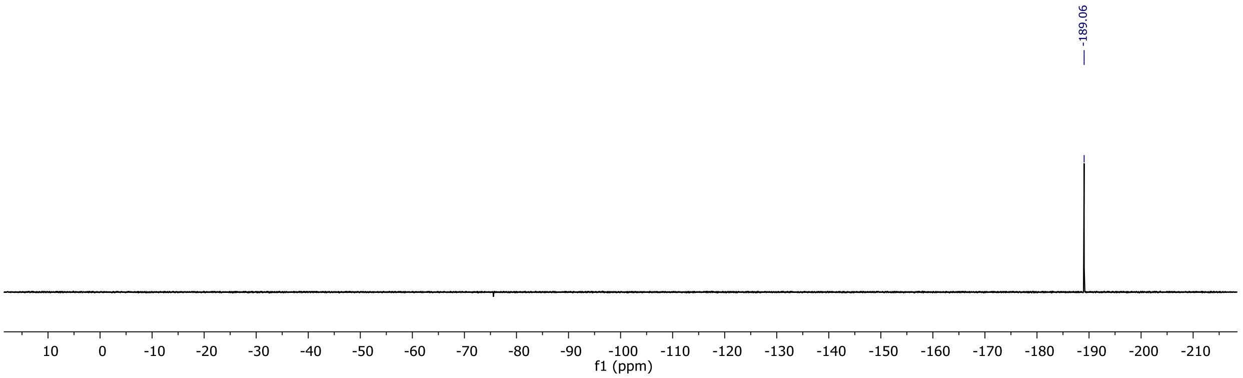
-189.06
10 0 -10 -20 -30 -40 -50 -60 -70 -80 -90 -100 -110 -120 -130 -140 -150 -160 -170 -180 -190 -200 -210
f1 (ppm)

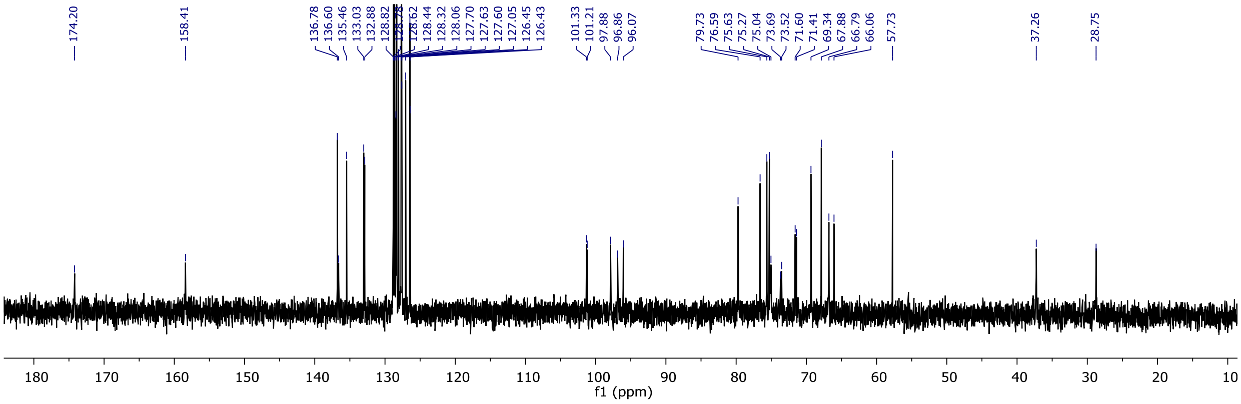
174.20
158.41
136.78
136.60
135.46
133.03
132.88
128.82
128.62
128.44
128.32
128.06
127.70
127.63
127.60
127.05
126.45
126.43
101.33
101.21
97.88
96.86
96.07
79.73
76.59
75.63
75.27
75.04
73.69
73.52
71.60
71.41
69.34
67.88
66.79
66.06
57.73
37.26
28.75
f1 (ppm)

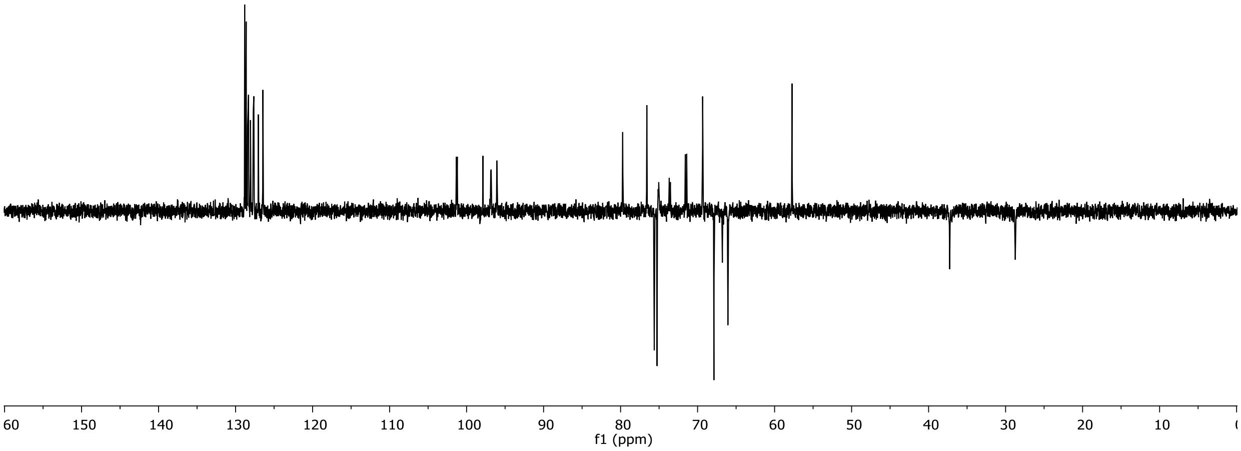
f1 (ppm)

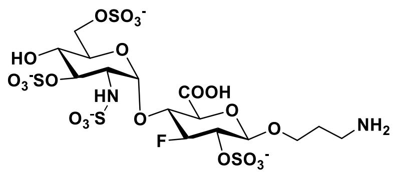

HDF-7

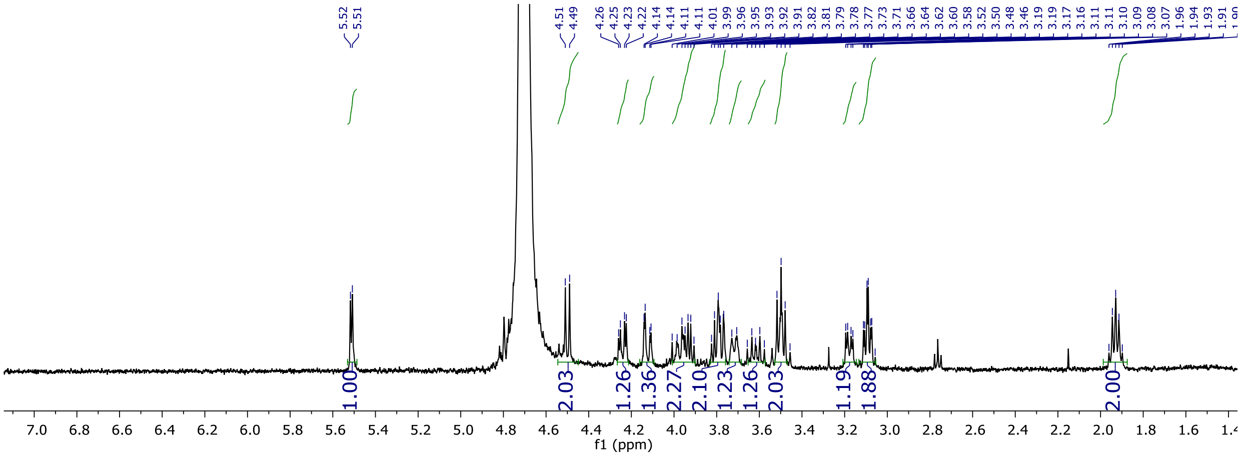

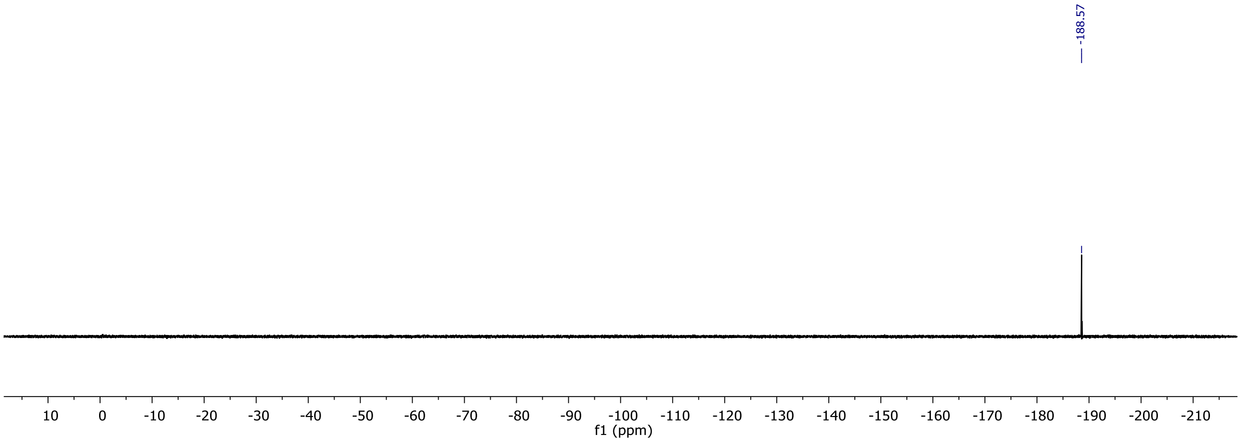

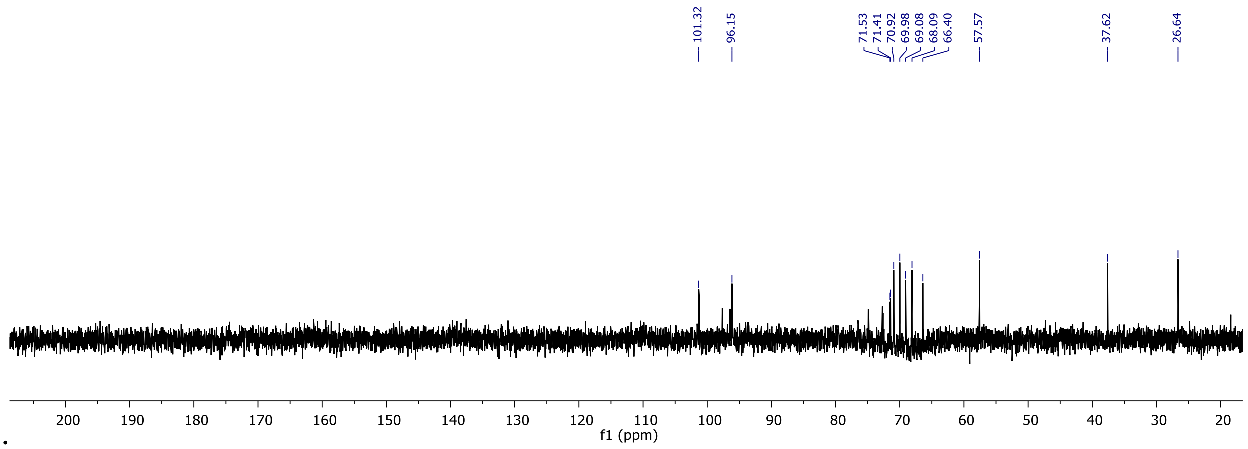
101.32
96.15
71.53
71.41
70.92
69.98
69.08
68.09
66.40
57.57
37.62
26.64
f1 (ppm)

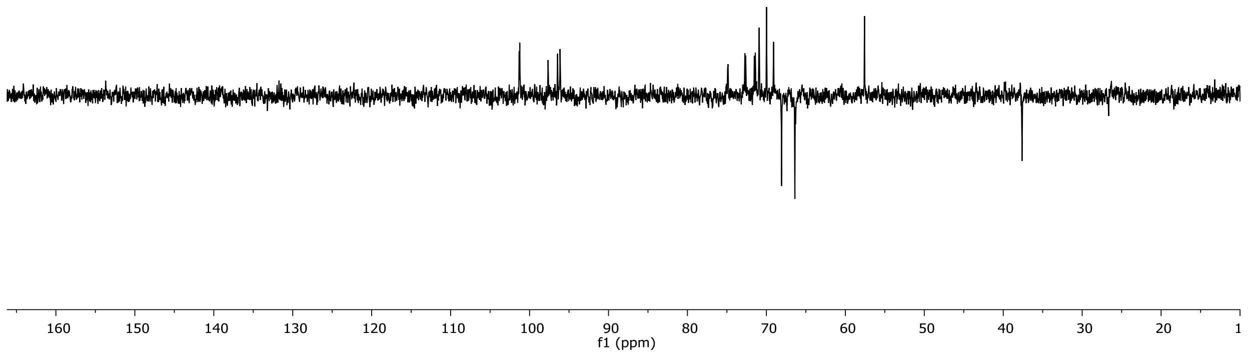
f1 (ppm)

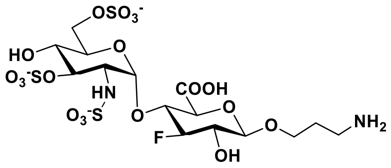
OSO3-
HO
O3-SO
O
HN
O3-S
O
COOH
O
O
NH2
F
OH

HDF-6

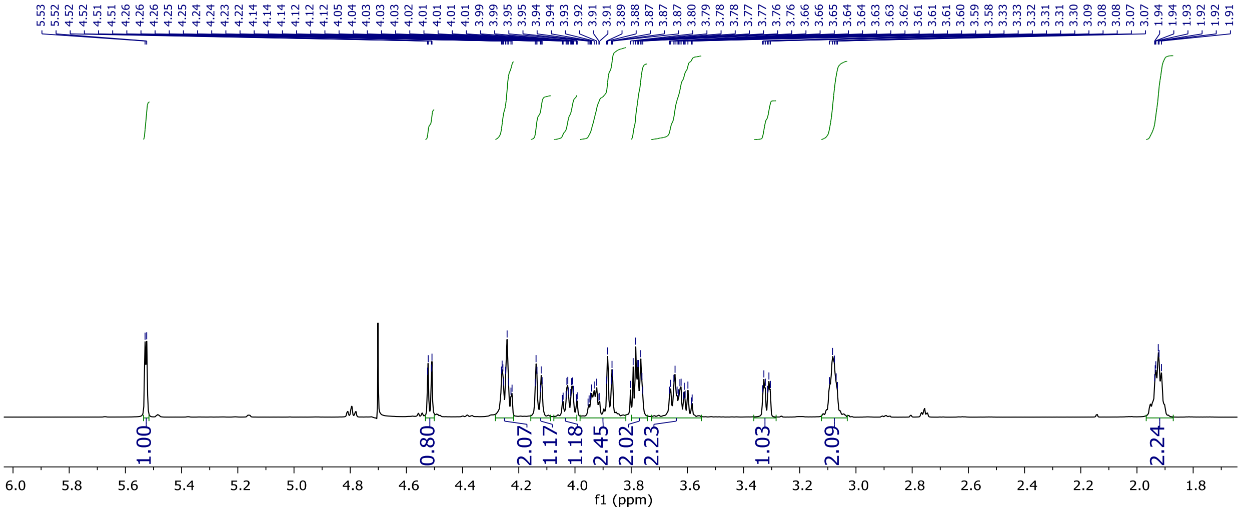
5.53 5.52 4.52 4.52 4.51 4.51 4.26 4.26 4.25 4.25 4.24 4.24 4.23 4.22 4.14 4.14 4.14 4.12 4.12 4.12 4.05 4.04 4.03 4.03 4.02 4.01 4.01 4.01 4.01 3.99 3.95 3.95 3.94 3.94 3.93 3.92 3.91 3.91 3.89 3.88 3.87 3.87 3.80 3.79 3.78 3.78 3.77 3.76 3.66 3.65 3.64 3.63 3.63 3.62 3.61 3.61 3.60 3.59 3.58 3.33 3.33 3.32 3.31 3.31 3.30 3.09 3.08 3.08 3.07 1.94 1.94 1.93 1.92 1.92 1.91
1.00
0.80
2.07
1.17
1.18
2.45
2.02
2.23
1.03
2.09
2.24
6.0 5.8 5.6 5.4 5.2 5.0 4.8 4.6 4.4 4.2 4.0 3.8 3.6 3.4 3.2 3.0 2.8 2.6 2.4 2.2 2.0 1.8
f1 (ppm)

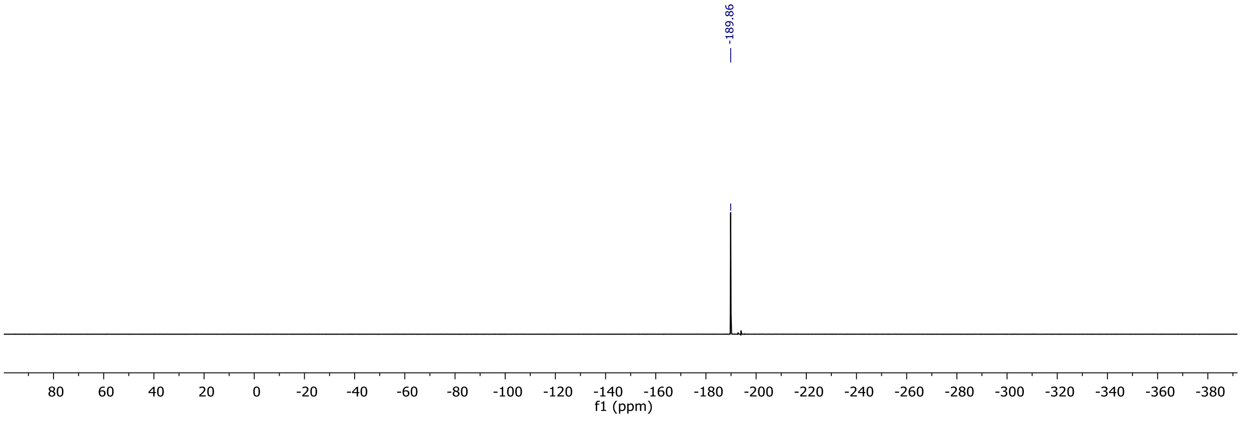
-189.86
80 60 40 20 0 -20 -40 -60 -80 -100 -120 -140 -160 -180 -200 -220 -240 -260 -280 -300 -320 -340 -360 -380
f1 (ppm)

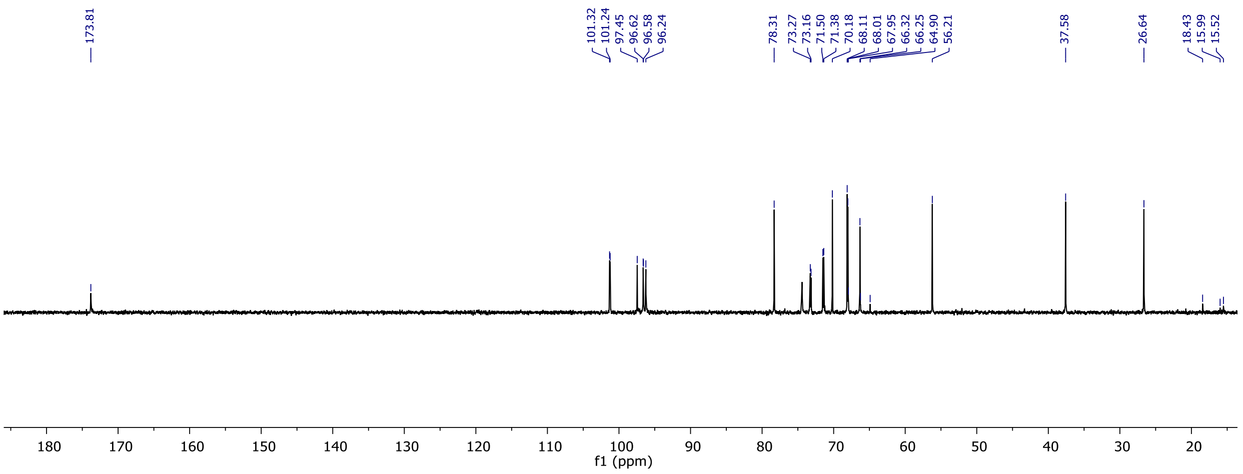
173.81
101.32
101.24
97.45
96.62
96.58
96.24
78.31
73.27
73.16
71.50
71.38
70.18
68.11
68.01
67.95
66.32
66.25
64.90
56.21
37.58
26.64
18.43
15.99
15.52
f1 (ppm)

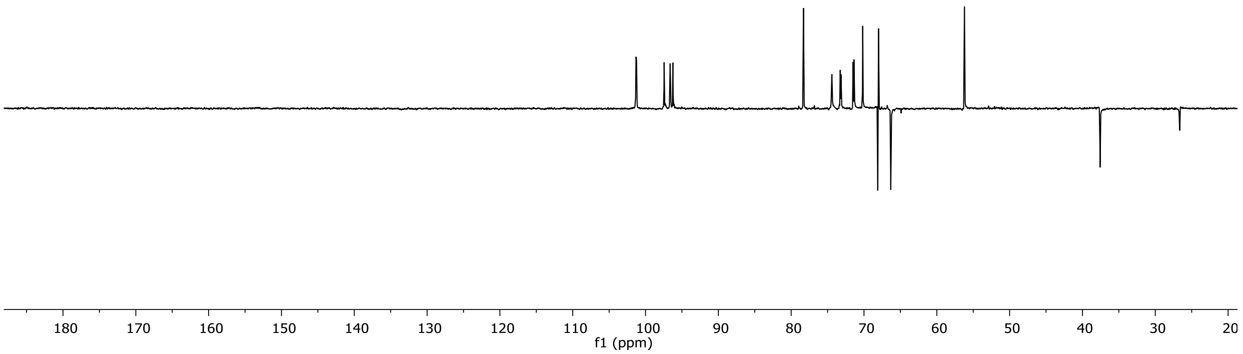
f1 (ppm)

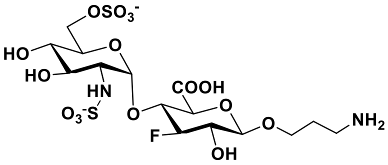

HDF-5

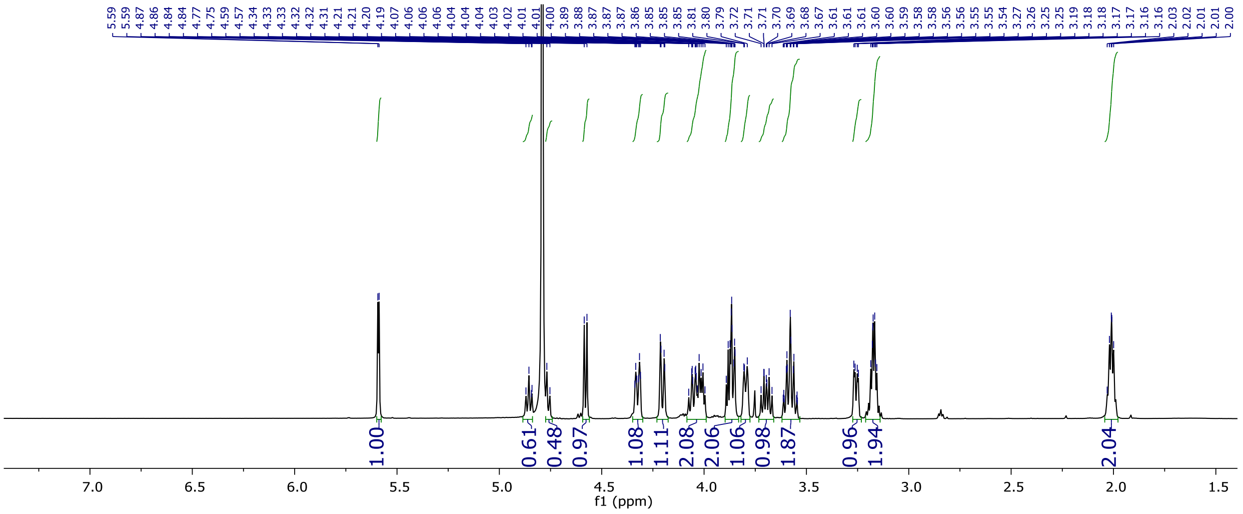

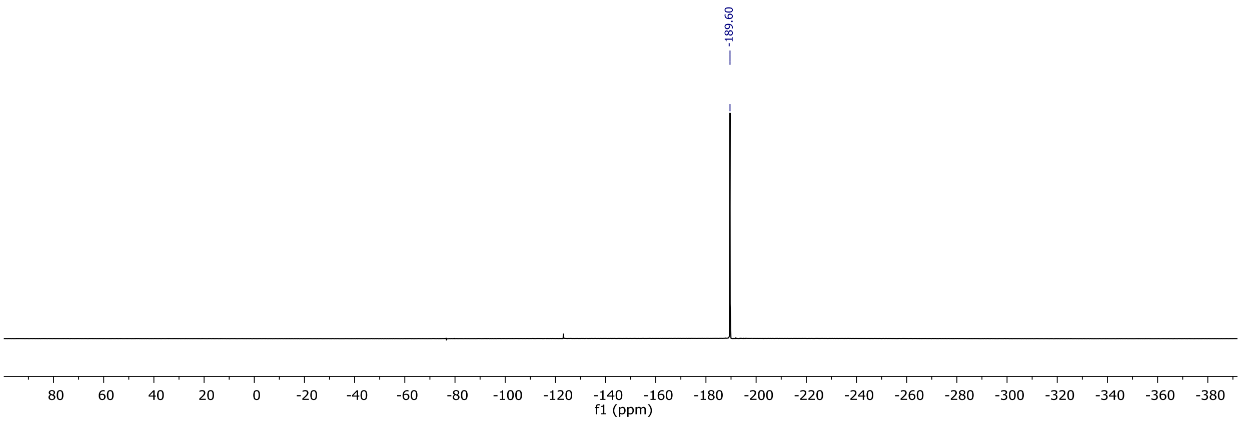

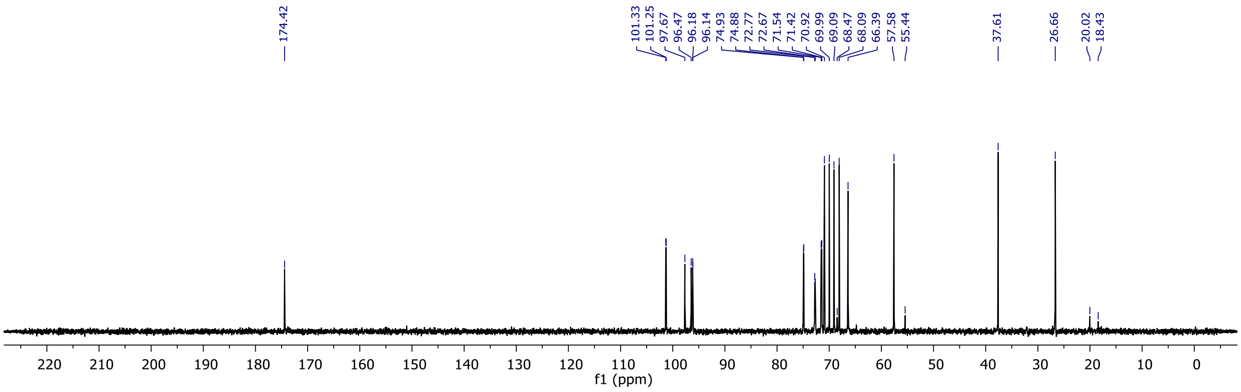
174.42
101.33
101.25
97.67
96.47
96.18
96.14
74.93
74.88
72.77
72.67
71.54
71.42
70.92
69.99
69.09
68.47
68.09
66.39
57.58
55.44
37.61
26.66
20.02
18.43
220 210 200 190 180 170 160 150 140 130 120 110 100 90 80 70 60 50 40 30 20 10 0
f1 (ppm)

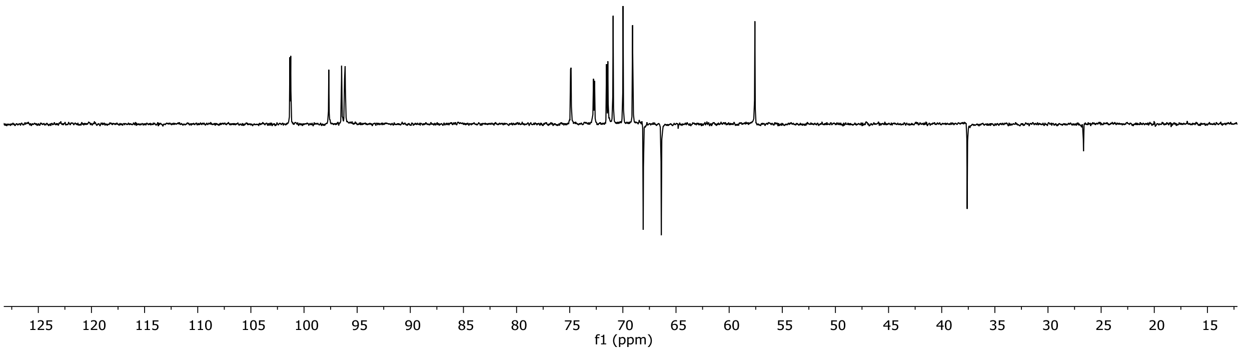
125 120 115 110 105 100 95 90 85 80 75 70 65 60 55 50 45 40 35 30 25 20 15
f1 (ppm)

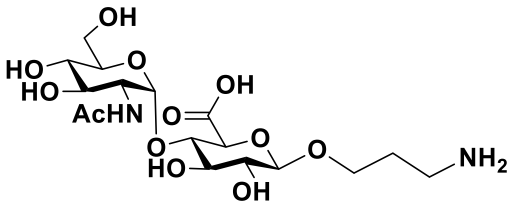
OH
HO
O
HO
AcHN
O
OH
O
O
O
HO
OH
NH2

HD-1

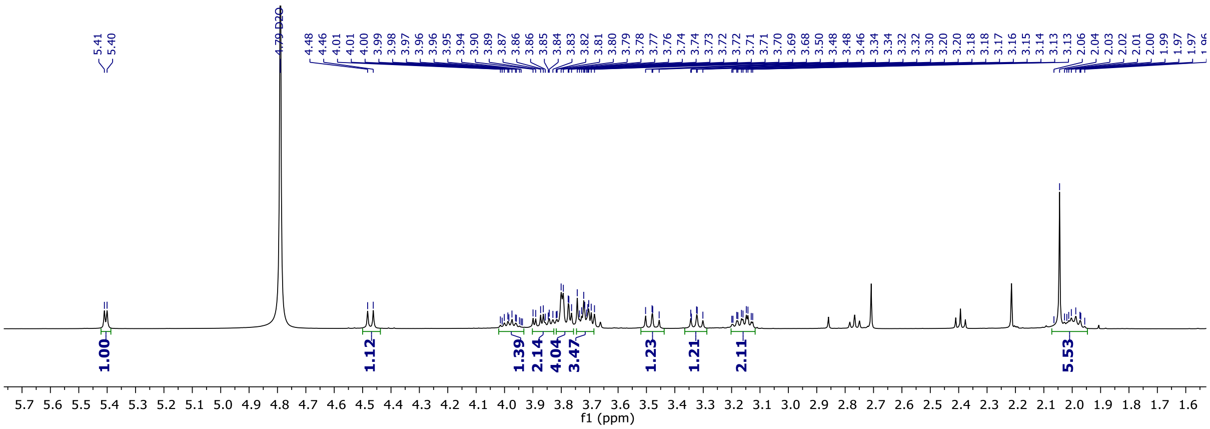
5.41
5.40
4.79 D2O
1.00
1.12
1.39
2.14
4.04
3.47
1.23
1.21
2.11
5.53
f1 (ppm)

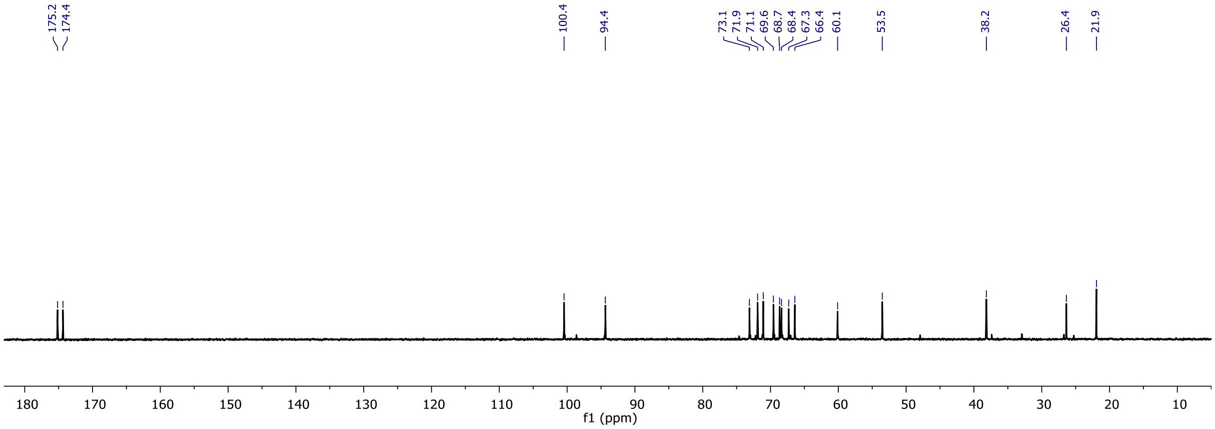
175.2
174.4
100.4
94.4
73.1
71.9
71.1
69.6
68.7
68.4
67.3
66.4
60.1
53.5
38.2
26.4
21.9
f1 (ppm)

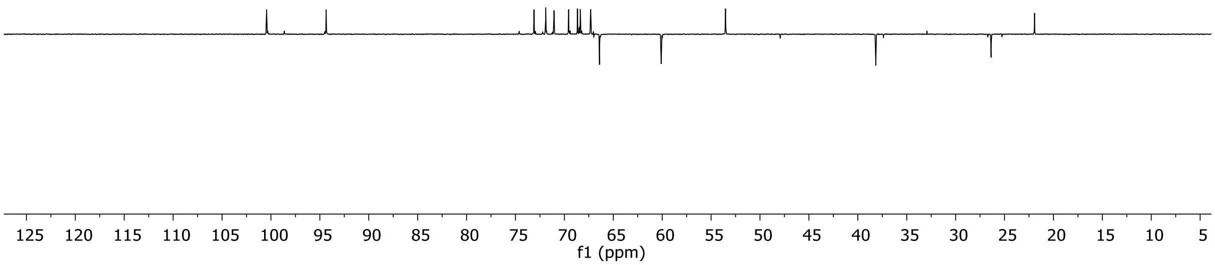
f1 (ppm)

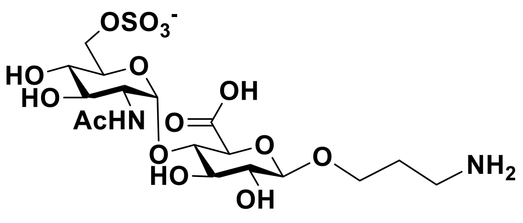
OSO3-
HO
HO
AcHN
O
OH
HO
OH
NH2

HD-2

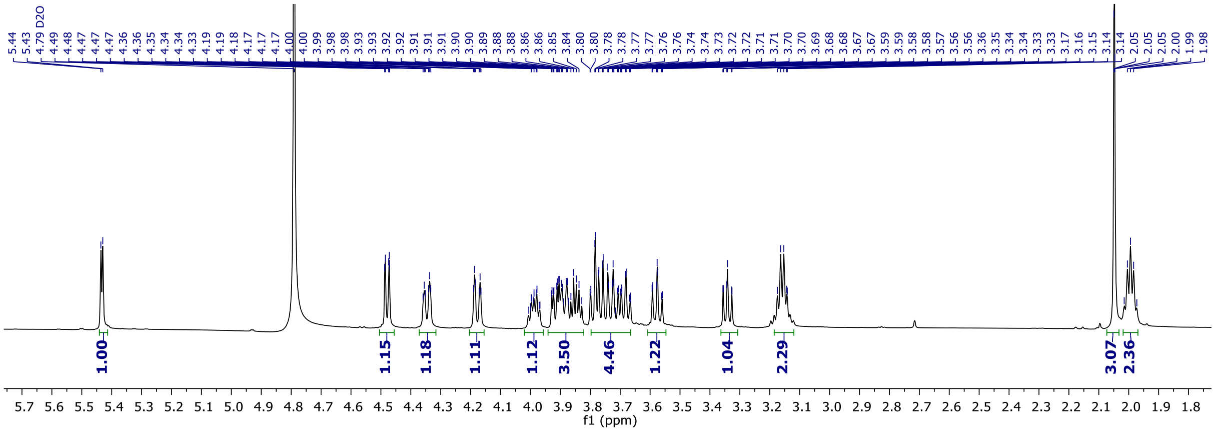
1.00
1.15
1.18
1.11
1.12
3.50
4.46
1.22
1.04
2.29
3.07
2.36
f1 (ppm)

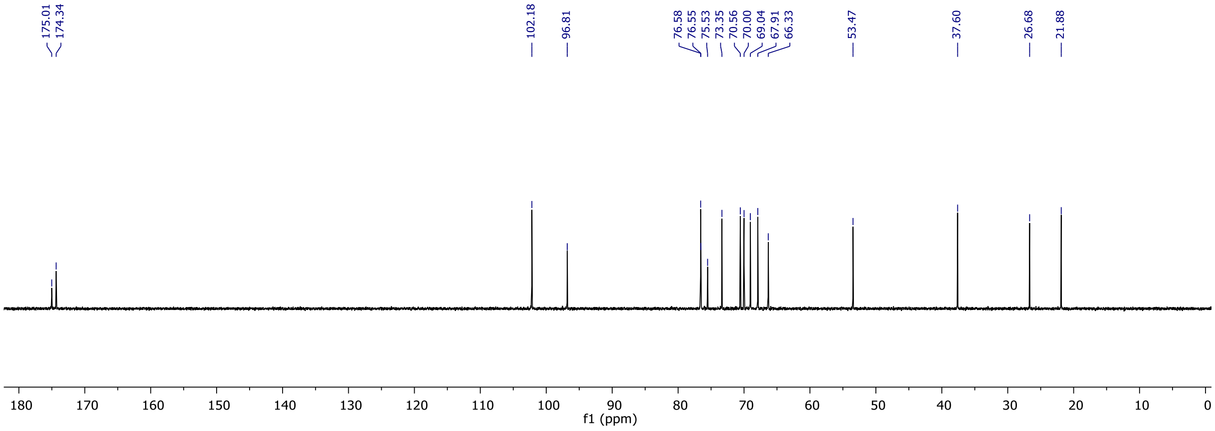
175.01
174.34
102.18
96.81
76.58
76.55
75.53
73.35
70.56
70.00
69.04
67.91
66.33
53.47
37.60
26.68
21.88
f1 (ppm)

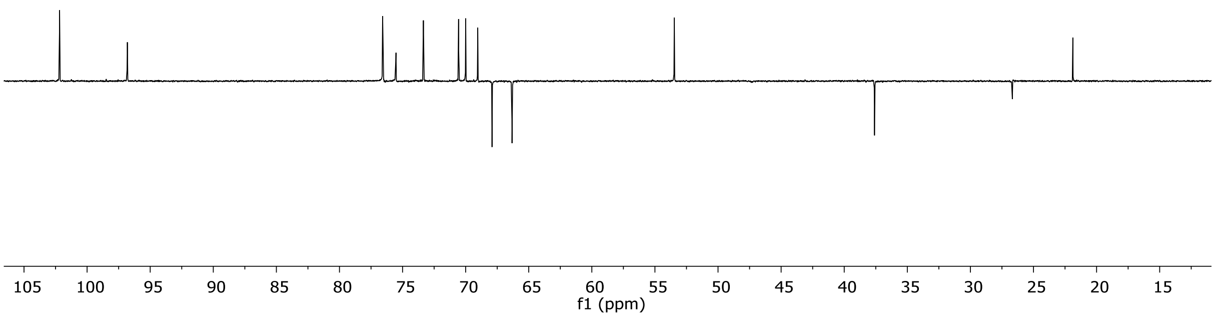
f1 (ppm)

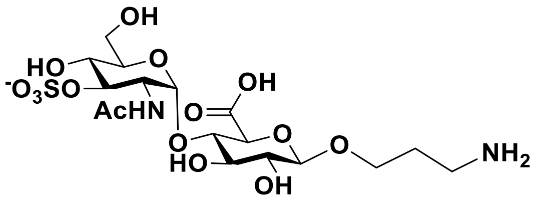

HD-3

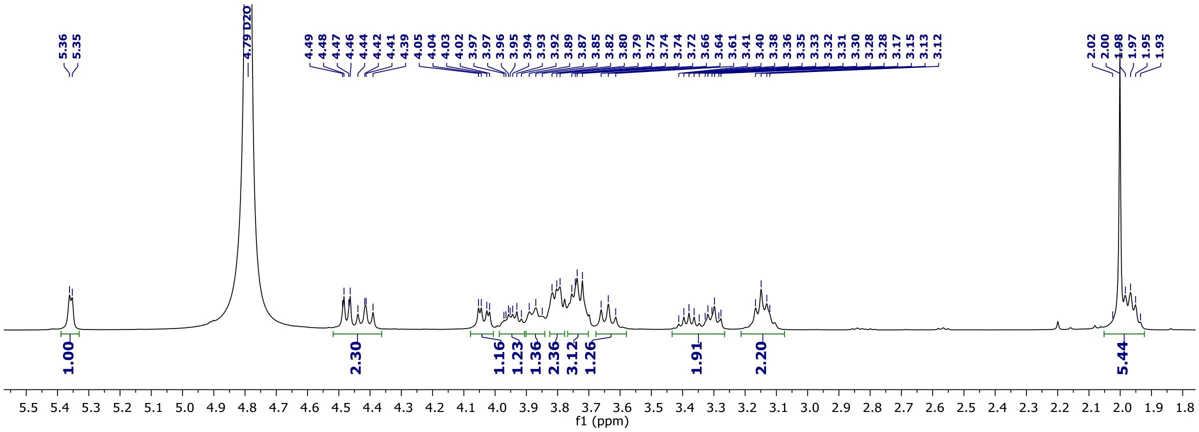

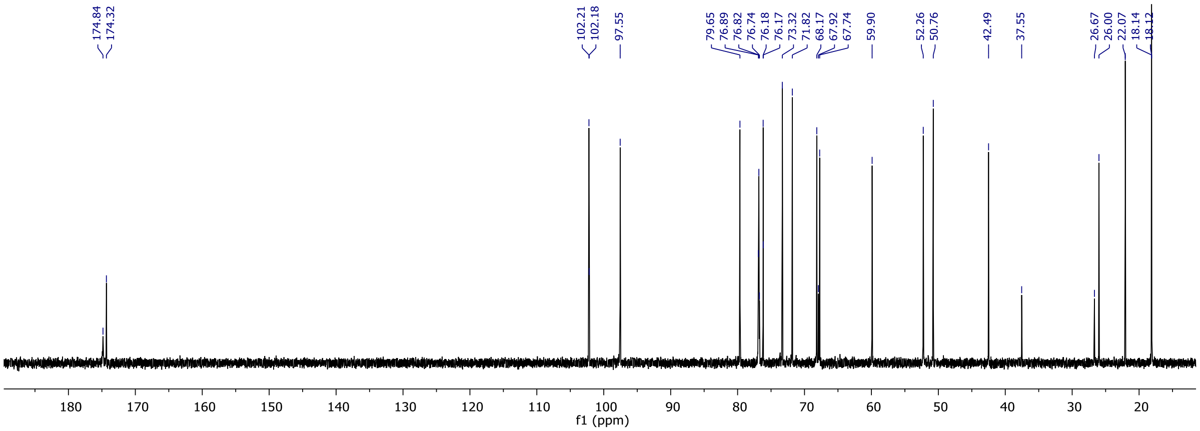

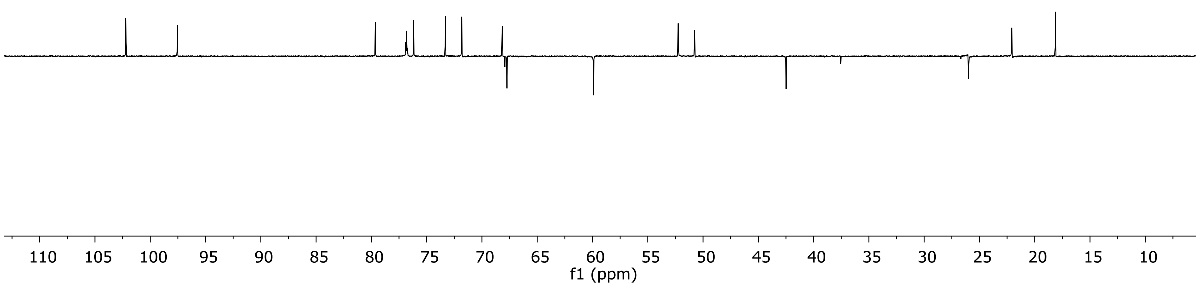

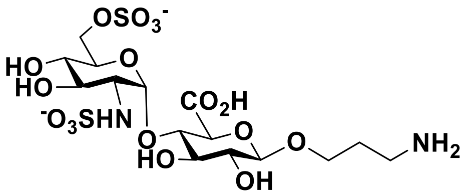
OSO3-
HO
HO
O
-O3SHN
CO2H
O
O
HO
OH
O
NH2

HD-4

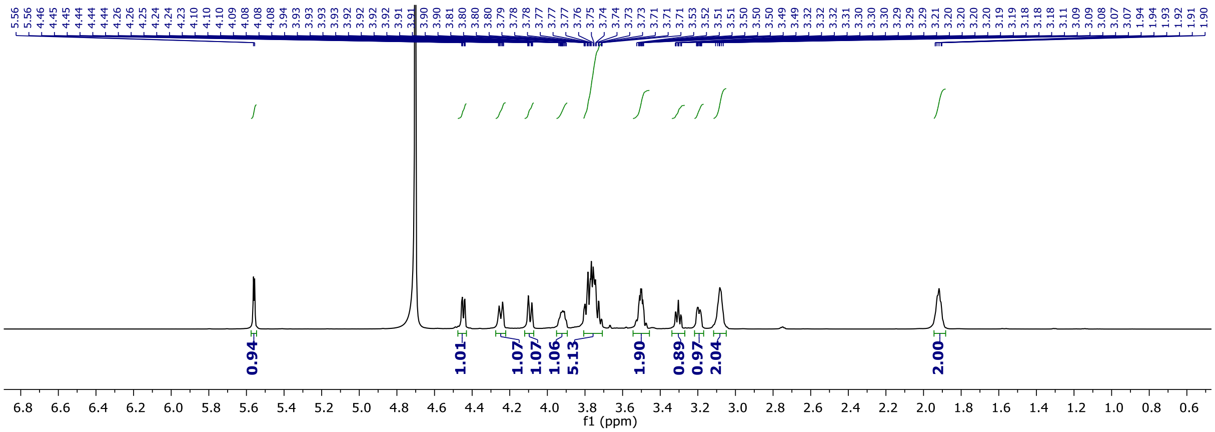
0.94
1.01
1.07
1.07
1.06
5.13
1.90
0.89
0.97
2.04
2.00
f1 (ppm)

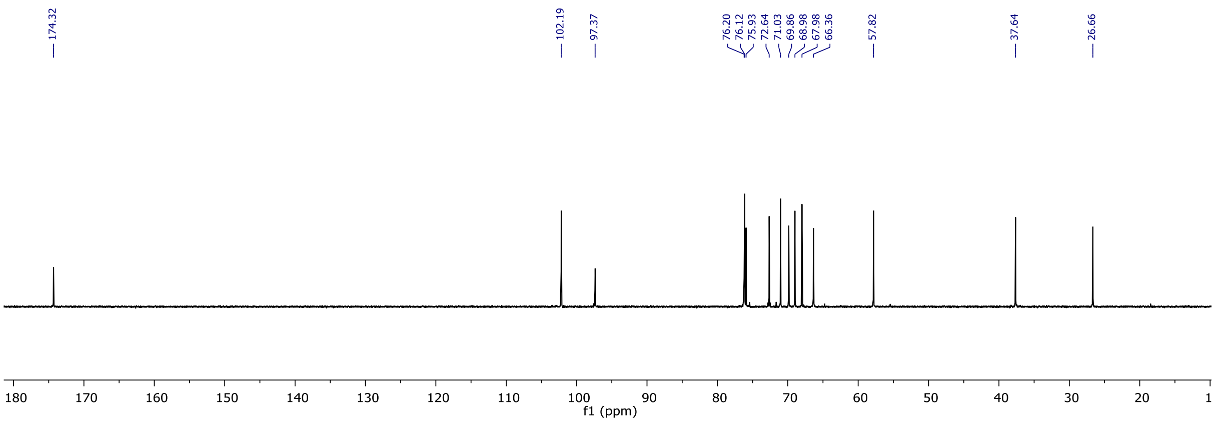
174.32
102.19
97.37
76.20
76.12
75.93
72.64
71.03
69.86
68.98
67.98
66.36
57.82
37.64
26.66
f1 (ppm)

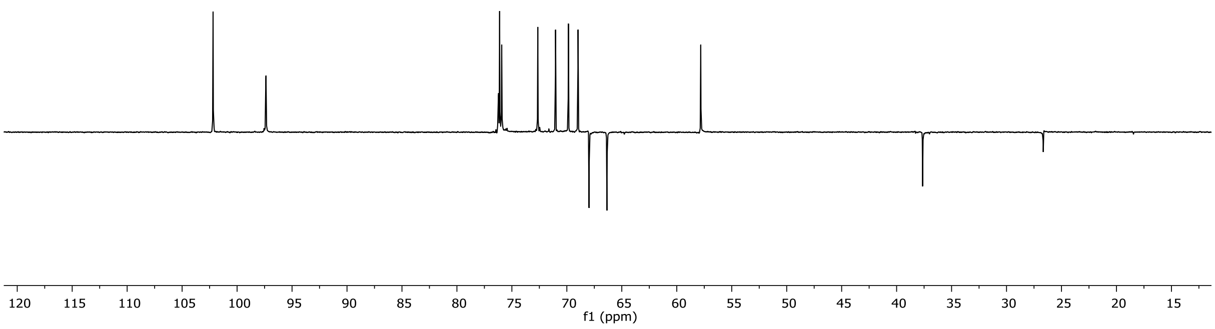
f1 (ppm)

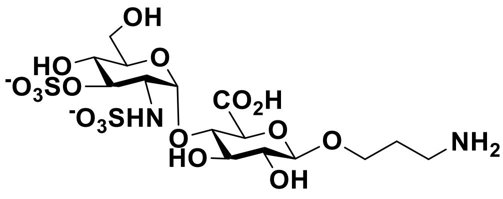

HD-5

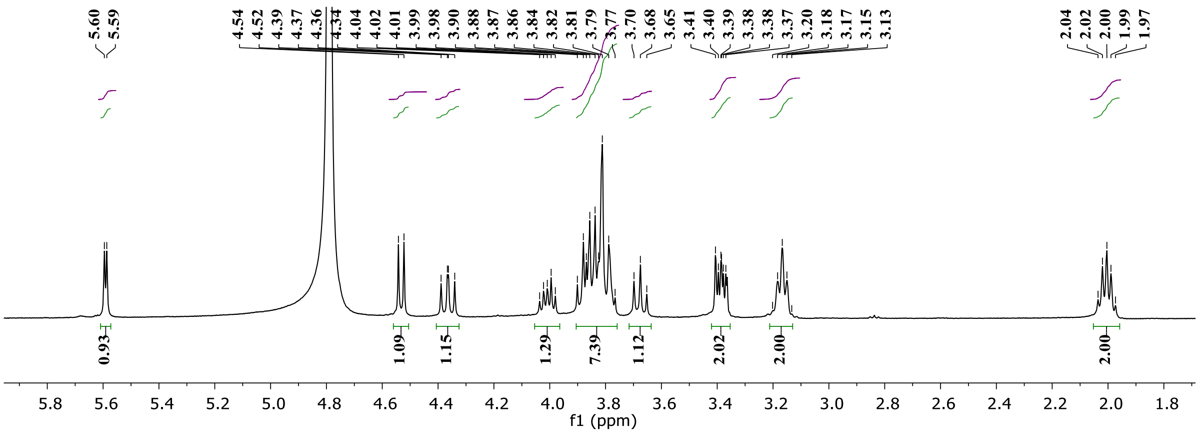
5.60
5.59
4.54
4.52
4.39
4.37
4.36
4.34
4.04
4.02
4.01
3.99
3.98
3.90
3.88
3.87
3.86
3.84
3.82
3.81
3.79
3.77
3.70
3.68
3.65
3.41
3.40
3.39
3.38
3.38
3.37
3.20
3.18
3.17
3.15
3.13
2.04
2.02
2.00
1.99
1.97
0.93
1.09
1.15
1.29
7.39
1.12
2.02
2.00
2.00
f1 (ppm)

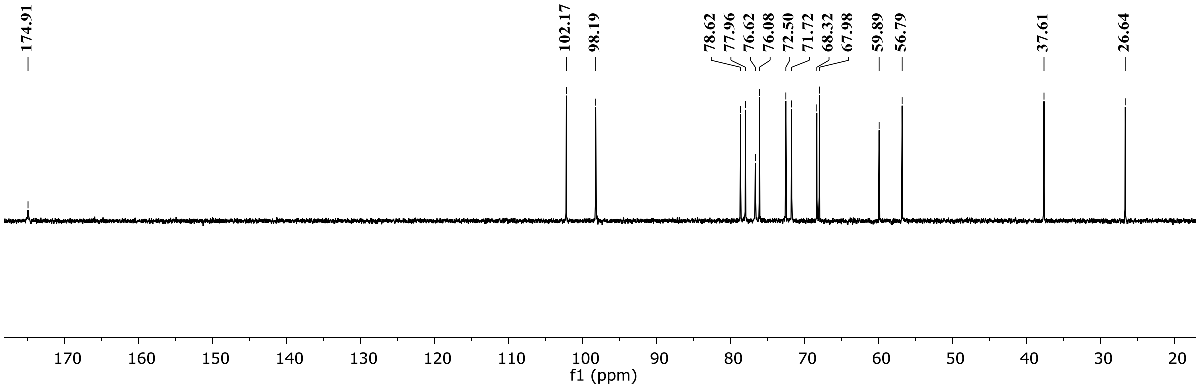
174.91
102.17
98.19
78.62
77.96
76.62
76.08
72.50
71.72
68.32
67.98
59.89
56.79
37.61
26.64
f1 (ppm)

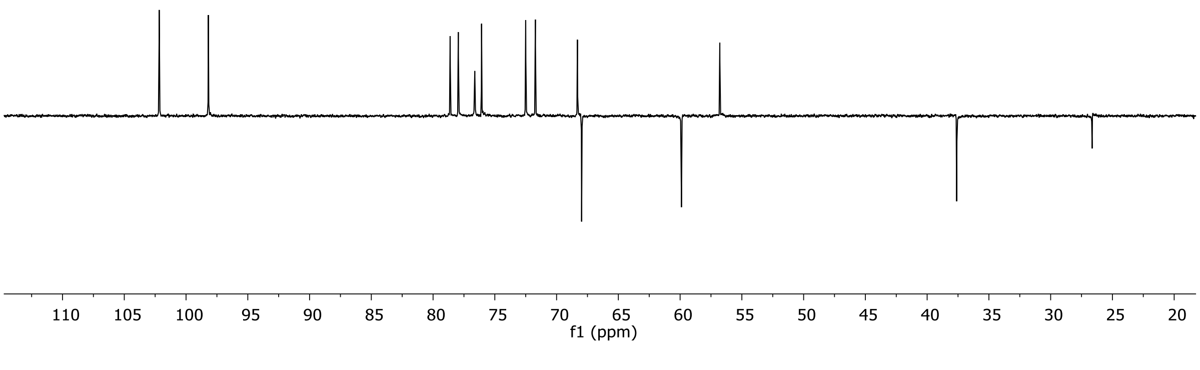
f1 (ppm)

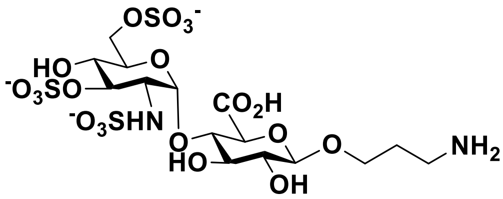

HD-6

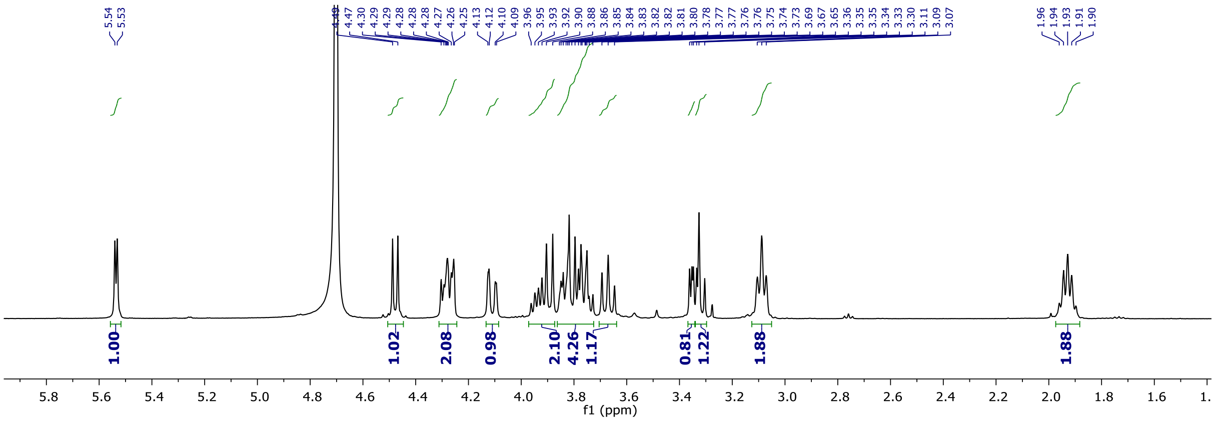

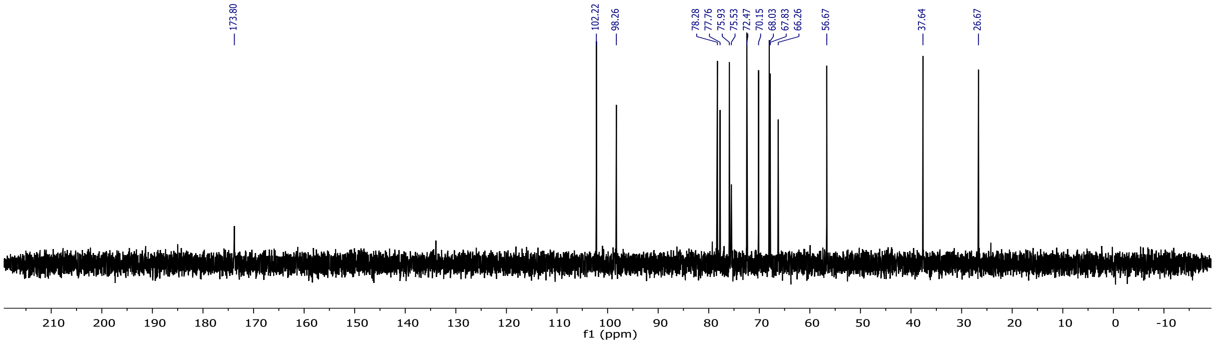

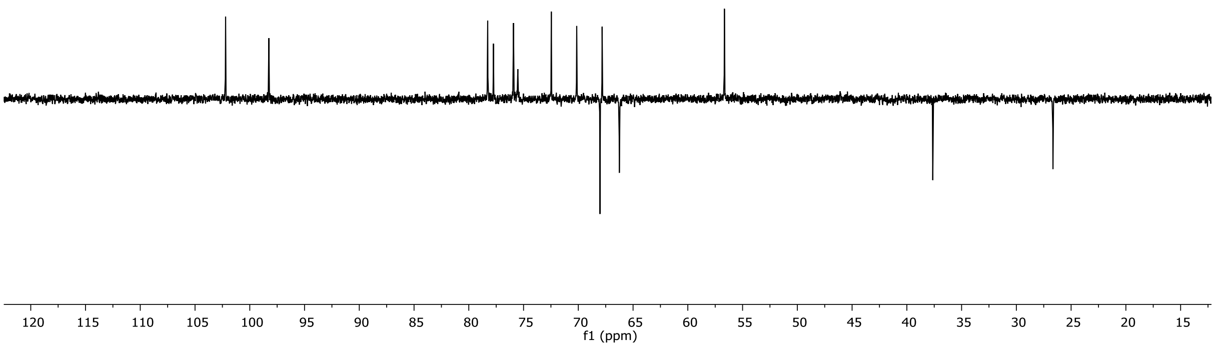

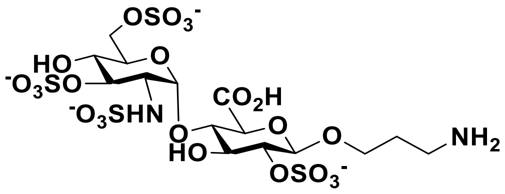

HD-7

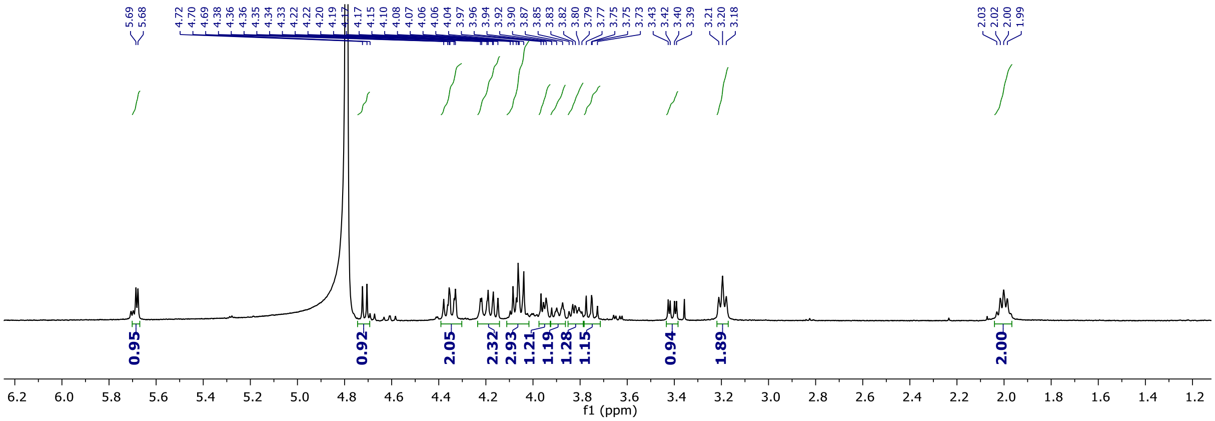
5.69 5.68
4.72 4.70 4.69 4.38 4.36 4.35 4.34 4.33 4.22 4.22 4.20 4.19 4.17 4.17 4.15 4.10 4.08 4.07 4.06 4.06 4.04 3.97 3.96 3.94 3.92 3.90 3.87 3.85 3.83 3.82 3.80 3.79 3.77 3.75 3.75 3.73
3.43 3.42 3.40 3.39
3.21 3.20 3.18
2.03 2.02 2.00 1.99
0.95 0.92 2.05 2.32 2.93 1.21 1.19 1.28 1.15 0.94 1.89 2.00
f1 (ppm)

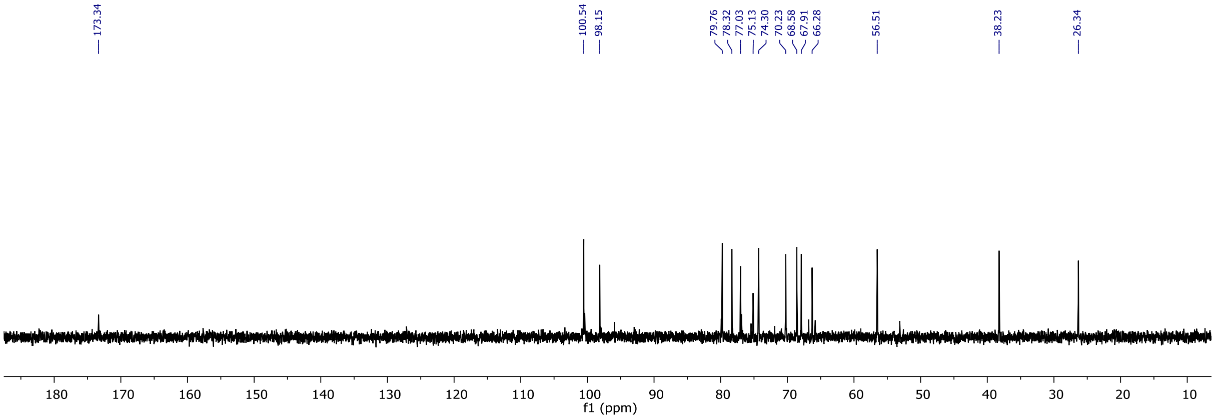
173.34
100.54 98.15
79.76 78.32 77.03 75.13 74.30 70.23 68.58 67.91 66.28
56.51
38.23
26.34
f1 (ppm)

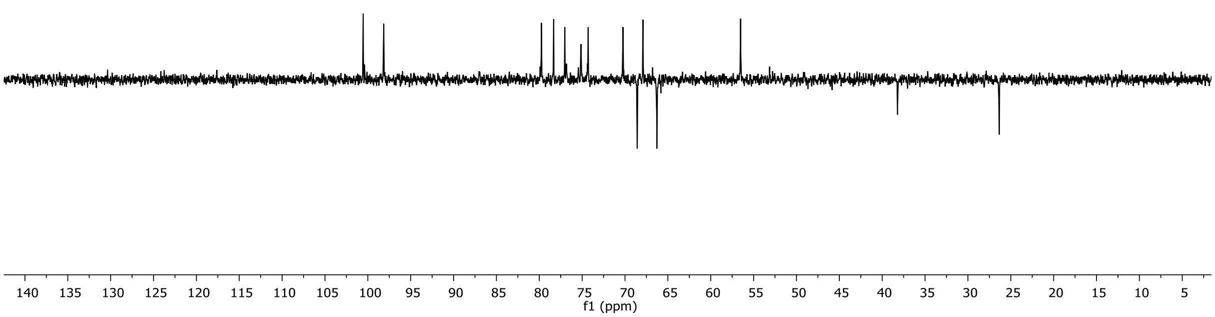
f1 (ppm)

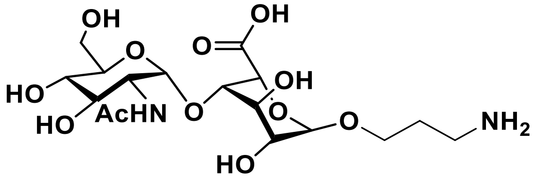

HD-8

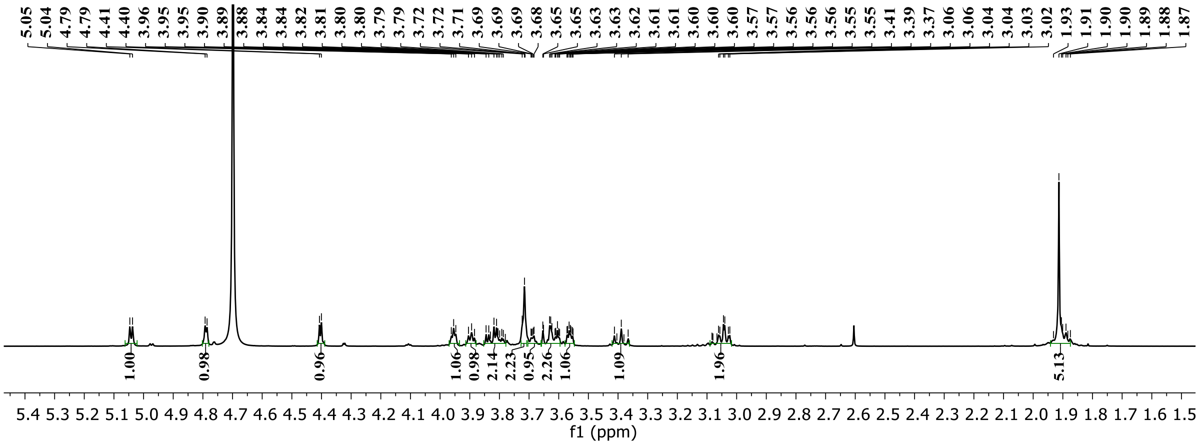

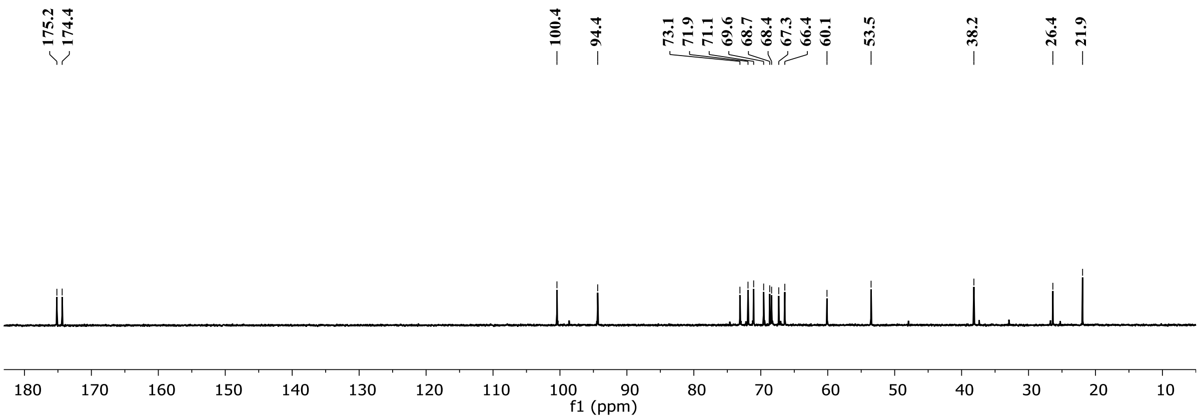

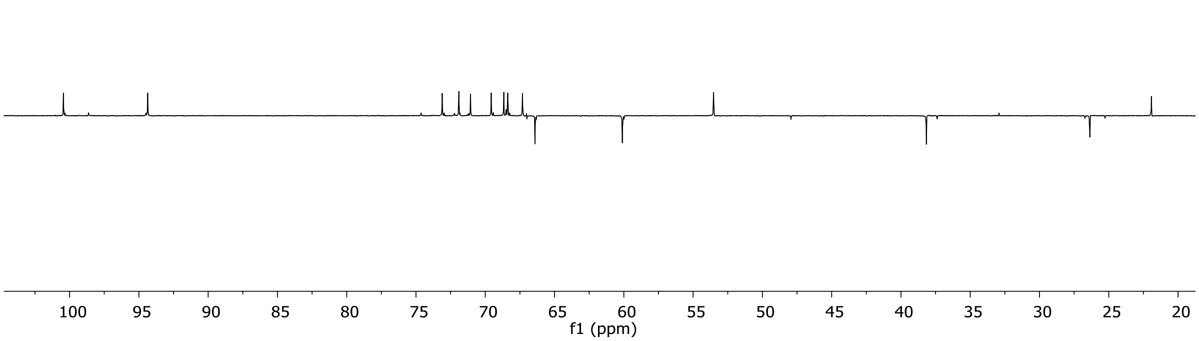

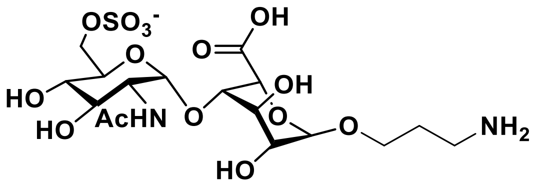

HD-9

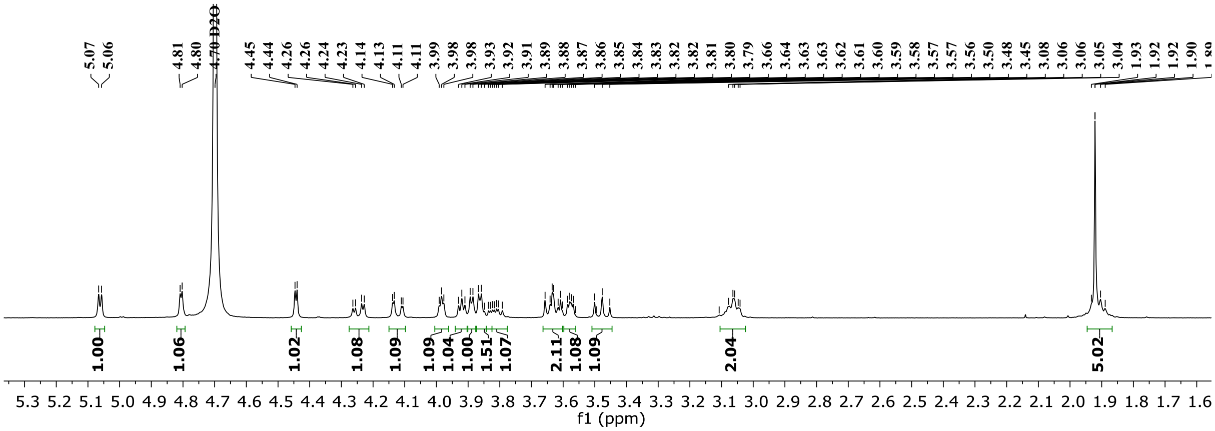

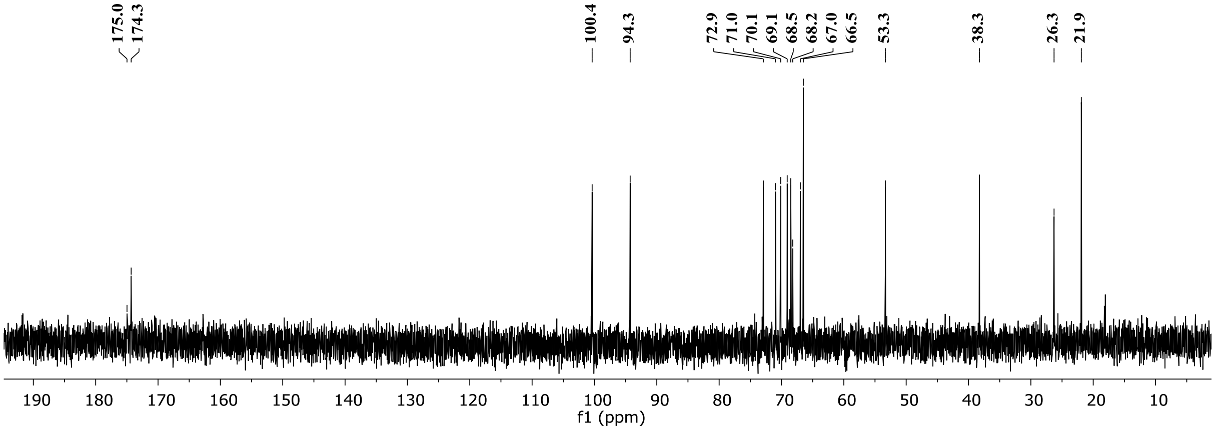

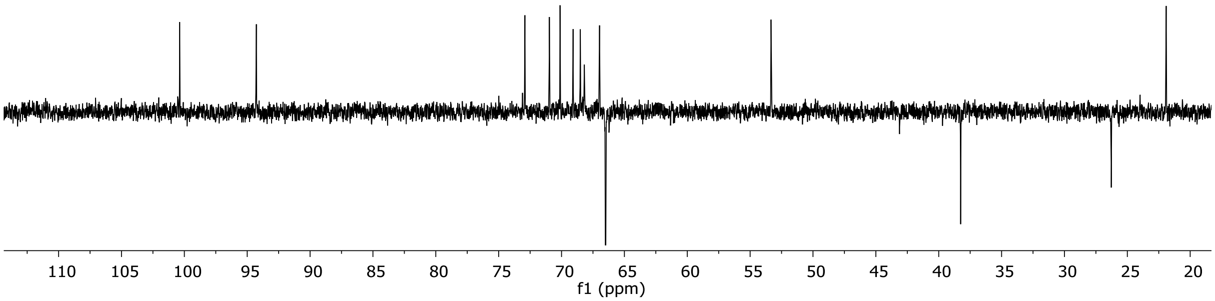

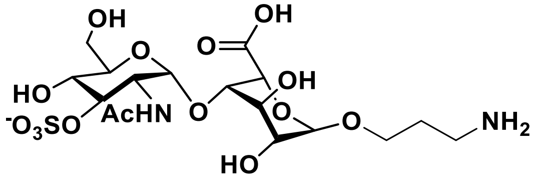
OH
OH
O
O
HO
OH
-O3SO
AcHN
O
O
O
NH2
HO

HD-10

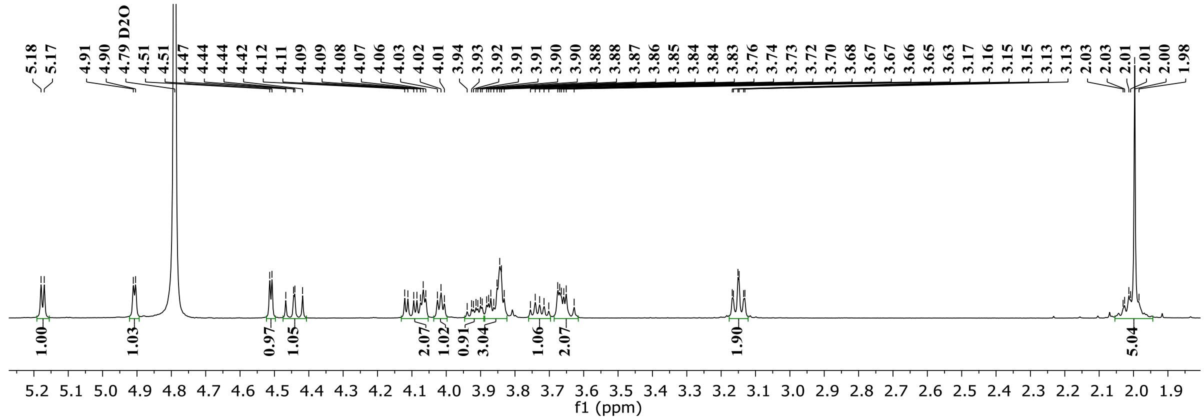
5.18
5.17
4.91
4.90
4.79 D2O
4.51
4.51
4.47
4.44
4.44
4.42
4.12
4.11
4.09
4.09
4.08
4.07
4.06
4.03
4.02
4.01
3.94
3.93
3.92
3.91
3.91
3.90
3.90
3.88
3.88
3.87
3.86
3.85
3.84
3.84
3.83
3.76
3.74
3.73
3.72
3.70
3.68
3.67
3.67
3.66
3.65
3.63
3.17
3.16
3.15
3.15
3.13
3.13
2.03
2.03
2.01
2.01
2.00
1.98
1.00
1.03
0.97
1.05
2.07
1.02
0.91
3.04
1.06
2.07
1.90
5.04
5.2 5.1 5.0 4.9 4.8 4.7 4.6 4.5 4.4 4.3 4.2 4.1 4.0 3.9 3.8 3.7 3.6 3.5 3.4 3.3 3.2 3.1 3.0 2.9 2.8 2.7 2.6 2.5 2.4 2.3 2.2 2.1 2.0 1.9
f1 (ppm)

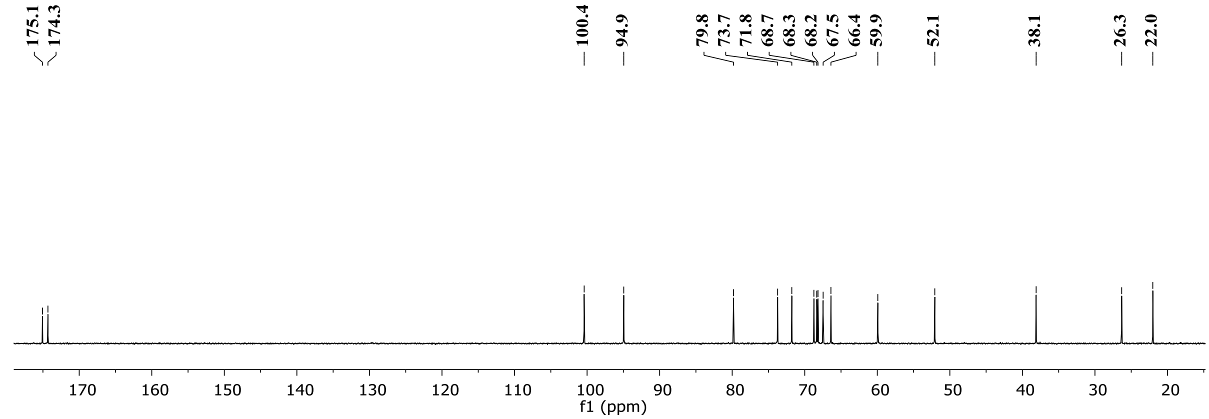
175.1
174.3
100.4
94.9
79.8
73.7
71.8
68.7
68.3
68.2
67.5
66.4
59.9
52.1
38.1
26.3
22.0
170 160 150 140 130 120 110 100 90 80 70 60 50 40 30 20
f1 (ppm)

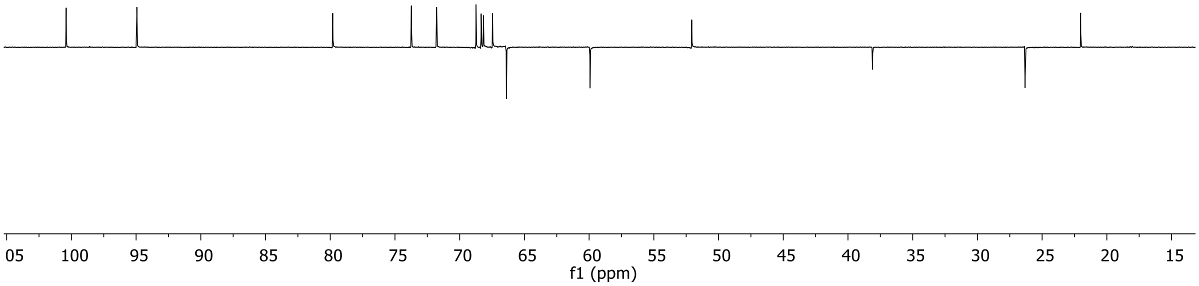
05 100 95 90 85 80 75 70 65 60 55 50 45 40 35 30 25 20 15
f1 (ppm)

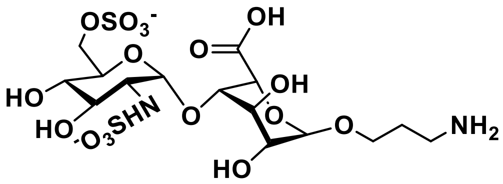
OSO3-
OH
HO
HO
-O3SHN
O
OH
NH2
HO

HD-11

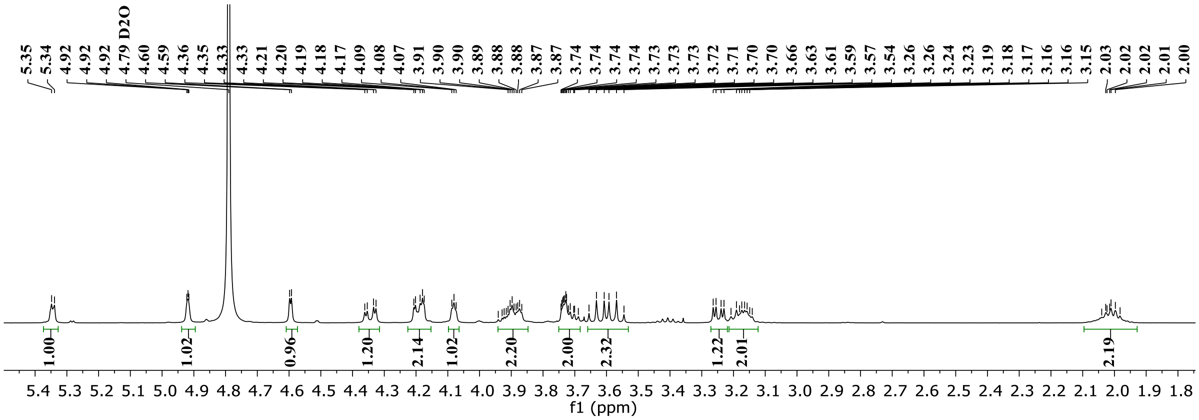
5.35 5.34 4.92 4.92 4.92 4.79 D2O 4.60 4.59 4.36 4.35 4.33 4.33 4.21 4.20 4.19 4.18 4.17 4.09 4.08 4.07 3.91 3.90 3.90 3.89 3.88 3.88 3.87 3.87 3.74 3.74 3.74 3.74 3.73 3.73 3.73 3.72 3.71 3.70 3.70 3.66 3.63 3.61 3.59 3.57 3.54 3.26 3.26 3.24 3.23 3.19 3.18 3.17 3.16 3.16 3.15 2.03 2.02 2.02 2.01 2.00
1.00 1.02 0.96 1.20 2.14 1.02 2.20 2.00 2.32 1.22 2.01 2.19
5.4 5.3 5.2 5.1 5.0 4.9 4.8 4.7 4.6 4.5 4.4 4.3 4.2 4.1 4.0 3.9 3.8 3.7 3.6 3.5 3.4 3.3 3.2 3.1 3.0 2.9 2.8 2.7 2.6 2.5 2.4 2.3 2.2 2.1 2.0 1.9 1.8
f1 (ppm)

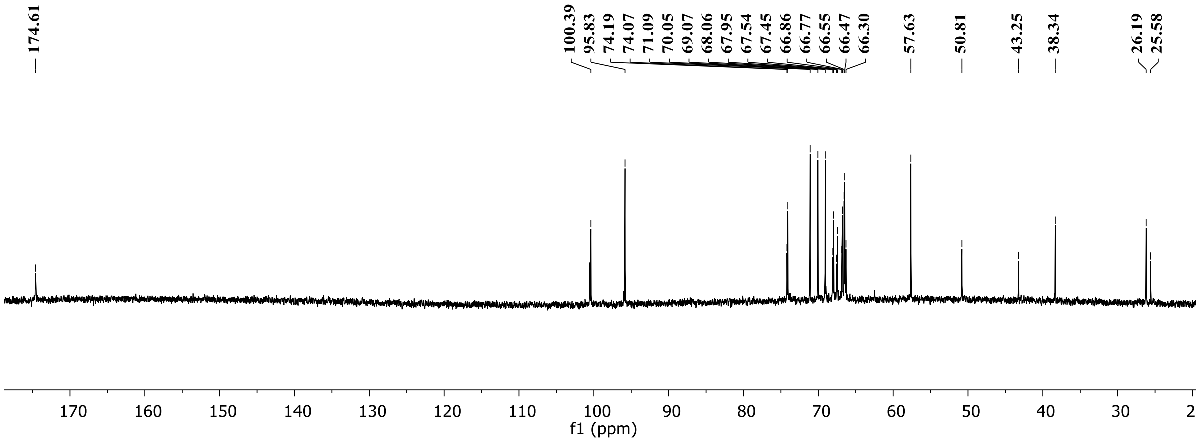
174.61
100.39 95.83 74.19 74.07 71.09 70.05 69.07 68.06 67.95 67.54 67.45 66.86 66.77 66.55 66.47 66.30
57.63
50.81
43.25
38.34
26.19 25.58
170 160 150 140 130 120 110 100 90 80 70 60 50 40 30 2
f1 (ppm)

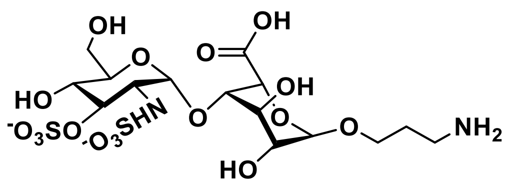

HD-12

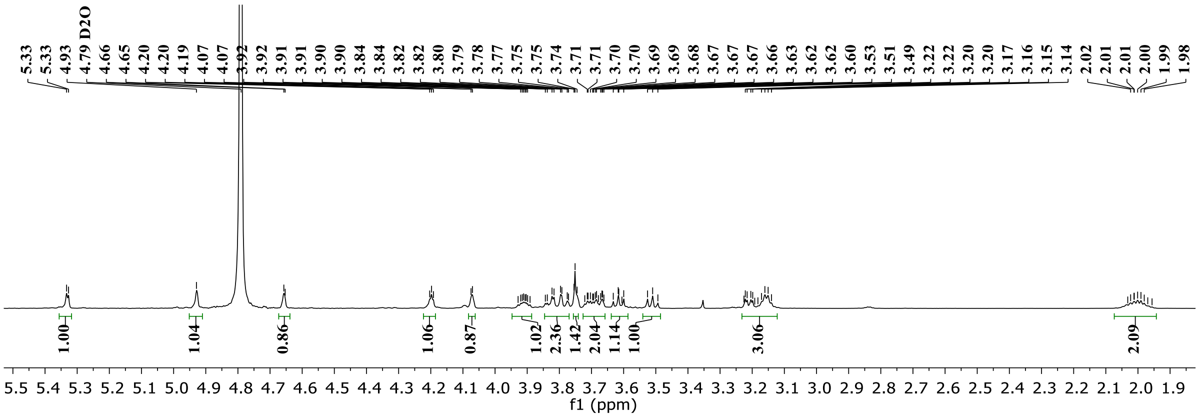
5.33
5.33
4.93
4.79 D2O
4.66
4.65
4.20
4.20
4.19
4.07
4.07
3.92
3.92
3.91
3.91
3.90
3.90
3.84
3.84
3.82
3.82
3.80
3.79
3.78
3.77
3.75
3.75
3.74
3.71
3.71
3.70
3.70
3.69
3.69
3.68
3.67
3.67
3.66
3.63
3.62
3.62
3.60
3.53
3.51
3.49
3.22
3.22
3.20
3.20
3.17
3.16
3.15
3.14
2.02
2.01
2.01
2.00
1.99
1.98
1.00
1.04
0.86
1.06
0.87
1.02
2.36
1.42
2.04
1.14
1.00
3.06
2.09
f1 (ppm)

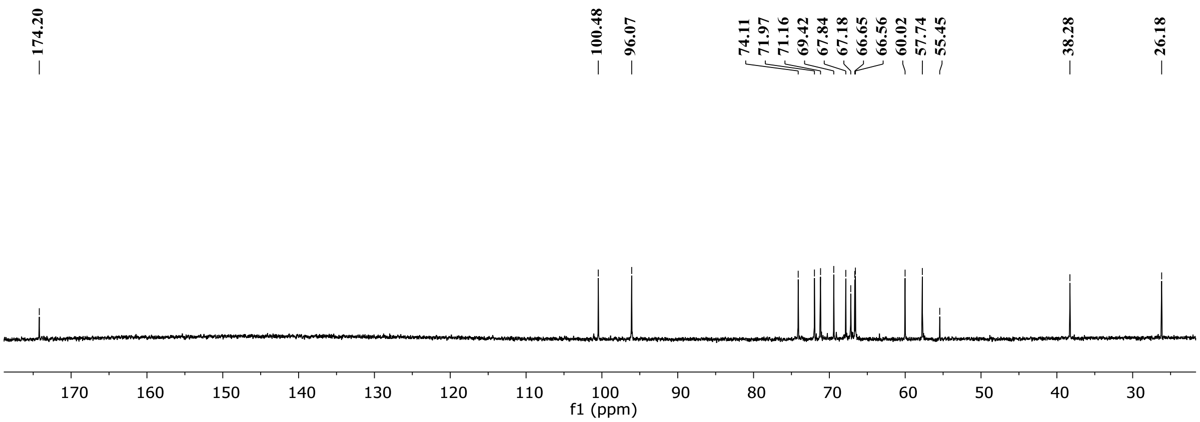
174.20
100.48
96.07
74.11
71.97
71.16
69.42
67.84
67.18
66.65
66.56
60.02
57.74
55.45
38.28
26.18
f1 (ppm)

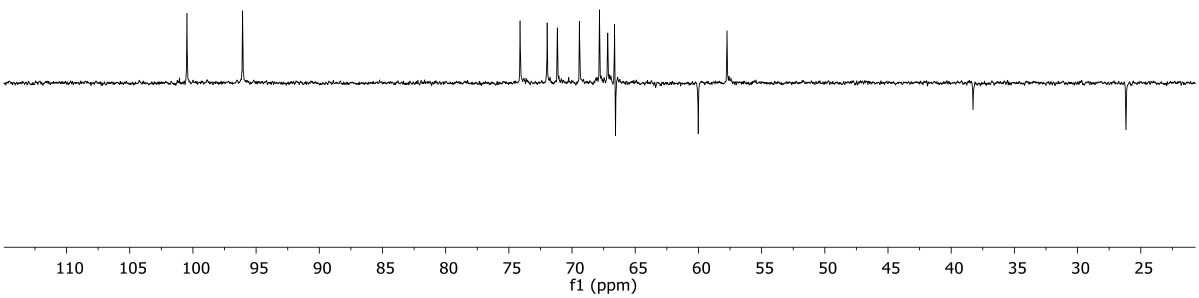
f1 (ppm)

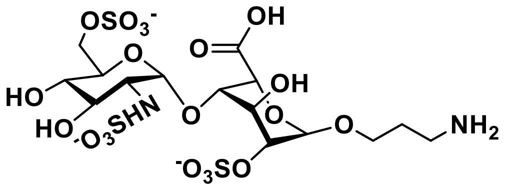
OSO3-
OH
O
HO
HO
-O3SHN
O
OH
O
O
NH2
-O3SO

HD-13

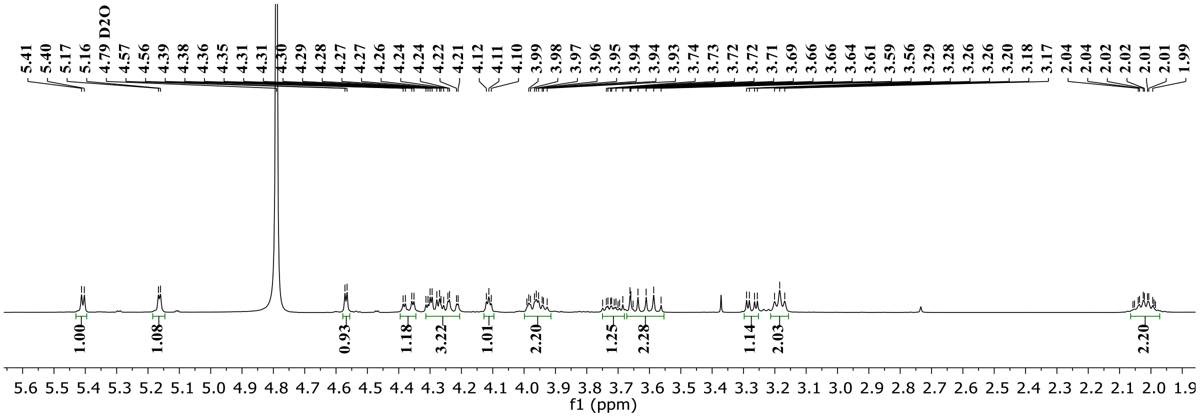
5.41
5.40
5.17
5.16
4.79 D2O
4.57
4.56
4.39
4.38
4.36
4.35
4.31
4.31
4.30
4.29
4.28
4.27
4.27
4.26
4.24
4.24
4.22
4.21
4.12
4.11
4.10
3.99
3.98
3.97
3.96
3.95
3.94
3.94
3.93
3.74
3.73
3.72
3.72
3.71
3.69
3.66
3.66
3.64
3.61
3.59
3.56
3.29
3.28
3.26
3.20
3.18
3.17
2.04
2.04
2.02
2.02
2.01
2.01
1.99
1.00
1.08
0.93
1.18
3.22
1.01
2.20
1.25
2.28
1.14
2.03
2.20
5.6 5.5 5.4 5.3 5.2 5.1 5.0 4.9 4.8 4.7 4.6 4.5 4.4 4.3 4.2 4.1 4.0 3.9 3.8 3.7 3.6 3.5 3.4 3.3 3.2 3.1 3.0 2.9 2.8 2.7 2.6 2.5 2.4 2.3 2.2 2.1 2.0 1.9
f1 (ppm)

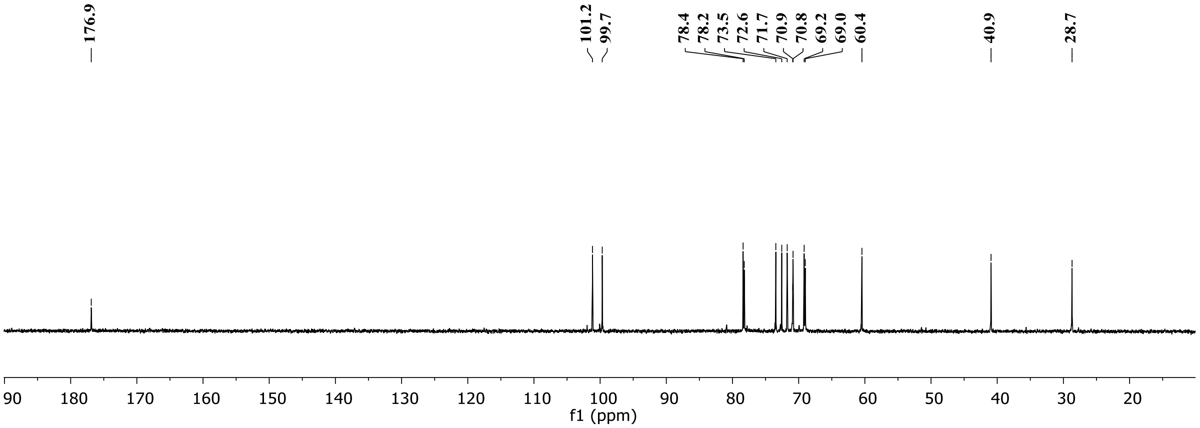
176.9
101.2
99.7
78.4
78.2
73.5
72.6
71.7
70.9
70.8
69.2
69.0
60.4
40.9
28.7
90 180 170 160 150 140 130 120 110 100 90 80 70 60 50 40 30 20
f1 (ppm)

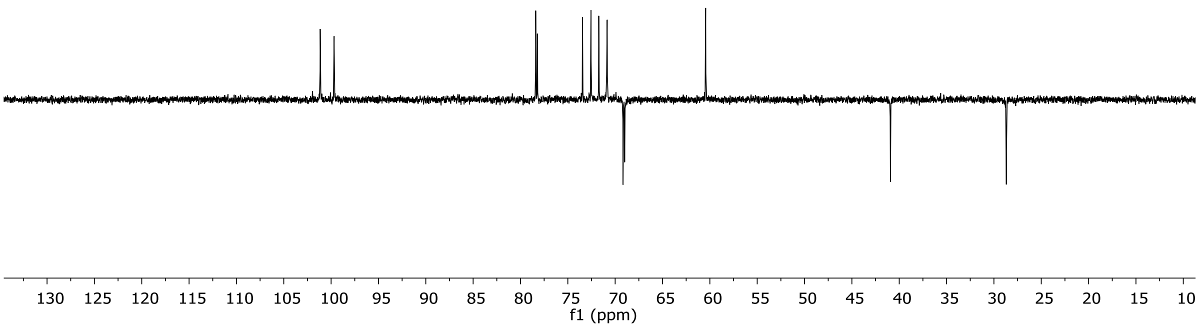
130 125 120 115 110 105 100 95 90 85 80 75 70 65 60 55 50 45 40 35 30 25 20 15 10
f1 (ppm)

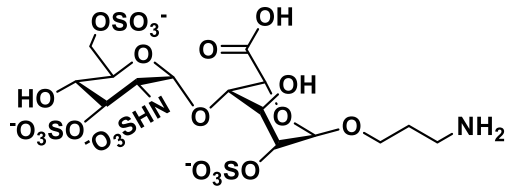

HD-14

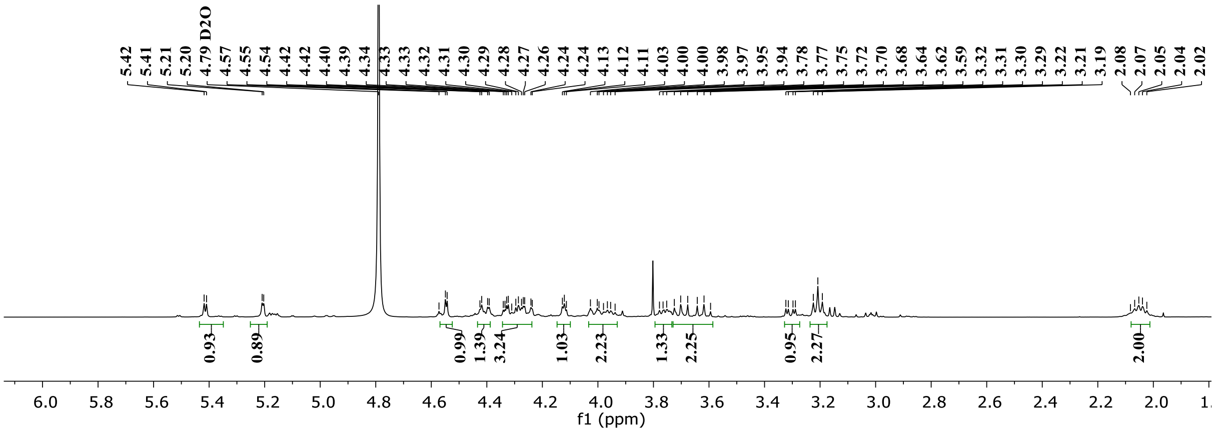
5.42 5.41 5.21 5.20 4.79 D2O 4.57 4.55 4.54 4.42 4.42 4.40 4.39 4.34 4.33 4.33 4.32 4.31 4.30 4.29 4.28 4.27 4.26 4.24 4.24 4.13 4.12 4.11 4.03 4.00 4.00 3.98 3.97 3.95 3.94 3.78 3.77 3.75 3.72 3.70 3.68 3.64 3.62 3.59 3.32 3.31 3.30 3.29 3.22 3.21 3.19 2.08 2.07 2.05 2.04 2.02
0.93 0.89 0.99 1.39 3.24 1.03 2.23 1.33 2.25 0.95 2.27 2.00
f1 (ppm)

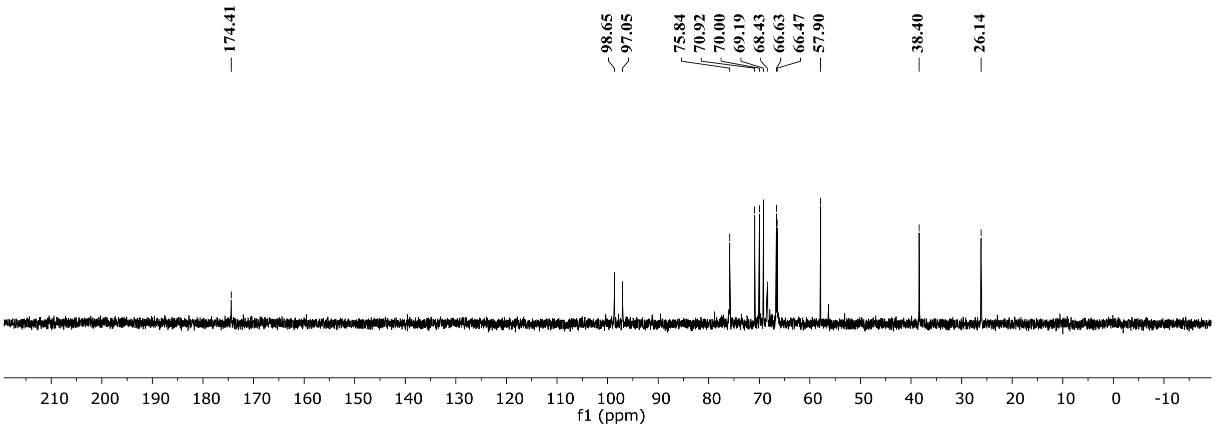
174.41
98.65 97.05
75.84 70.92 70.00 69.19 68.43 66.63 66.47 57.90
38.40
26.14
f1 (ppm)

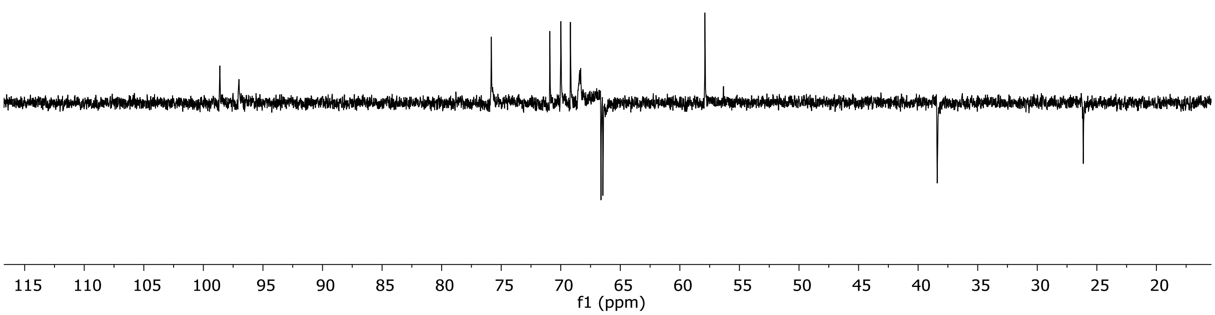
f1 (ppm)
